# Synthesis and One‐Electron Reduction of Donor‐Acceptor‐Stabilized Si^II^–E^II^–Si^II^ Trimetallylenes (E = Ge, Sn, Pb)

**DOI:** 10.1002/anie.6871080

**Published:** 2026-07-03

**Authors:** Chaopeng Hu, Shenglai Yao, Christian Lorent, Matthias Driess

**Affiliations:** ^1^ Department of Chemistry: Metalorganics and Inorganic Materials Technische Universität Berlin Berlin Germany; ^2^ Department of Chemistry: (Bio)Physical Chemistry Technische Universität Berlin Berlin Germany

**Keywords:** acyclic silylene, Ge^I^ radical anion, Sn^I^ radical anion, tetrylenes

## Abstract

Herein, we report isolable donor–acceptor‐stabilized Si^II^–E^II^–Si^II^ trimetallylenes (E = Ge (**2**), Sn (**3**), Pb (**4**); Si^II^ = Mes(cAAC)Si; Mes = 2,4,6‐Me_3_C_6_H_2_; cAAC = C(CH_2_)(CMe_2_)_2_N‐2,6‐*^i^* Pr_2_C_6_H_3_), stabilized by a cyclic alkylaminocarbene attached to the divalent Si atoms. They were synthesized through salt‐metathesis reactions of the dimeric silanylidene anion (Si^II^K)_2_ **1a** and EX_2_ precursors (X = Cl, N(SiMe_3_)_2_). Computational analyses reveal that **2–4** adopt bent donor–acceptor‐stabilized Si^II^–E^II^–Si^II^ frameworks featuring polarized Si–C(cAAC) π interactions together with delocalized Si–E π interactions involving the Lewis‐acidic E^II^ center. This donor–acceptor electronic structure results in small HOMO–LUMO gaps, giving rise to pronounced near‐infrared (NIR) absorption. Remarkably, the one‐electron reduction of **2** and **3** leads to the Ge^I^‐ and Sn^I^‐ radical anions **2^•−^** and **3^•−^**, respectively, thereby highlighting that this donor–acceptor motif can even stabilize heavy Group 14 elements in lower oxidation states.

## Graphical abstract

The first isolable trimetallylenes (Si^II^)_2_E^II^ (E = Ge, Sn, Pb) with p_π_‐type silanylidenyl donors have been synthesized, having donor–acceptor electronic structures that give rise to near‐infrared absorption and enable access to bis(silanylidenyl)‐supported Ge^I^ and Sn^I^ radical anions upon one‐electron reduction with elemental potassium.

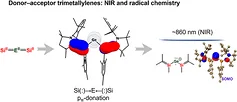

## 1 Introduction

**SCHEME 1:**
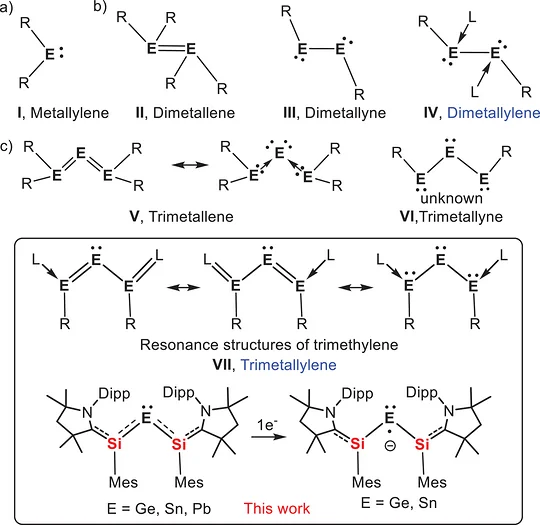
Schematic representations of selected low‐valent Group 14 species: (a) metallylene **I**, (b) dimetallene **II**, dimetallyne **III**, and dimetallylene **IV**, (c) trimetallene **V**, trimetallyne **VI**, and trimetallylene **VII**, the trimetallylenes from this work and their radical anions. Dipp = 2,6‐*^i^* Pr_2_C_6_H_3_; E = Si, Ge, Sn, Pb; L = Lewis base; Mes = 2,4,6‐Me_3_C_6_H_2_; R = organo, silyl, amino.

Metallylenes R_2_E: (E = Si, Ge, Sn, Pb) are heavier analogues of carbenes that exhibit distinctive electronic structures and reactivity patterns (**I**, Scheme 1a). Since the isolation of Lappert's famous stannylene in 1973 [1], tremendous progress has been made in synthesizing various Group 14 metallylenes that mimic transition‐metal species in small‐molecule activation, catalysis, and coordination chemistry [2, 3, 4]. To date, most studies have focused on cyclic derivatives, including *N*‐heterocyclic silylenes, cyclic dialkylsilylenes, and cyclic alkyl(amino)silylenes [5, 6, 7, 8, 9, 10, 11, 12]. By contrast, acyclic silylenes remain comparatively underexplored despite their greater electronic variability [13, 14, 15, 16, 17, 18], which renders them particularly attractive targets for the construction of Group 14 frameworks containing more than one low‐valent center.

Beyond monomeric metallylenes, increasing attention has been directed toward low‐valent Group 14 frameworks featuring E–E σ‐ and π‐bonding interactions. Representative examples include dimetallenes (R_2_E═ER_2_, **II**) and dimetallynes (RE≡ER, **III**), the heavier analogues of alkenes and alkynes, respectively (Scheme 1b) [19]. In addition, Lewis base‐stabilized dimetallylenes (**IV**, Scheme 1b) have been extensively investigated as donor–acceptor molecules containing reactive E–E bonds [20, 21, 22, 23, 24, 25, 26, 27, 28]. Together, these studies demonstrate that Group 14 frameworks containing more than one low‐valent center profoundly affect the bonding and reactivity of low‐valent Group 14 compounds. They also raise a central question that remains largely unexplored: How does the incorporation of three connected low‐valent centers influence the electronic structure and reactivity of low‐valent Group 14 chains?

In 1999, Wiberg and co‐workers demonstrated the feasibility of triatomic unsaturated heavy Group 14 chains through the isolation of a bent tristannaallene Sn[(Sn(Si*^t^* Bu_3_)_2_]_2_ (**V**, Scheme 1c) [29], which features a formally zero‐valent tin center supported by two stannylene units and was later described as a stannylone [30]. Subsequent studies by Kira and Iwamoto extended this concept to silicon and germanium molecules, revealing dynamic bonding and conformationally flexible silylone and germylone motifs [31, 32, 33, 34]. Further developments have provided access to other types of silylones [35] and to the heavier Sn^0^ and Pb^0^ congeners, stannylones and plumbylones [36, 37]. Additional precedents include the trigermenyl anion reported by Power and co‐workers [38], NHC‐stabilized disilenyl metallylenes reported by Scheschkewitz and co‐workers [39, 40], and divinyl tetrylenes reported by Rivard and co‐workers [41, 42], which represent related unsaturated low‐valent Group 14 chains containing low‐valent centers. Despite these advances, well‐defined trimetallyne (**VI**) and donor–acceptor‐stabilized trimetallylene (**VII**) chains remain unknown, leaving a fundamental gap in understanding how incorporation of three divalent centers modulates bonding and reactivity in low‐valent Group 14 chemistry.

**SCHEME 2:**
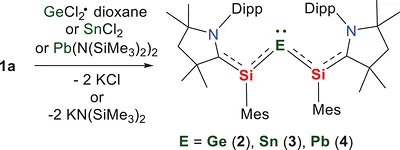
Synthesis of the trimetallylenes **2–4**. Dipp = 2,6‐*^i^* Pr_2_C_6_H_3_.

Building on our previous studies of Group 14 metallylones stabilized by bis(silylenes) ligands [43, 44, 45, 46, 47, 48, 49, 50, 51], we envisioned that a nucleophilic silanylidenyl transfer strategy could provide access to Si^II^–E^II^–Si^II^ trimetallylene frameworks containing three divalent Group 14 centers. Herein, we report the synthesis of **2–4** employing the silanylidene anion **1a** as a nucleophilic silanylidenyl source (Scheme 2). They feature p_π_‐type silanylidenyl donors interacting with a Lewis‐acidic divalent Group 14 center E, thereby generating a donor–acceptor electronic structure. This electronic configuration gives rise to near‐infrared (NIR) absorption and also enables access to monovalent radical anions upon one‐electron reduction.

## 2 Results and Discussion

### 2.1 Synthesis and Characterization of **1** and **2–4**

Following the strategy reported by Roesky and co‐workers for a Tip‐substituted silanylidene anion (Tip = 2,4,6‐*^i^* Pr_3_C_6_H_2_) [52], the mesityl‐substituted derivative (Si^II^K)_2_ **1a** (Si^II^ = Mes(cAAC)Si; Mes = 2,4,6‐Me_3_C_6_H_2_; cAAC = C(CH_2_)(CMe_2_)_2_N‐2,6‐*^i^* Pr_2_C_6_H_3_) was prepared as a red solid. Compound **1a** is readily soluble in hexane and exhibits a symmetric solution structure in C_6_D_6_ (Figure S2), analogous to the Tip‐substituted silanylidene anion [52]. The ^2^ ^9^Si{^1^H} NMR spectrum of **1a** displays a singlet at *δ* = 42.2 ppm in C_6_D_6_, shifted downfield relative to the Tip‐substituted analogue (*δ* = 26.6 ppm in C_6_D_6_) [52]. Likewise, the carbene carbon resonance in the ^1^ ^3^C{^1^H} NMR spectrum appears at *δ* = 194.2 ppm, close to that of the corresponding Tip‐substituted derivative (*δ* = 195.1 ppm) [52]. Treatment of **1a** with [2.2.2]cryptand afforded the solvent‐separated ion pair (SSIP) **1b**, which is poorly soluble in hexane and toluene but dissolves readily in THF. The ^2^ ^9^Si{^1^H} NMR resonance of **1b** appears at δ = 60.5 ppm in THF‐*d_8_*, shifted downfield relative to **1a** (δ = 49.5 ppm in THF‐d8). Such downfield shifts are consistent with the formation of solvent‐separated ion pairs observed for related silenyl lithium [53]. In contrast, the comparable diffusion coefficients obtained from the 2D DOSY ^1^H NMR spectra (7 × 10^−^^10^ m^2^ s^−^^1^ for **1a** vs. 6 × 10^−^^10^ m^2^ s^−^^1^ for **1b**; Figures S8, S13, and S14) suggest comparable hydrodynamic sizes under these conditions, consistent with substantial solvation and possible partial dissociation of **1a** in THF‐d8.

Single‐crystal x‐ray diffraction analyses confirm that **1a** adopts a dimeric structure, whereas **1b** exists as an SSIP. The Si–C bond metrics of **1a** [Si(1)–C(1) = 1.790(3) Å; Si(1)–C(3) = 1.946(4) Å] and **1b** [Si(1)–C(1) = 1.7875(12) Å; Si(1)–C(3) = 1.9509(13) Å] closely resemble those reported for the Tip‐substituted silanylidene anion [Si(1)–C(1) = 1.793(2) Å; Si(1)–C(3) = 1.946(2) Å] (Table S1), indicating a similar bonding situation. The Si–C(cAAC) distances in these silanylidene anions are shorter than a typical Si–C single bond (1.91 Å), but slightly longer than the reference Si═C double‐bond distance (1.74 Å) [54, 55] and those of representative structurally characterized silene‐type compounds (**VIII–XI**, Table 1) [56, 57, 58, 59]. These comparisons support a polarized Si–C(cAAC) donor–acceptor interaction with limited localized Si═C double‐bond character rather than a classical silene‐type formulation. DFT calculations further reveal a large singlet–triplet energy gap of 28.6 kcal mol^−^^1^ for the mesityl‐substituted silanylidene anion, together with two localized nonbonding lone pairs at silicon (Figure S43), consistent with its nucleophilic low‐valent silicon character.

**TABLE 1:** Comparison of Si–C bond lengths in **1–4** and silene‐type compounds **VIII–XI**, together with Pyykkö’s standard values for Si–C single^a^ and Si═C double^b^ bonds.

Si═C^a^		VIII	IX	X	XI	
1.74 Å	Si(1)–C(1)	1.746(2)	1.773(3)/1.778(3)	1.735(2)	1.725(2)	
Si–C^b^		**1a**	**1b**	**2**	**3**	**4**
1.91 Å	Si(1)–C(1)	1.790(3)	1.7875(12)	1.8230(13)	1.819(3)	1.805(5)
	Si(2)–C(2)	1.796(3)		1.8230(13)	1.814(3)	1.800(4)
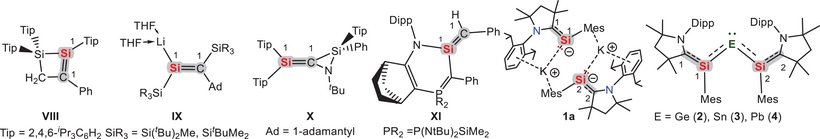						

Treatment of **1a** with one molar equivalent of GeCl_2_·dioxane, SnCl_2_, or Pb(N(SiMe_3_)_2_)_2_ in THF afforded deep‐green solutions, from which the trimetallylenes (Si^II^)_2_E (**2–4**; E = Ge, Sn, Pb) were isolated in 91%, 90%, and 76% yields, respectively (Scheme 2). The ^1^H NMR spectra of **2–4** indicate symmetric bis(silanylidenyl)metallylene structures in solution. The ^2^ ^9^Si{^1^H} NMR resonances at δ = 91.1 (**2)**, 121.0 (**3**), and 309.1 ppm (**4**) are markedly downfield relative to **1a**, reflecting increased deshielding with increasing atomic number of the central Group 14 element. The pronounced downfield shift of the ^2^ ^9^Si{^1^H} NMR resonances from **2** to **4** is consistent with the increasing influence of the heavier central Group 14 atom. Similar trends have been observed in related terphenyl‐substituted silylmetallylenes [60, 61, 62]. This deshielding likely reflects heavy‐atom‐induced relativistic effects, including heavy‐atom‐on‐light‐atom (HALA) and spin–orbit HALA contributions [63]. The ^1^ ^1^ ^9^Sn{^1^H} NMR signal of **3** at *δ* = 1738 ppm is significantly deshielded compared with that of Lappert's stannylene (*δ* = 768 ppm) [64, 65] and a diphosphinostannylene (*δ* = 1467 ppm) [66, 67], yet remains more shielded than a diborylstannylene (*δ* = 4755 ppm) [14]. This trend is consistent with a weak π‐donor character of the silanylidenyl group toward Sn^II^.

**FIGURE 1:**
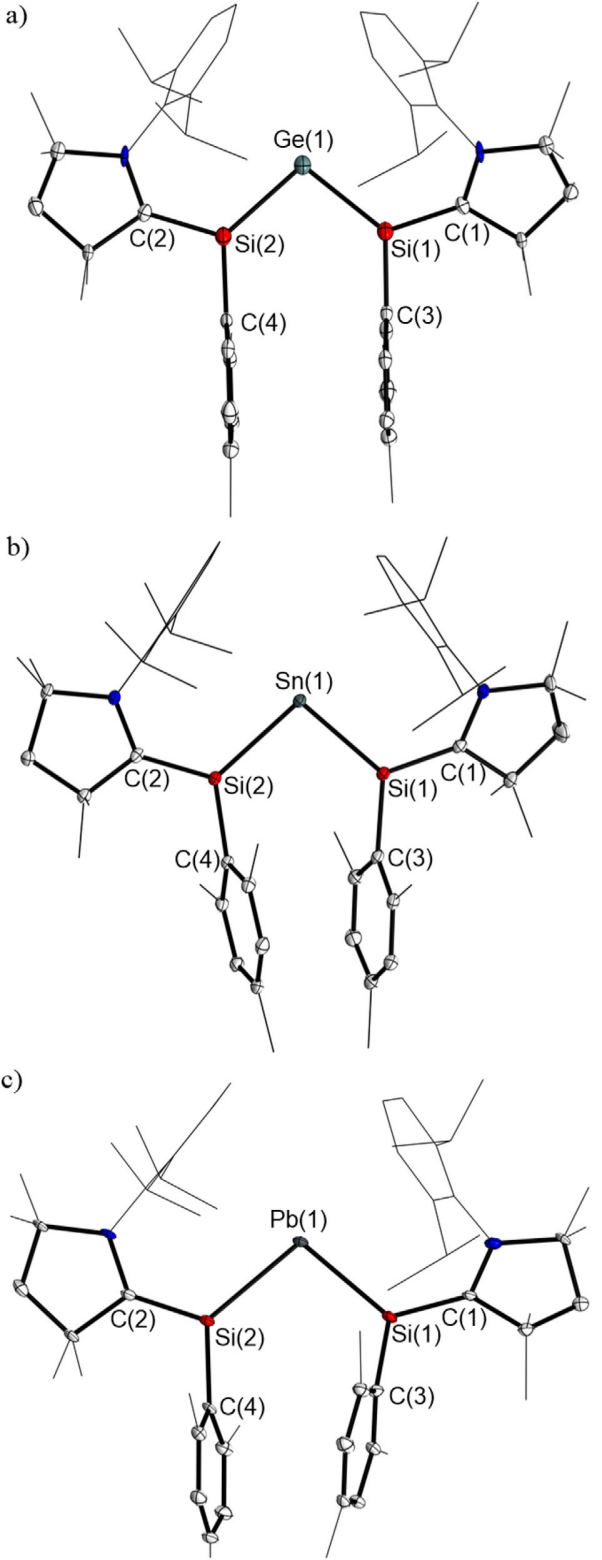
Molecular structures of trimetallylenes (a) **2**, (b) **3**, and (c) **4**, with thermal ellipsoids at the 30% probability level [69]. Hydrogen atoms are omitted for clarity.

Single crystals of **2–4** suitable for sc‐XRD analyses were obtained from hexamethyldisiloxane solutions at −30°C (Figure 1). The structures reveal bent Si–E–Si frameworks that are sterically protected by the Dipp substituents of the cAAC ligands. The E(1)–Si(1) and E(1)–Si(2) distances are 2.3520(4)/2.3520(4) Å for **2**, 2.5660(8)/2.5609(8) Å for **3**, and 2.6307(11)/2.6438(12) Å for **4**, respectively. These values indicate progressively weaker partial Si–E π‐donor contributions from Ge to Pb, in line with the increasing E–Si bond lengths and the expected decrease in orbital overlap (reference E–Si/E═Si bond lengths: 2.37/2.18 Å for E═Ge, 2.56/2.37 Å for E═Sn, and 2.60/2.42 Å for E═Pb) [54, 55]. The Si–C(cAAC) distances in **2–4** [Si(1)–C(1)/Si(2)–C(2) = 1.8230(13)/1.8230(13) Å for **2**, 1.819(3)/1.814(3) Å for **3**, and 1.805(5)/1.800(4) Å for **4**] are longer than those in **1a**/**1b** and also longer than those of representative silene‐type Si═C bonds (**VIII–XI**, Table 1) [56, 57, 58, 59]. Nevertheless, they remain shorter than the reference Si–C single bond (1.91 Å). This indicates that the Si–C(cAAC) multiple‐bond character is lowered upon formation of the Si–E–Si framework, while delocalized Si–C(cAAC) π interactions remain embedded within the overall donor–acceptor chain. The sum of bond angles around each silicon center is close to 360°, indicating an approximately trigonal coordination sphere (Figure 1). Notably, the Si–Pb–Si angle of 98.58(3)° in **4** is substantially smaller than that of the silyl‐supported plumbylene Pb(Hyp)_2_ (113.6 and 115.7°; Hyp = Si(SiMe_3_)_3_) [68], indicating a distinct electronic influence of the silanylidene donors relative to silyl substituents. Taken together, these structural data support a highly polarized donor–acceptor bonding description featuring delocalized π interactions across the Si–E–Si framework rather than localized Si═C double bonds.

The steric properties of the silanylidenyl ligand were quantified using the percent buried volume (%*V*_bur_) [70]. A high %*V*_bur_ value of 94.3% was obtained at the Pb center in **4**, significantly exceeding that of the Hyp (73.8%) [68]. For comparison, values for related molecules (Lappert's stannylene, diphosphinostannylene, diborylstannylene, and **3**) range from 77.1% to 96.2% (Table S9 and Figures S44 and S49). These results demonstrate that the silanylidenyl ligand provides massive steric protection, which is important for stabilizing low oxidation states of reactive Group 14 centers.

The intense colors of **2–4** prompted investigation of their electronic absorption properties. All three compounds exhibit strong NIR absorption bands at 857, 861, and 875 nm, with molar absorption coefficients ε of 3.4 × 10^4^, 3.1 × 10^4^, and 2.2 × 10^4^ M^−^^1^ cm^−^^1^, respectively (Figures S33 and S34). In contrast, **1a**/**1b** and previously reported bis(silylenes) [71, 72, 73, 74, 75, 76], lacking a Lewis‐acidic spacer, show no absorption in the NIR region, highlighting the particular electronic incident of the Si–E–Si frameworks. Time‐dependent DFT (TD‐DFT) calculations reproduce the experimental absorption bands at 793, 828, and 871 nm (Figures S50–S52). The lowest‐energy transition (S_0_ → S_1_) is assigned to excitation from polarized p_π_‐type lone pairs at silicon to the vacant valence p orbitals of the central E atom. Electron–hole analyses [77, 78, 79] further confirm this donor–acceptor transition character (Figures S50–S52). Together, these results support the assignment of the NIR absorption originating from donor–acceptor interactions between the p_π_‐type silanylidenyl donors and the Lewis‐acidic central atom. Consistently, **2–4** exhibit relatively small HOMO–LUMO gaps of 1.90, 1.93, and 1.89 eV, respectively (Figure S54). This feature distinguishes **2–4** from related disilenyl metallylenes and trimetallenes lacking an intrinsic donor–acceptor framework.

### 2.2 Electronic Structures of the Trimetallylenes **2–4**

To gain deeper insight into the electronic structures of **2–4**, DFT calculations were performed at the B3LYP‐D3/def2‐SVP level. The optimized geometries are in good agreement with the experimentally determined molecular structures. The calculated singlet–triplet energy gaps are 15.8, 17.7, and 17.9 kcal mol^−^^1^ for **2–4**, respectively. These results indicate electronically well‐defined closed‐shell ground states despite the presence of three low‐valent Group 14 centers.

**FIGURE 2:**
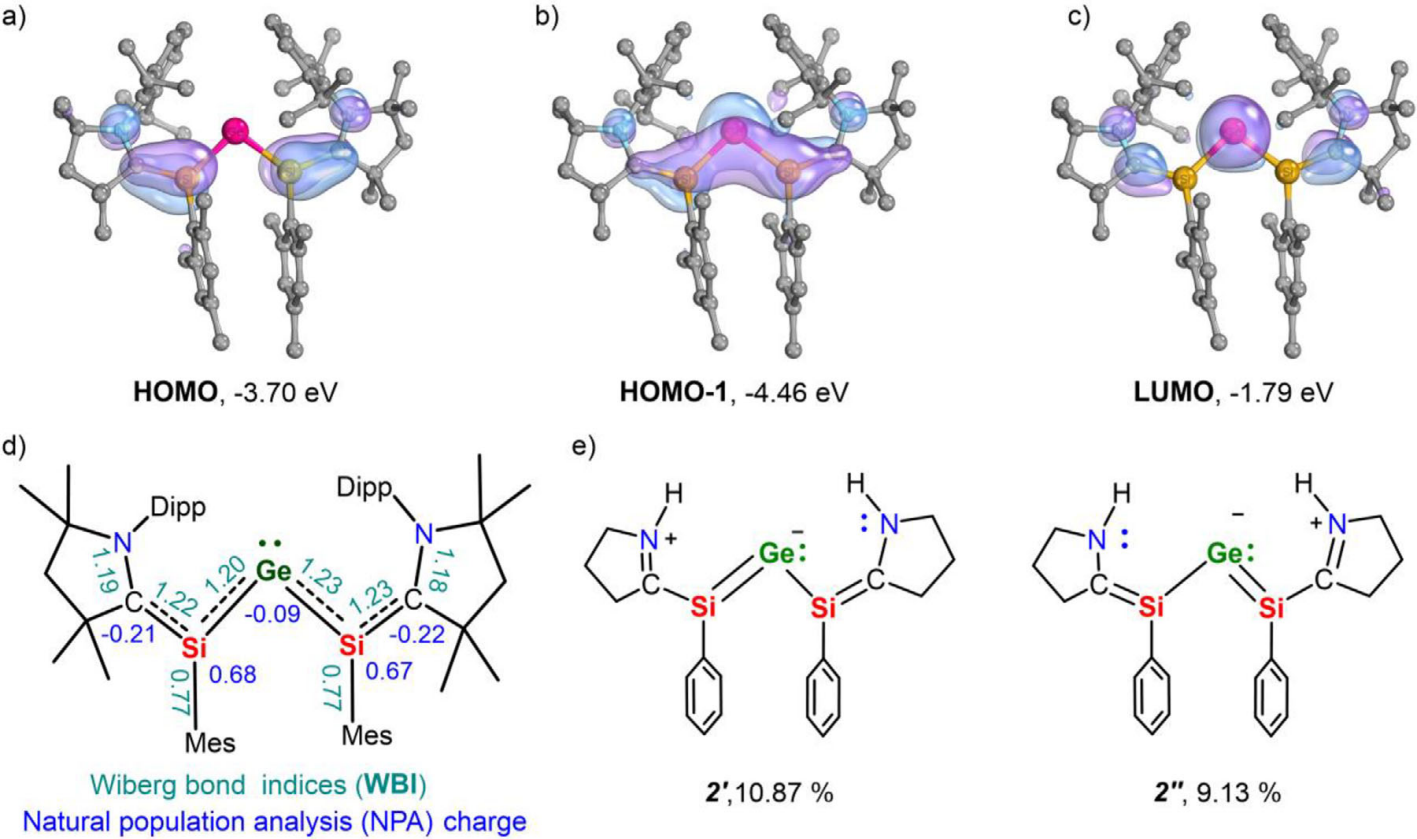
(a) HOMO of **2**, (b) HOMO‐1 of **2**, and (c) LUMO of **2**. (d) Selected Wiberg bond indices (WBIs) (green) and NPA charges (blue) for **2**. (e) The two NRT (natural resonance theory) major resonance structures (each > 5% contribution) **2′** and **2″** for a simplified model of **2**.

Frontier molecular orbital analyses revealed a highly polarized donor–acceptor electronic structure. In compound **2**, the occupied frontier orbitals (HOMO, HOMO‐1) are dominated by delocalized π interactions extending over the C(cAAC)–Si–Ge–Si–C(cAAC) framework (Figure 2a,b), whereas the LUMO is predominantly localized on the vacant Ge 4p orbital, with minor contributions from the antibonding C–N π* orbitals of the cAAC ligands, which exhibit slight delocalization onto the Si centers (Figure 2c). Similar orbital patterns are observed for **3** and **4** (Figure S53), although the Si–E π‐donor interactions become progressively weaker from Ge to Pb, consistent with elongation of the E–Si bonds and decreasing orbital overlap.

This electronic structure is further supported by natural bond orbital (NBO) analyses. The calculated Wiberg bond indices (WBIs) for the E–Si bonds decrease from 1.20/1.23 in **2** to 1.03/1.02 in **4** (Figure 2d and Table S13), indicating progressively weaker Si–E π‐donor contributions from Ge to Pb. At the same time, substantial charge polarization is observed across the Si–E–Si frameworks according to the NPA charges (Table S14), consistent with donor–acceptor interactions between the silanylidenyl donors and the central Group 14 atom. Second‐order perturbation analysis likewise identifies donor–acceptor interactions between the occupied C–Si π orbitals and the vacant orbitals at the central E atom, with stabilization energies that decrease from Ge to Pb (Figure S56).

The bonding situation was also analyzed by natural resonance theory (NRT) and energy decomposition analysis (EDA) calculations using a simplified model of **2** as a representative molecule of the series, since the electronic structure trends across **2–4** are closely related. Importantly, the two major calculated resonance contributors do not support a description based on two localized Si═C double bonds flanking an isolated metallylene center (**2′** and **2″**, Figure 2e). This result is consistent with the experimentally observed Si–C(cAAC) bond distances (Table 1), which are longer than those of typical silenes but remain significantly shorter than conventional Si–C single bonds. Additional insight was obtained from EDA calculations on a truncated model of **2** using both charged closed‐shell and neutral open‐shell fragmentation schemes (Table S15). The large orbital interaction terms obtained from both fragmentation approaches support substantial covalent contributions to the Si–Ge interactions within an overall polarized donor–acceptor framework [80, 81].

Taken together, the combined experimental and computational analyses support a highly polarized donor–acceptor Si–E–Si framework with delocalized π interactions across the C(cAAC)–Si–E–Si–C(cAAC) chain. The bonding situation cannot be adequately represented by a single Lewis formulation and is best described as a donor–acceptor‐stabilized trimetallylene‐type framework with contributions from dimetallenyl metallylene and related polarized resonance descriptions.

### 2.3 Reactivity of the Trimetallylenes **2–4**

The reactivity of trimetallylenes **2** and **3** was probed toward a series of small molecules and chalcogen‐transfer reagents. Compound **3** is inert toward CO, H_2_, or NH_3_. By contrast, treatment of **3** with CO_2_ resulted in fast Si–Sn bond cleavage and formation of plenty of unidentified products. Reactions of **2** and **3** with several chalcogen‐transfer reagents, including N_2_O, Me_3_P═Se, and *^t^* Bu_3_P═Te, led to inseparable mixtures of unidentified products. Similarly, reactions of **2** and **3** with elemental sulfur in toluene led to cAAC═S [82] as the only isolable product. Attempts to generate the corresponding radical cation by oxidation of **3** using silver reagents (AgBF_4_, AgOTf, AgNTf_2_, and AgSbF_6_; OTf = triflate) or NOPF_6_ afforded merely complex mixtures, consistent with the limited oxidative stability of the trimetallylene framework. Collectively, these observations suggest that the donor–acceptor‐stabilized Si–E–Si framework preferentially undergoes ligand‐centered degradation or framework decomposition under strongly oxidizing conditions rather than clean chalcogen‐atom transfer at the central Group 14 atom E.

**SCHEME 3:**
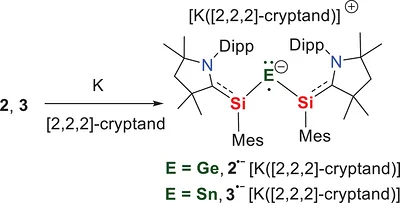
One‐electron reduction of trimetallylenes **2** and **3**. Dipp = 2,6‐*i*Pr_2_C_6_H_3_.

Fortunately, the one‐electron reduction of **2** and **3** led to isolable products. Treatment of **2** with elemental potassium in THF at room temperature in the presence of [2.2.2]‐cryptand afforded a deep purple solution of **2^•−^** [K([2.2.2]‐cryptand)], which could be isolated in 65% yield after recrystallization (Scheme 3). As expected, the resulting product is NMR silent, and only signals corresponding to the diamagnetic [K([2.2.2]‐cryptand)] cation are observed in the ^1^H NMR spectrum (Figure S30), consistent with the formation of a paramagnetic anion. The latter is highly reactive and rapidly decolorizes upon exposure to trace amounts of O_2_ and moisture. Similarly, reduction of **3** yielded the corresponding radical anion **3^•−^** [K([2.2.2]‐cryptand)] as black crystals (Figure S30). Owing to its increased lability, however, isolation of an analytically pure sample failed, as shown by Electron Paramagnetic Resonance (EPR) measurements (see below), and an accurate yield could not be established despite extensive purification attempts.

The UV–vis spectra of **2^•−^** [K([2.2.2]‐cryptand)] and **3^•−^** [K([2.2.2]‐cryptand)] exhibit multiple absorption bands extending into the NIR region (Figures S35 and S36), consistent with low‐energy electronic transitions associated with a delocalized singly occupied molecular orbital (SOMO). EPR spectroscopy further supports the assignment of **2^•−^** [K([2.2.2]‐cryptand)] and **3^•−^** [K([2.2.2]‐cryptand)] as Ge‐ and Sn‐ radical anions, respectively (Figures S39 and S40).

Notably, **2^•−^** [K([2.2.2]‐cryptand)] represents a well‐defined example of a two‐coordinate Ge^I^ radical anion supported by two silanylidenyl donors. Likewise, although the corresponding Sn species **3^•−^** [K([2.2.2]‐cryptand)] exhibits increased lability, the spectroscopic features of **3^•−^** are in accordance with a Sn^I^ radical anion. While previously isolated Ge^I^ and Sn^I^ radical anions contain a xanthene‐based diamido ligand [83], the successful generation of **2^•−^** [K([2.2.2]‐cryptand)] and **3^•−^** [K([2.2.2]‐cryptand)] is noteworthy because the lower electronegativity of silicon (χ_Si_ = 1.90) renders the stabilization of Ge^I^ and Sn^I^ radical anions inherently more challenging. By contrast, reduction of the Pb analogue **4** under identical conditions resulted exclusively in Si–Pb bond cleavage, affording the silanylidene anion **1b** and elemental Pb. This behavior suggests that the corresponding Pb^I^ radical anion is significantly less stable than its lighter congeners, thereby delineating the stability limit of this trimetallylene platform.

**FIGURE 3:**
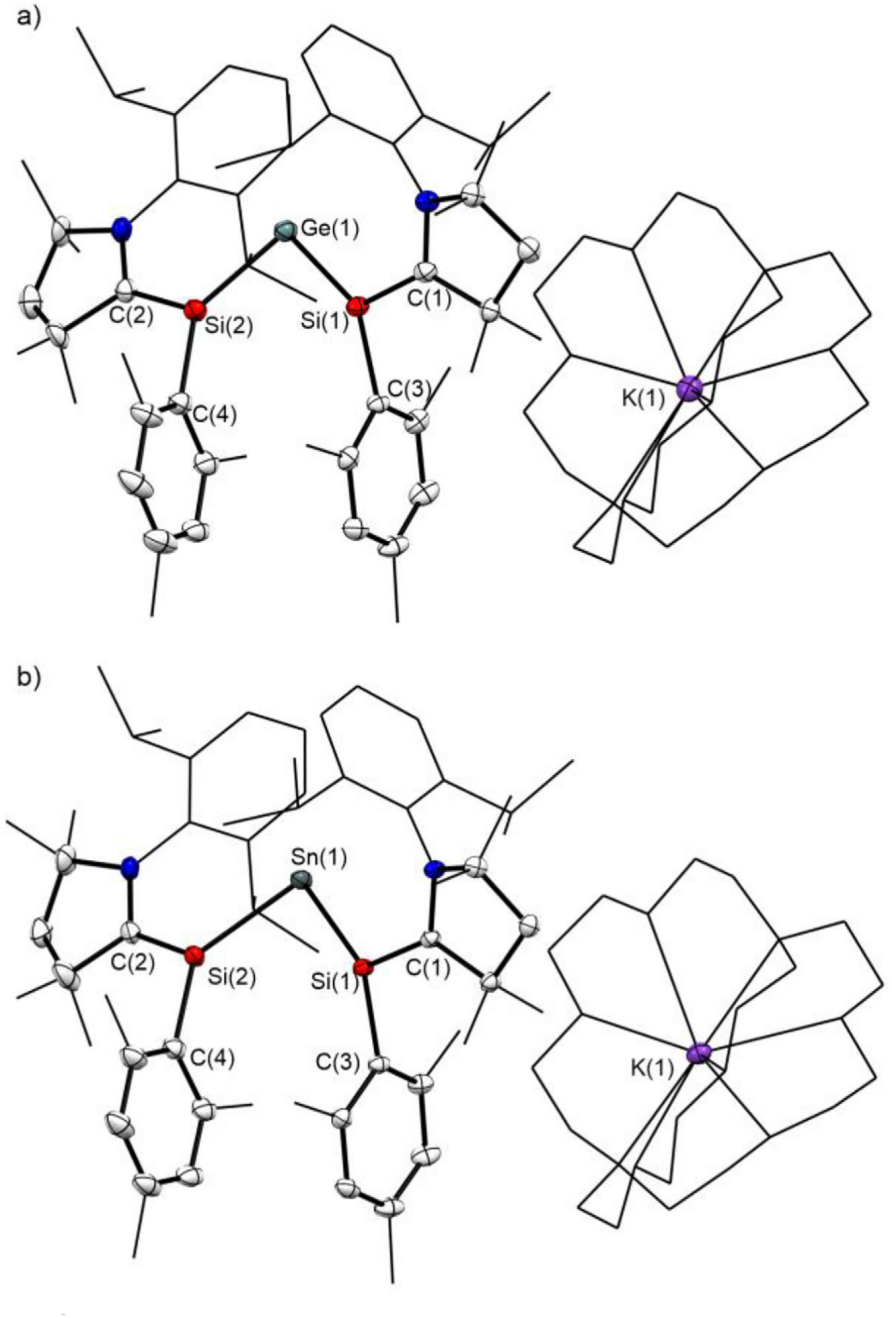
Molecular structures of (a) **2^•−^** [K([2.2.2]‐cryptand) and (b) **3^•−^** [K([2.2.2]‐cryptand)], with thermal ellipsoids at the 30% probability level [69]. Hydrogen atoms are omitted for clarity.

Both **2^•−^** [K([2.2.2]‐cryptand)] and **3^•−^** [K([2.2.2]‐cryptand)] display poor solubility in hexane and diethyl ether (Et_2_O). Single‐crystals suitable for sc‐XRD analysis were obtained from mixed THF/Et_2_O/pentane solutions at −30°C. They crystallize in the monoclinic space group *P*2_1_/*n* (Figure 3). In both molecular structures, the Si(1)–C(1) (1.791(4) and 1.7885(16) Å) and Si(2)–C(2) bonds (1.807(4) and 1.7931(18) Å) retain partial double bond character, whereas the Si(1)–C(3) (1.918(3) and 1.9114(17) Å) and Si(2)–C(4) (1.914(4) and 1.9133(19) Å) distances remain characteristic of Si–C single bonds (Table 1) [54, 55]. Compared with their neutral precursors, the Si(1)–E(1)–Si(2) bond angles change only slightly upon one‐electron reduction (E = Ge: 100.41(4)° vs. 99.00°; E = Sn: 98.86(15)° vs. 98.68(3)°), indicating that the overall trimetallylene framework is largely retained in the radical anions. Together, these results support the formulation of **2^•−^** [K([2.2.2]‐cryptand)] and **3^•−^** [K([2.2.2]‐cryptand)] as structurally characterized radical‐anion species.

**FIGURE 4:**
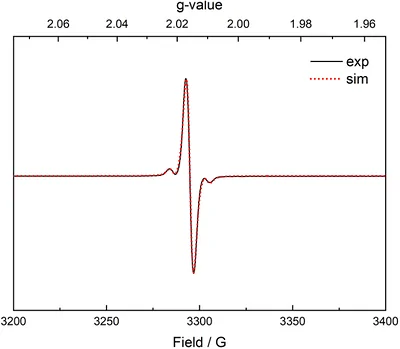
CW X‐band EPR spectrum of **2^•−^** [K([2.2.2]‐cryptand)] recorded in THF at 298 K.

The continuous‐wave (CW) X‐band EPR spectrum of **2^•−^** [K([2.2.2]‐cryptand)], recorded in THF at 298 K, displays an S = 1/2 signal with an isotropic *g* value (g) of 2.016 (Figure 4). This value is slightly smaller than that reported for the transient digermene radical anion (*g* = 2.027) by Baumgartner et al. [84], but larger than the free‐electron value (*g*_e_ = 2.0023) [85], consistent with appreciable Ge involvement in the SOMO and associated spin–orbit coupling effects. The remaining satellite features expected from ^73^Ge hyperfine coupling, however, are not fully resolved, most likely due to overlap with the intense central signal, together with the low natural abundance and small nuclear magnetic moment of the ^73^Ge nucleus [86]. Notably, this is in line with a stronger hyperfine coupling to ^29^Si (*I* = 1/2), with a(^29^Si) = 18.1 G. This coupling is substantially larger than that reported for the transient digermene radical anion (a(^29^Si) = 7.8 G) and approaches the value observed for a disilene radical anion (a(^29^Si) = 24.5 G) [87]. These data indicate significant delocalization of spin density from the Ge onto the adjacent Si atoms in **2^•−^** [K([2.2.2]‐cryptand)].

Due to the typical accelerated spin relaxation of heavy metal‐type radicals, the EPR spectrum of **3^•ˉ^** [K([2.2.2]‐cryptand)] was recorded in frozen solution at 10 K (Figure S40). The dominant spectral component is characterized by a rhombic *g* tensor with principal values of *g*_x_ = 2.08, *g*_y_ = 2.02, *g*_z_ = 1.99, *g*_av_ = 2.03, together with large anisotropic hyperfine coupling to ^117,119^Sn nuclei (I = 1/2, natural abundances 7.7% and 8.6%, respectively), with coupling constants of *A*_x_ = 561 MHz, *A*_y_ = 623 MHz, and *A*_z_ = 988 MHz. The observed saturation behavior further supports the assignment of this signal to a heavy‐element radical species (Figure S42). Although ^29^Si hyperfine interactions may also be present, they are not resolved, most likely because of the broadness of the spectral features. Nevertheless, the dominant components are sufficiently well resolved to allow reliable extraction of the principal *g* values and ^117,119^Sn hyperfine coupling parameters. Compared with previously reported Sn^I^ radical species, including the xanthene‐based diamido Sn^I^ radical anion (*g*_x_ = 1.88, *g*_y_ = 1.91, *g*_z_ = 1.98, *g*_av_ = 1.92 and *A*_x_ = 1411 MHz, *A*_y_ = 1426 MHz, and *A*_z_ = 1287 MHz) and the monosubstituted neutral Sn^I^ radical reported by Tan, Ye, and Zhao (*g*_x_ = 1.96, *g*_y_ = 1.90, *g*_z_ = 1.58, *g*_av_ = 1.81 and *A*_x_ = 1412 MHz, *A*_y_ = 1873 MHz, and *A*_z_ = 698 MHz), **3^•−^** [K([2.2.2]‐cryptand)] exhibits distinct average g‐values, g‐tensor anisotropy and smaller Sn hyperfine coupling constants. These differences point to a markedly different electronic environment at the Sn center and a stronger delocalization of the spin towards the Si atoms. Taken together, the EPR data support the assignment of **3^•−^** [K([2.2.2]‐cryptand)] as Sn‐dominant radical anion with appreciable ligand delocalization.

The redox properties of **2** and **3** were also investigated by cyclic voltammetry (CV). Both compounds show reversible redox waves at ΔE_1/2_ = −2.51 V and −2.52 V, respectively, referenced to the Fc/Fc^+^ couple (Figures S37 and S38), consistent with one‐electron redox events. In agreement with this interpretation, chemical oxidation of **2^•−^** [K([2.2.2]‐cryptand)] with one molar equivalent of FcBAr^F^_4_ (Ar^F^ = 3,5‐(CF_3_)_2_C_6_H_3_) regenerates neutral **2**, further corroborating the electrochemical reversibility (Figure S32).

**FIGURE 5:**
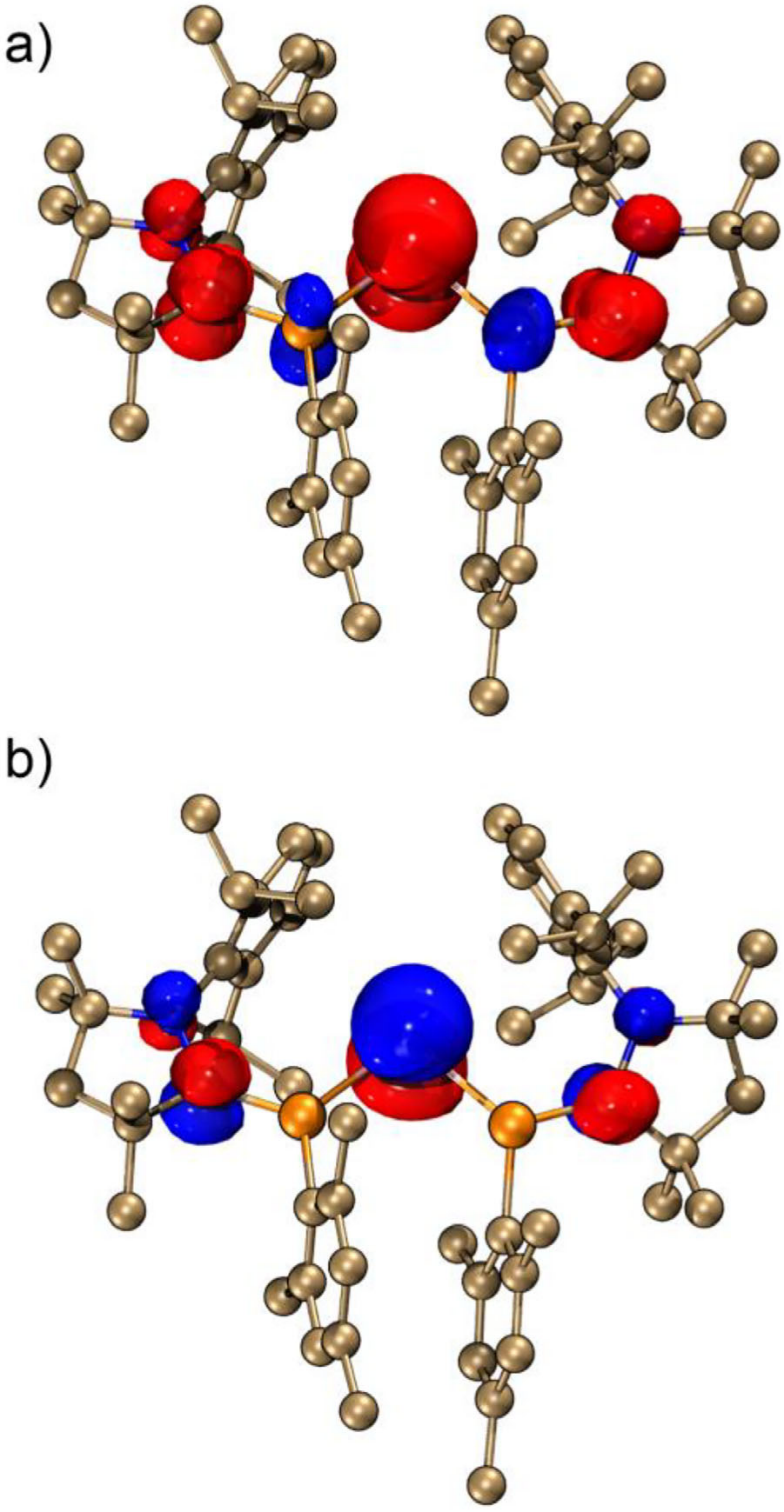
(a) Spin density distribution of **2^•−^** (isovalue = 0.003); (b) SOMO of **2^•−^** (isovalue = 0.05).

To gain further insight into the electronic structure of **2^•−^** [K([2.2.2]‐cryptand)] and **3^•−^** [K([2.2.2]‐cryptand)], DFT calculations were carried out. The optimized geometries are in good agreement with the experimentally determined molecular structures. The calculated spin‐density distributions indicate that the unpaired electron is predominantly localized at the central Ge and Sn atoms, respectively, with partial delocalization onto the adjacent Si atoms and the cAAC framework (Figure 5 and Figure S59). Consistently, the SOMOs exhibit similar spatial distributions. These computational results are consistent with the EPR data and support the assignment of **2^•−^** [K([2.2.2]‐cryptand)] and **3^•−^** [K([2.2.2]‐cryptand)] as Ge‐ and Sn‐ radical anions stabilized by two silanylidenyl donor units.

## 3 Conclusion

In summary, isolable donor–acceptor‐stabilized trimetallylene chains (Si^II^)_2_E (**2–4**, E = Ge, Sn, Pb) have been synthesized from the silanylidene anion **1a**. These compounds adopt bent Si–E–Si frameworks in which two p_π_‐type silanylidenyl donors interact with a Lewis‐acidic divalent Group 14 center through delocalized Si–E π interactions. The donor–acceptor electronic structure gives rise to pronounced NIR absorption and also enables access to Ge^I^ / Sn^I^ radical anions upon chemical one‐electron reduction. Overall, these findings establish acyclic trimetallylenes as a new class of donor–acceptor‐stabilized subvalent main‐group species capable of low‐energy optical transitions and one‐electron reduction chemistry.

## Author Contributions

**Chaopeng Hu**: investigation, methodology, writing – review and editing. **Shenglai Yao**: investigation, methodology, data curation, writing – review and editing. **Christian Lorent**: investigation, writing – review and editing, methodology. **Matthias Driess**: conceptualization, funding acquisition, writing – original draft, supervision.

## Conflicts of Interest

The authors declare no conflicts of interest.

## Supporting information



The authors have cited additional references within the Supporting Information [89–127].
**Supporting File 1**: anie73460‐sup‐0001‐SuppMat.pdf.

**Supporting File 2**: anie73460‐sup‐0002‐cif.zip.

## Data Availability

The data that supports the findings of this study are available in the supplementary material of this article.
