# Supplementary material for: Synthesis and One‐Electron Reduction of Donor‐Acceptor‐Stabilized SiII–EII–SiII Trimetallylenes (E = Ge, Sn, Pb)
Source: Angew Chem Int Ed Engl. 2026 Jul 3;65(37):e6871080. doi: 10.1002/anie.6871080 (PMC13549058; doi:10.1002/anie.6871080)
Supplement: Supplementary file 1 [file ANIE-65-e6871080-s002.pdf]

# Synthesis and One-Electron Reduction of Donor-Acceptor-Stabilized $Si^{II}$-$E^{II}$-$Si^{II}$ Trimetallylenes (E = Ge, Sn, Pb)

Chaopeng Hu, Shenglai Yao, Christian Lorent, and Matthias Driess*


A. Experimental Section ........ 1

A1 General Considerations ........ 1

A2 Single-Crystal X-ray Structure Determination ........ 2

A3. EPR Measurement ........ 3

A4 Synthesis and Characterization ........ 4

A5 Details of the Single Crystal X-ray Diffraction Analyses ........ 29

A6 UV-vis-NIR Spectra ........ 37

A7 Electrochemistry ........ 39

A8 Electron Paramagnetic Resonance (EPR) Spectroscopy ........ 41

A8.1 EPR Spectrum of $\mathbf{2}^{\bullet-}$[K([2.2.2]-cryptand)] ........ 41

A8.2 EPR Spectra of $\mathbf{3}^{\bullet-}$[K([2.2.2]-cryptand)] ........ 42

B. Theoretical Calculations ........ 45

B1 Computational Methods ........ 46

B1.1 General Method ........ 46

B1.2 Method for Determination of Percent Buried Volume ........ 46

B1.3 Method for Excited-State Studies ........ 47

B2 Calculated results ........ 48

B2.1 The Silanylidene Anion ........ 48

B2.2 Percent Buried Volume Data ........ 49

B2.3 Excited State Analysis ........ 52

B2.4 FMO, NBO, and IBO analyses ........ 55

B2.5 Natural Resonance Theory (NRT) of simplified model of **2** ........ 60

B2.6 Energy Decomposition Analysis (EDA) of **2** ........ 61

B2.7 Spin-density and SOMO of Ge(I) and Sn(I) radical anions, $\mathbf{2}^{\bullet-}$ and $\mathbf{3}^{\bullet-}$ ........ 62

B3 Optimized x,y,z-coordinates ........ 63

C. References ........ 113

# *A. Experimental Section*

## A1 General Considerations

All experiments were carried out under dry oxygen-free nitrogen using standard Schlenk techniques or an Ar-system MBraun glove box fitted with a gas purification and recirculation unit. Solvents were dried by standard methods and freshly distilled and were degassed by three freeze-pump-thaw cycles prior to use. $MesSiCl_3$,[1] cAAC[2], $Pb(N(SiMe_3)_2)_2$[3] and bis(silylenes) XT(*L′*Si:)$_2$ (XT = 9,9-dimethyl-xanthene-4,5-diyl, *L′* = PhC(*t*BuN)$_2$, **A**),[4] $Ph_2Si$(*L′*Si:)$_2$ (**B**),[5,6] CB(*L′*Si:)$_2$ (CB = 1,2-$C_2B_{10}H_{10}$, **C**)[7] and PhN(*L′*Si:)$_2$) (**D**)[8,9] were prepared using the reported methods (Mes represents mesityl: 1,3,5-$Me_3C_6H_2$). Both [2.2.2]-Cryptand and Lappert stannylene were purchased from Sigma-Aldrich. The NMR spectra were recorded with Bruker spectrometers Avance II 400, Avance III 400, and Avance III 500 referenced to residual solvent signals as internal standards. Abbreviations: s = singlet; d = doublet; t = triplet; sept = septet; m = multiplet; br = broad. High-resolution ESI-MS (HRMS) were measured on a Thermo Scientific LTQ orbitrap XL. UV/Vis spectra were recorded on an Analytik Jena Specord S600 diode array spectrometer. IR spectra were measured with a Nicolet iS5 FT-IR-Spectrometer from the company of Thermo Scientific. Melting points were measured on a Stuart SMP30 melting point apparatus.

## A2 Single-Crystal X-ray Structure Determination

The crystals were mounted on a glass capillary in polyisobutylene oil and measured in a cold $N_2$ flow. The data of compounds **1a**, **1b**, **2**, **3**, **4**, **2**$^{\bullet-}$[K([2.2.2]-cryptand)] and **3**$^{\bullet-}$[K([2.2.2]-cryptand)] were collected on an Oxford Diffraction Supernova, Single source at offset, Atlas at 150 K (Cu-Kα radiation, λ = 1.5418 Å). The structures were solved with the SHELXT[10] and refined with Olex2[11] software package. The positions of the H atoms were calculated and considered isotropically according to a riding model. CCDC 2503070 (compound **1a**), 2503071 (compound **1b**), 2503073 (compound **2**), 2503074 (compound **3**) and 2503075 (compound **4**), 2535034 (compound **2**$^{\bullet-}$[K([2.2.2]-cryptand)]), 2535035 (compound **3**$^{\bullet-}$[K([2.2.2]-cryptand)]) contain the supplementary crystallographic data for this paper. These data can be obtained free of charge by contacting The Cambridge Crystallographic Data Centre, 12, Union Road, Cambridge CB2 1EZ, UK; fax: +44 1223 336033.

### A3. EPR Measurement

The EPR spectra of **2**$^{\bullet-}$[K([2.2.2]-cryptand)] in THF (3 mM) were recorded at 293 K using a Bruker EMXplus spectrometer with an ER 4122 SHQE resonator. Spectra of the frozen solution from **3**$^{\bullet-}$[K([2.2.2]-cryptand)] in THF (3 mM) measured at 10 K were obtained using a custom-built X-band spectrometer equipped with a Bruker SHQ resonator, an ESR 910 helium flow cryostat, and an ITC503 temperature controller. Experiments were performed under the following conditions: microwave power of 1 mW, frequency between 9.3 and 9.4 GHz, a modulation amplitude of 0.5 G respectively 2 G for **2**$^{\bullet-}$[K([2.2.2]-cryptand)] and **3**$^{\bullet-}$[K([2.2.2]-cryptand)], and modulation frequency of 100 kHz. Spectral simulations were performed using the EasySpin MATLAB toolbox (version 6.0.12), employing the "garlic" fitting function for isotropic radicals in solution and "pepper" fitting function for solid state continuous wave EPR spectra.

## A4 Synthesis and Characterization

Synthesis of **1a**

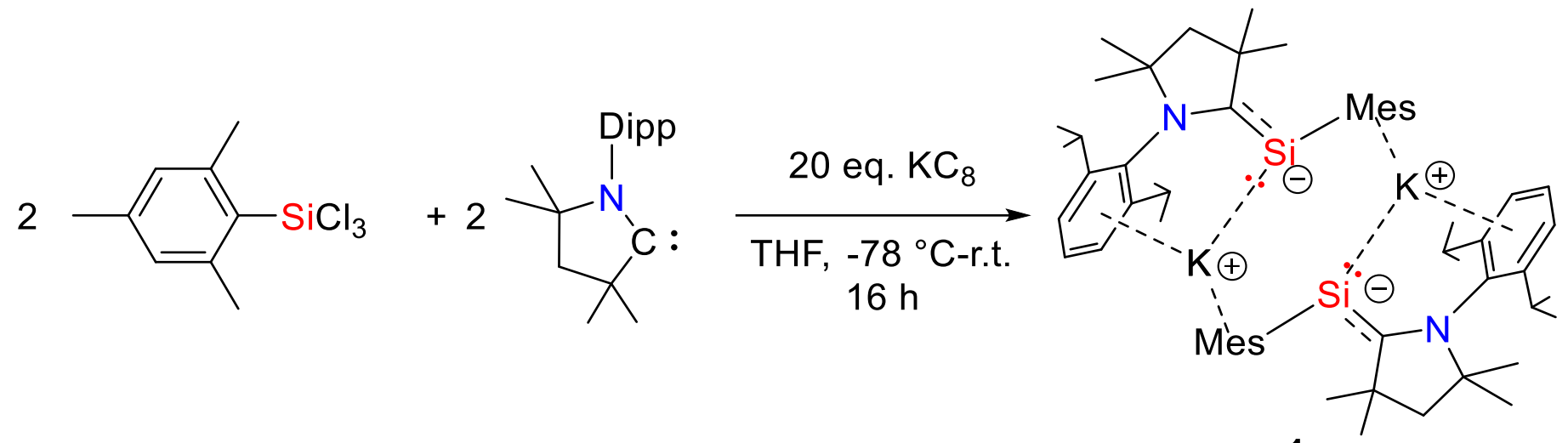


**Scheme S1**. Synthesis of dimeric potassium silanylidene salt $(Si^{II}K)_2$ **1a**.

A cold THF (200 mL) was slowly added to a mixture of $MesSiCl_3$ (5.3 g, 20.9 mmol), cAAC (5.97 g, 20.9 mmol), and $KC_8$ (28.3 g, 209.3 mmol) at -78 °C. The resulting solution was gradually warmed to room temperature and stirred for 16 hours, during which the starting materials were completely consumed, yielding a dark red solution. All volatiles from this solution were then removed under vacuum. The remaining mixture was extracted with a mixture of hexane and $Et_2O$, and subsequently filtered, resulting in a red solution. Concentrating the extract yielded **1a** as a red solid (4.5 g, 4.8 mmol, 46% yield). Single crystals of **1a** suitable for X-ray diffraction were obtained through the gradual evaporation of a concentrated $Et_2O$ solution at -30 °C. **M.P.** 247 °C (decomp.).

**$^1$H NMR** (400 MHz, $C_6D_6$, 298K): δ (ppm) 6.94 – 6.88 (m, 2H), 6.83 (d, *J* = 7.2 Hz, 4H), 6.78 (s, 4H), 3.66 – 3.48 (m, 4H), 2.68 (s, 12H), 2.23 (s, 6H), 2.01 (s, 4H), 1.56 (s, 12H), 1.47 (d, *J* = 6.7 Hz, 12H), 1.23 (d, *J* = 6.8 Hz, 12H), 1.21 (s, 12H).

**$^{13}$C{$^1$H} NMR** (101 MHz, $C_6D_6$, 298K): δ (ppm) 194.2 (*C* in cAAC), 156.7 (*C*), 154.2 (*C*), 143.8 (*C*), 143.4 (*C*), 134.0 (*C*), 128.3 (*C*H), 127.7 (*C*H), 128.3 (*C*H), 126.0 (*C*H), 124.4 (*C*H), 63.7, 60.0 ($CH_2$ in the five-member ring of cAAC), 48.5, 33.8, 29.3, 28.2, 27.7, 26.7, 24.9, 21.3.

**$^{29}$Si{$^1$H} NMR** (79 MHz, $C_6D_6$, 298K): δ (ppm) 42.2.

**$^1$H NMR** (400 MHz, THF-$d_8$): δ (ppm) 7.22 – 7.12 (m, 2H), 7.13 – 7.06 (m, 4H), 6.51 (s, 4H), 3.66 – 3.59 (m, 4H), 2.47 (s, 12H), 2.09 (s, 6H), 1.86 (s, 4H), 1.60 (d, *J* = 6.7 Hz, 12H), 1.22 (d, *J* = 6.8 Hz, 12H), 1.18 (d, *J* = 5.1 Hz, 24H).

**$^{13}$C{$^1$H} NMR** (101 MHz, THF-$d_8$): δ (ppm) 191.5 (*C* in cAAC), 156.9 (*C*), 154.4 (*C*), 144.9 (*C*), 143.4 (*C*), 131.9 (*C*), 126.8 (*C*), 126.7 (*C*H), 125.8 (*C*H), 124.8 (*C*H), 63.1, 61.8($CH_2$ in the five-member ring of cAAC), 48.6, 34.1, 29.8, 28.9, 28.3, 27.1, 26.5, 21.6.

**$^{29}$Si{$^1$H} NMR** (79 MHz, THF-$d_8$): δ (ppm) 49.5.

**IR** ( $cm^{-1}$): 2966.34 (w), 2921.56 (w), 2857.07 (w), 1564.62 (w), 1447.23 (w), 1434.43 (w), 1375.19 (w), 1356.83 (w), 1322.58 (m), 1279.13 (w), 1244.52 (w), 1216.43 (w), 1189.79 (m), 1158.02 (s), 1106.89 (w), 1060.89 (w), 1047.73 (w), 1031.06 (w), 1006.68 (w), 942.38 (w), 931.60 (w), 900.17 (w), 852.00 (m), 810.80 (m), 780.86 (m), 741.80 (w), 725.97 (w), 695.44 (w), 611.55 (w), 599.35 (w), 589.98 (w), 580.84 (w), 565.17 (w).

**Note:** Due to the sensitivity of **1a** to the ionization source during measurement, the compound was found to decompose under mass spectrometric conditions, preventing reliable mass spectrometric characterization.

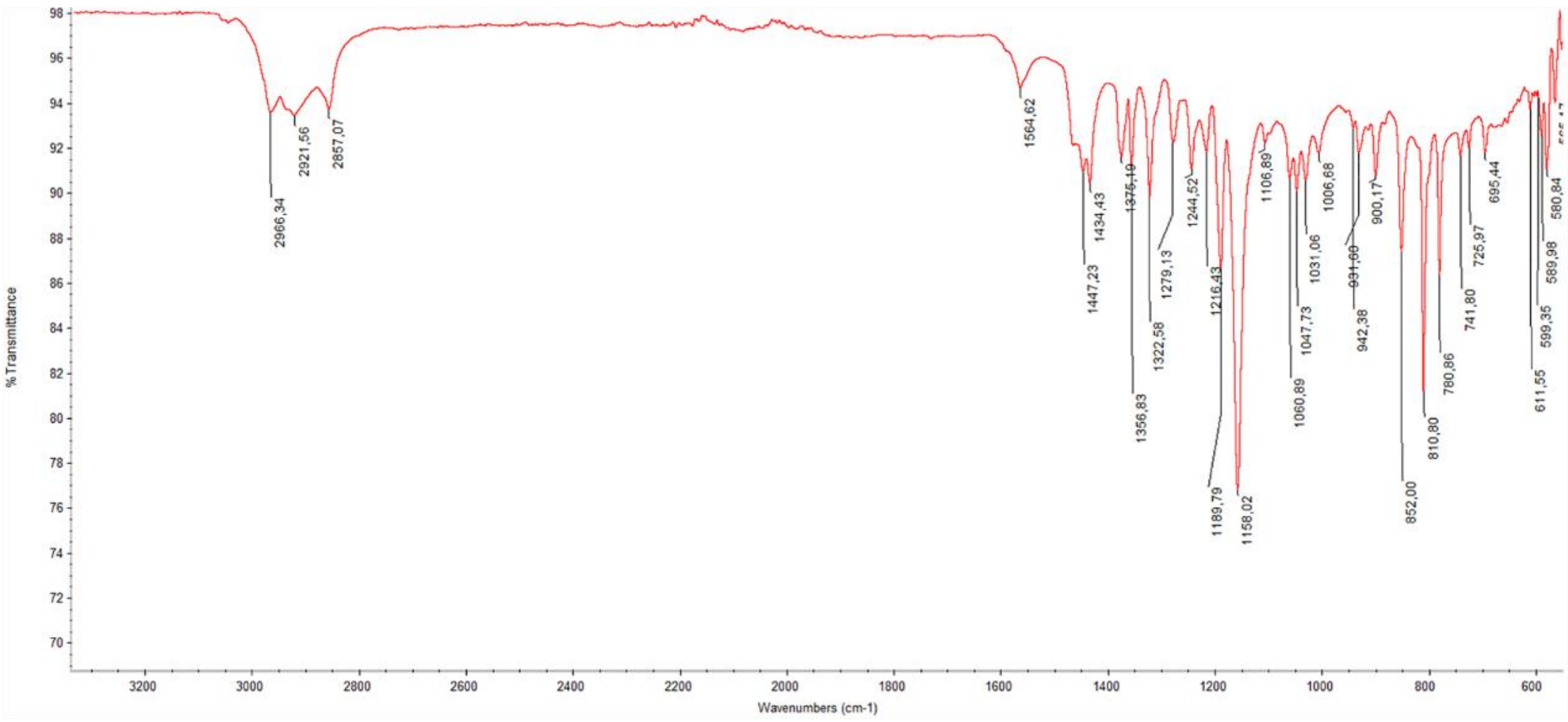


**Figure S1.** IR spectrum of **1a**.

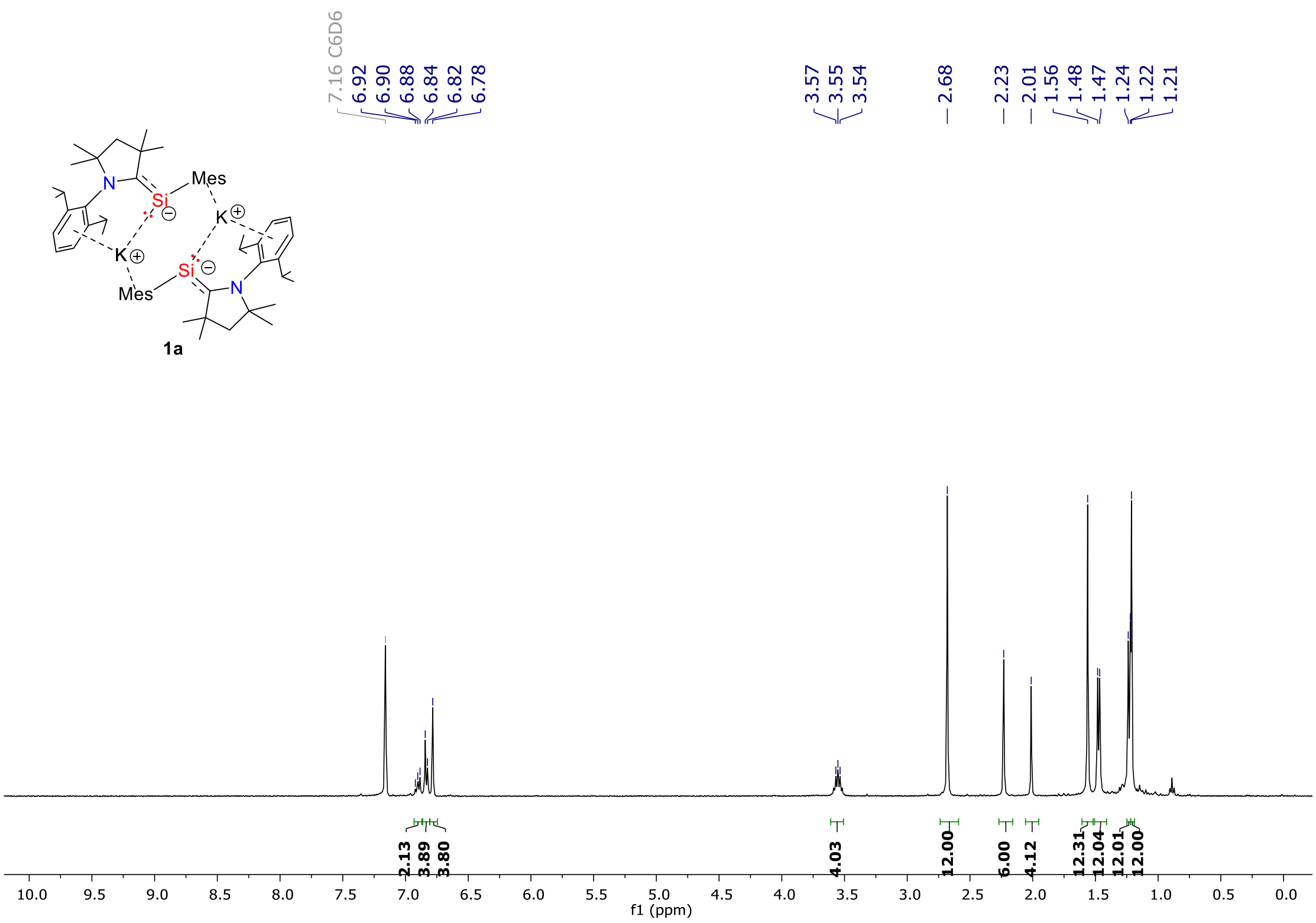


**Figure S2**. $^{1}$H NMR (400 MHz, $C_6D_6$, 298K) spectrum of **1a**.

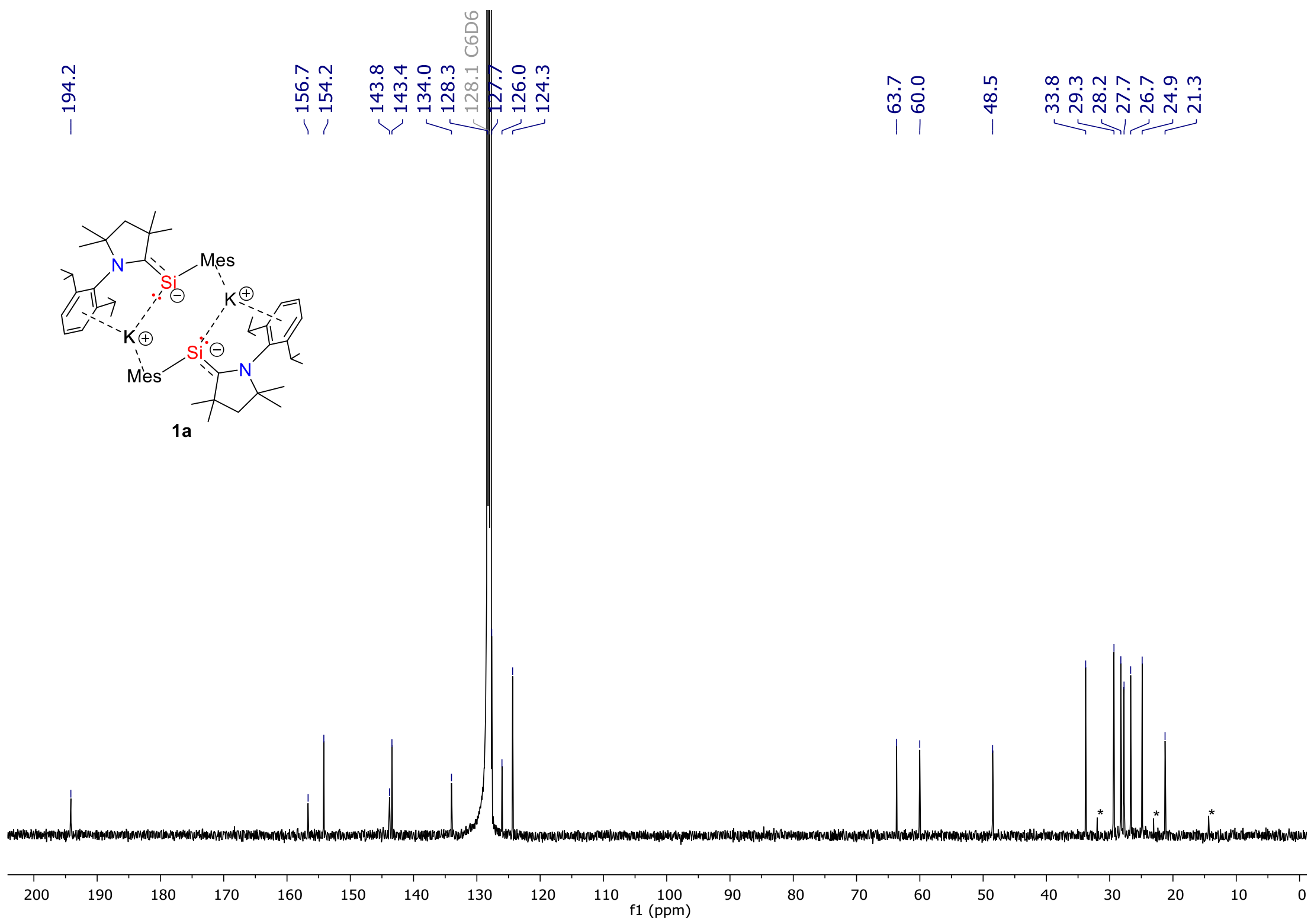


**Figure S3**. $^{13}C\{^{1}H\}$ NMR spectrum of **1a** (101 MHz, $C_6D_6$, 298K). *marks the residue of n-hexane.

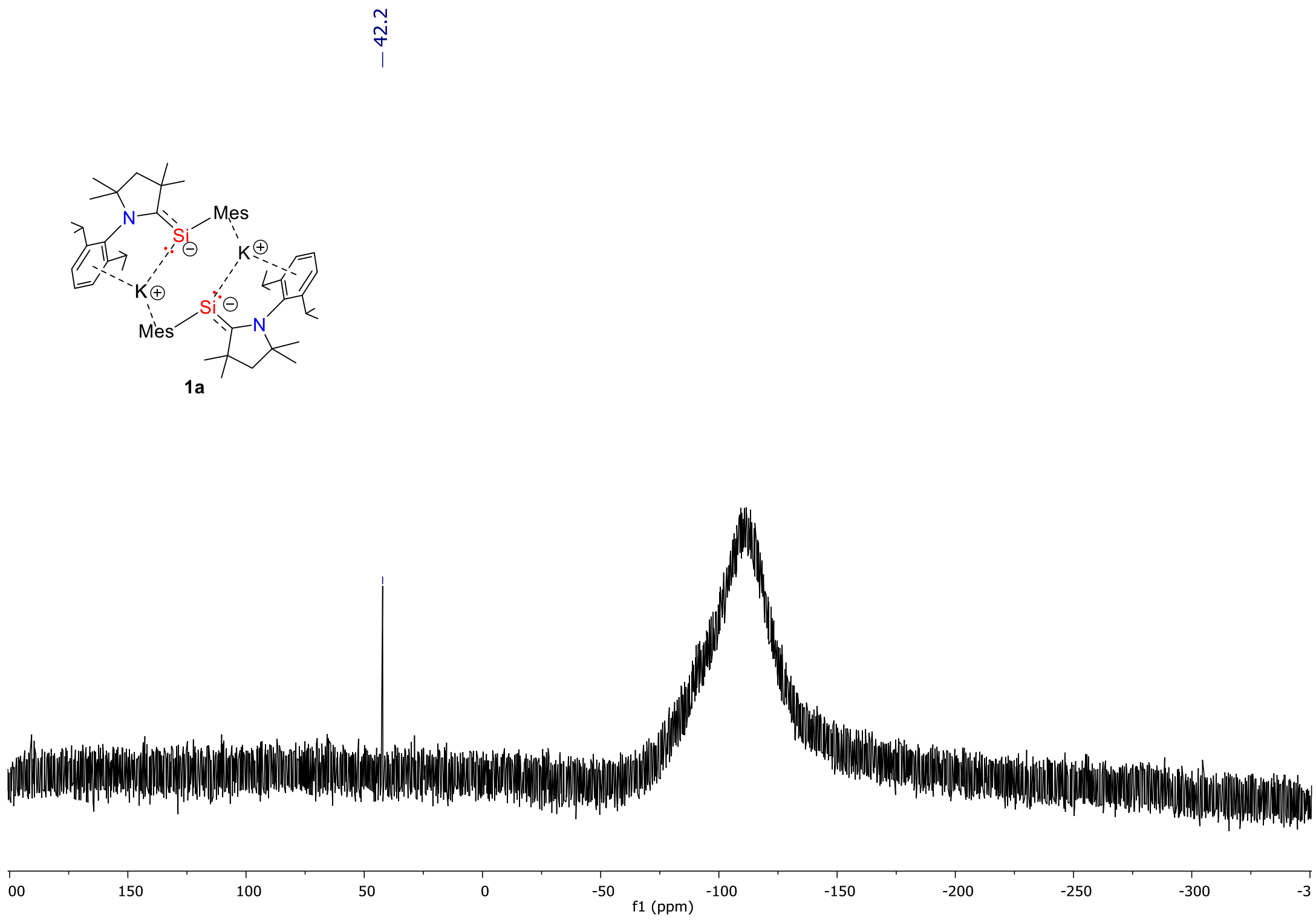


**Figure S4**. $^{29}Si\{^{1}H\}$ NMR spectrum of **1a** (79 MHz, $C_6D_6$, 298K).

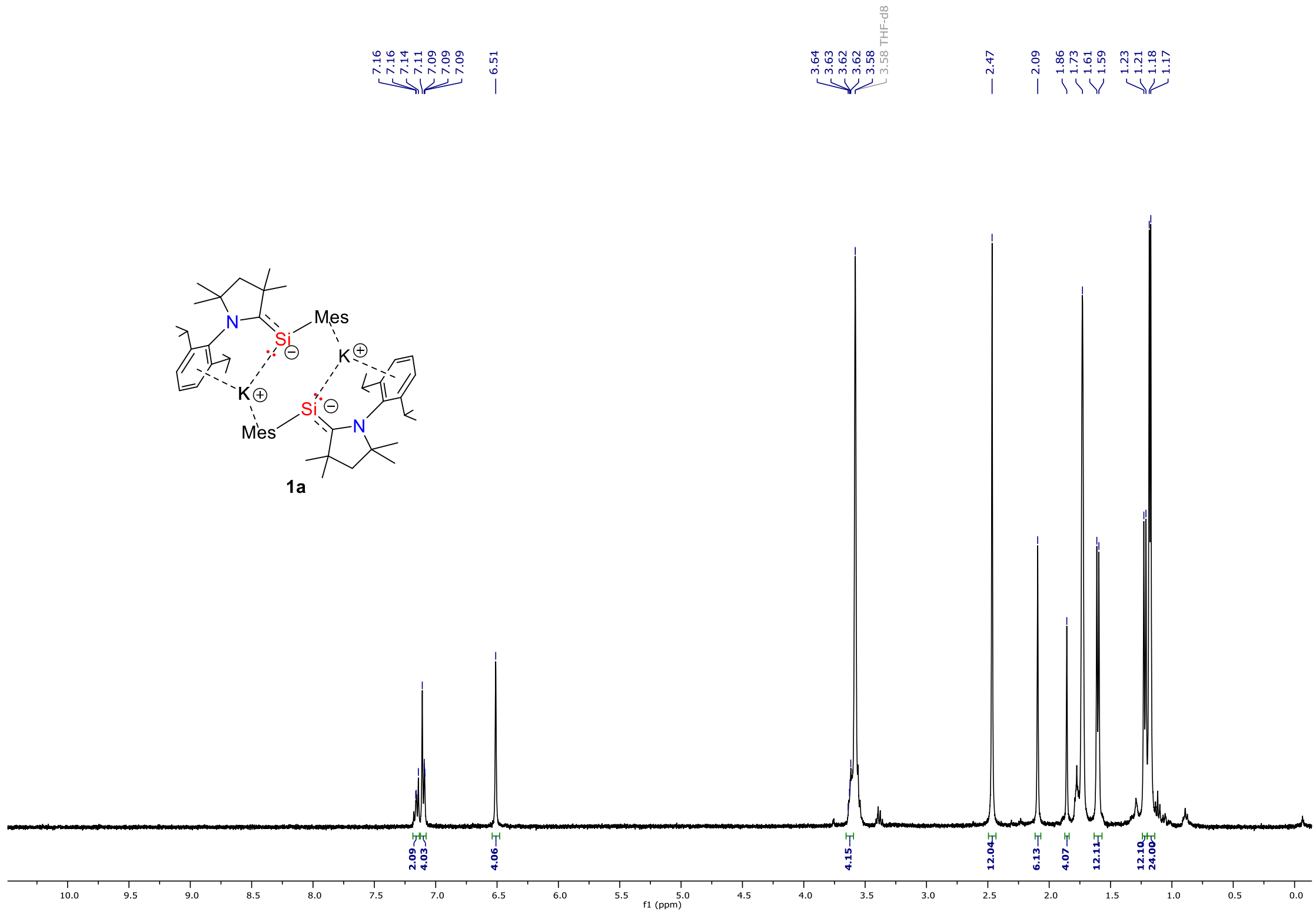

**Figure S5**. $^{1}$H NMR (400 MHz, THF-$d_8$, 298K) spectrum of **1a**.

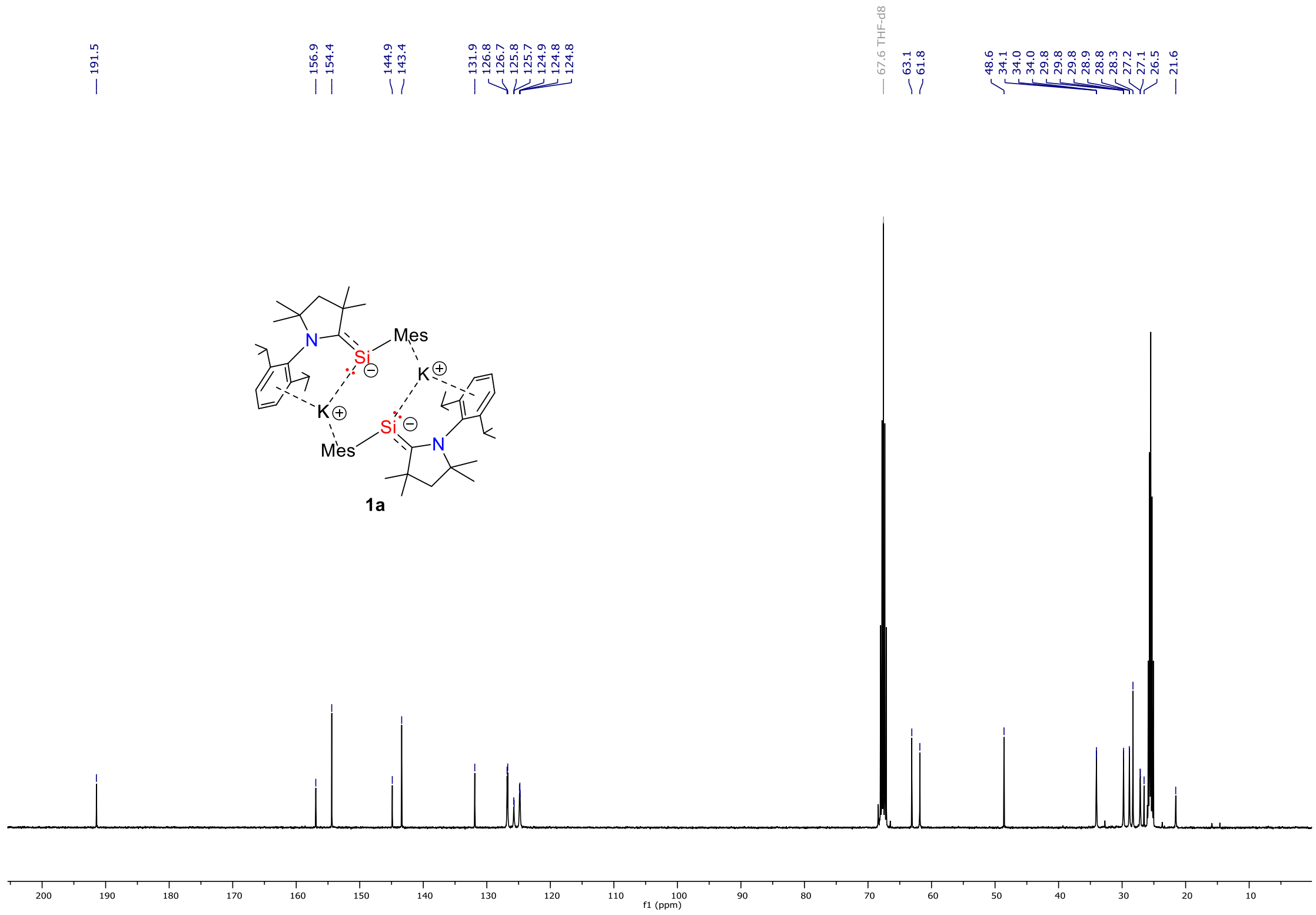

**Figure S6**. $^{13}$C{$^{1}$H} NMR spectrum of **1a** (101 MHz, THF-$d_8$, 298K).

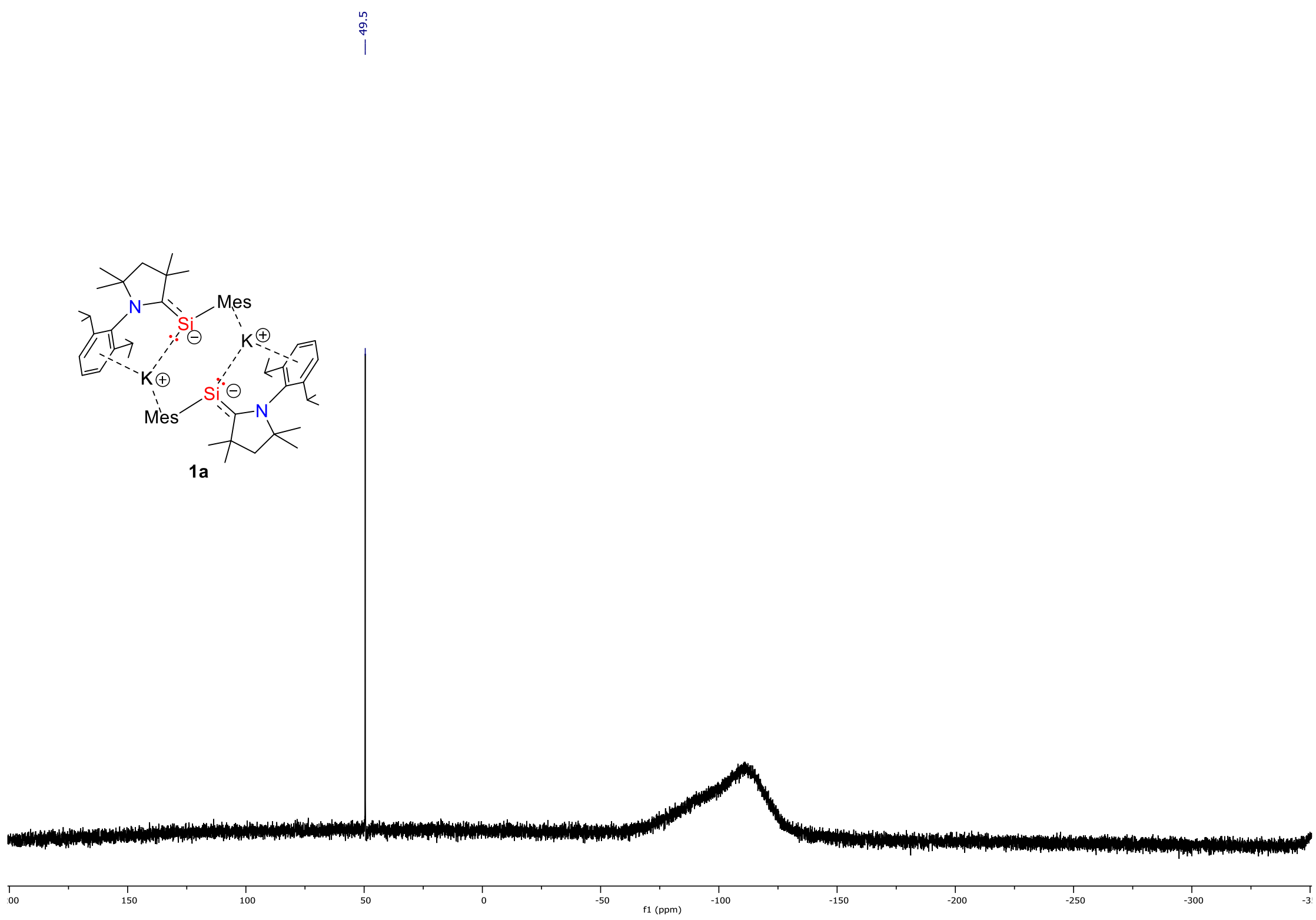

**Figure S7**. $^{29}Si\{^{1}H\}$ NMR spectrum of **1a** (79 MHz, THF-$d_8$, 298K).

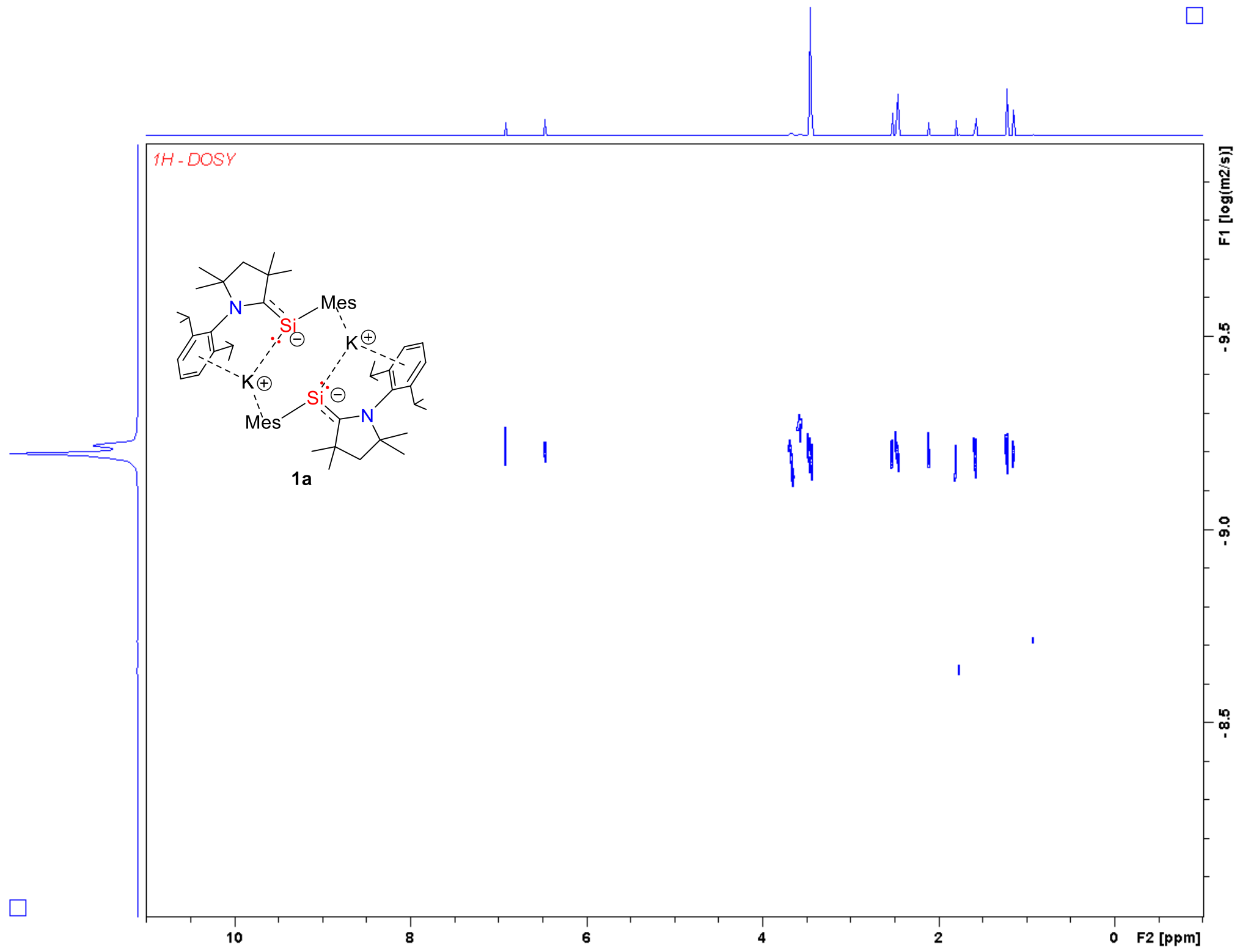

**Figure S8.** $^{1}H$ DOSY NMR spectrum of **1a** (500 MHz, THF-$d_8$, 298K, 0.015 M), showing a diffusion coefficient of $7 \times 10^{-10}$ m² s⁻¹.

Synthesis of **1b**

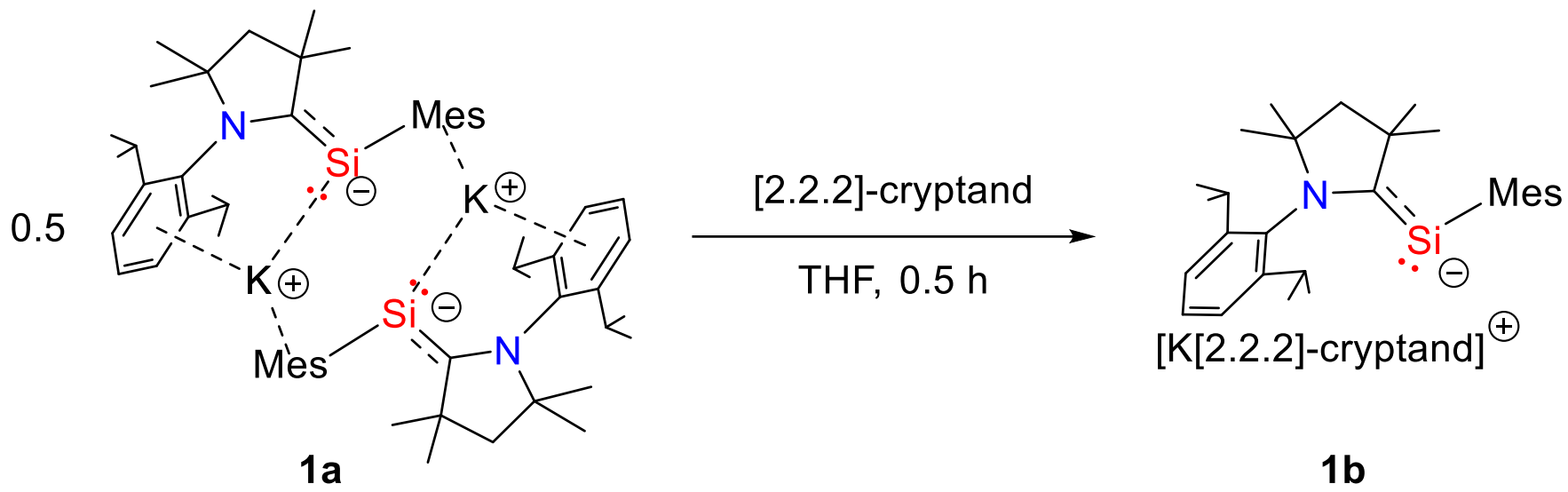


**Scheme S2**. Synthesis of potassium silanylidene salt [K([2.2.2]-cryptand)][$Si^{II}$] **1b**.

A solution of [2.2.2]-cryptand (100 mg, 0.27 mmol) in THF (10.0 mL) was slowly added to a separate THF solution (10 mL) containing **1a** (79.8 mg, 0.2 mmol) at room temperature. After stirring for 30 minutes, the starting material was completely consumed, resulting in a red solution. The volatiles in this solution were then removed under vacuum, yielding a deep red solid. The mixture was washed with pentane. After removing the remaining volatiles, **1b** was obtained as a red solid (144.3 mg, 0.17 mmol, 85% yield). Single crystals of **1b** suitable for X-ray diffraction analysis were obtained by gradually evaporating a concentrated diethyl ether solution at -30°C. **M.P.** 87 °C (decomp.).

**$^{1}$H NMR** (400 MHz, THF-$d_8$, 298K): δ (ppm) 6.88 (s, 3H), 6.43 (s, 2H), 3.68 – 3.60 (m, 2H), 3.47 – 3.34 (m, 24H, OC$H_2$ in [2.2.2]-cryptand), 2.49 (s, 6H), 2.43 (s, 12H, NC$H_2$ in [2.2.2]-cryptand), 2.07 (s, 3H), 1.76 (s, 2H), 1.54 (d, $J$ = 6.7 Hz, 6H), 1.19 – 1.16 (m, 12H), 1.11 (s, 6H).

**$^{13}$C{$^{1}$H} NMR** (101 MHz, THF-$d_8$, 298K): δ (ppm) 185.7(*C* in cAAC), 163.1(*C*), 152.8(*C*), 143.9(*C*), 143.4(*C*), 129.9 (*C*), 126.3(*C*H), 124.9(*C*H), 123.9(*C*H), 71.5, 68.9, 62.6, 62.2, 55.5, 48.4, 34.5, 29.2, 28.8, 28.7, 27.3, 25.7, 21.7.

**$^{29}$Si{$^{1}$H} NMR** (79 MHz, THF-$d_8$, 298K): δ (ppm) 60.5.

**IR** ($cm^{-1}$): 2953.54 (w), 2870.36 (w), 2811.66 (w), 1590.79 (w), 1477.33 (w), 1443.29 (m), 1360.47 (m), 1353.88 (m), 1297.59 (m), 1257.60 (m), 1221.80 (w), 1186.05 (w), 1153.02 (m), 1131.14 (m), 1101.24 (s), 1078.37 (s), 983.08 (w), 948.42 (m), 931.00 (m), 845.24 (m), 830.94 (m), 818.80 (m), 806.45 (m), 771.87 (m), 751.57 (m), 720.56 (m), 692.74 (w), 610.11 (w), 590.55 (w), 568.03 (w).

**Note:** Due to the sensitivity of **1b** to the ionization source during measurement, the compound was found to decompose under mass spectrometric conditions, preventing reliable mass spectrometric characterization.

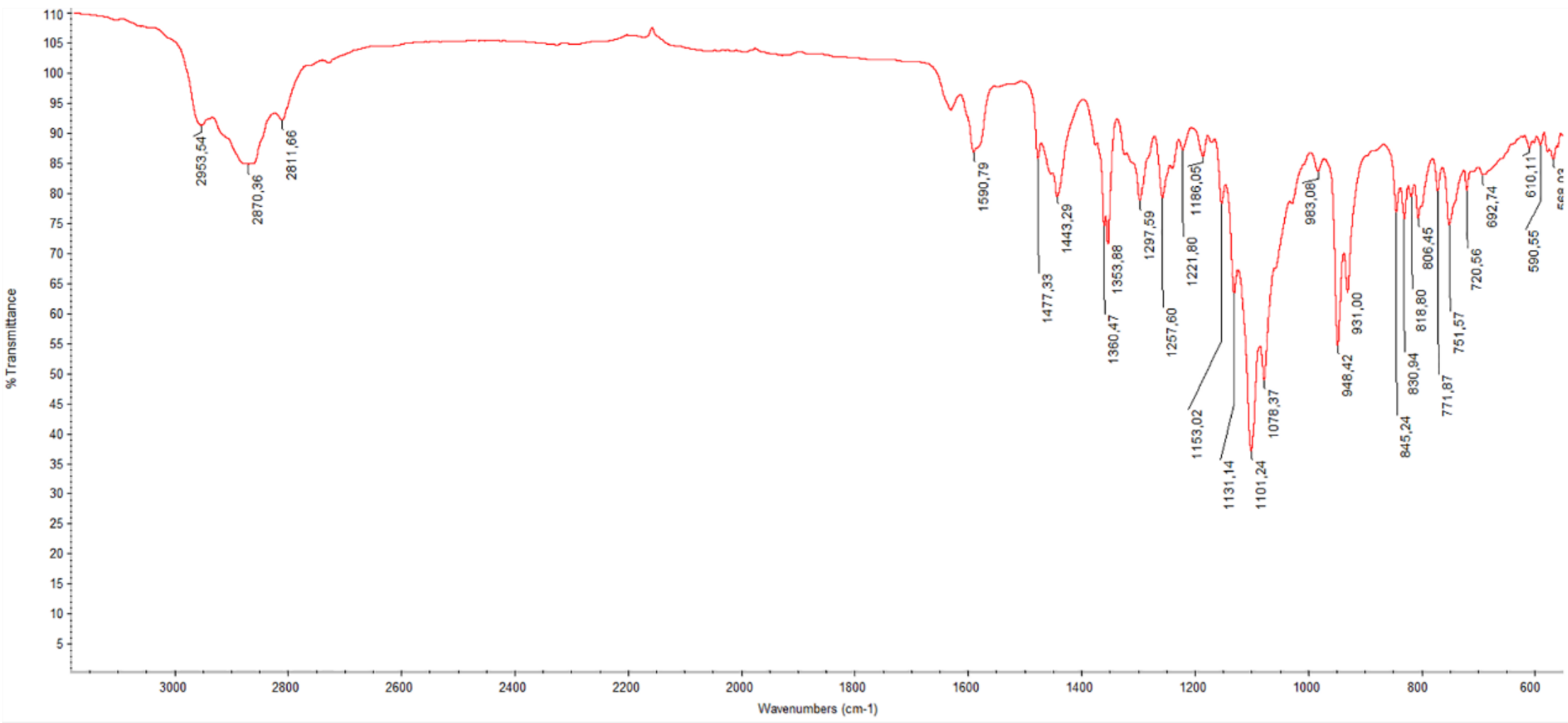

**Figure S9.** IR spectrum of **1b**.

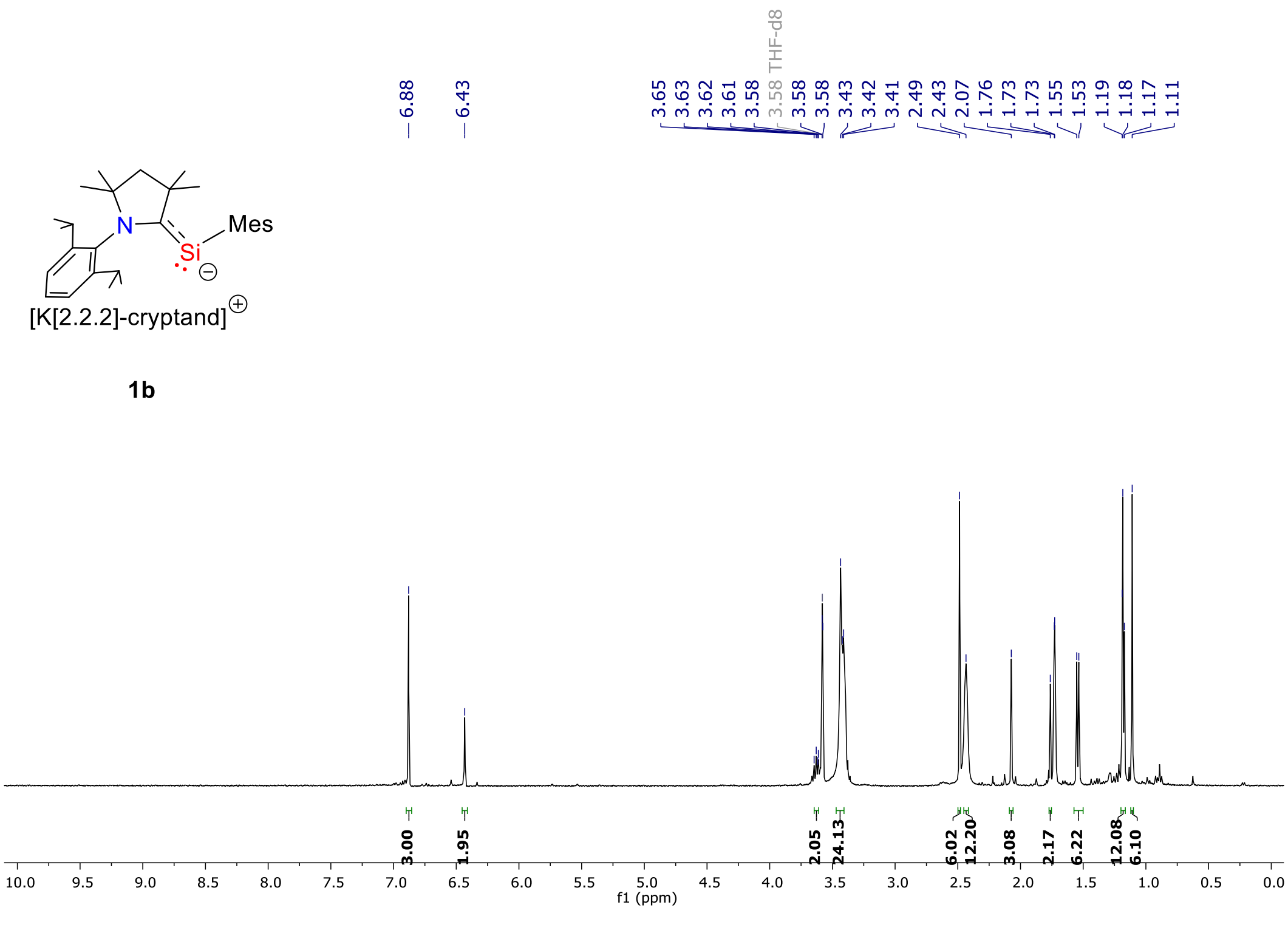


**Figure S10.** $^{1}$H NMR spectrum of **1b** (400 MHz, THF-*d8*, 298K).

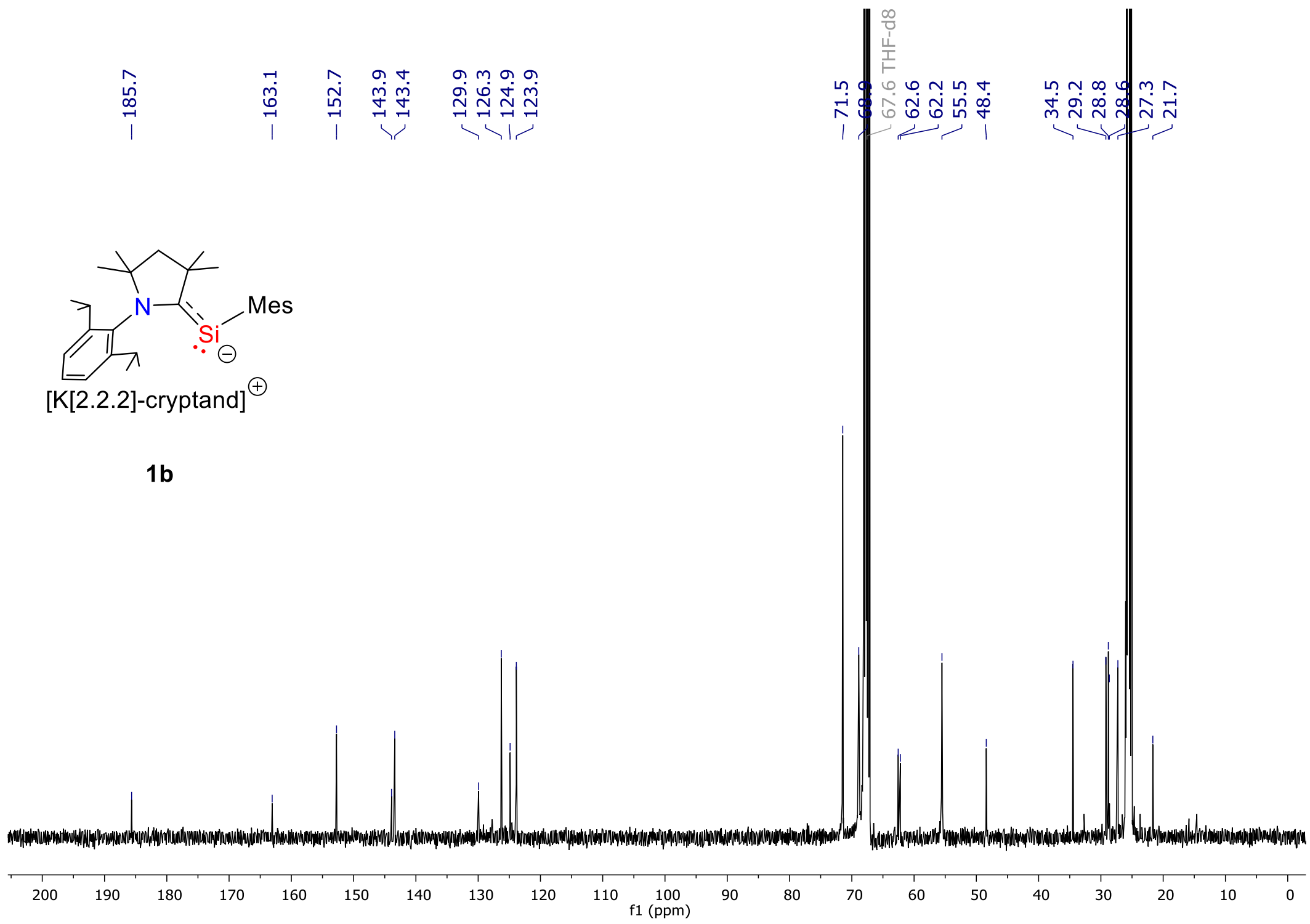


**Figure S11.** $^{13}C\{^{1}H\}$ NMR spectrum of **1b** (101 MHz, THF-*d8*, 298K).

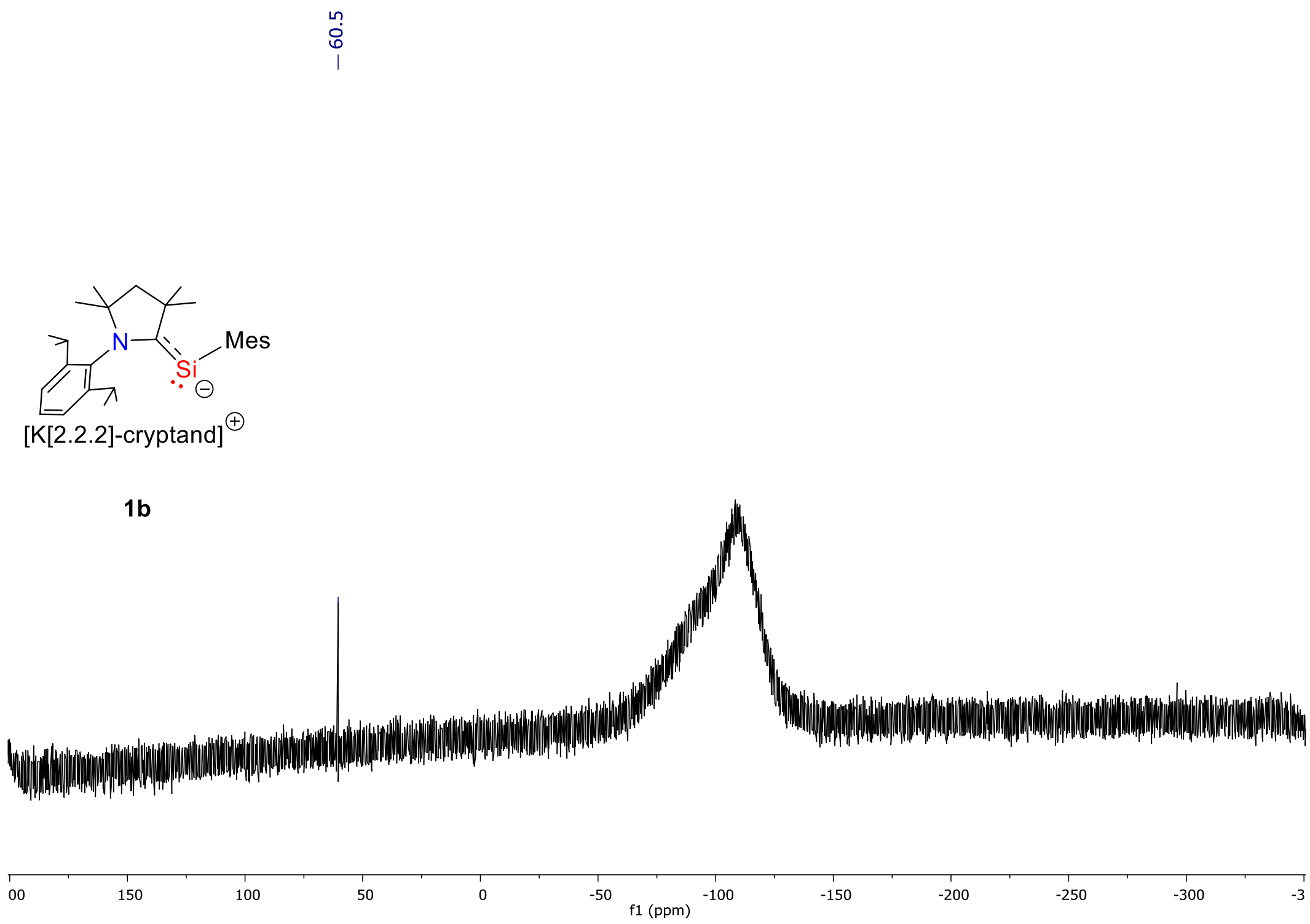


**Figure S12**. $^{29}Si\{^{1}H\}$ NMR spectrum of **1b** (79 MHz, THF-*d8*, 298K).

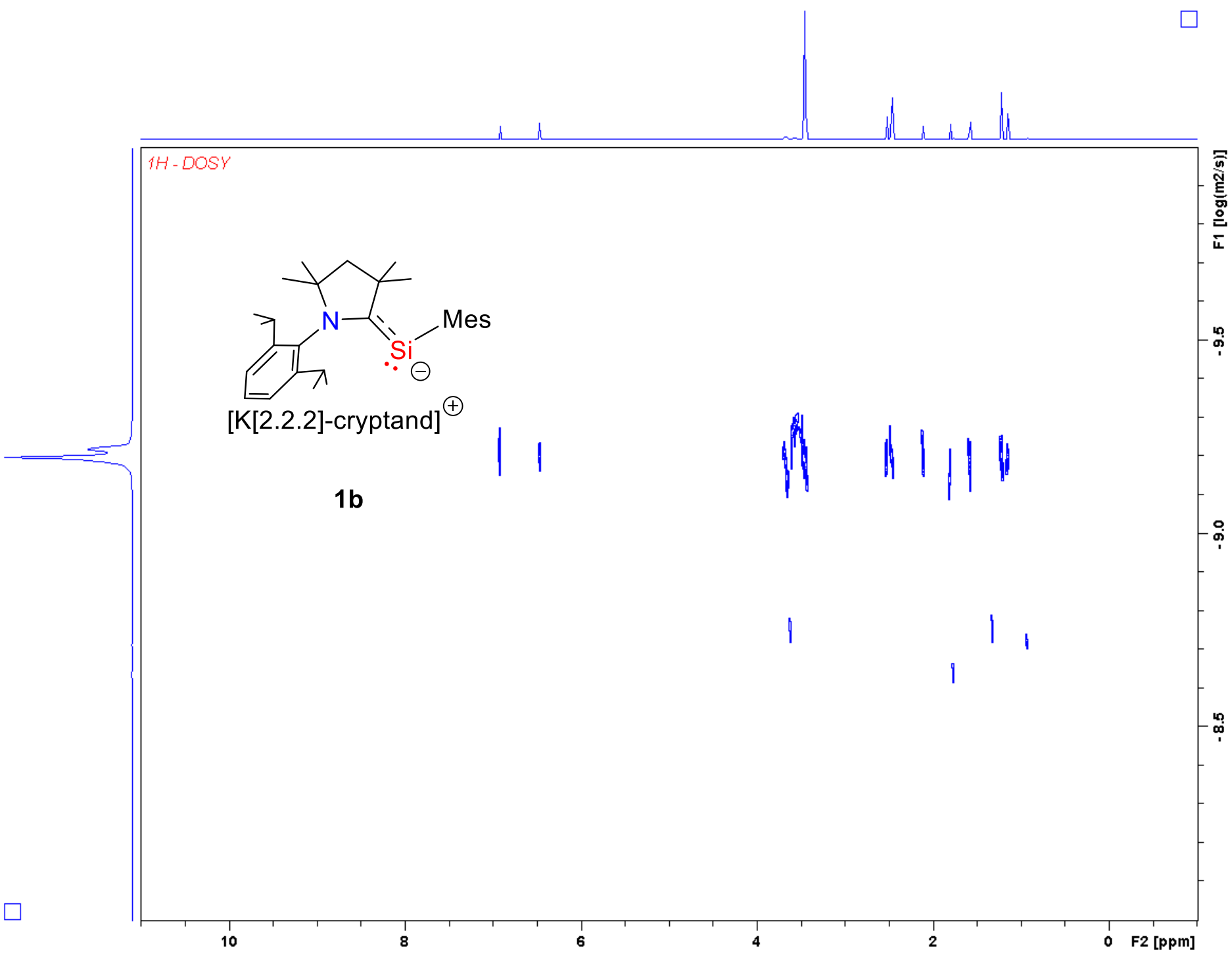


**Figure S13.** ¹H DOSY NMR spectrum of **1b** (500 MHz, THF-$d_8$, 298K, 0.015 M), showing a diffusion coefficient of $6 \times 10^{-10}$ m² s⁻¹.

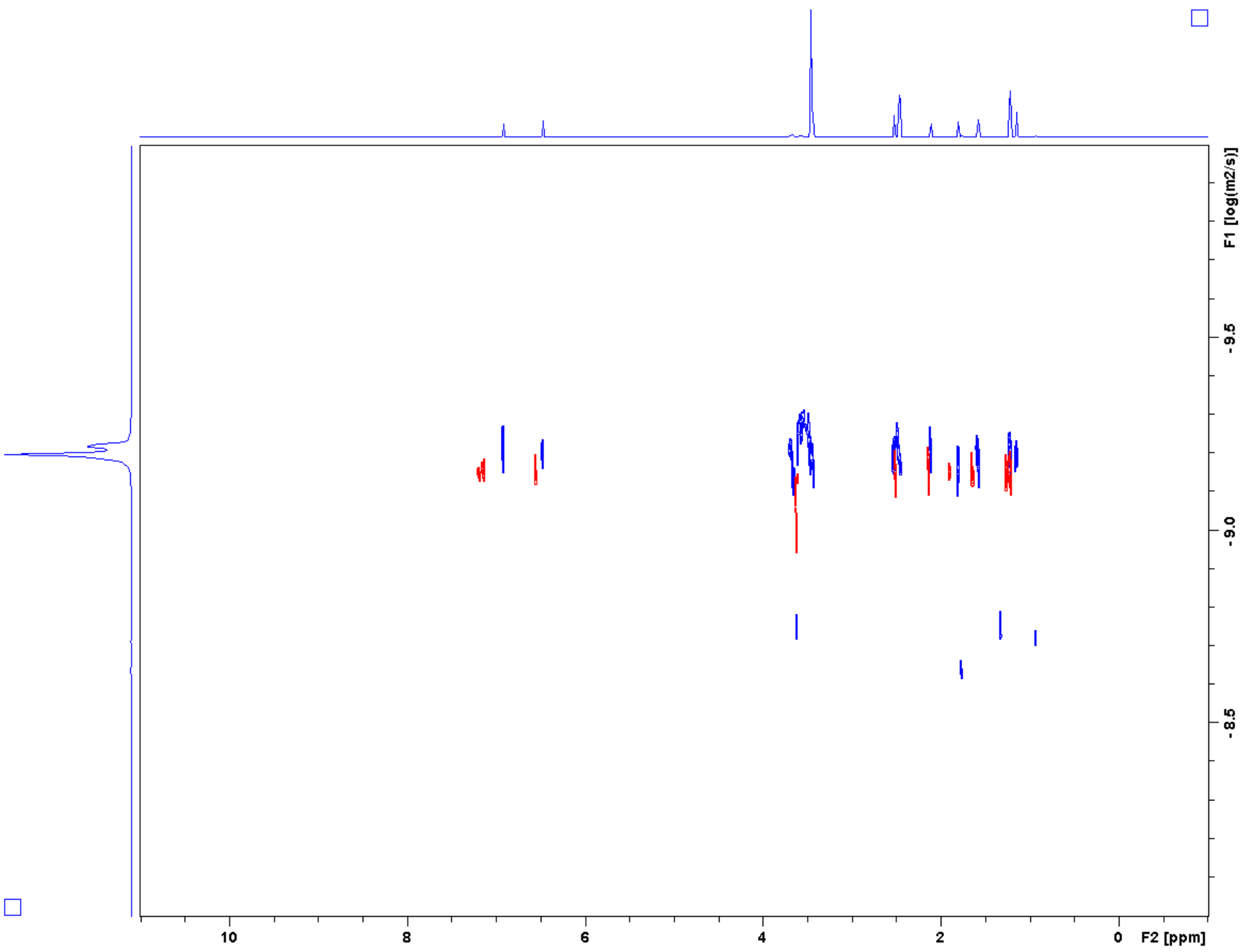


**Figure S14.** Overlay of the ¹H DOSY NMR spectra of **1a** (red) and **1b** (blue) recorded in THF-$d_8$ at 298 K (500 MHz, 0.015 M).

Note: The DOSY data are used only for qualitative comparison of hydrodynamic sizes under identical conditions. They do not by themselves establish a unique aggregation state.

Synthesis of **2**

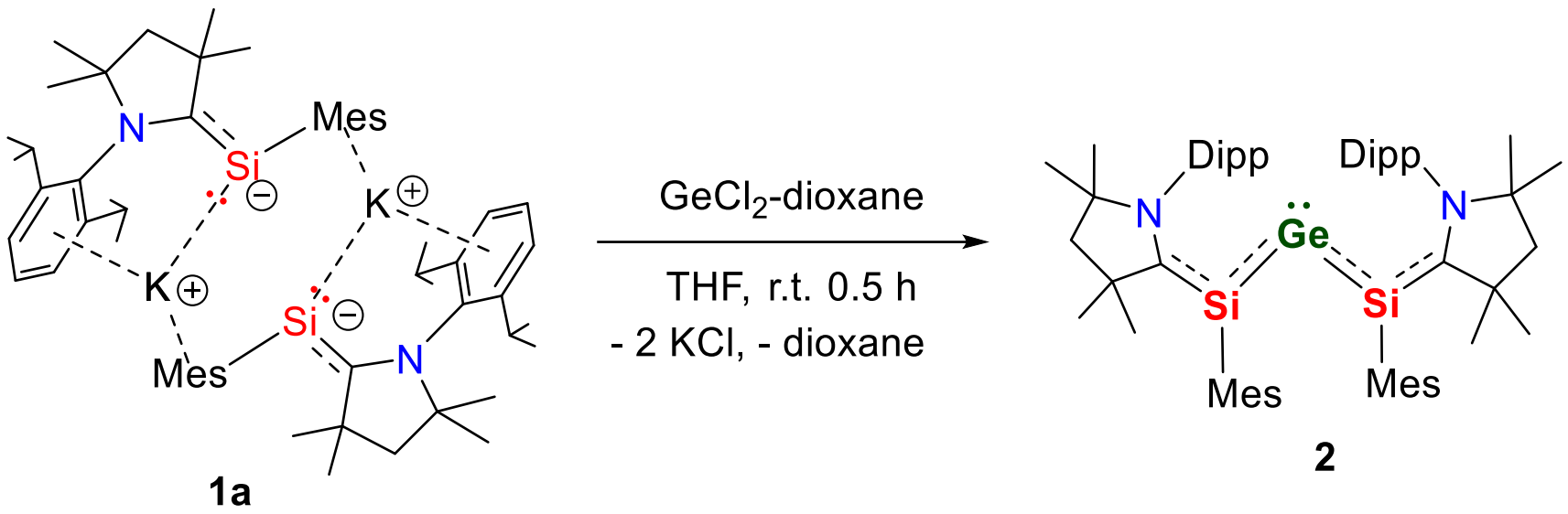


**Scheme S3**. Synthesis of **2**.

A solution of $GeCl_2$-dioxane (9.3 mg, 0.04 mmol in THF (10.0 mL) was gradually added to a THF solution (10.0 mL) containing **1a** (40 mg, 0.04 mmol) at room temperature. After stirring for 30 minutes, the starting material was completely consumed, resulting in a dark green solution. All volatile components, including dioxane, were then removed under vacuum, yielding a deep green solid. The residue was extracted with pentane and filtered, resulting in a green solution. Concentration of the pentane solution afforded **2** as a green solid (37 mg, 0.04 mmol, 91 % yield). Single crystals of **2** suitable for X-ray diffraction were obtained by gradually evaporating a concentrated solution of hexamethyldisiloxane (HMDSO) at -30°C. **M.P.** 85 °C (decomp.).

The same synthetic method produces **2** by reacting **1b** with $GeCl_2$-dioxane.

**$^1$H NMR** (400 MHz, $C_6D_6$, 298K): δ (ppm) 7.35 – 7.26 (m, 2H), 7.19 (s, 4H), 6.58 (s, 4H), 3.16 (p, $J$ = 6.7 Hz, 4H), 2.47 (s, 12H), 2.24 (s, 6H), 1.70 (s, 4H), 1.50 (d, $J$ = 6.7 Hz, 12H), 1.34 (d, $J$ = 6.7 Hz, 12H), 1.19 (s, 12H), 1.11 (s, 12H).

**$^{13}$C{$^1$H} NMR** (101 MHz, $C_6D_6$, 298K): δ (ppm) 190.3(s, *C*), 147.5(s, *C*), 143.5(s, *C*), 142.6(s, *C*), 136.6(s, *C*), 136.2(s, *C*), 129.5(s, *C*H), 128.6(s, *C*H), 128.4(s, *C*H), 127.8(s, *C*H), 127.5(s, *C*H), 68.5(s, *C*), 57.9(*C*$H_2$ in the five-member ring of cAAC), 49.8(s, *C*), 31.4, 29.6, 29.1, 29.0, 27.1, 25.7, 21.6.

**$^{29}$Si{$^1$H} NMR** (79 MHz, $C_6D_6$, 298K): δ (ppm) 91.1.

**HR-MS(ESI)**: (m/z) calcd for $[M]^+$($C_{58}H_{84}N_2Si_2Ge^+$) 938.5379, found 938.5374.

**IR** (cm$^{-1}$): 2961.08 (w), 2917.63 (w), 2861.76 (w), 1600.33 (w), 1453.16 (w), 1437.97 (w), 1381.89 (w), 1364.19 (w), 1293.44 (s), 1250.81 (m), 1223.42 (w), 1186.00 (w), 1162.69 (m), 1127.79 (s), 1081.29 (w), 1049.99 (w), 1039.77 (w), 1008.80 (s), 994.75 (s), 972.55 (m), 929.50 (w), 905.86 (m), 884.63 (w), 842.73 (m), 802.10 (m), 782.83 (m), 734.26 (w), 715.07 (w), 697.79 (w), 649.91 (w), 628.43 (m), 615.75 (w), 606.14 (w), 582.22 (m), 577.89 (m), 571.59 (m).

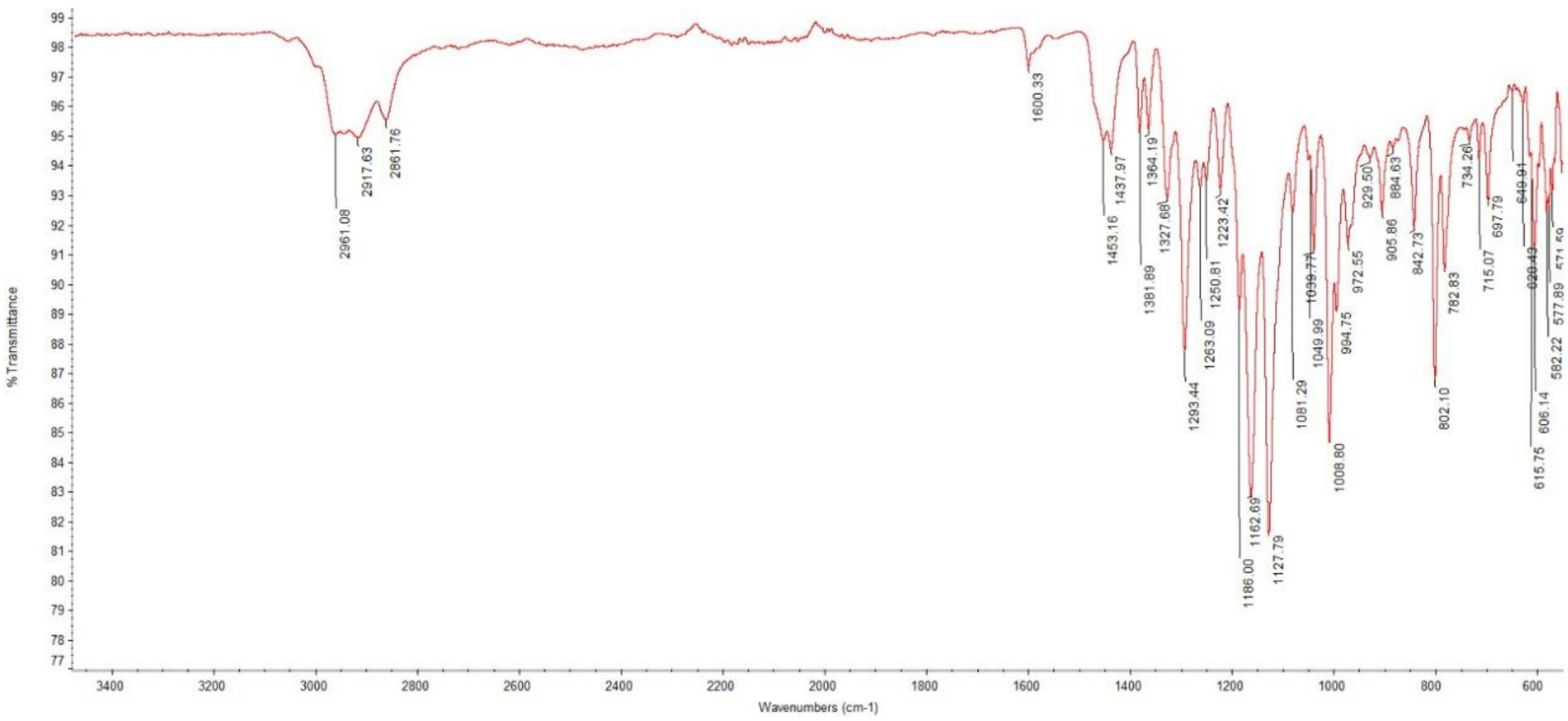

**Figure S15.** IR spectrum of compound **2**.

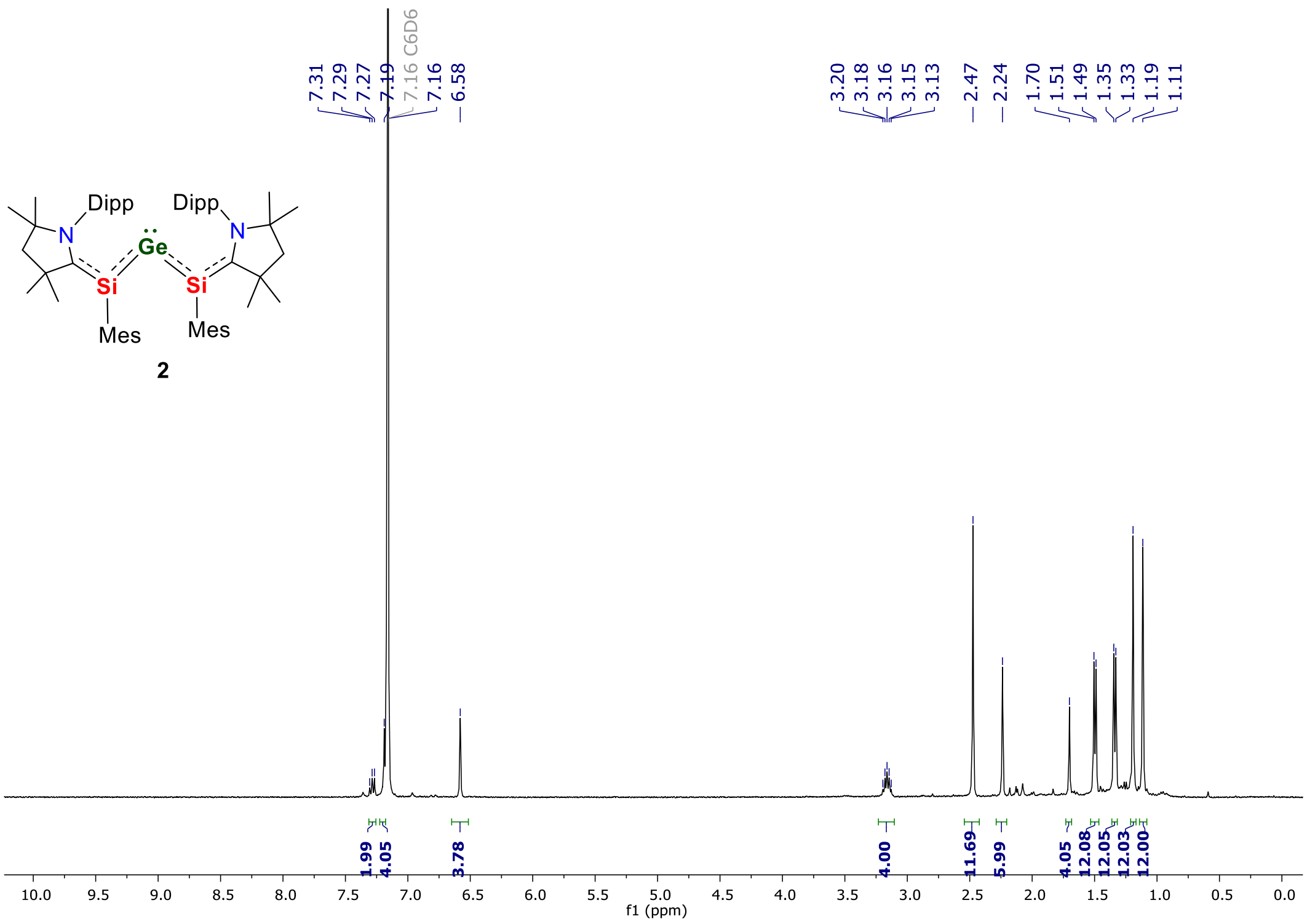

**Figure S16**. $^{1}$H NMR spectrum of **2** (400 MHz, $C_6D_6$, 298K).

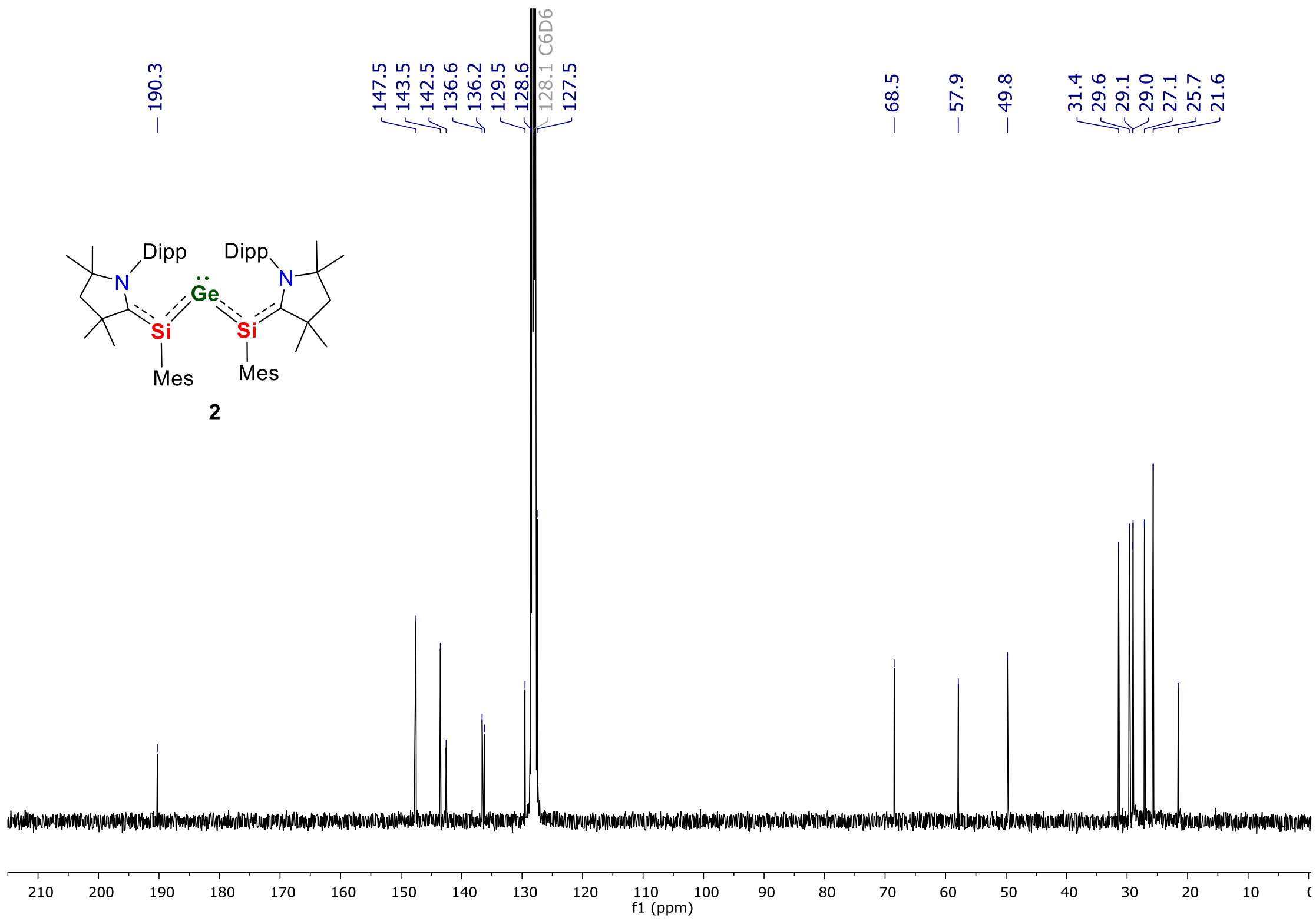


**Figure S17**. $^{13}C\{^{1}H\}$ NMR spectrum of **2** (101 MHz, $C_6D_6$, 298K).

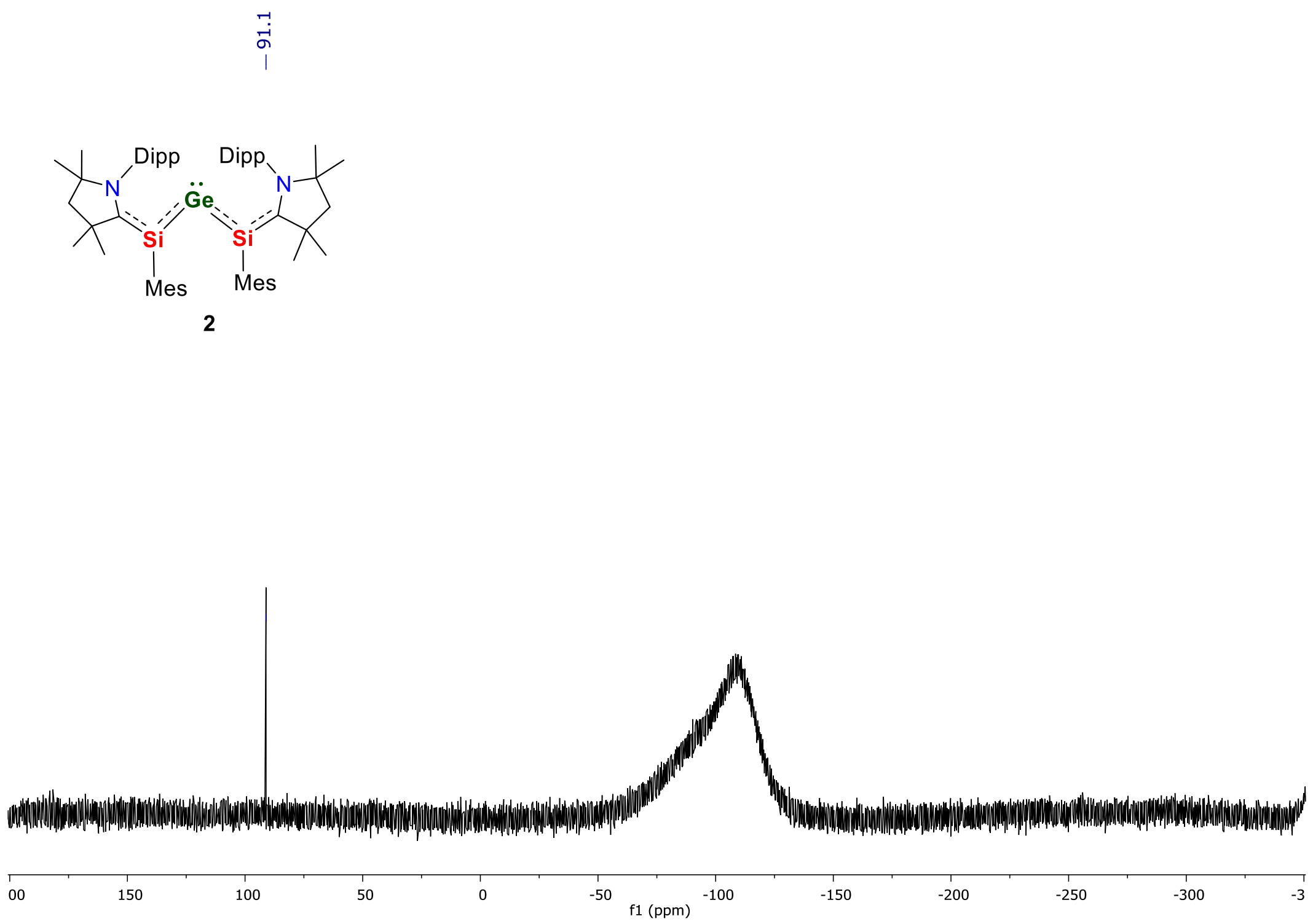


**Figure S18**. $^{29}Si\{^{1}H\}$ NMR spectrum of **2** (79 MHz, $C_6D_6$, 298K).

Synthesis of **3**

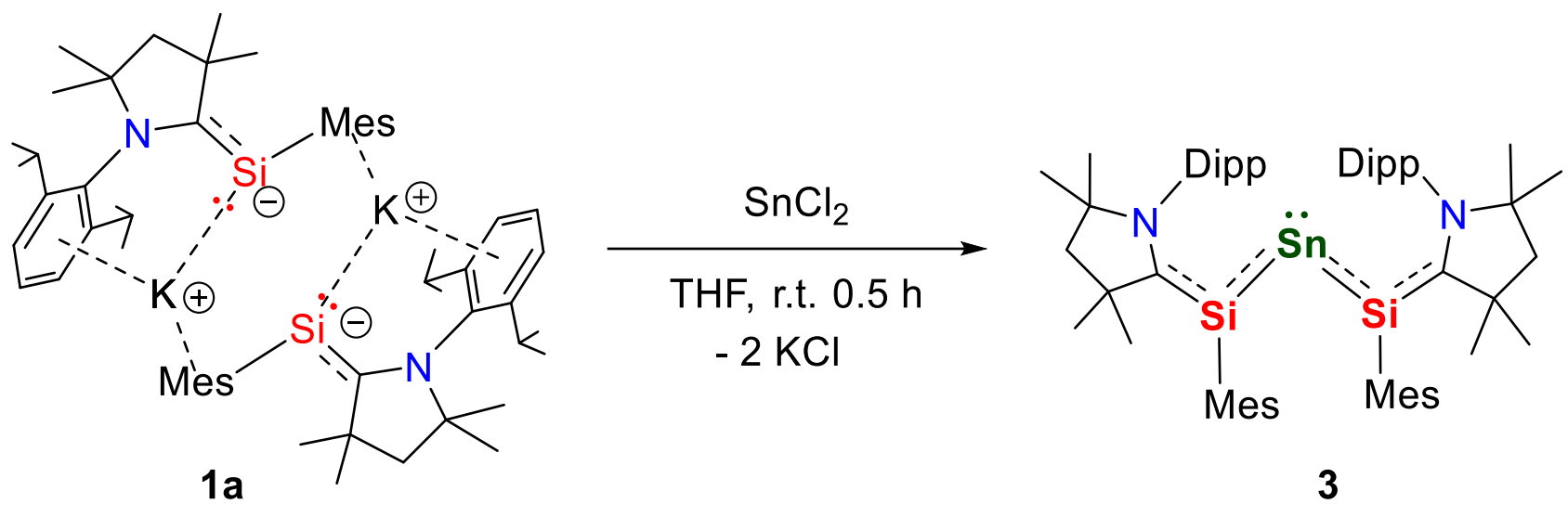


**Scheme S4**. synthesis of **3**.

A solution of $SnCl_2$ (10.0 mg, 0.05 mmol) in THF (10.0 mL) was slowly added to a THF solution (10 mL) of compound **1a** (50 mg, 0.05 mmol) at room temperature. After stirring for 0.5 hour at room temperature, the starting material was completely consumed, resulting in a dark green solution. All volatiles in this solution were removed under vacuum, affording a deep green solid. The mixture was then extracted with pentane and filtered, producing a green solution. Concentration of the pentane solution yielded compound **3** as a green solid (46.9 mg, 0.05 mmol, 90% yield). Single crystal of compound **3** suitable for X-ray diffraction were obtained through the gradual evaporation of a concentrated solution of HMDSO at -30°C. **M.P.** 95 °C (decomp.).

**$^{1}$H NMR** (400 MHz, THF-$d_8$, 298K): δ (ppm) 7.32 (dd, $J$ = 8.2, 7.1 Hz, 2H), 7.20 (d, $J$ = 7.6 Hz, 4H), 6.38 (s, 4H), 3.10 (hept, $J$ = 7.0 Hz, 4H), 2.22 (s, 6H), 2.07 (s, 12H), 1.91 (s, 4H), 1.30 (d, $J$ = 6.7 Hz, 12H), 1.26 – 1.21 (m, 24H), 1.02 (s, 12H).

**$^{13}$C{$^{1}$H} NMR** (101 MHz, $C_6D_6$, 298K): δ (ppm) 194.1 (s, *C*), 147.8 (s, *C*), 146.5 (s, *C*), 143.8 (s, *C*), 137.1 (s, *C*), 136.7 (s, *C*), 129.6 (s, *C*H), 128.6 (s, *C*H), 128.4 (s, *C*H), 128.2 (s, *C*H), 124.1 (s, *C*H), 68.0, 58.1 (s, $CH_2$ in the five-member ring of cAAC), 49.8, 31.6, 30.0, 29.0, 28.9, 27.0, 25.7, 24.3, 21.5.

**$^{29}$Si{$^{1}$H} NMR** (79 MHz, $C_6D_6$, 298K): δ (ppm) 121.0

**$^{119}$Sn{$^{1}$H} NMR** (150 MHz, $C_6D_6$, 298K): δ (ppm) 1737.9.

**HR-MS(ESI)**: (m/z) calcd for $[M-H]^+$($C_{58}H_{85}N_2Si_2Sn^+$) 985.5268, found 985.5267.

**IR** (cm$^{-1}$): 2960.92 (w), 2915.09 (w), 2859.08 (w), 1453.05 (w), 1436.49 (w), 1380.85 (w), 1364.55 (w), 1325.01 (w), 1292.60 (m), 1264.44 (w), 1249.05 (w), 1224.41 (w), 1186.72 (m), 1165.19 (s), 1130.72 (s), 1082.26 (w), 1049.48 (w), 1039.28 (w), 1015.07 (s), 996.89 (w), 975.20 (w), 964.00 (w), 928.21 (w), 906.34 (w), 883.94 (w), 839.56 (w), 802.84 (m), 784.23 (w).

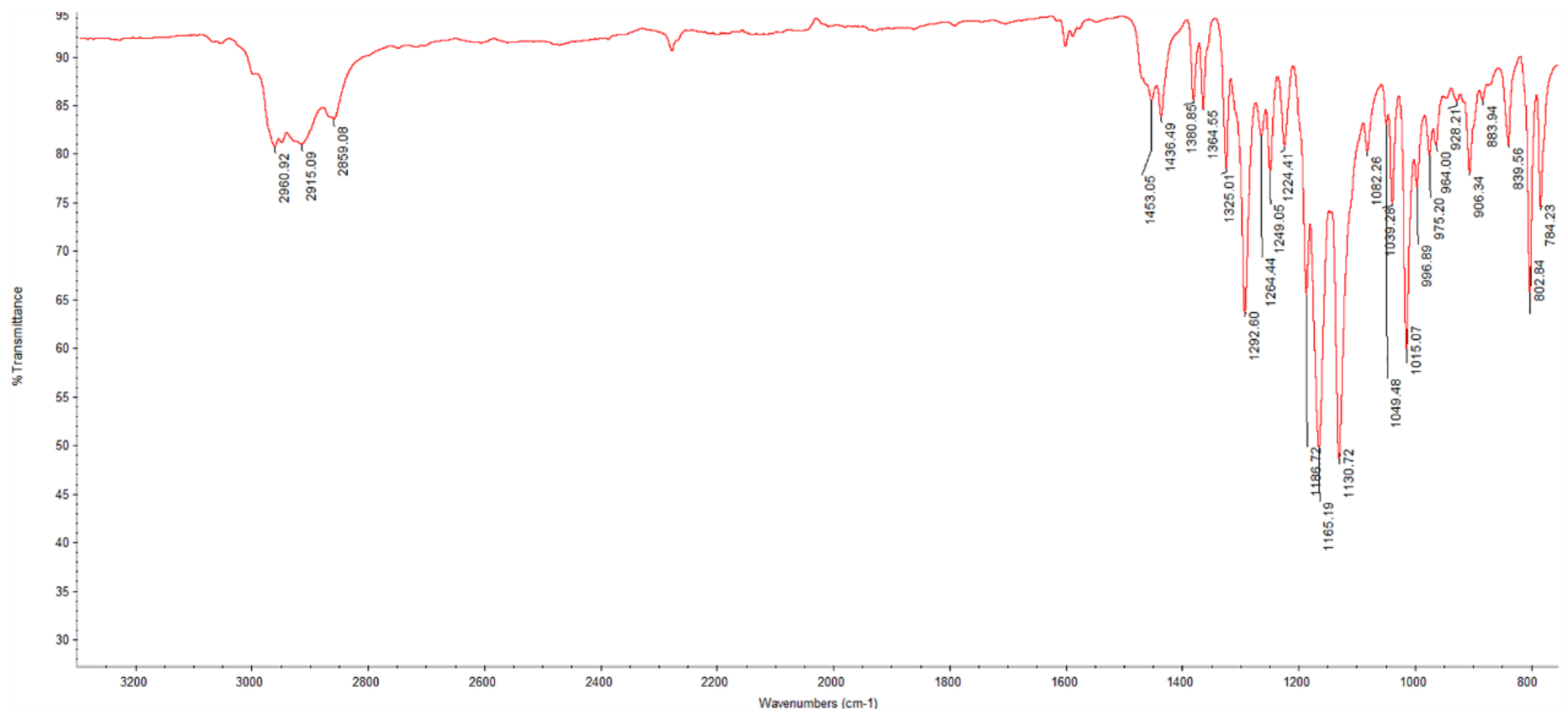

**Figure S19.** IR spectrum of compound **3**.

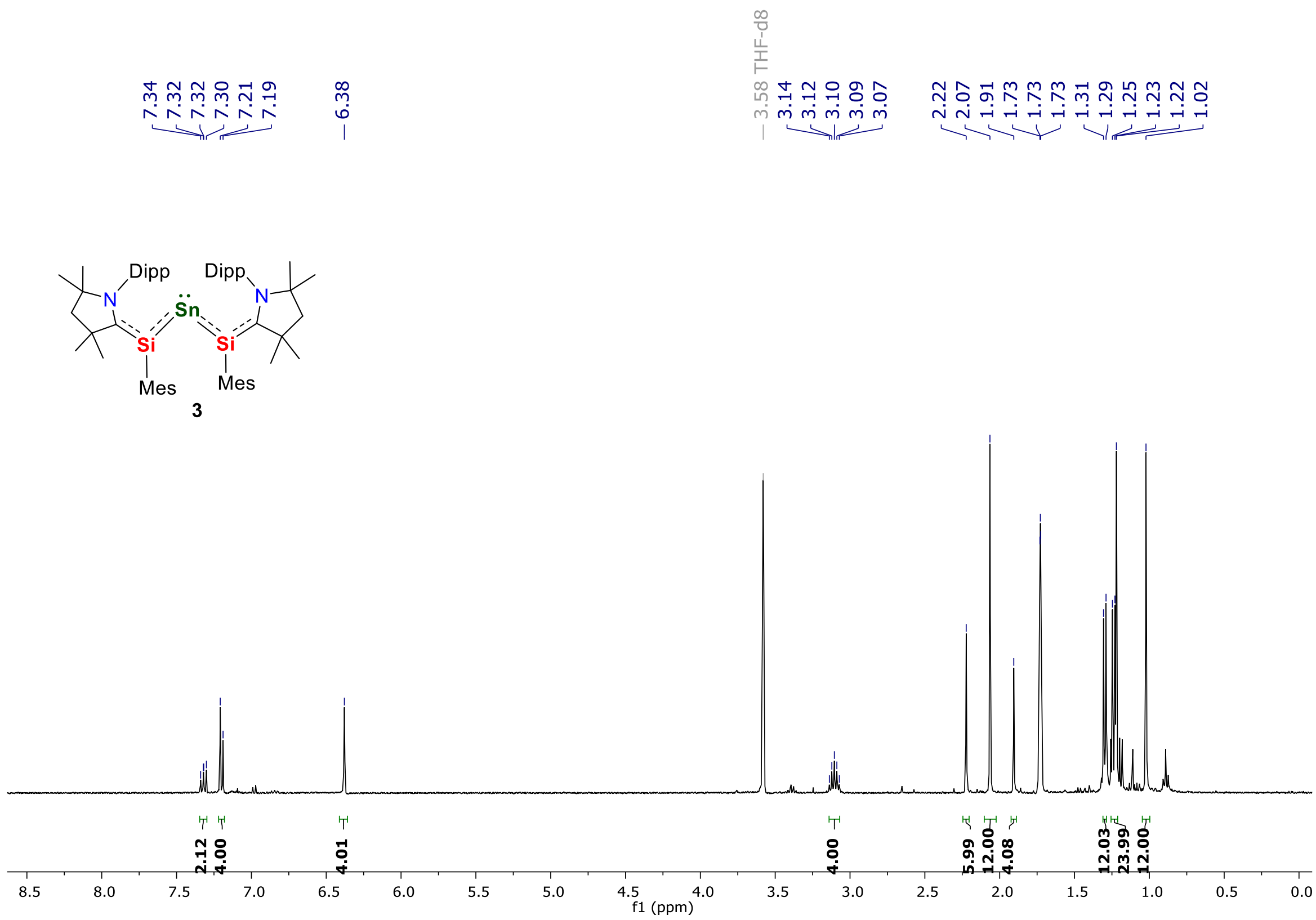

**Figure S20**. $^{1}$H NMR spectrum of **3** (400 MHz, THF-*d8*, 298K).

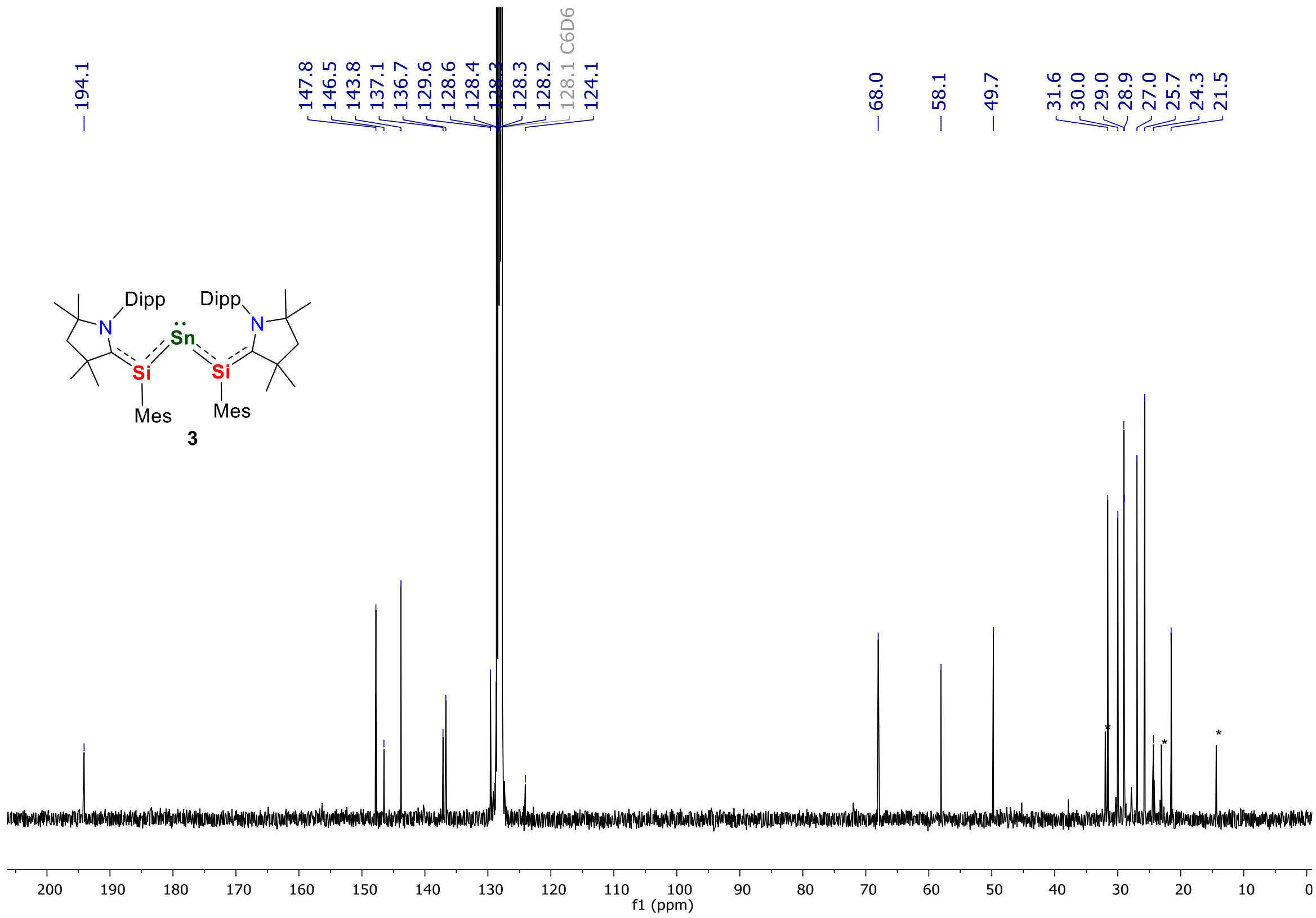


**Figure S21.** $^{13}C\{^{1}H\}$ NMR spectrum of **3** (101 MHz, $C_6D_6$, 298K). * marks the residue of n-hexane.

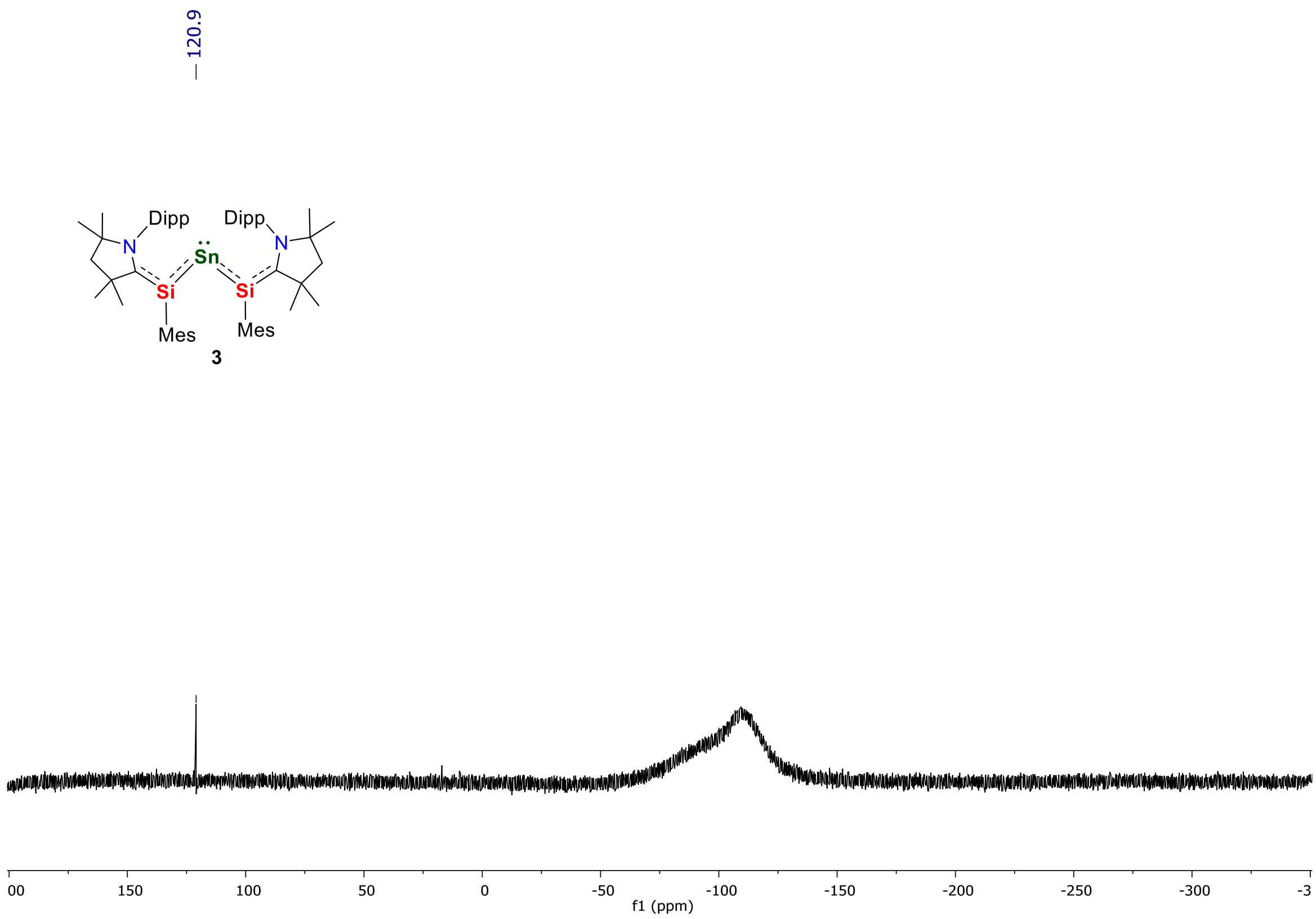


**Figure S22**. $^{29}Si\{^{1}H\}$ NMR spectrum of **3** (79 MHz, $C_6D_6$, 298K).

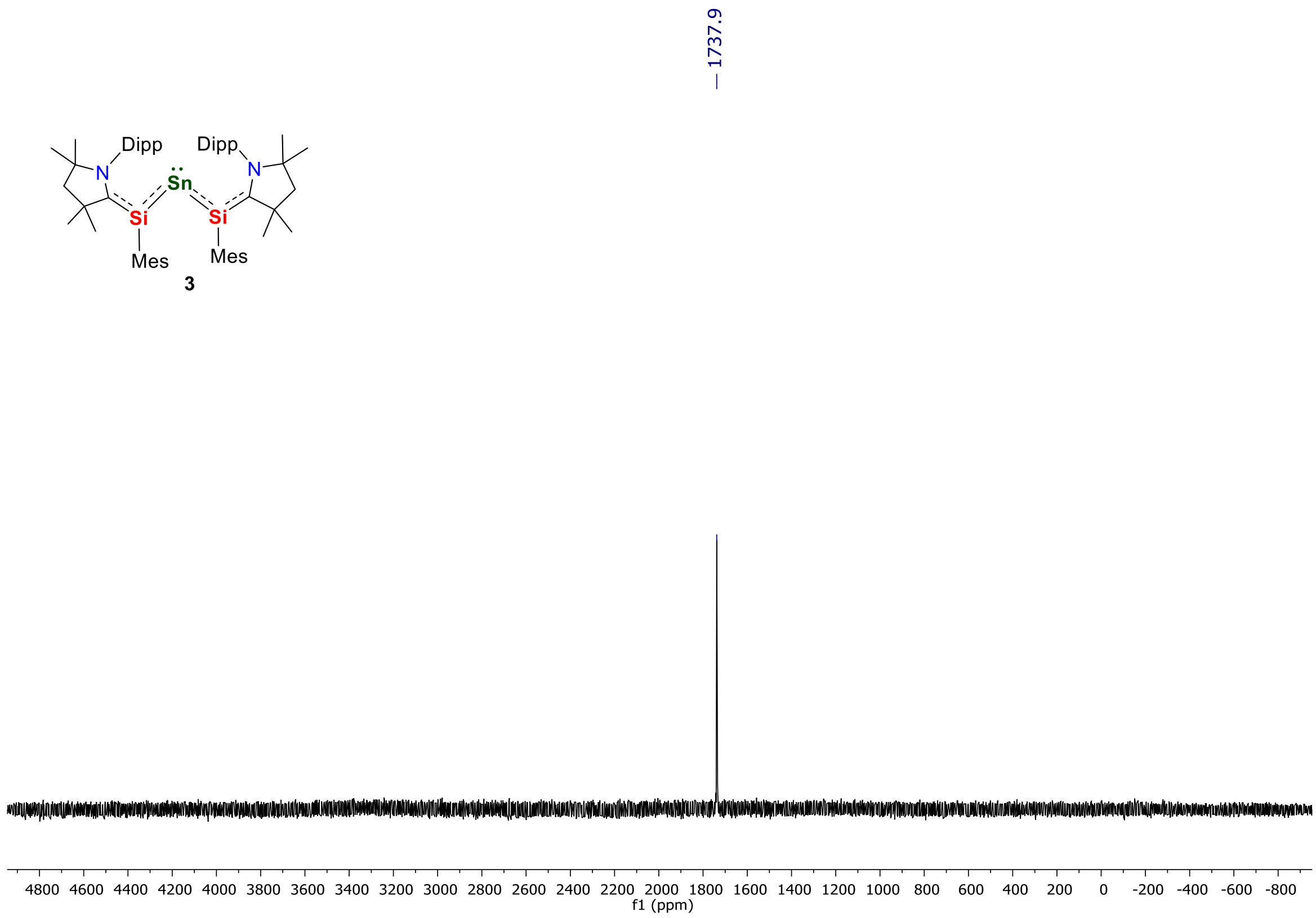


**Figure S23**. $^{119}Sn\{^1H\}$ NMR spectrum of **3** (150 MHz, $C_6D_6$, 298K).

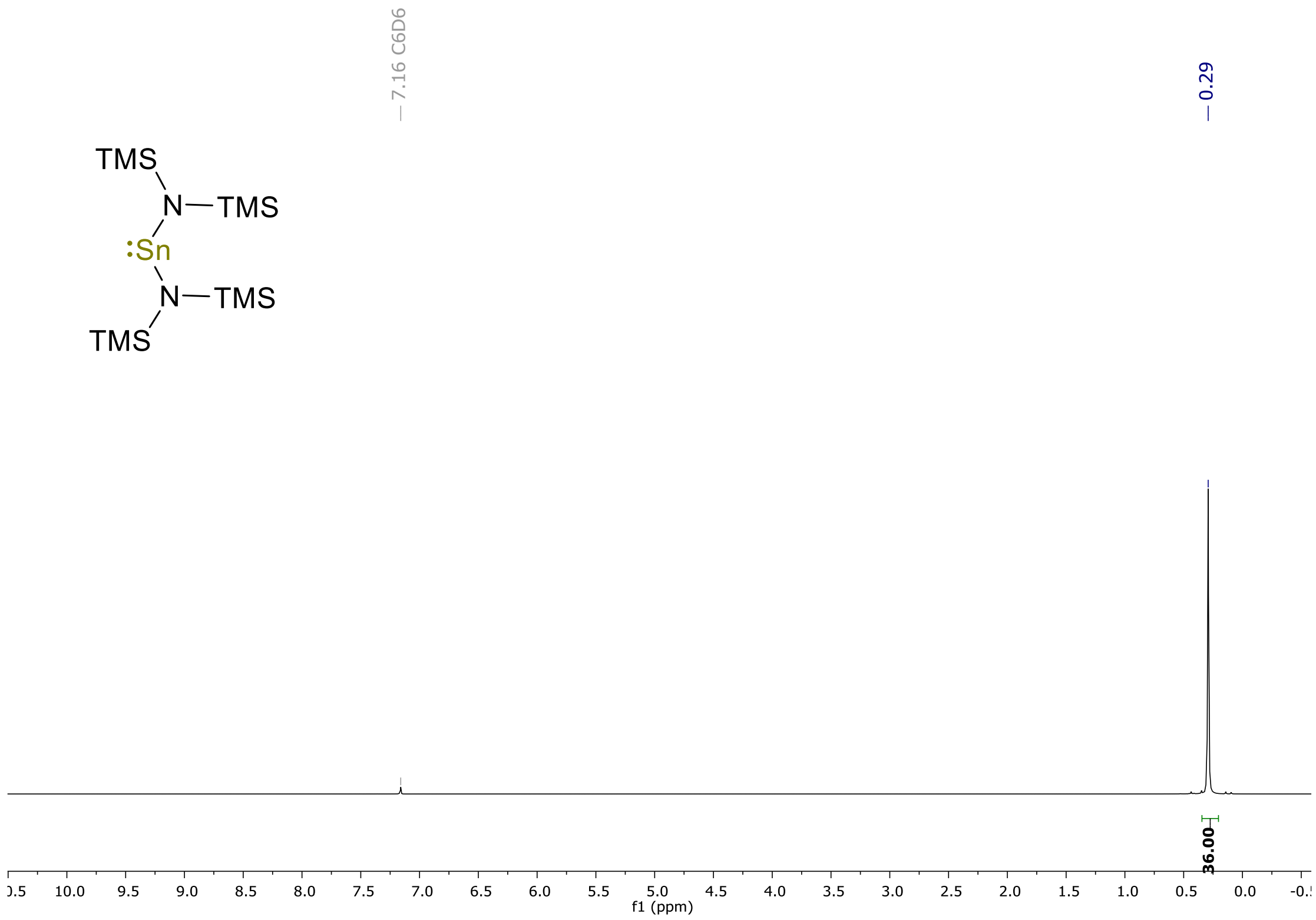


**Figure S24**. $^1H$ NMR spectrum of Lappert stannylene (**E**,400 MHz, $C_6D_6$, 298K).

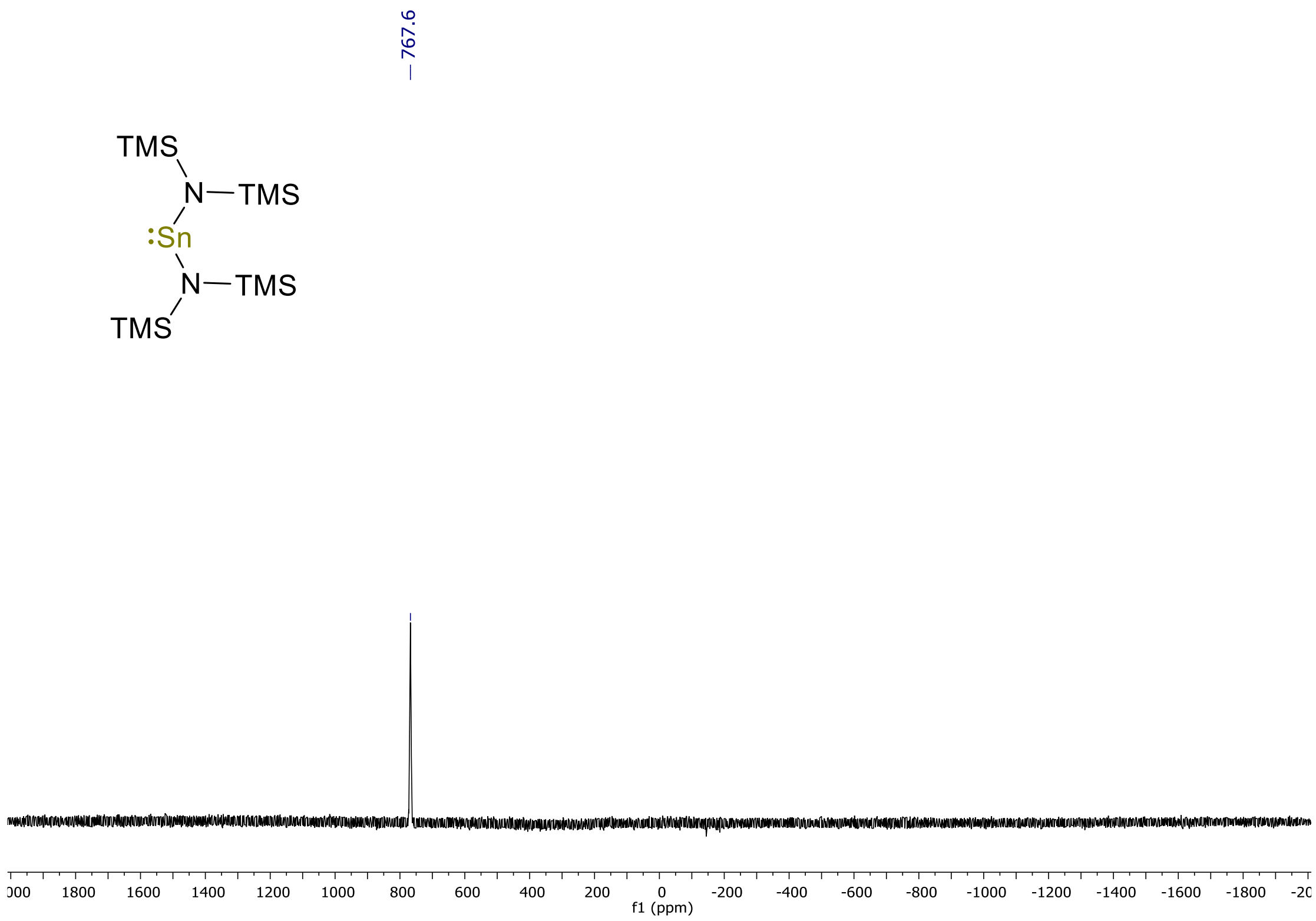


**Figure S25**. $^{119}Sn\{^1H\}$ NMR spectrum of Lappert stannylene (**E**, 150 MHz, $C_6D_6$, 298K).

Synthesis of **4**

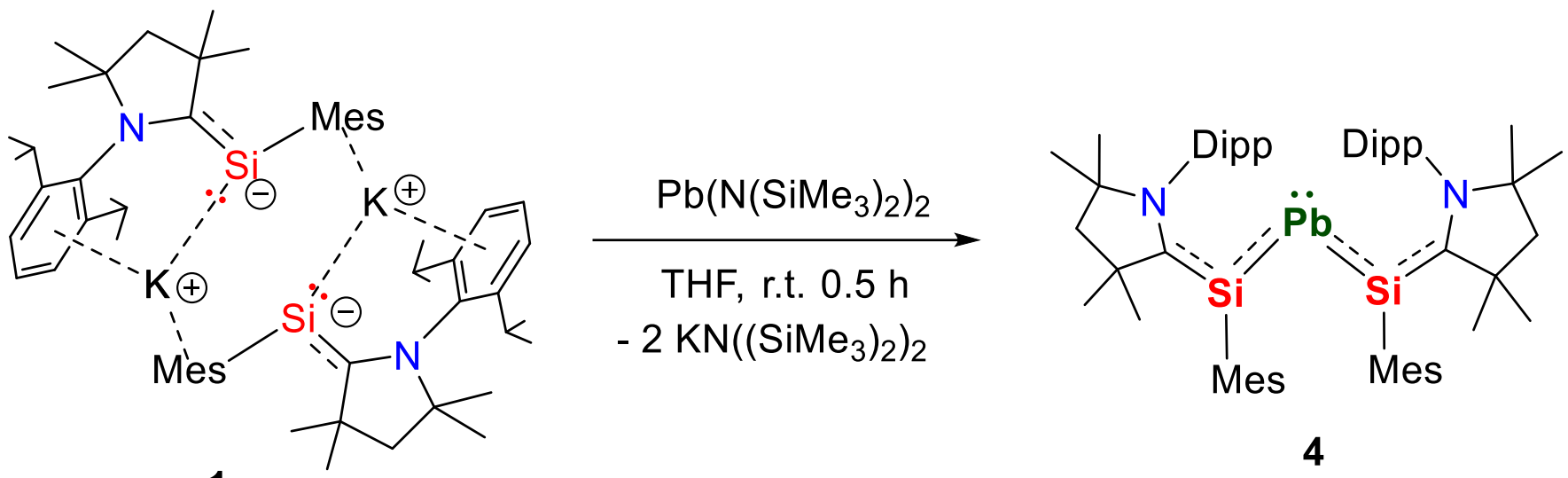


**Scheme S5**. Synthesis of **4**.

A solution of $Pb(N(SiMe_3)_2)_2$ (22.4 mg, 0.04 mmol) in THF (10.0 mL) was added dropwise to a separate THF solution (10.0 mL) of compound **1a** (40 mg, 0.04 mmol) at room temperature. The reaction mixture was stirred for 30 min, during which the starting material was fully consumed, affording a brown solution. Removal of the volatiles under reduced pressure yielded a black-green solid. The residue was extracted with HMDSO and filtered to give a green solution. Concentration of the HMDSO extract by heating at 70 °C under vacuum overnight afforded compound **4** as a green solid (33 mg, 0.03 mmol, 76%). Single crystals suitable for X-ray diffraction were obtained by slow evaporation of a concentrated HMDSO solution of **4** at –30 °C. **M.P.** 80 °C (decomp.).

The same synthetic method produces compound **4** by reacting **1b** with $Pb(N(SiMe_3)_2)_2$.

**$^1$H NMR** (400 MHz, $C_6D_6$, 298K): δ (ppm) 7.24 – 7.19 (m, 6H), 6.78 (s, 4H), 3.26 (q, *J* = 6.8 Hz, 4H), 2.50 (s, 12H), 2.46 (s, 6H), 1.75 (s, 4H), 1.37 (d, *J* = 6.6 Hz, 12H), 1.32 (d, *J* = 6.7 Hz, 12H), 1.30 (s, 12H), 1.16 (s, 12H).

**$^{13}$C{$^1$H} NMR** (101 MHz, $C_6D_6$, 298K): δ (ppm) 202.7 (s, *C*), 166.4 (s, *C*), 149.3 (s, *C*), 145.1 (s, *C*), 137.4 (s, *C*), 136.1 (s, *C*), 129.7 (s, *C*H), 128.9 (s, *C*H), 128.6 (s, *C*H), 128.4 (s, *C*H), 127.9 (s, *C*H), 66.8, 58.9 (s, $CH_2$ in the five-member ring of cAAC), 50.7, 33.2, 29.8, 29.5, 28.8, 26.1, 25.4, 21.3.

**$^{29}$Si{$^1$H} NMR** (79 MHz, $C_6D_6$, 298K): δ (ppm) 309.10.

Attempts to record the **$^{207}$Pb{$^1$H} NMR** spectrum of **4** measured in $C_6D_6$ at high concentration ca. 0.1 M over −3300 to +1600 ppm; no detectable resonance was observed.

**HR-MS(ESI)**: (m/z) calcd for $[M]^+$($C_{58}H_{84}N_2Si_2Pb^+$) 1027.5934, found 1027.5934.

**IR** ($cm^{-1}$): 2959.28 (w), 2867.14 (w), 1436.48 (w), 1381.30 (w), 1363.10 (w), 1323.88 (m), 1290.03 (m), 1248.84 (m), 1225.46 (m), 1187.22 (m), 1164.65 (s), 1132.87 (s), 1081.10 (w), 1039.53 (m), 1017.63 (m), 995.53 (m), 928.30 (w), 905.67 (m), 842.49 (m), 804.89 (m), 782.67 (m), 748.85 (w), 698.14 (w), 606.91 (w), 582.63 (w).

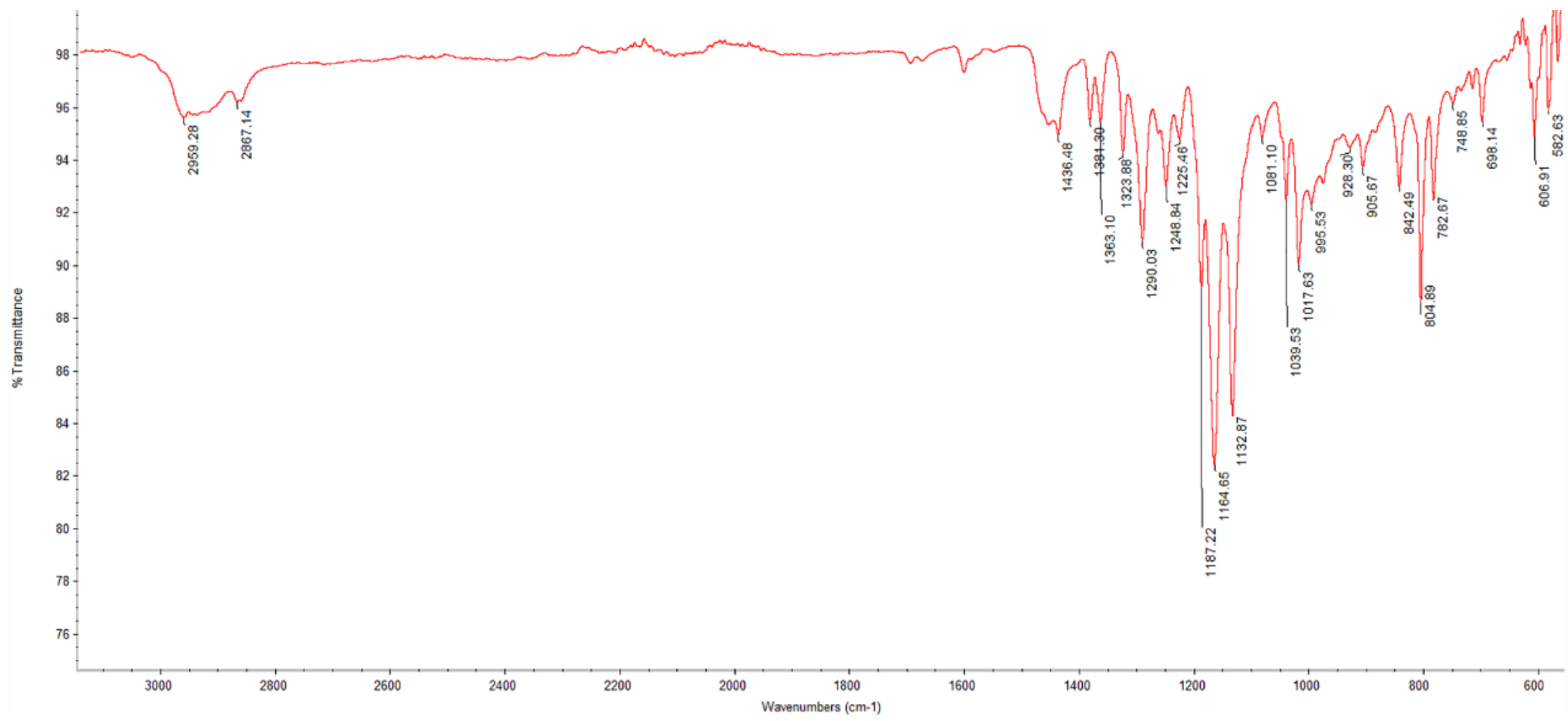


**Figure S26.** IR spectrum of compound **4**.

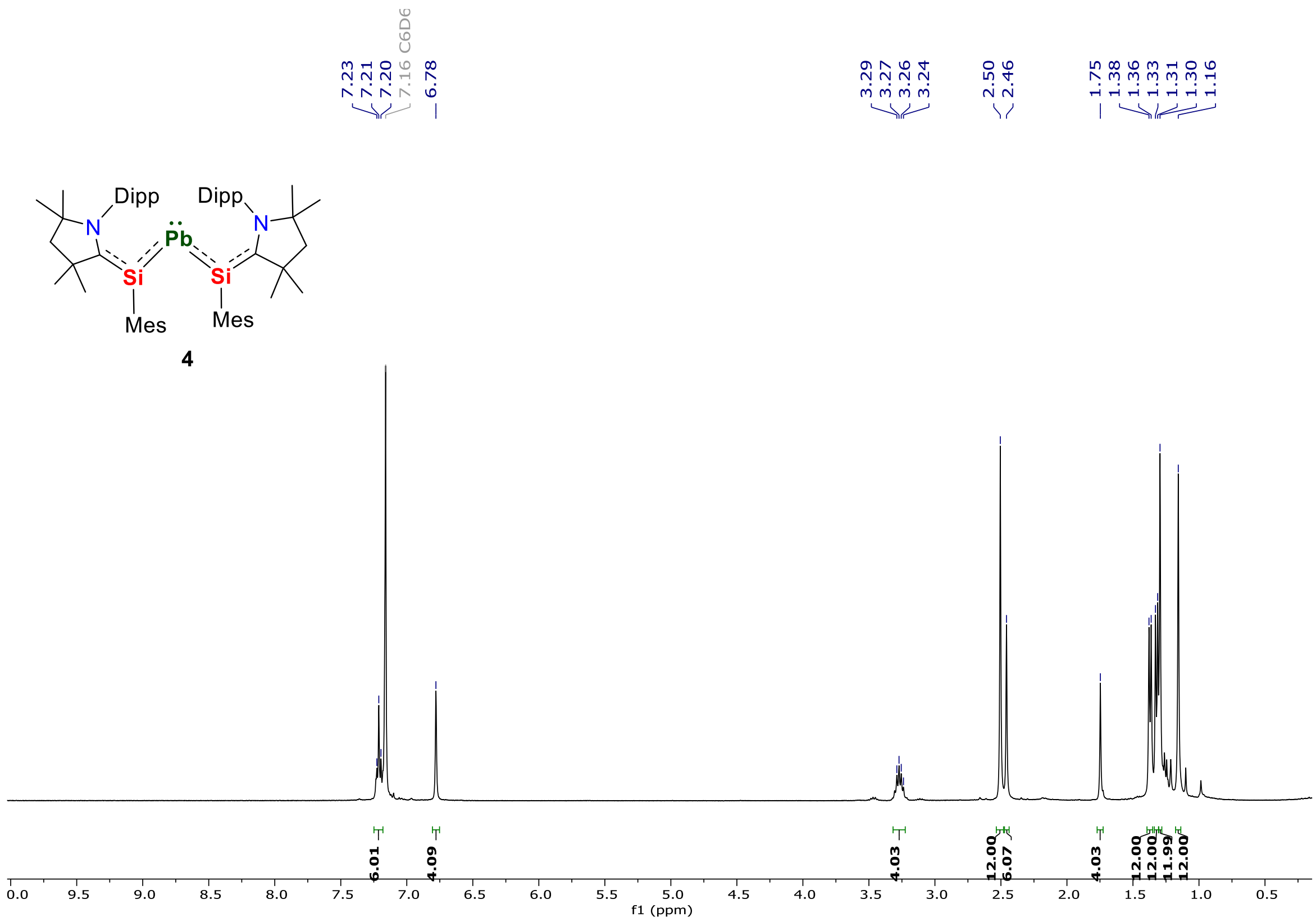


**Figure S27**. $^{1}$H NMR spectrum of **4** (400 MHz, $C_6D_6$, 298K).

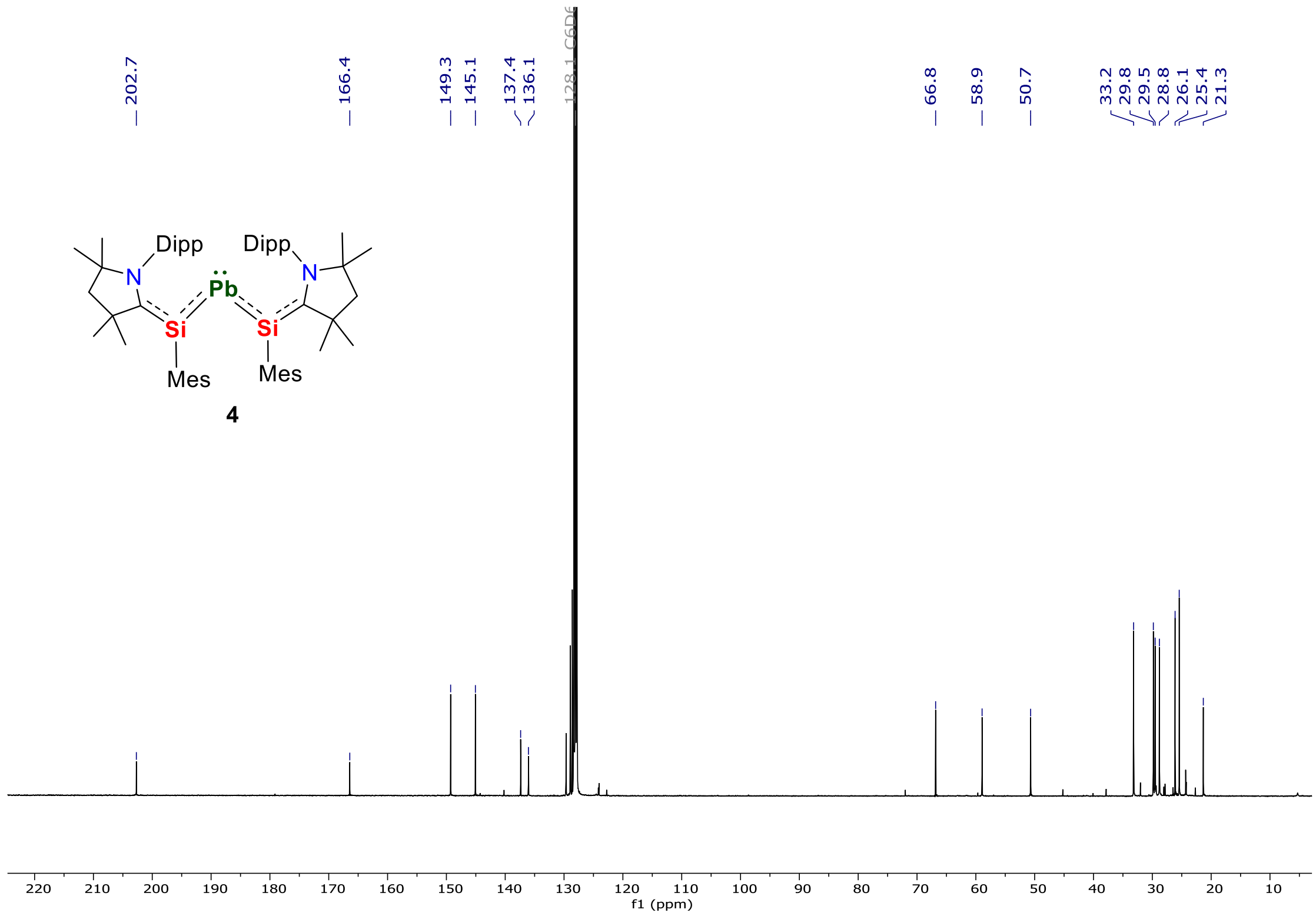


**Figure S28.** $^{13}C\{^{1}H\}$ NMR spectrum of **4** (101 MHz, $C_6D_6$, 298K).

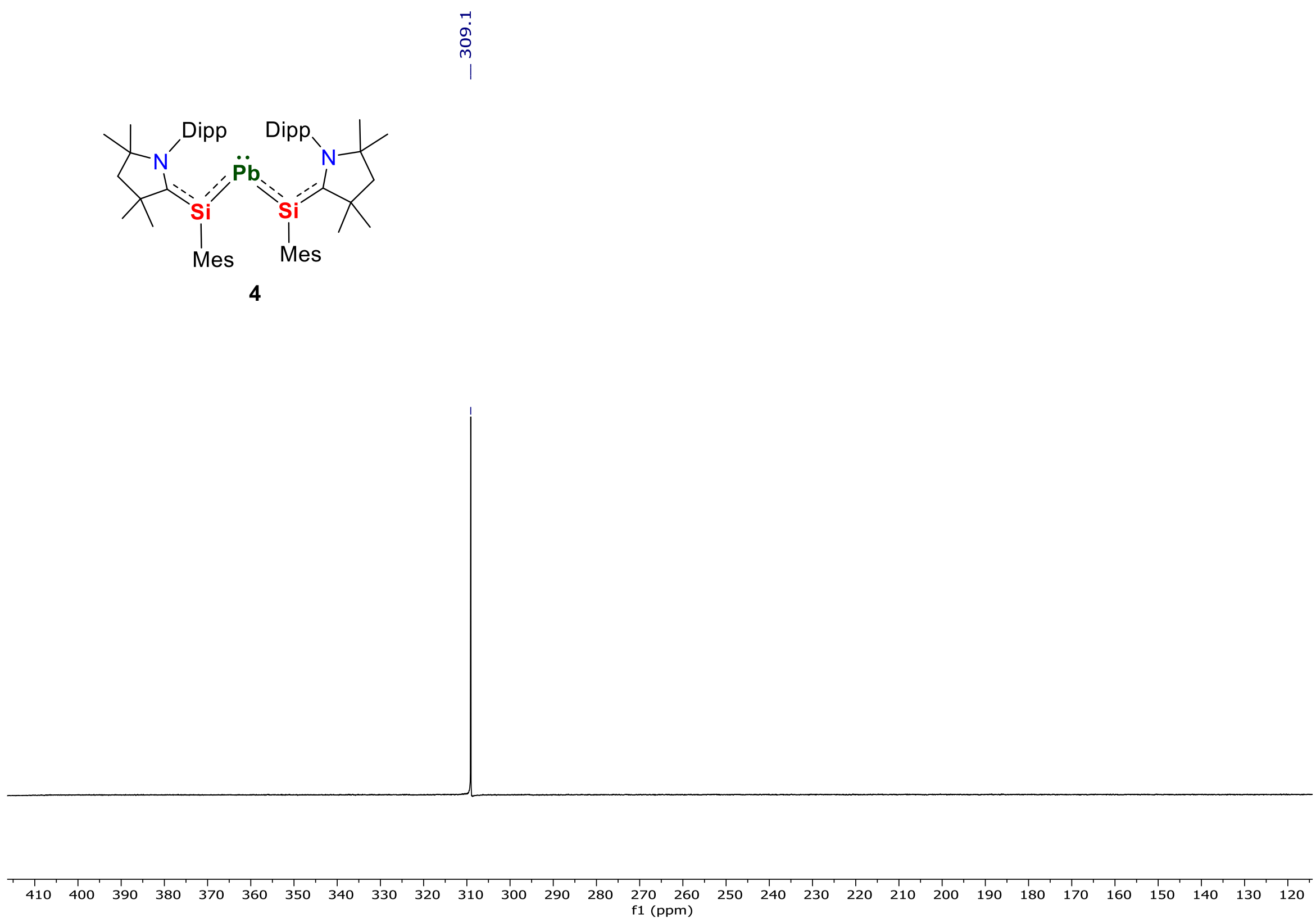


**Figure S29**. $^{29}Si\{^{1}H\}$ NMR spectrum of **4** (79 MHz, $C_6D_6$, 298K).

Synthesis of **2**$^{\bullet-}$[K([2.2.2]-cryptand)]

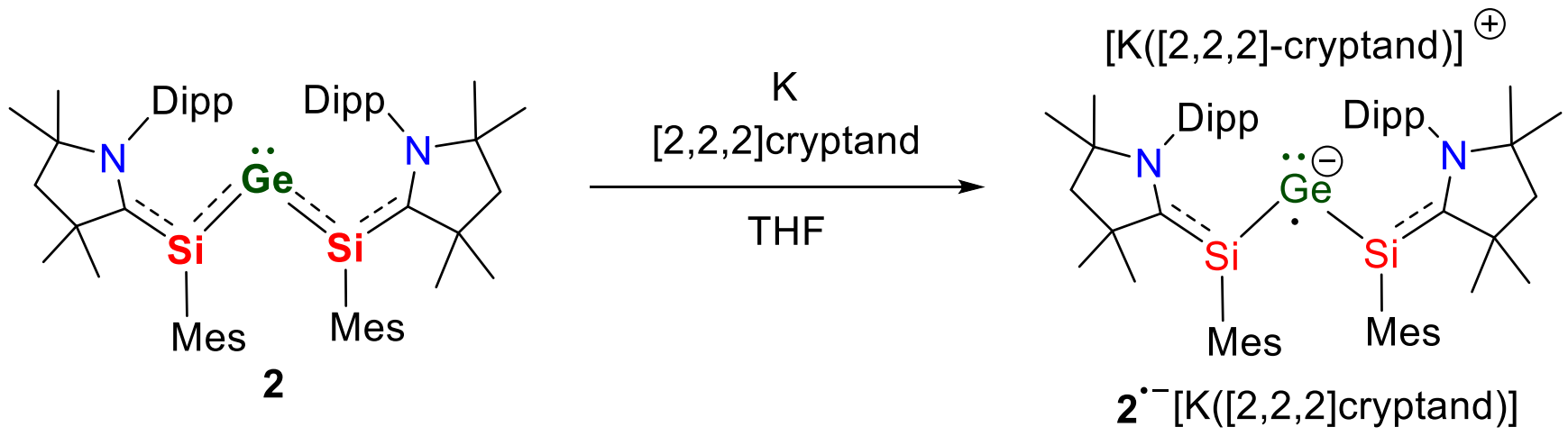


**Scheme S6**. Synthesis of **2**$^{\bullet-}$[K([2.2.2]-cryptand)].

Compound **2** (20 mg, 21.3 µmol), freshly cut potassium (1.0 mg, 25.6 µmol, 1.2 equiv), and [2.2.2]cryptand (9.6 mg, 25.6 µmol, 1.2 equiv) were combined in a vial equipped with a magnetic stir bar. THF (1.0 mL) was added at room temperature to initiate the reaction. Upon addition of THF, the reaction mixture gradually changed color from green to purple. After stirring for ca. 5 min, pentane (10 mL) was added to quench the reaction. The magnetic stir bar and any residual potassium metal were carefully removed, and the resulting THF/pentane mixture was left undisturbed. After ca. 10 min, a dark purple precipitate formed. The supernatant was carefully decanted, and the solid was washed twice with pentane. The resulting dark purple solid was dissolved in THF and filtered through a pre-rinsed glass microfiber filter. Removal of volatiles under reduced pressure afforded **2**$^{\bullet-}$[K([2.2.2]-cryptand)] as a purple solid in approximately 65% yield (18.7 mg, 13.9 µmol). Single crystals of **2**$^{\bullet-}$[K([2.2.2]-cryptand)] suitable for X-ray diffraction were obtained by cooling a THF/$Et_2O$/pentane solution to −30 °C.

**Note:** Due to the high instability of **2**$^{\bullet-}$[K([2.2.2]-cryptand)] in both solution and the solid state, attempts to obtain a mass spectrum were unsuccessful under the conditions employed. Nevertheless, its formation is supported by the combined EPR spectroscopy, UV-vis spectroscopy, single-crystal X-ray diffractionand DFT calculations.

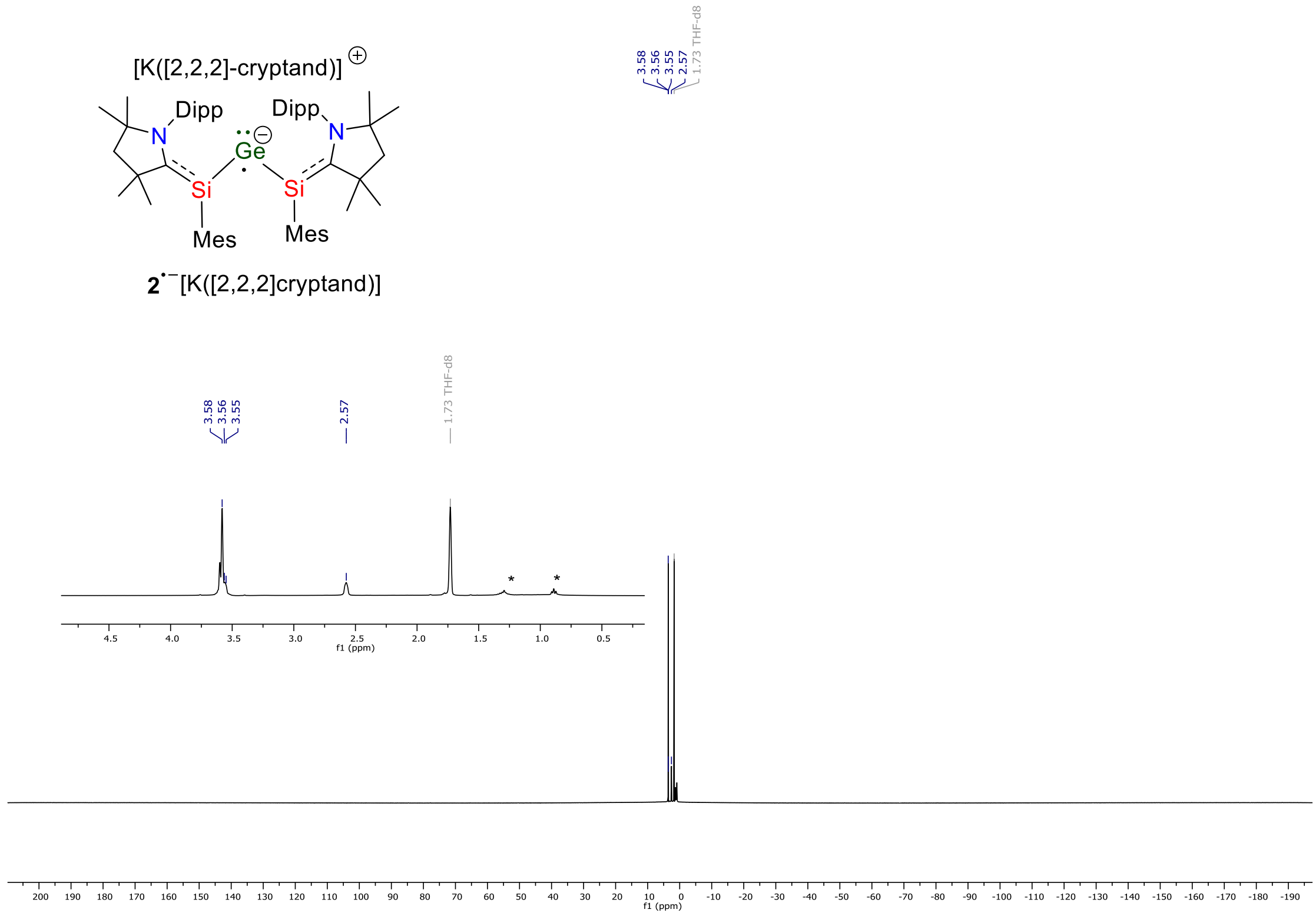


**Figure S30**. $^{1}$H NMR spectrum of **2•−**[K([2.2.2]-cryptand)] (400 MHz, THF-$d_8$, 298 K). *marks the residue of n-pentane, only signals corresponding to the [K([2.2.2]-cryptand)] cation at 2.57 ppm and 3.55 ppm are observed.

Synthesis of **3**•[K([2.2.2]-cryptand)]

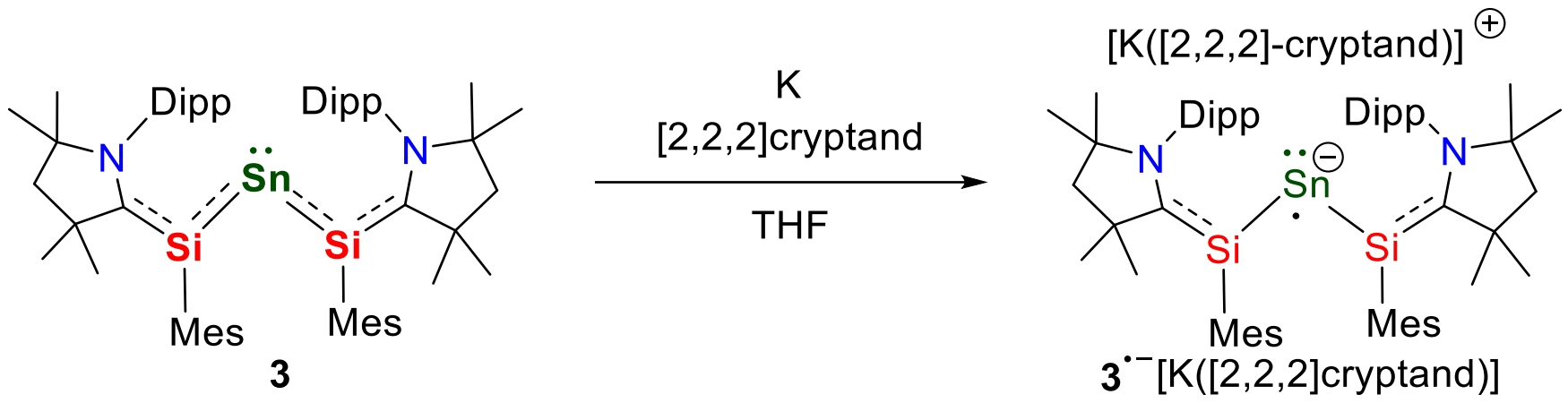


**Scheme S7**. Synthesis of **3**•⁻[K([2.2.2]-cryptand)].

**Method 1**: Compound **3** (10 mg, 10.1 µmol), freshly cut potassium (0.5 mg, 12.2 µmol, 1.2 equiv), and [2.2.2]cryptand (4.6 mg, 12.2 µmol, 1.2 equiv) were combined in a vial equipped with a magnetic stir bar. THF (0.5 mL) was added at room temperature to initiate the reaction. Upon addition of THF, the reaction mixture gradually changed in color from green to black. After stirring for approximately 5 min, pentane (5 mL) was added to quench the reaction. **Caution:** Prolonged reaction times should be avoided, as the product readily decomposes to the potassium silanylidene salt **1b**. In addition, even a slight excess of potassium can result in over-reduction, likewise leading to the formation of **1b**. The magnetic stir bar and any residual potassium metal were carefully removed, and the resulting THF/pentane mixture was left undisturbed. After ca. 10 min, a black precipitate formed. The supernatant was carefully decanted, and the black solid was washed twice with pentane. The crude product was dissolved in THF and filtered through a pre-rinsed glass microfiber filter. Removal of volatiles under reduced pressure afforded **3**•⁻[K([2.2.2]-cryptand)] as a black solid. Single crystals of **3**•⁻[K([2.2.2]-cryptand)] suitable for X-ray diffraction were obtained by cooling a THF/$Et_2O$/pentane solution to −30 °C.

**Method 2:** A THF solution of $KC_8$ (1.5 mg, 11.2 µmol, 1.1 equiv) and [2.2.2]cryptand (4.2 mg, 11.2 µmol, 1.1 equiv) was added dropwise to a THF solution of compound **3** (10 mg, 10.1 µmol) at room temperature. After stirring for ca. 2 min, the reaction mixture turned black. Subsequent addition of pentane (5 mL) afforded a THF/pentane mixture, resulting in the formation of a black precipitate. The solvent was removed, and the resulting solid was washed with pentane. The crude product was dissolved in THF and filtered through a pre-rinsed glass microfiber filter. Removal of volatiles under reduced pressure afforded **3**•⁻[K([2.2.2]-cryptand)] as a black solid.

**Note**: Due to the high instability of **3**•⁻[K([2.2.2]-cryptand)] in both solution and the solid state, partial decomposition was observed even during filtration through a glass frit under glovebox conditions. Attempts to obtain mass spectrometric data and the isolated yield were unsuccessful, owing to decomposition during handling. Nevertheless, the formation of **3**•[K([2.2.2]-cryptand)] is unambiguously supported by EPR spectroscopy, UV-vis spectroscopy, single-crystal X-ray diffraction, and DFT calculations.

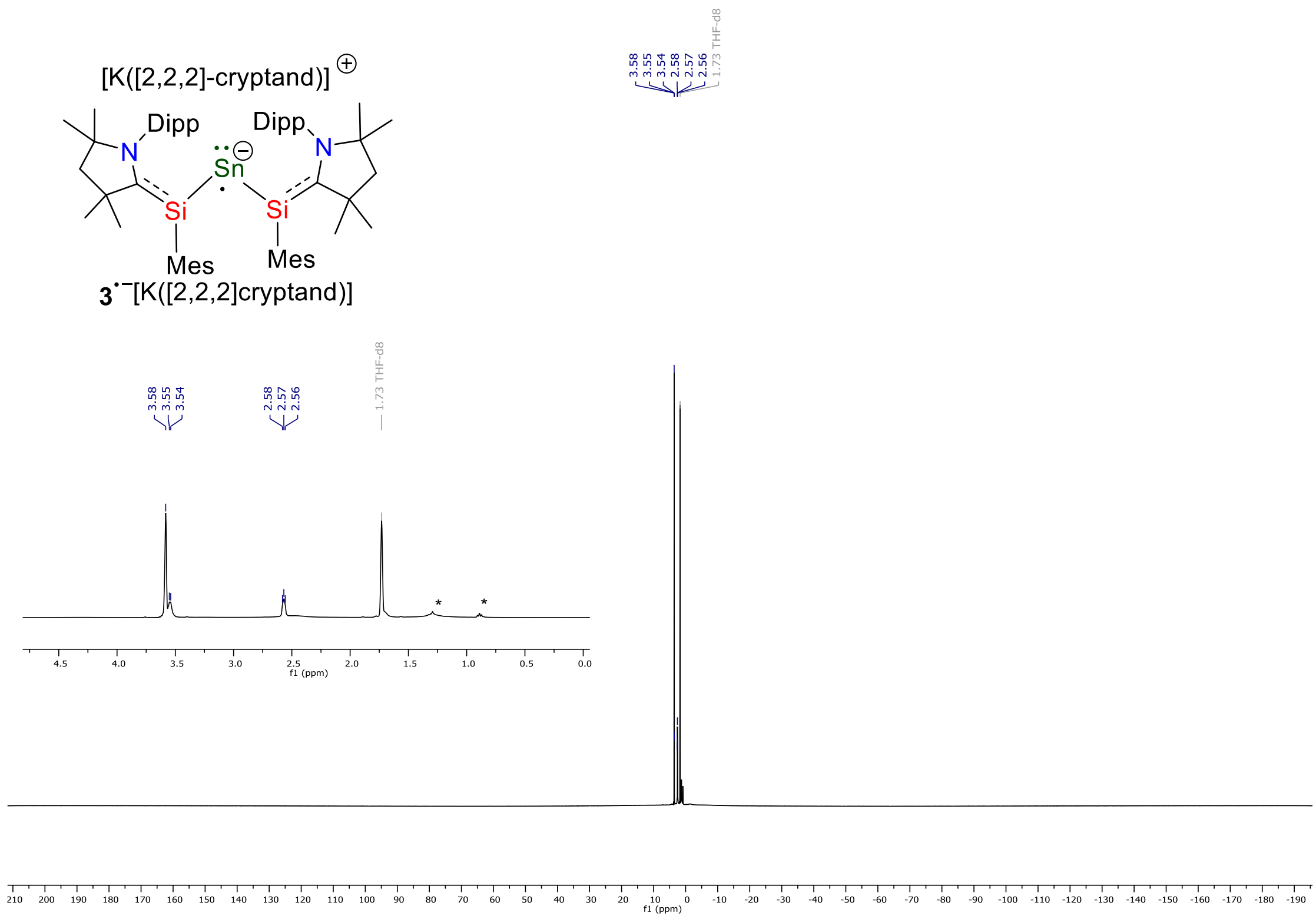


**Figure S31**. $^{1}$H NMR spectrum of **3**$^{\bullet-}$[K([2.2.2]-cryptand)] (400 MHz, THF-*d₈*, 298 K). *marks the residue of n-pentane, only signals corresponding to the [K([2.2.2]-cryptand)] cation at 2.57 ppm and 3.55 ppm are observed.

Chemical Oxidation of $\mathbf{2}^{\bullet-}$[K([2.2.2]-cryptand)] with $FcBAr^F_4$

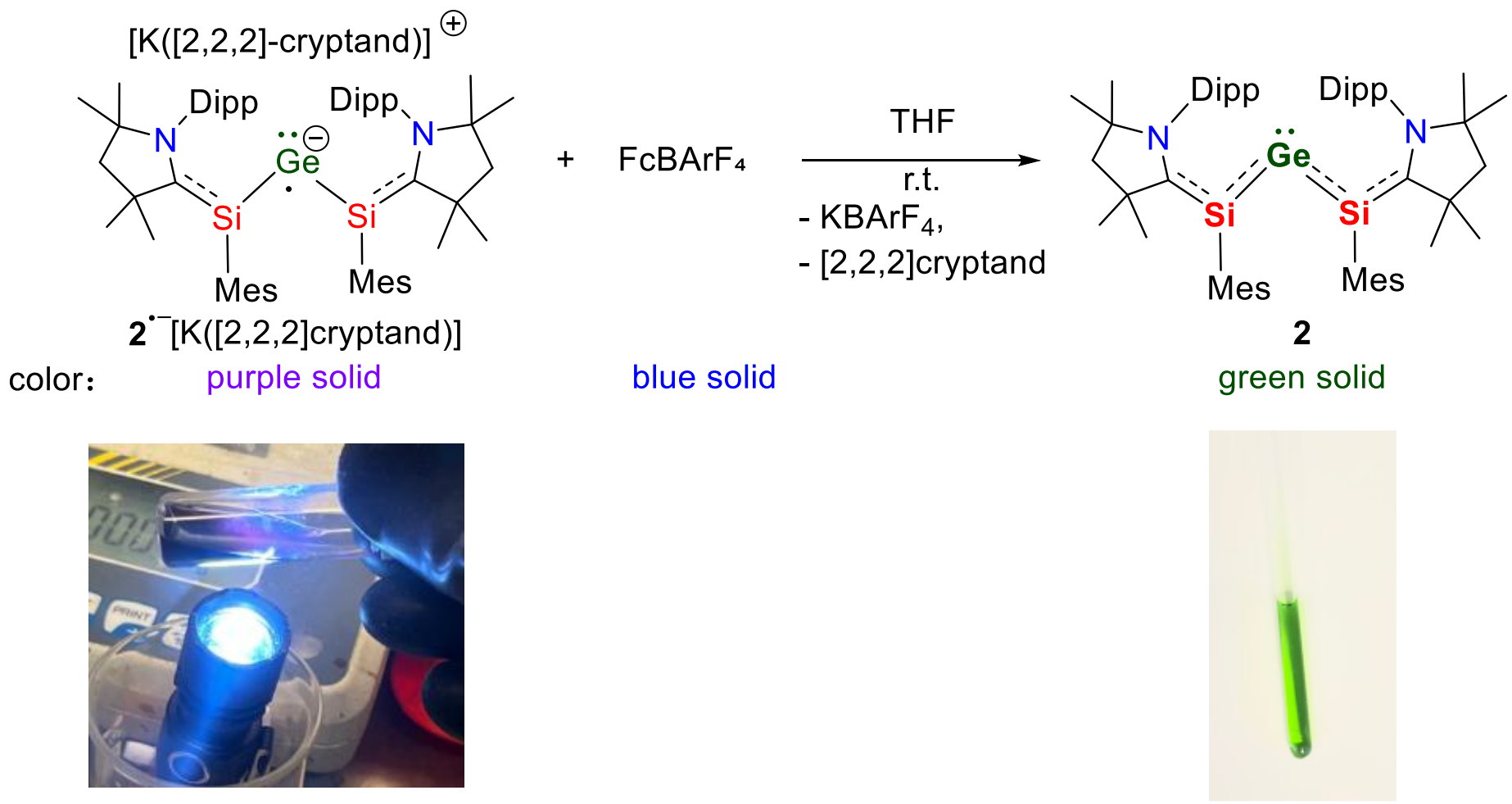


**Scheme S8**. Oxidation of $\mathbf{2}^{\bullet-}$[K([2.2.2]-cryptand)] with $FcBAr^F_4$.

A THF solution of $FcBAr^F_4$ (4 mg, 3.7 µmol; $Ar^F$=3,5-$(CF_3)_2C_6H_3$) was added dropwise to a THF solution of $\mathbf{2}^{\bullet-}$[K([2.2.2]-cryptand)] (5.0 mg, 3.7 µmol) at room temperature. Upon addition, the reaction mixture gradually changed color from purple to green. After stirring for 2 min, the solvent was removed under reduced pressure. The residue was extracted with *n*-hexane to afford compound **2** as a green solid (3.1 mg, 3.3 µmol, 90%). The $^1$H NMR spectrum of the crude mixture (Figure S24) shows signals corresponding to compound **2**, ferrocene (Fc), [2.2.2]cryptand, and residual solvent. These results confirm that $\mathbf{2}^{\bullet-}$[K([2.2.2]-cryptand)] is readily oxidized to **2** by $FcBAr^F_4$.

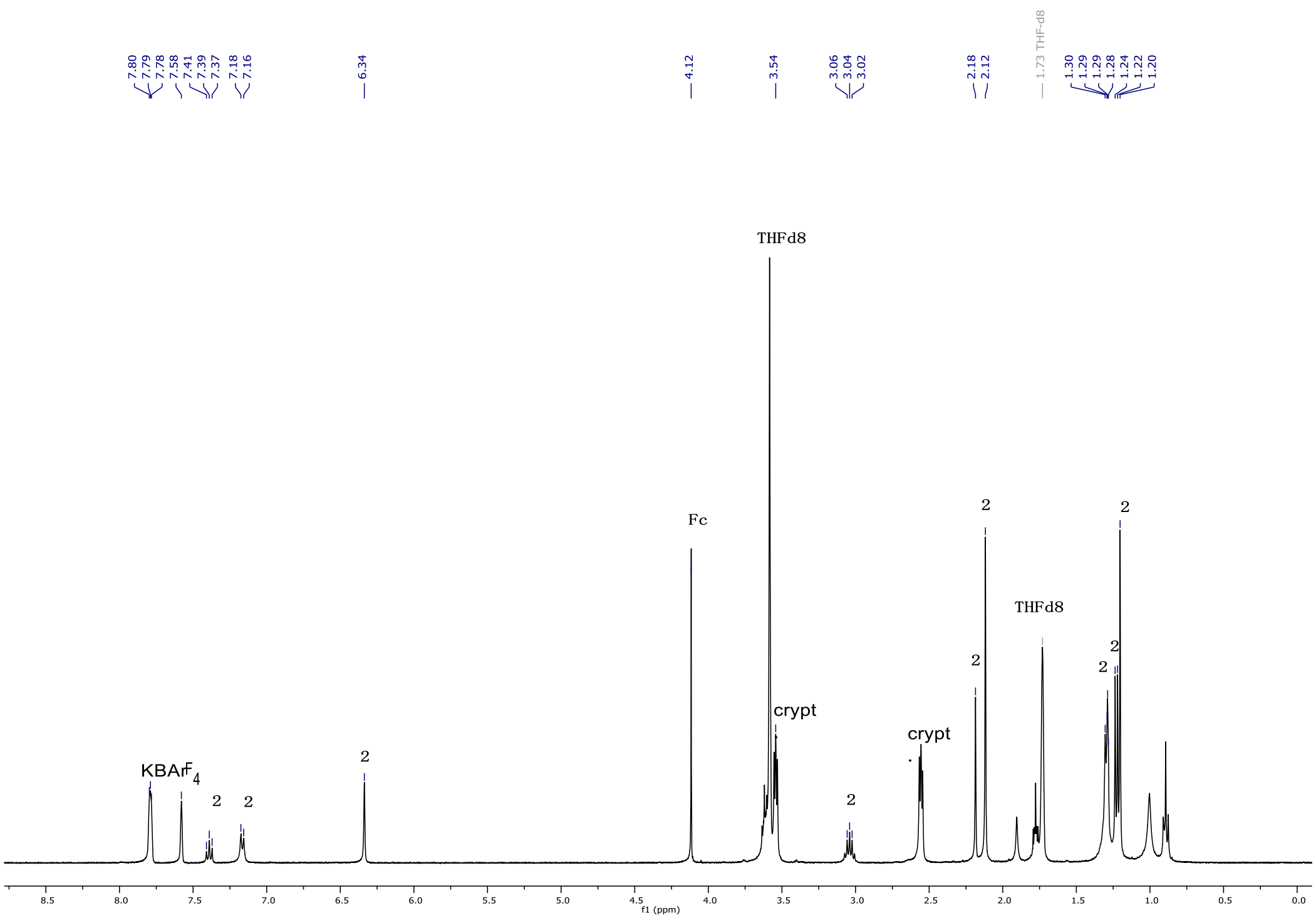


**Figure S32**. $^1$H NMR spectrum of the reaction mixture of $\mathbf{2}^{\bullet-}$[K([2.2.2]-cryptand)] with $FcBAr^F_4$ (400 MHz, THF-$d_8$, 298 K).

## A5 Details of the Single Crystal X-ray Diffraction Analyses

**Table S1**. Selected Si–C distances (Å) of a) **1a**, b) **1b**, and c) Tip-silanylidene anion. ([a] the Pyykkö's standard value for a Si–C double and single bonds)

| | **1a** | **1b** | Tip-silanylidene anion | Si=C / Si–C[[a] |
|---|---|---|---|---|
| Si(1)–C(1) | 1.790(3) | 1.7875(12) | 1.793(2) | 1.74 Å / 1.91 Å |
| Si(1)–C(3) | 1.946(4) | 1.9509(13) | 1.946(2) | |
| Si(2)–C(2) | 1.796(3) | ---- | 1.793(2) | |
| Si(2)–C(4) | 1.948(4) | ---- | 1.946(2) | |
| Si(1)…Si(2) | 5.424 | ---- | 5.415 | |

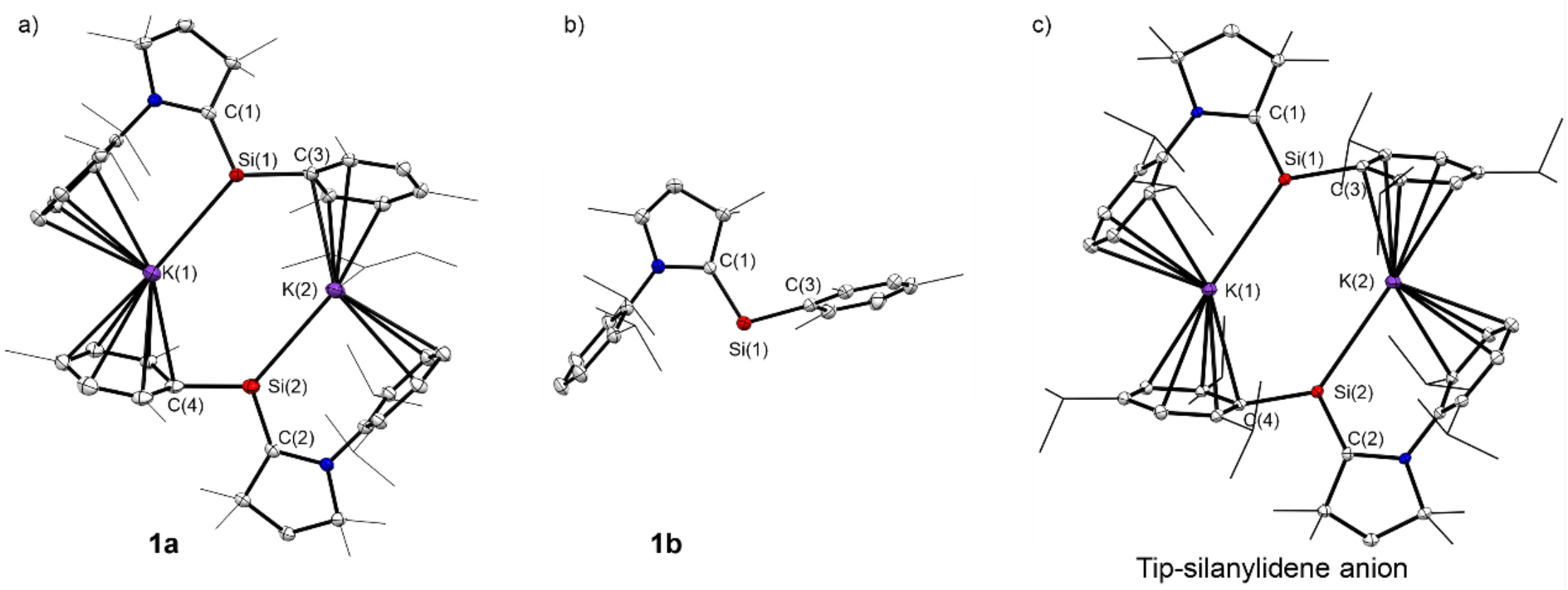

**Table S2**. Crystal data and structure refinement for **1a**.

| | |
|---|---|
| Empirical formula | $C_{62}H_{94}K_2N_2OSi_2$ |
| Formula weight | 1017.77 |
| Temperature/K | 150.15 |
| Crystal system | Monoclinic |
| Space group | $P2_1/n$ |
| a/Å | 19.2284(9) |
| b/Å | 14.0073(7) |
| c/Å | 22.5525(10) |
| α/° | 90 |
| β/° | 99.062(4) |
| γ/° | 90 |
| Volume/Å$^3$ | 5998.4(5) |
| Z | 4 |
| $\rho_{calc}$g/cm$^3$ | 1.127 |
| μ/mm$^{-1}$ | 2.069 |
| F(000) | 2216.0 |
| Crystal size/mm$^3$ | 0.03 × 0.03 × 0.02 |
| Radiation | CuKα (λ = 1.54184) |
| 2Θ range for data collection/° | 5.62 to 144.87 |
| Index ranges | $-23 \leq h \leq 16$, $-14 \leq k \leq 17$, $-27 \leq l \leq 27$ |
| Reflections collected | 25042 |
| Independent reflections | 11583 [$R_{int}$ = 0.0866, $R_{sigma}$ = 0.1048] |
| Data/restraints/parameters | 11583/0/646 |
| Goodness-of-fit on $F^2$ | 1.043 |
| Final R indexes [I>=2σ (I)] | $R_1$ = 0.0698, $wR_2$ = 0.1777 |
| Final R indexes [all data] | $R_1$ = 0.1150, $wR_2$ = 0.2133 |
| Largest diff. peak/hole / e Å$^{-3}$ | 1.43/-0.57 |
| CCDC number | 2503070 |

**Table S3.** Crystal data and structure refinement for **1b**.

| | |
|---|---|
| Empirical formula | $C_{47}H_{78}KN_3O_6Si$ |
| Formula weight | 848.31 |
| Temperature/K | 150.15 |
| Crystal system | Monoclinic |
| Space group | $P2_1/c$ |
| a/Å | 14.09960(10) |
| b/Å | 16.92000(10) |
| c/Å | 21.6807(2) |
| α/° | 90 |
| β/° | 90.5960(10) |
| γ/° | 90 |
| Volume/Å$^3$ | 5171.98(7) |
| Z | 4 |
| $\rho_{calc}$g/cm$^3$ | 1.089 |
| μ/mm$^{-1}$ | 1.469 |
| F(000) | 1848.0 |
| Crystal size/mm$^3$ | 0.04 × 0.03 × 0.02 |
| Radiation | CuKα (λ = 1.54184) |
| 2Θ range for data collection/° | 6.268 to 145.29 |
| Index ranges | $-17 \leq h \leq 17$, $-14 \leq k \leq 20$, $-26 \leq l \leq 26$ |
| Reflections collected | 38199 |
| Independent reflections | 10147 [$R_{int}$ = 0.0274, $R_{sigma}$ = 0.0221] |
| Data/restraints/parameters | 10147/0/534 |
| Goodness-of-fit on $F^2$ | 1.026 |
| Final R indexes [I>=2σ (I)] | $R_1$ = 0.0342, $wR_2$ = 0.0890 |
| Final R indexes [all data] | $R_1$ = 0.0411, $wR_2$ = 0.0943 |
| Largest diff. peak/hole / e Å$^{-3}$ | 0.33/-0.24 |
| CCDC number | 2503071 |

**Table S4**. Crystal data and structure refinement for **2**.

| | |
|---|---|
| Empirical formula | $C_{58}H_{84}GeN_2Si_2$ |
| Formula weight | 938.04 |
| Temperature/K | 150.15 |
| Crystal system | Monoclinic |
| Space group | *I*2/*a* |
| a/Å | 15.5087(2) |
| b/Å | 18.4471(2) |
| c/Å | 19.8207(3) |
| α/° | 90 |
| β/° | 108.725(2) |
| γ/° | 90 |
| Volume/Å$^3$ | 5370.38(14) |
| Z | 4 |
| $\rho_{calc}$g/cm$^3$ | 1.160 |
| μ/mm$^{-1}$ | 1.471 |
| F(000) | 2024.0 |
| Crystal size/mm$^3$ | 0.04 × 0.03 × 0.02 |
| Radiation | CuKα (λ = 1.54184) |
| 2Θ range for data collection/° | 6.718 to 145.09 |
| Index ranges | -19 ≤ h ≤ 18, -22 ≤ k ≤ 21, -23 ≤ l ≤ 24 |
| Reflections collected | 20034 |
| Independent reflections | 5288 [$R_{int}$ = 0.0290, $R_{sigma}$ = 0.0222] |
| Data/restraints/parameters | 5288/0/296 |
| Goodness-of-fit on F$^2$ | 1.029 |
| Final R indexes [I>=2σ (I)] | $R_1$ = 0.0290, w$R_2$ = 0.0778 |
| Final R indexes [all data] | $R_1$ = 0.0323, w$R_2$ = 0.0805 |
| Largest diff. peak/hole / e Å$^{-3}$ | 0.28/-0.23 |
| CCDC number | 2503073 |

**Table S5**. Crystal data and structure refinement for **3**.

| | |
|---|---|
| Empirical formula | $C_{61}H_{93}N_2O_{0.5}Si_3Sn$ |
| Formula weight | 1065.33 |
| Temperature/K | 150.15 |
| Crystal system | Triclinic |
| Space group | *P*-1 |
| a/Å | 9.9572(3) |
| b/Å | 17.4221(6) |
| c/Å | 19.7370(6) |
| α/° | 113.668(3) |
| β/° | 93.288(2) |
| γ/° | 104.006(3) |
| Volume/Å$^3$ | 2996.31(18) |
| Z | 2 |
| $\rho_{calc}$g/cm$^3$ | 1.181 |
| μ/mm$^{-1}$ | 4.242 |
| F(000) | 1138.0 |
| Crystal size/mm$^3$ | 0.32 × 0.13 × 0.11 |
| Radiation | CuKα (λ = 1.54184) |
| 2Θ range for data collection/° | 4.964 to 145.262 |
| Index ranges | $-10 \leq h \leq 12$, $-21 \leq k \leq 15$, $-23 \leq l \leq 24$ |
| Reflections collected | 22313 |
| Independent reflections | 11522 [$R_{int}$ = 0.0622, $R_{sigma}$ = 0.0626] |
| Data/restraints/parameters | 11522/51/638 |
| Goodness-of-fit on $F^2$ | 1.025 |
| Final R indexes [I>=2σ (I)] | $R_1$ = 0.0468, $wR_2$ = 0.1200 |
| Final R indexes [all data] | $R_1$ = 0.0540, $wR_2$ = 0.1276 |
| Largest diff. peak/hole / e Å$^{-3}$ | 1.33/-1.70 |
| CCDC number | 2503074 |

**Table S6**. Crystal data and structure refinement for **4**.

| | |
|---|---|
| Empirical formula | $C_{61}H_{93}N_2O_{0.5}PbSi_3$ |
| Formula weight | 1153.83 |
| Temperature/K | 150.15 |
| Crystal system | Triclinic |
| Space group | *P*-1 |
| a/Å | 9.9506(2) |
| b/Å | 17.5013(5) |
| c/Å | 19.7101(4) |
| α/° | 113.785(2) |
| β/° | 93.317(2) |
| γ/° | 104.063(2) |
| Volume/$Å^3$ | 2999.47(13) |
| Z | 2 |
| $\rho_{calc}g/cm^3$ | 1.278 |
| $\mu/mm^{-1}$ | 6.318 |
| F(000) | 1202.0 |
| Crystal size/$mm^3$ | 0.1 × 0.06 × 0.04 |
| Radiation | CuKα (λ = 1.54184) |
| 2Θ range for data collection/° | 4.976 to 136.49 |
| Index ranges | $-10 \leq h \leq 11$, $-20 \leq k \leq 21$, $-23 \leq l \leq 21$ |
| Reflections collected | 21161 |
| Independent reflections | 10942 [$R_{int}$ = 0.0646, $R_{sigma}$ = 0.0538] |
| Data/restraints/parameters | 10942/0/632 |
| Goodness-of-fit on $F^2$ | 1.101 |
| Final R indexes [I>=2σ (I)] | $R_1$ = 0.0437, $wR_2$ = 0.1280 |
| Final R indexes [all data] | $R_1$ = 0.0464, $wR_2$ = 0.1324 |
| Largest diff. peak/hole / e $Å^{-3}$ | 1.77/-4.09 |
| CCDC number | 2503075 |

**Table S7**. Crystal data and structure refinement for **2**$^{\bullet-}$[K([2.2.2]-cryptand)].

| | |
|---|---|
| Empirical formula | $C_{80}H_{130}GeKN_4O_7Si_2$ |
| Formula weight | 1427.74 |
| Temperature/K | 150.15 |
| Crystal system | monoclinic |
| Space group | $P2_1/n$ |
| a/Å | 14.8256(3) |
| b/Å | 28.7239(8) |
| c/Å | 19.7485(4) |
| α/° | 90 |
| β/° | 101.244(2) |
| γ/° | 90 |
| Volume/Å$^3$ | 8248.5(3) |
| Z | 4 |
| $\rho_{calc}$g/cm$^3$ | 1.150 |
| μ/mm$^{-1}$ | 1.631 |
| F(000) | 3092.0 |
| Crystal size/mm$^3$ | 0.21 × 0.1 × 0.03 |
| Radiation | CuKα (λ = 1.54184) |
| 2Θ range for data collection/° | 5.504 to 145.49 |
| Index ranges | $-18 \leq h \leq 15$, $-35 \leq k \leq 34$, $-22 \leq l \leq 24$ |
| Reflections collected | 63004 |
| Independent reflections | 16163 [$R_{int}$ = 0.1284, $R_{sigma}$ = 0.1082] |
| Data/restraints/parameters | 16163/194/889 |
| Goodness-of-fit on $F^2$ | 0.998 |
| Final R indexes [I>=2σ (I)] | $R_1$ = 0.0631, $wR_2$ = 0.1415 |
| Final R indexes [all data] | $R_1$ = 0.1132, $wR_2$ = 0.1687 |
| Largest diff. peak/hole / e Å$^{-3}$ | 0.65/-0.49 |
| CCDC number | 2535034 |

**Table S8**. Crystal data and structure refinement for **3**$^{\bullet-}$[K([2.2.2]-cryptand)].

| | |
|---|---|
| Empirical formula | $C_{77}H_{122}KN_4O_{6.25}Si_2Sn$ |
| Formula weight | 1417.75 |
| Temperature/K | 150.15 |
| Crystal system | monoclinic |
| Space group | $P2_1/n$ |
| a/Å | 14.66720(10) |
| b/Å | 29.0232(2) |
| c/Å | 19.75710(10) |
| α/° | 90 |
| β/° | 100.4710(10) |
| γ/° | 90 |
| Volume/Å$^3$ | 8270.32(9) |
| Z | 4 |
| $\rho_{calc}$g/cm$^3$ | 1.139 |
| μ/mm$^{-1}$ | 3.557 |
| F(000) | 3036.0 |
| Crystal size/mm$^3$ | 0.025 × 0.02 × 0.01 |
| Radiation | CuKα (λ = 1.54184) |
| 2Θ range for data collection/° | 5.474 to 145.256 |
| Index ranges | $-17 \leq h \leq 18$, $-33 \leq k \leq 35$, $-23 \leq l \leq 24$ |
| Reflections collected | 61465 |
| Independent reflections | 16244 [$R_{int}$ = 0.0293, $R_{sigma}$ = 0.0240] |
| Data/restraints/parameters | 16244/58/865 |
| Goodness-of-fit on $F^2$ | 1.024 |
| Final R indexes [I>=2σ (I)] | $R_1$ = 0.0319, $wR_2$ = 0.0824 |
| Final R indexes [all data] | $R_1$ = 0.0358, $wR_2$ = 0.0857 |
| Largest diff. peak/hole / e Å$^{-3}$ | 1.09/-0.77 |
| CCDC number | 2535035 |

## A6 UV-vis-NIR Spectra

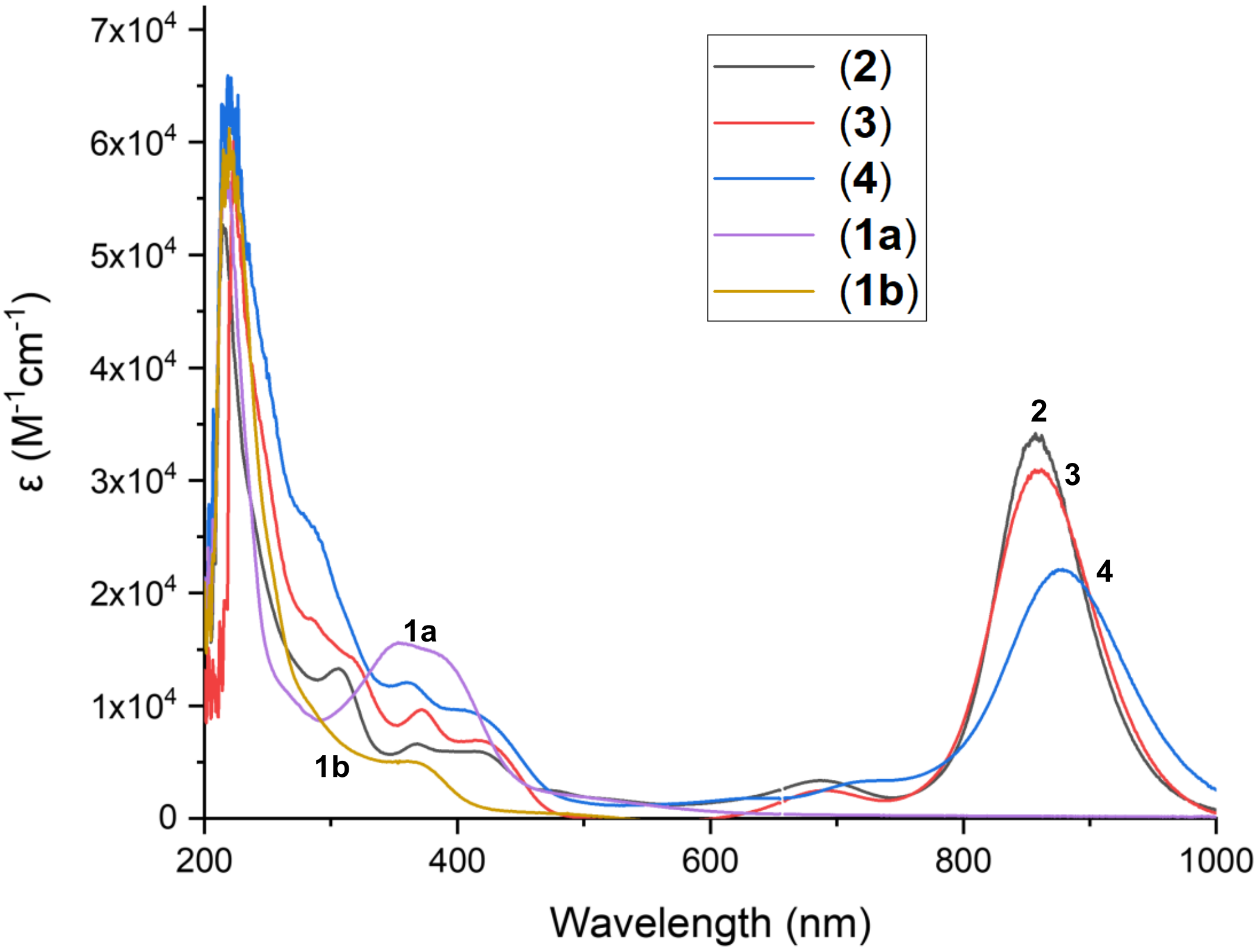


**Figure S33**. UV-vis spectra of **1-4** in n-hexane (5.0 × 10⁻⁵ M, 293 K)

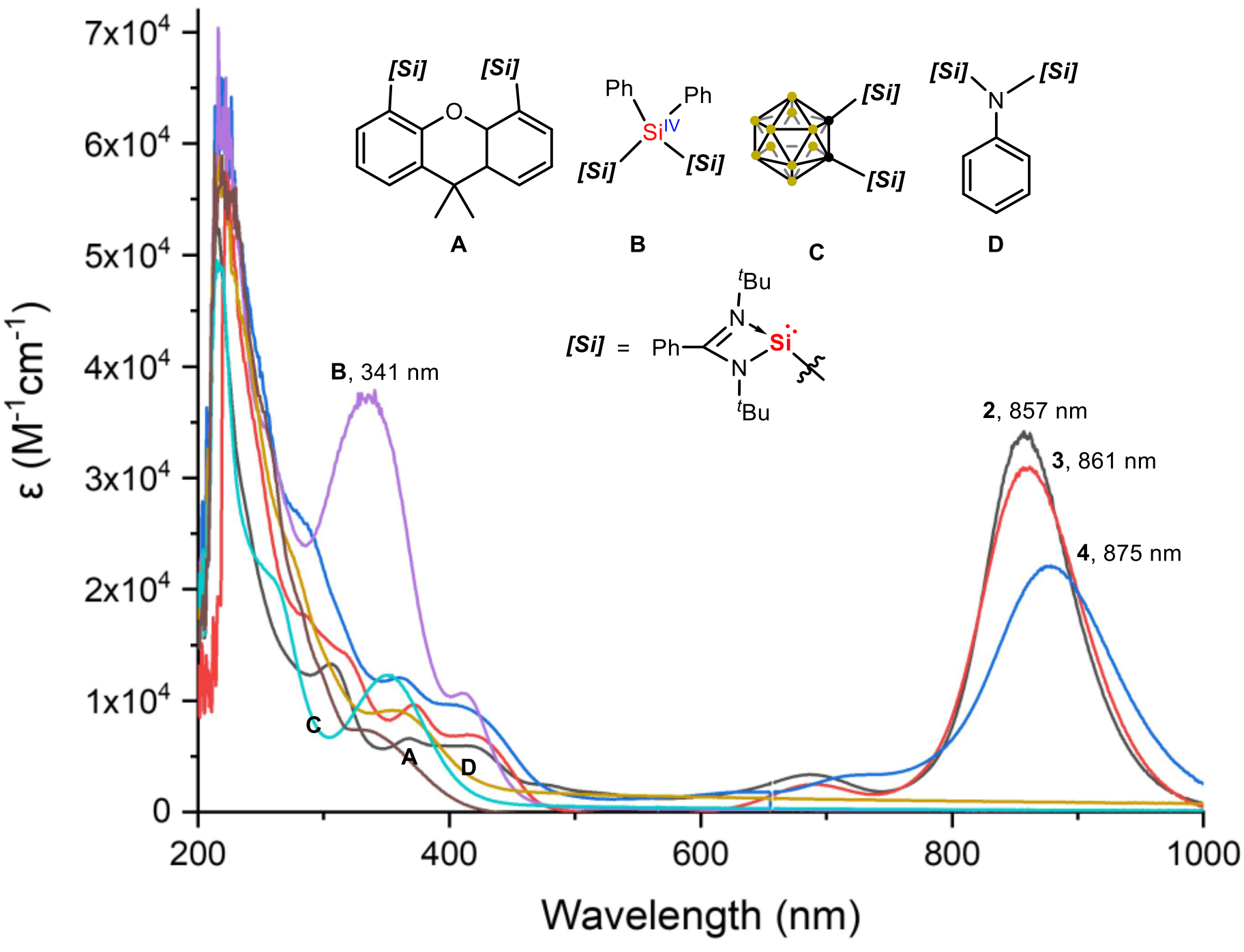


**Figure S34**. UV-vis spectra of **A-D** and **2-4** in n-hexane (5.0 × 10⁻⁵ M, 293 K)

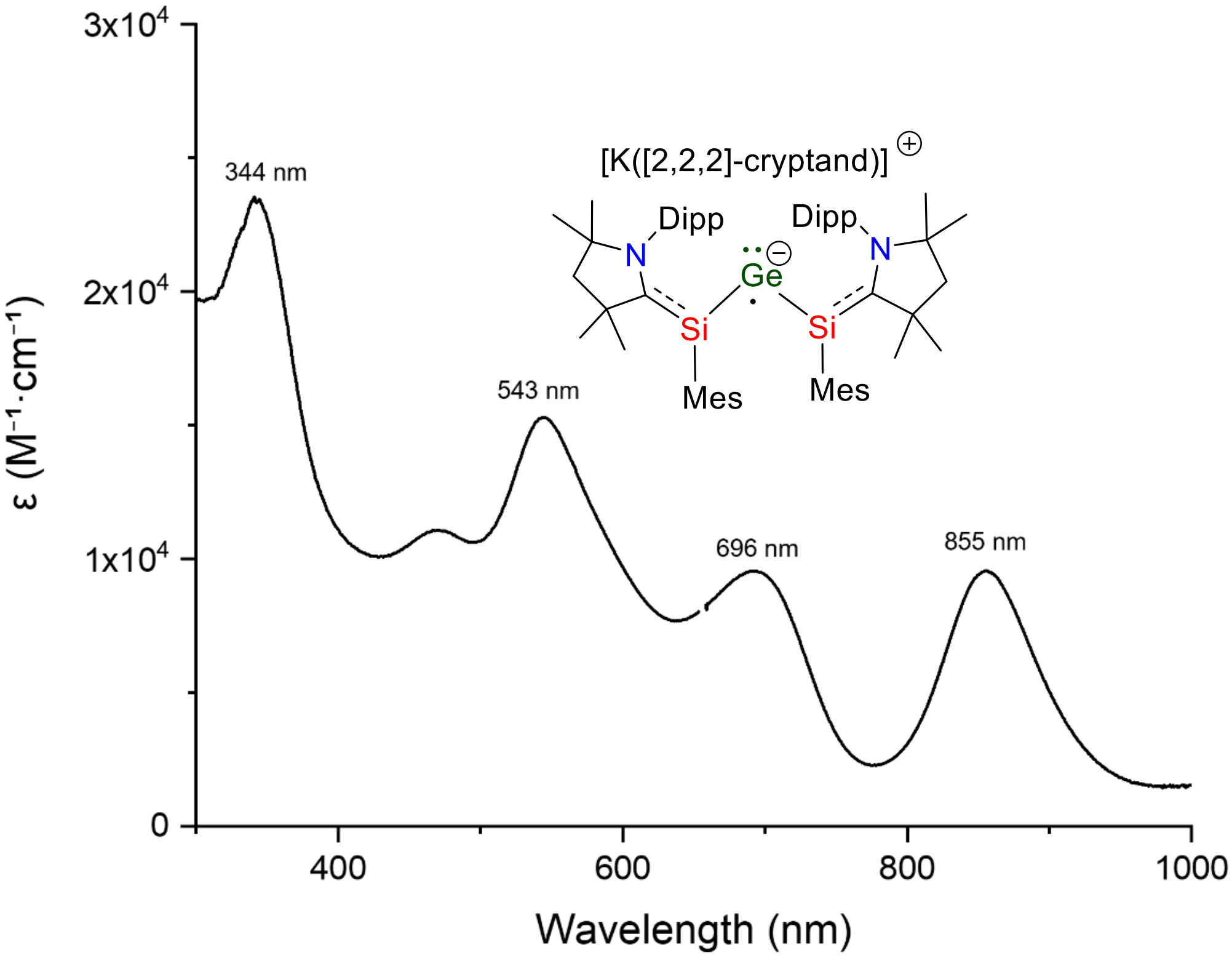


**Figure S35**. UV-vis spectra of **2**$^{\bullet-}$[K([2.2.2]-cryptand)] in THF ($5.0 \times 10^{-5}$ M, 293 K)

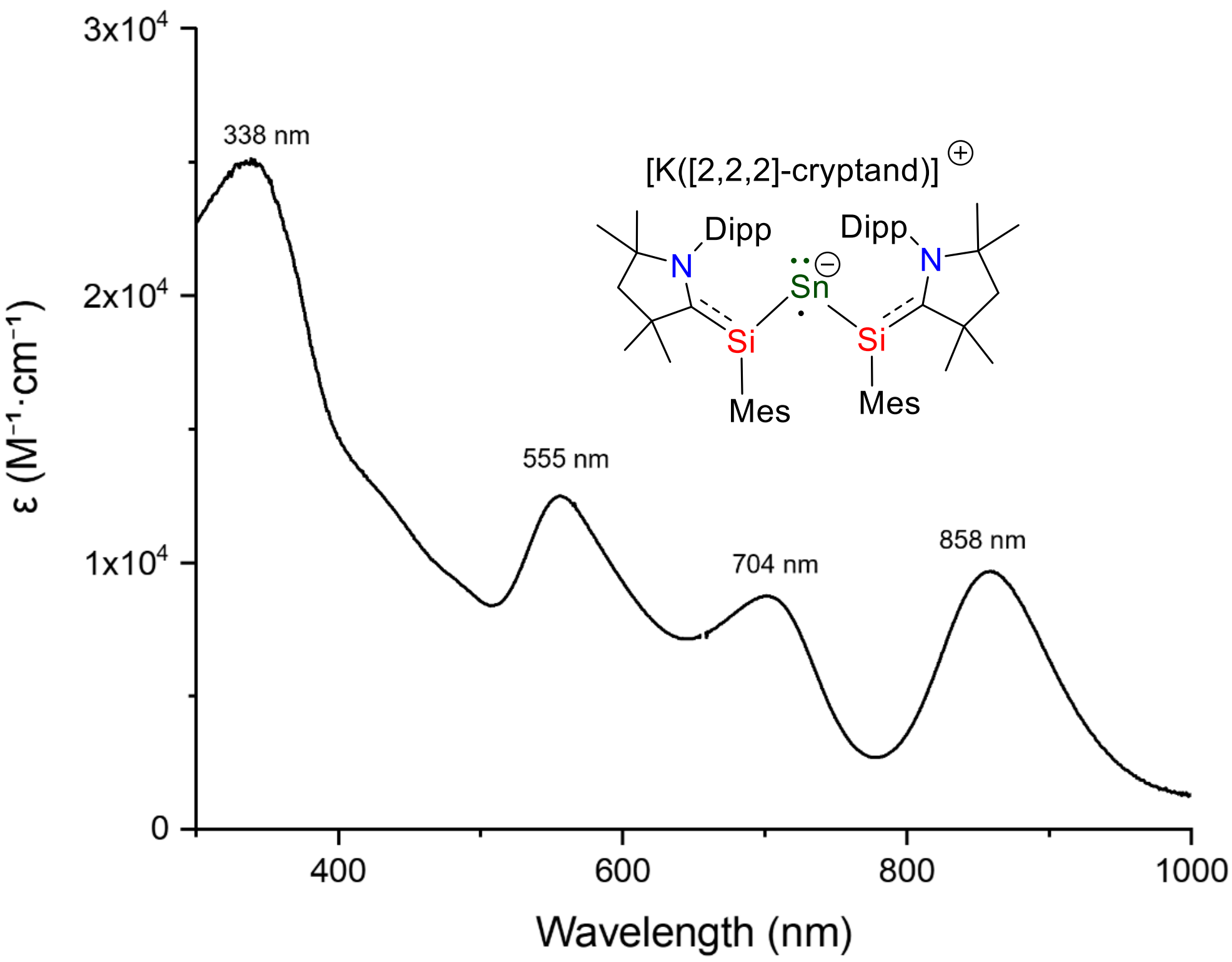


**Figure S36**. UV-vis spectra of **3**$^{\bullet-}$[K([2.2.2]-cryptand)] in THF ($5.0 \times 10^{-5}$ M, 293 K)

## A7 Electrochemistry

Cyclic voltammetry measurements were conducted at 295 K by using a Biologic SP-150 potentiostat and a three-electrode set-up inside a glovebox in a one-compartment cylindrical cell with glassy carbon as a working electrode, platinum wire as a counter-electrode and silver wire as a pseudo-reference electrode. All potentials were referenced to the external $Fc/Fc^+$ (ferrocene/ferrocenium) couple, which was measured under identical conditions before and after each experiment. Measurements were conducted in THF containing 0.1 M [*n*(Octyl)$_4$N][$BF_4$] as supporting electrolyte at room temperature under an inert atmosphere. The scan rate was 100 mV $s^{-1}$ unless otherwise specified. Compounds **2** and **3** exhibit reversible redox waves at $E_{1/2}$ = −2.51 V and −2.52 V vs. $Fc/Fc^+$, respectively (Figures S29–S30).

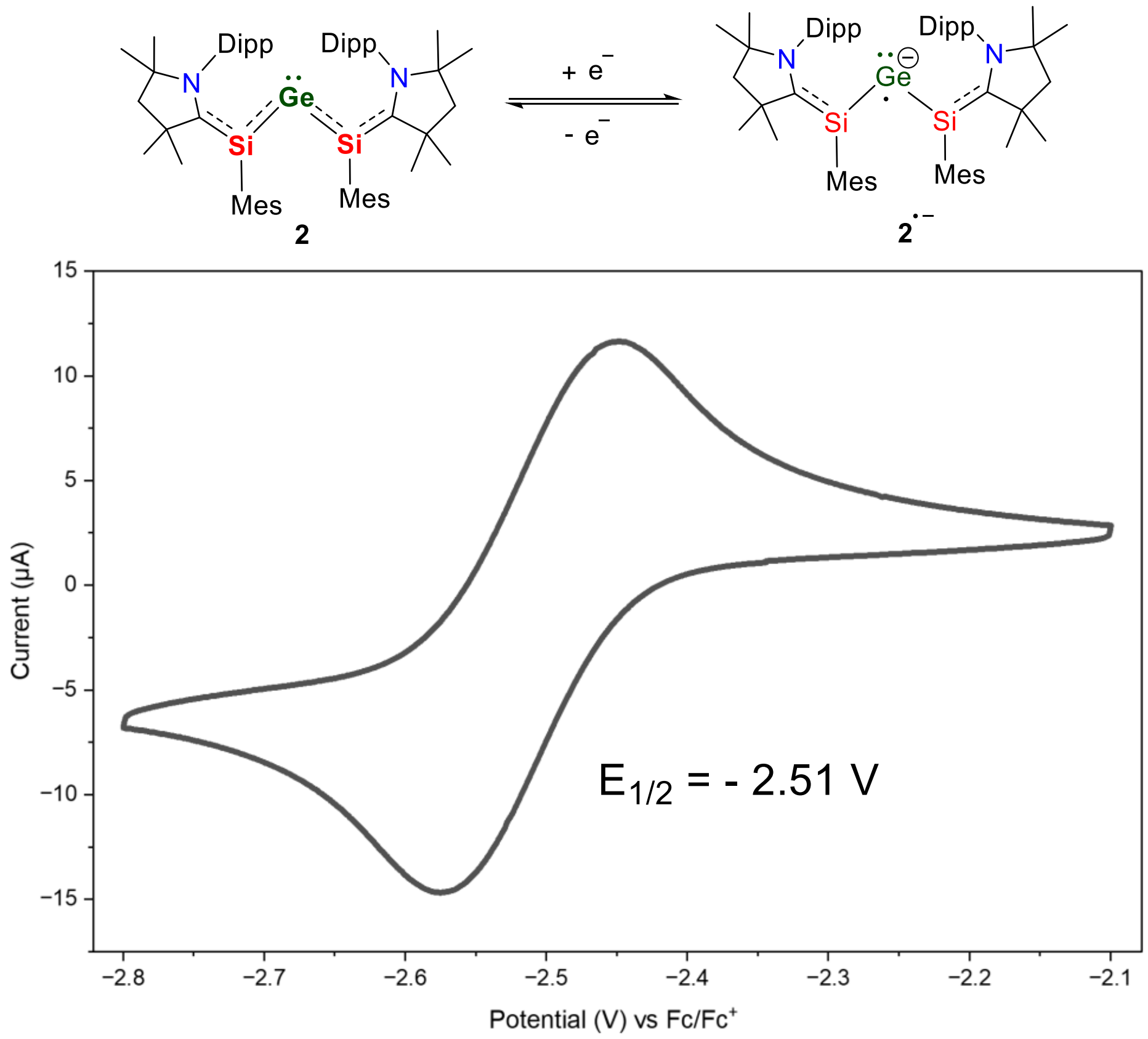


**Figure S37**. Cyclic voltammogram of **2** in THF / 0.1 M [*n*(Octyl)$_4$N][$BF_4$]

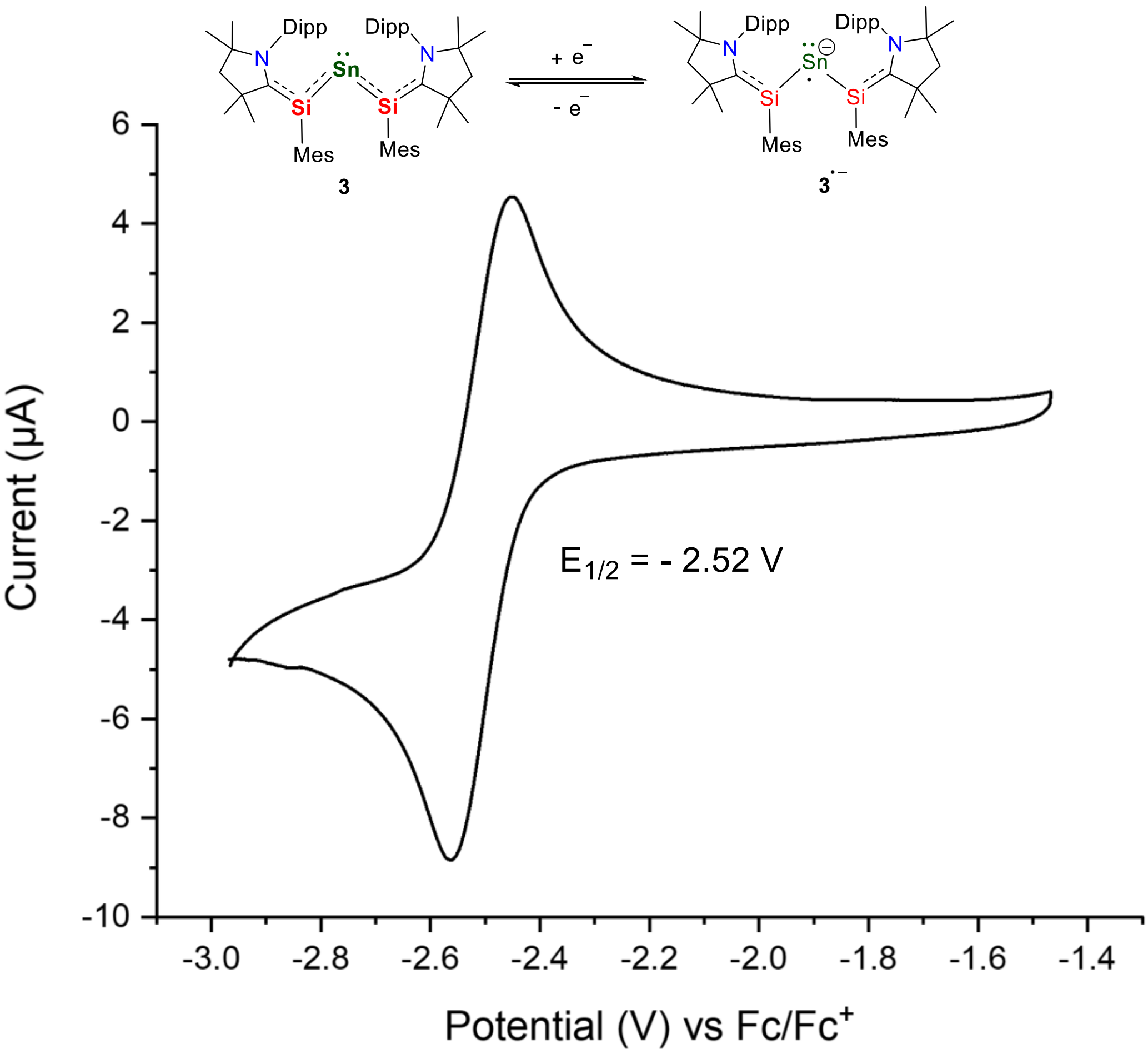


**Figure S38**. Cyclic voltammogram of **3** in THF / 0.1 M [*n*(Octyl)$_4$N][$BF_4$]

## A8 Electron Paramagnetic Resonance (EPR) Spectroscopy

### A8.1 EPR Spectrum of **2**$^{\bullet-}$[K([2.2.2]-cryptand)]

The X-band CW EPR spectrum of **2**$^{\bullet-}$[K([2.2.2]-cryptand)] recorded in THF at 298 K displays an intense, sharp isotropic signal at $g_{iso}$ = 2.016, slightly higher than the free-electron value ($g_e$ = 2.0023), consistent with significant spin localization at the heavier Ge center and associated spin–orbit coupling effects. The spectrum can be satisfactorily simulated by inclusion of hyperfine coupling to $^{29}$Si nuclei (I = 1/2), with a($^{29}$Si) = 18.1 G. The expected satellite features arising from coupling to $^{73}$Ge (I = 9/2, 7.8% natural abundance) are not resolved, most likely due to overlap with the intense central signal in combination with the low natural abundance and small magnetic moment of the $^{73}$Ge nucleus.

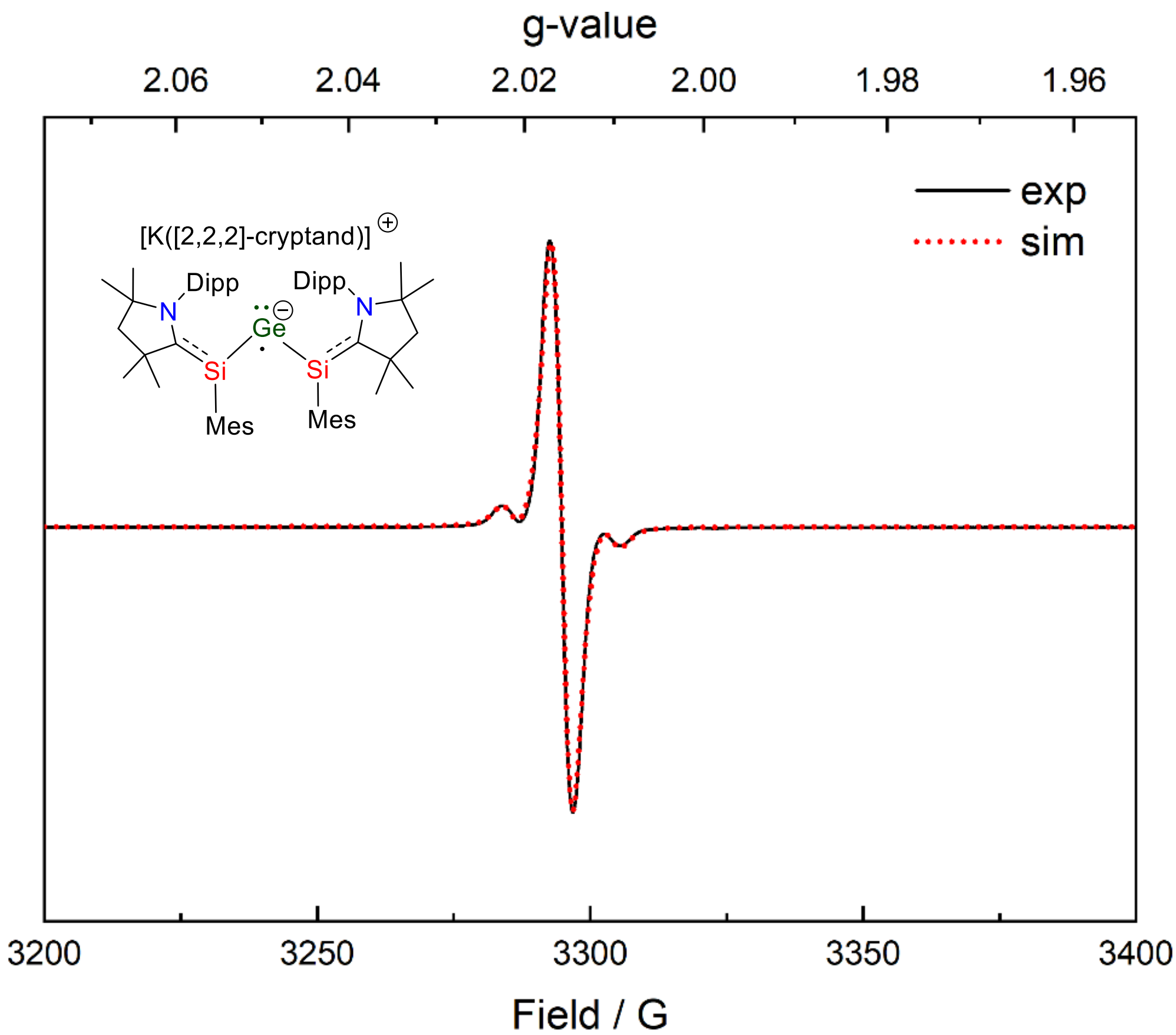


**Figure S39**. EPR spectra of **2**$^{\bullet-}$[K([2.2.2]-cryptand)] in THF measured at 298 K. The EPR spectrum of crystals of **2**$^{\bullet-}$[K([2.2.2]-cryptand)] clearly shows an intense and very sharp isotropic signal at $g_{iso}$ = 2.016.

A8.2 EPR Spectra of **3**$^{•−}$[K([2.2.2]-cryptand)]

Due to the accelerated spin relaxation characteristic of heavy-element-centered radicals, the EPR spectrum of **3**$^{•−}$[K([2.2.2]-cryptand)] was recorded in frozen THF solution at 10 K. The dominant spectral component is well resolved and characterized by a rhombic g tensor with principal values $g_x$ = 2.08, $g_y$ = 2.02, $g_z$ = 1.99, $g_{av}$ = 2.03, together with pronounced anisotropic hyperfine coupling to $^{117/119}$Sn nuclei (I = 1/2; natural abundances 7.7% and 8.6%), with coupling constants $A_x$ = 561 MHz, $A_y$ = 623 MHz, and $A_z$ = 988 MHz. Spectral simulations were performed using EasySpin.

In addition to the dominant component, minor spectral features are consistently observed, which can be simulated by a second component (species 2) with similar g values. The relative intensity of this minor component remains unchanged across independently prepared samples and after different workup procedures, indicating that it does not arise from a distinct isolable species. Furthermore, no additional features emerge upon variation of temperature, excluding dynamic exchange processes. These observations suggest that the minor component most likely originates from partial decomposition during sample handling, giving rise to a closely related Sn radical species with unresolved hyperfine structure.

Importantly, the dominant spectral component is highly reproducible and well resolved. The microwave power saturation behavior further supports assignment of the dominant species as a heavy-element-centered radical with fast relaxation properties. Taken together, these EPR data unambiguously support the assignment of **3**$^{•−}$[K([2.2.2]-cryptand)] as Sn-centered radical anions.

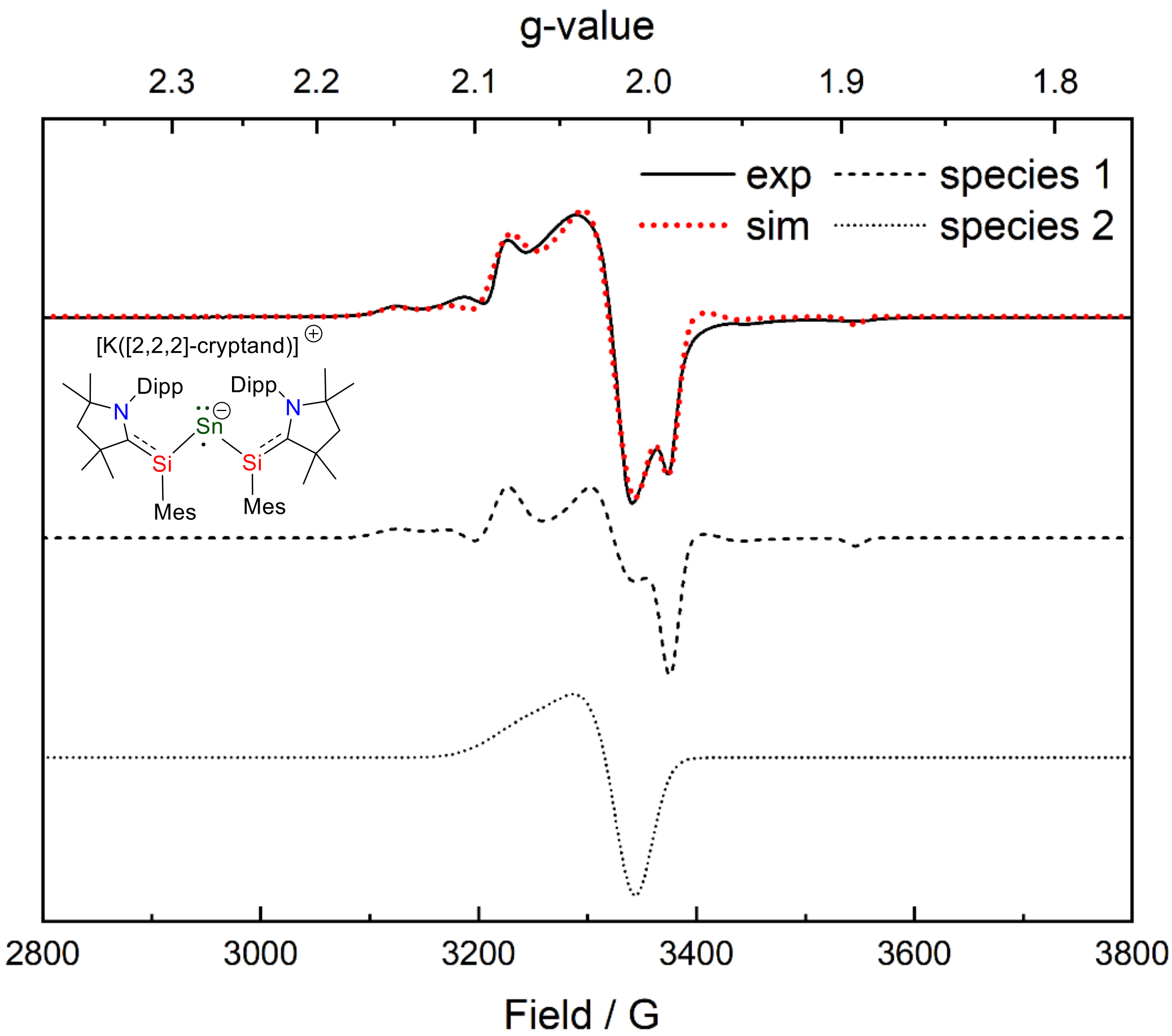


**Figure S40.** X-band EPR spectrum of **3**$^{\bullet-}$[K([2.2.2]-cryptand)] in THF recorded at 10 K. The experimental and simulated spectra are shown as solid black and dashed red traces, respectively. The simulation includes two components: species 1 with g = (2.08, 2.02, 1.99) and species 2 with g = (2.07, 2.03, 2.01), shown as dashed and dotted lines, respectively.

Note: Based on the similar $g_{iso}$ of species 1 and species 2 ($g_{av}$ = 2.03 and $g_{av}$ = 2.04 as well as similar linewidth and temperature dependence the second species is likely also a Sn centered radical for which the hyperfine coupling was not resolved. Species 2 likely arises from partial decomposition of **3**$^{\bullet-}$[K([2.2.2]-cryptand)] during handling.

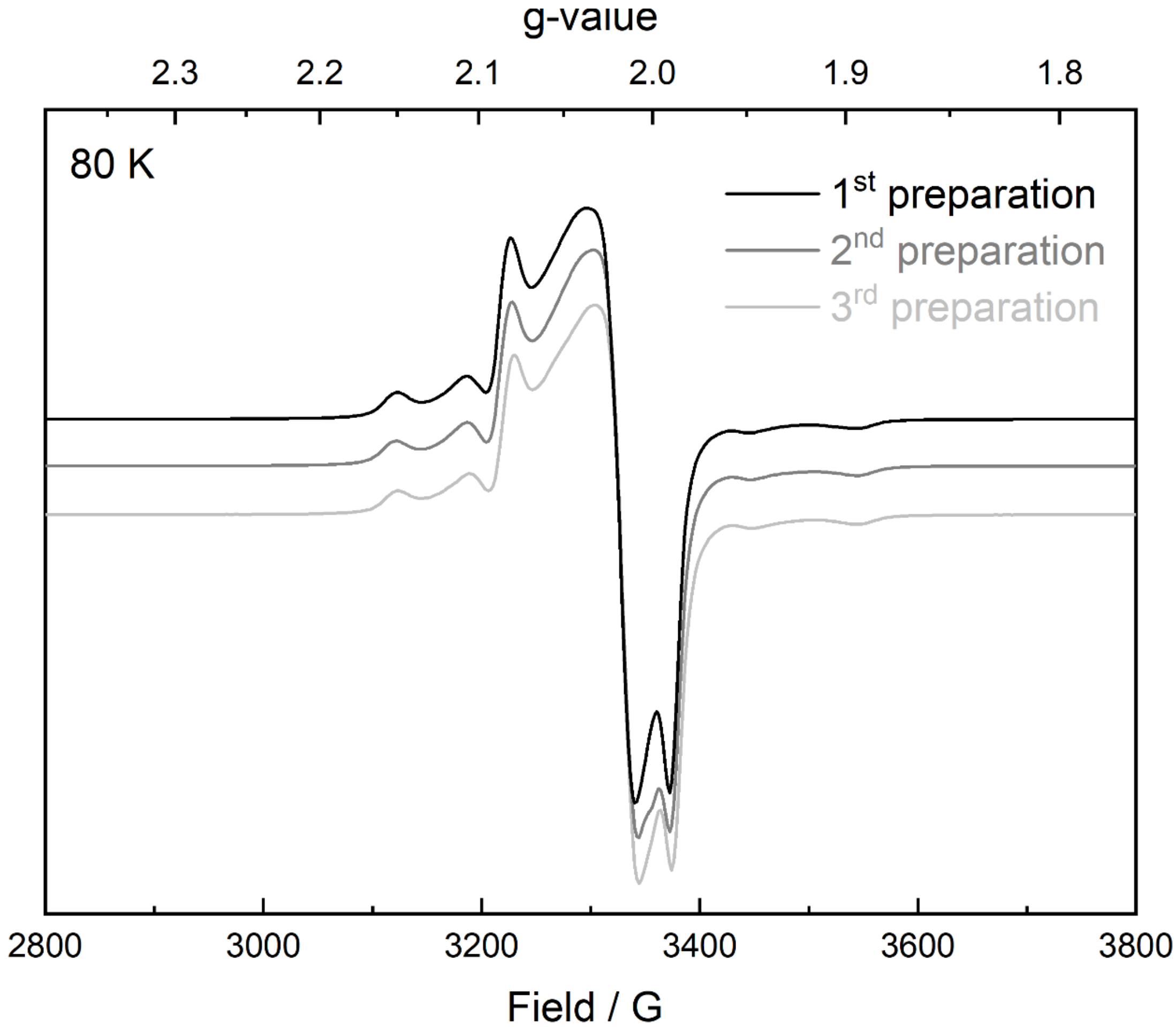


**Figure S41**. Low-temperature (80 K) X-band EPR spectra of three independently prepared samples of **3**$^{•-}$[K([2.2.2]-cryptand)] in THF, corresponding to the reaction solution (1$^{st}$), the filtered sample (2$^{nd}$), and the recrystallized sample (3$^{rd}$). The spectra display highly reproducible features, including consistent minor contributions from an additional paramagnetic species.

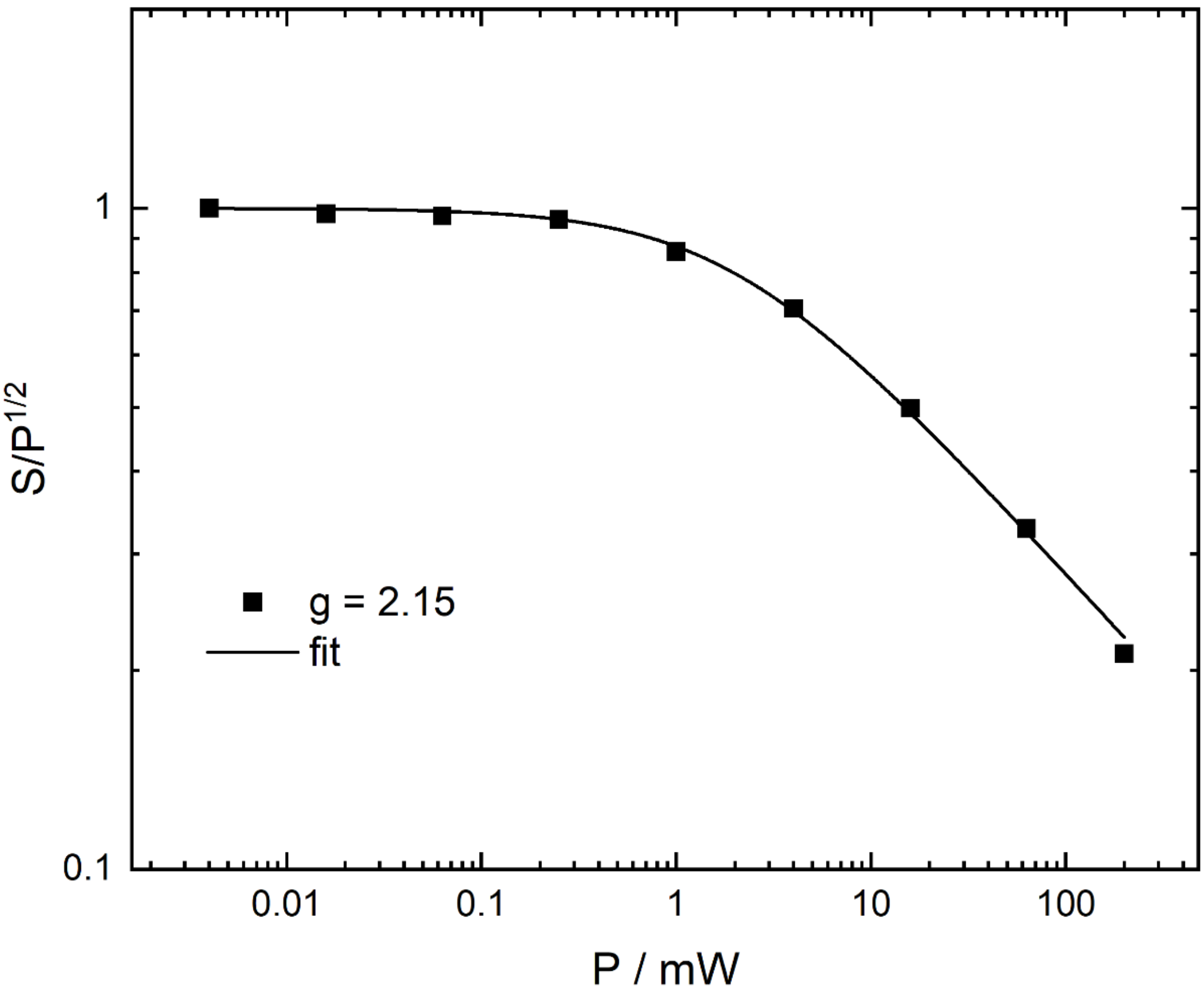


**Figure S42**. Microwave power saturation behavior measured at g ≈ 2.15 and 10 K. For compound **3**$^{\bullet-}$[K([2.2.2]-cryptand)], the characteristic power saturation of a heavy metal based radical with fast spin relaxation is observed.

Note: These observations support the assignment of the dominant species as a Sn radical, despite the presence of minor additional components.

## *B. Theoretical Calculations*

## B1 Computational Methods

### B1.1 General Method

All calculations relating to the geometry optimizations were performed with ORCA 6.0.0[12] with the B3LYP functional[13] augmented with the D3 version of Grimme's empirical dispersion correction.[14,15] The def2-SVP basis set was used for all the atoms.[16] The RI approximation in combination with the auxiliary basis set SARC/J was used to speed up these calculations.[17] Frequency calculations at the same level of theory were performed. All optimized structures were confirmed as minima by frequency calculations (no imaginary frequencies). Tighter-than-default scf ("tightscf") and optimization criteria ("tightopt") were applied. The gas-phase geometry was used for all the solution phase calculations. Natural bond orbital (NBO) calculations were carried out using NBO 7.0 program[18] at the B3LYP-D3/def2-SVP level of theory, and natural resonance theory (NRT) calculations were carried out using NBO 7.0 program at the same level of theory. These NRT calculations were done for a model of **2** in which Dipp and methyl groups were replaced by H atom, but at the geometry corresponding to **2**. Intrinsic bond orbitals (IBOs) were carried out using ORCA program at the same level (B3LYP-D3/def2-SVP). Optimized structures were visualized by the Chemcraft,[19] IBOview[20,21] or VMD 1.9[22] programs. Energy decomposition analysis (EDA) was carried out using the sobEDA method,[23] as implemented in Gaussian 16 (Rev. C.01),[24] with wavefunction analyses performed using Multiwfn,[25,26] at the B3LYP/6-31+G(d,p) level of theory.

### B1.2 Method for Determination of Percent Buried Volume

The percent buried volume (%$V_{bur}$) quantifies the portion of space occupied by a ligand within the first coordination sphere surrounding a metal center. In this calculation, a sphere of radius *R* is constructed around the metal and discretized into three-dimensional voxels, collectively defining the coordination sphere volume ($V_{sphere}$). For each voxel, the distance between its center and all atoms of the ligand is evaluated. If any ligand atom lies within a distance shorter than its van der Waals radius from the voxel center, that voxel is classified as buried volume ($V_{\beta ur}$). Otherwise, the voxel is designated as free volume ($V_{free}$). The percent buried volume is then calculated as ($V_{\beta ur}/V_{sphere}$) × 100%. All %$V_{bur}$ values reported in this study were computed using SambVca 2.1 based on DFT optimized geometry coordinates (**E**, **F**, **G**, **H**, **3**, **4**).[27–29]

B1.3 Method for Excited-State Studies

Time dependent density functional theory (TD-DFT) calculations were performed with the GGA functional mPWLYP (modified PW exchange and LYP correlation).[30] Scalar relativistic effects were taken into account using the zero-order regular approximation (ZORA),[31–34] combined with ZORA-recontracted versions[35] of the def2 basis sets. Triple-zeta basis sets def2-TZVPP were used for the ligands and the SARC-ZORA-TZVP basis set for Sn, Pb atoms.[35] The resolution of identity approximation and the chain-of-spheres approximation to exact exchange[36] (RIJCOSX in ORCA convention) were employed to reduce computational time along with the SARC/J auxiliary basis set. Tight convergence criteria were chosen (tightscf). Thirty spin-allowed transitions were calculated by TDDFT. Spin Orbit Coupling (SOC) TDDFT calculations were performed using the additional keyword RI-SOMF(1X).[37] The vertical spin-forbidden transitions were localized with triplets true as keyword. To account for solvent effects, a SMD model modelling hexane was used in all calculations.[38,39] The hole-electron distribution analysis was carried out using Multiwfn. The S1 excited state was optimized using the first excited singlet state with root=1 while the T1 state was optimized with multiplicity = 3 as implemented in ORCA.

## B2 Calculated results

B2.1 The Silanylidene Anion

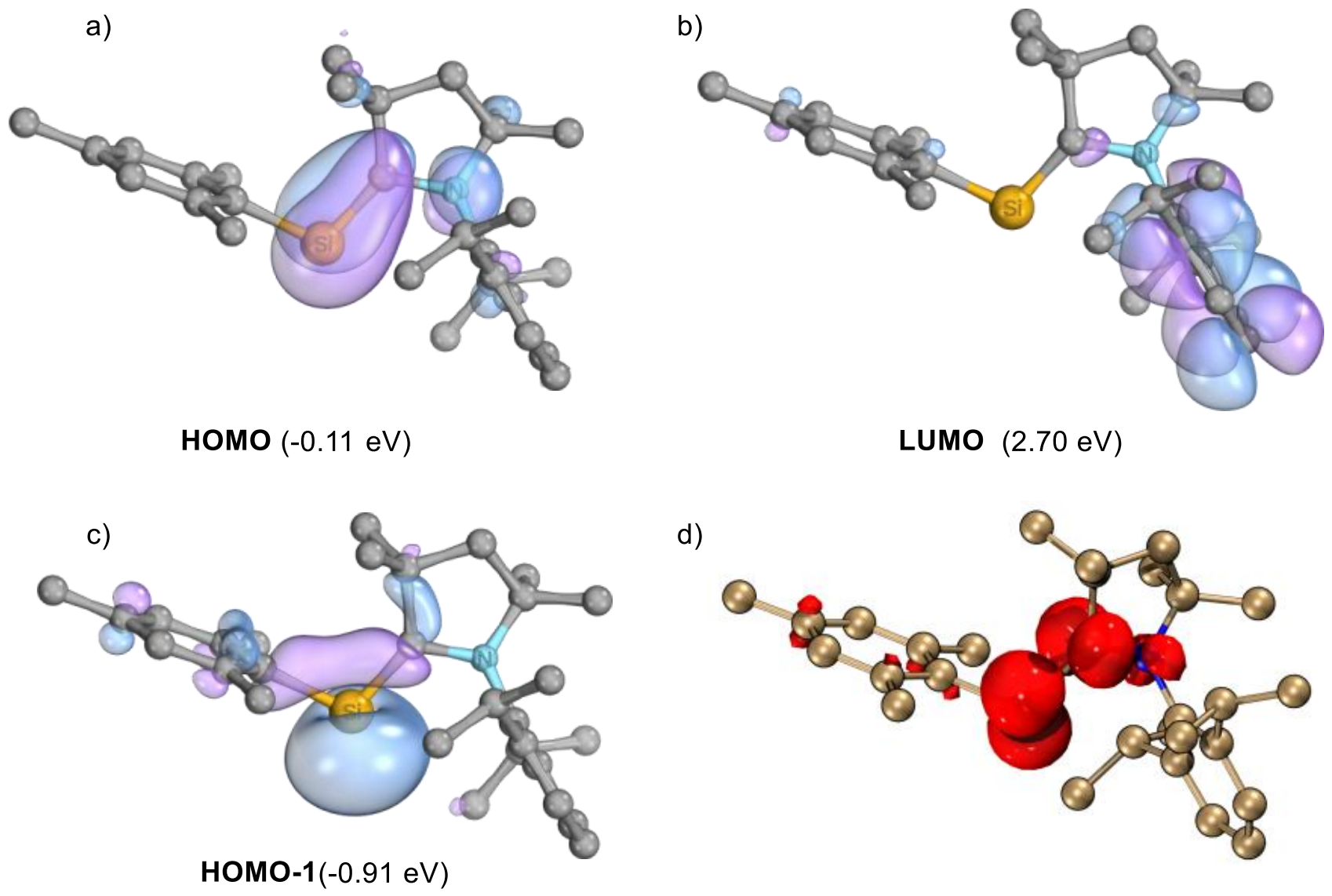


**Figure S43**. a-c) Frontier Molecular Orbitals (FMOs) of singlet silanylidene anion. d) Electron spin density of triplet silanylidene anion ($\Delta E_{S/T}$ = -28.6 kcal mol$^{-1}$).

B2.2 Percent Buried Volume Data

**Table S9**. %$V_{bur}$ data of **E – H**, **3**, and **4**.

| Compound | %$V_{bur}$ |
|---|---|
| **E** | 77.1 |
| **F** | 88.1 |
| **G** | 90.4 |
| **H** | 73.8 |
| **3** | 96.2 |
| **4** | 94.3 |

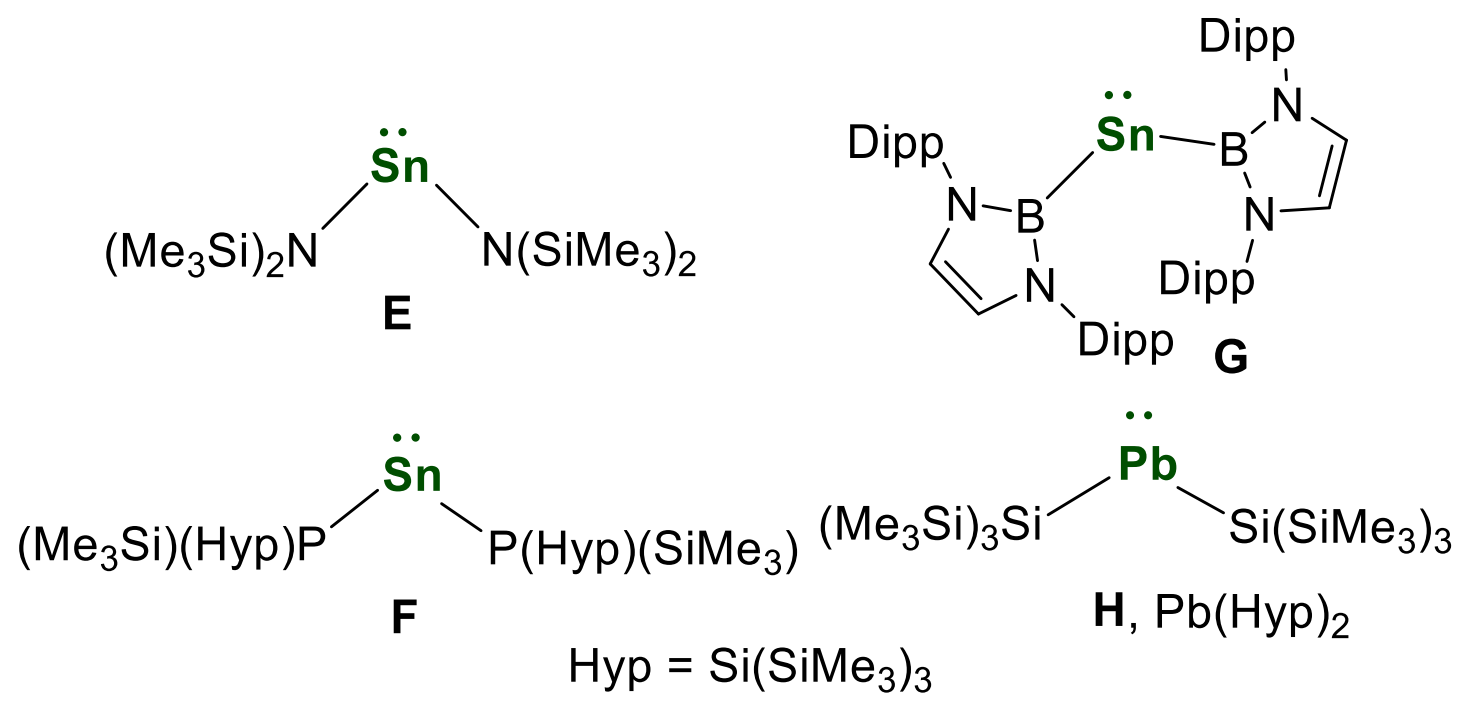


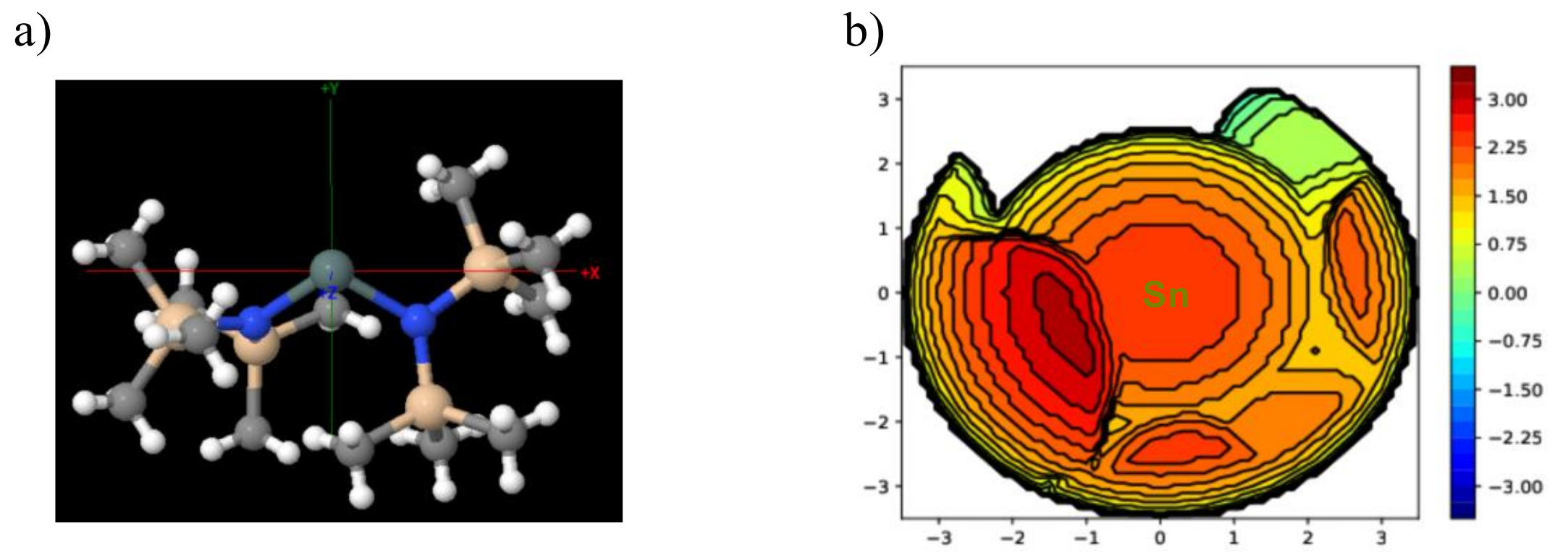


**Figure S44**. a) Orientation of **E** along the z-axis. b) Topographic steric map calculated for **E**, viewed along the z-axis . Steric map axes refer to the x- and y-axes. Brown: tin, blue: nitrogen, grey: carbon, white: hydrogen. %$V_{bur}$ = 77.1%

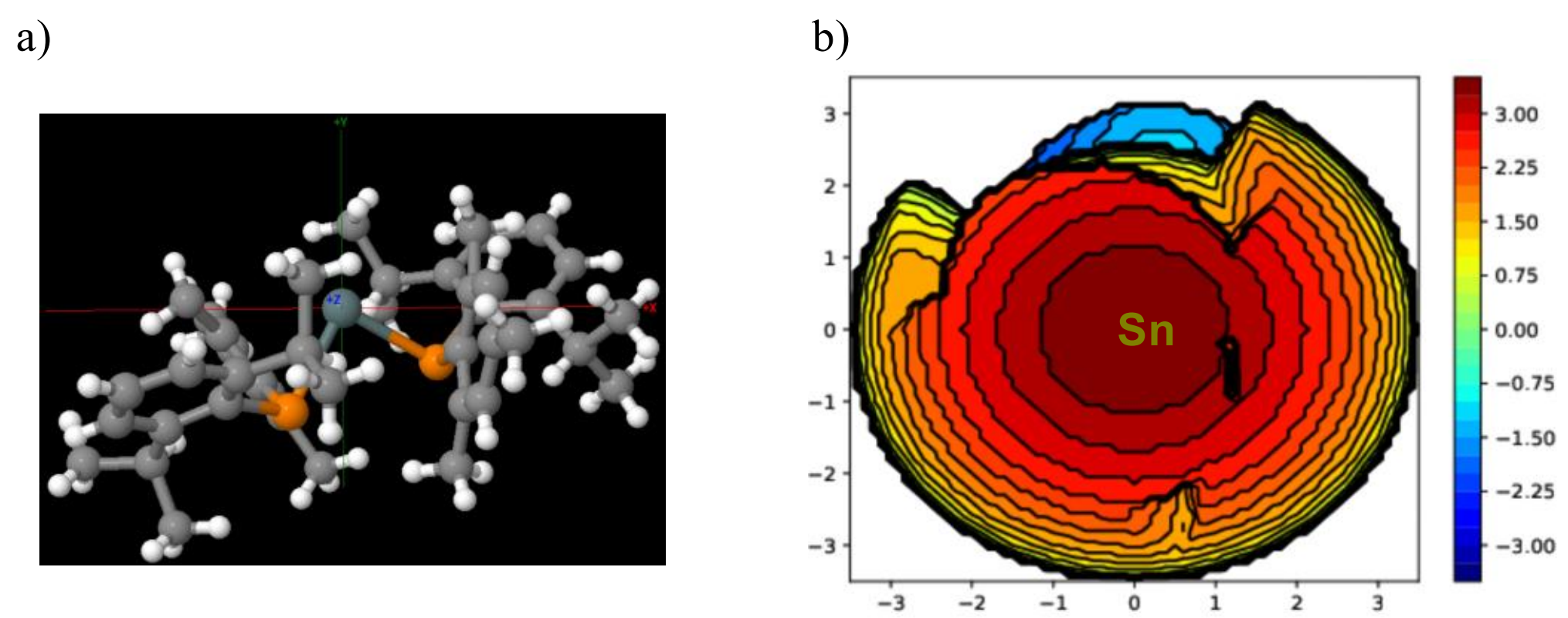


**Figure S45**. a) Orientation of **F** along the z-axis. b) Topographic steric map calculated for **F**, viewed along the z-axis. Steric map axes refer to the x- and y-axes. Brown: tin, organe: phopshorus, grey: carbon, white: hydrogen. %$V_{bur}$ = 88.1%

a) b)

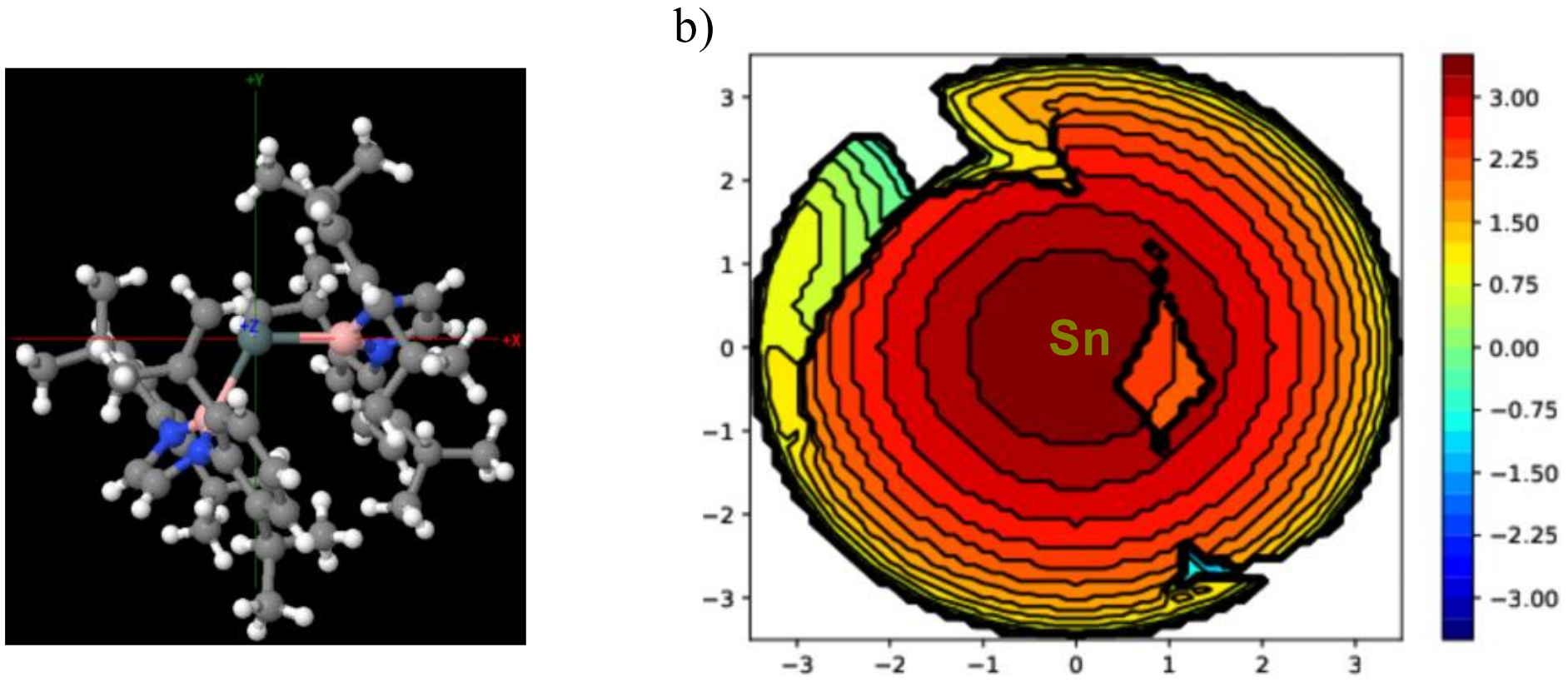


**Figure S46**. a) Orientation of **G** along the z-axis. b) Topographic steric map calculated for **G**, viewed along the z-axis. Steric map axes refer to the x- and y-axes. Brown: tin, pink: boron, blue: nitrogen, grey: carbon, white: hydrogen. $\%V_{bur}$ = 90.4%

a) b)

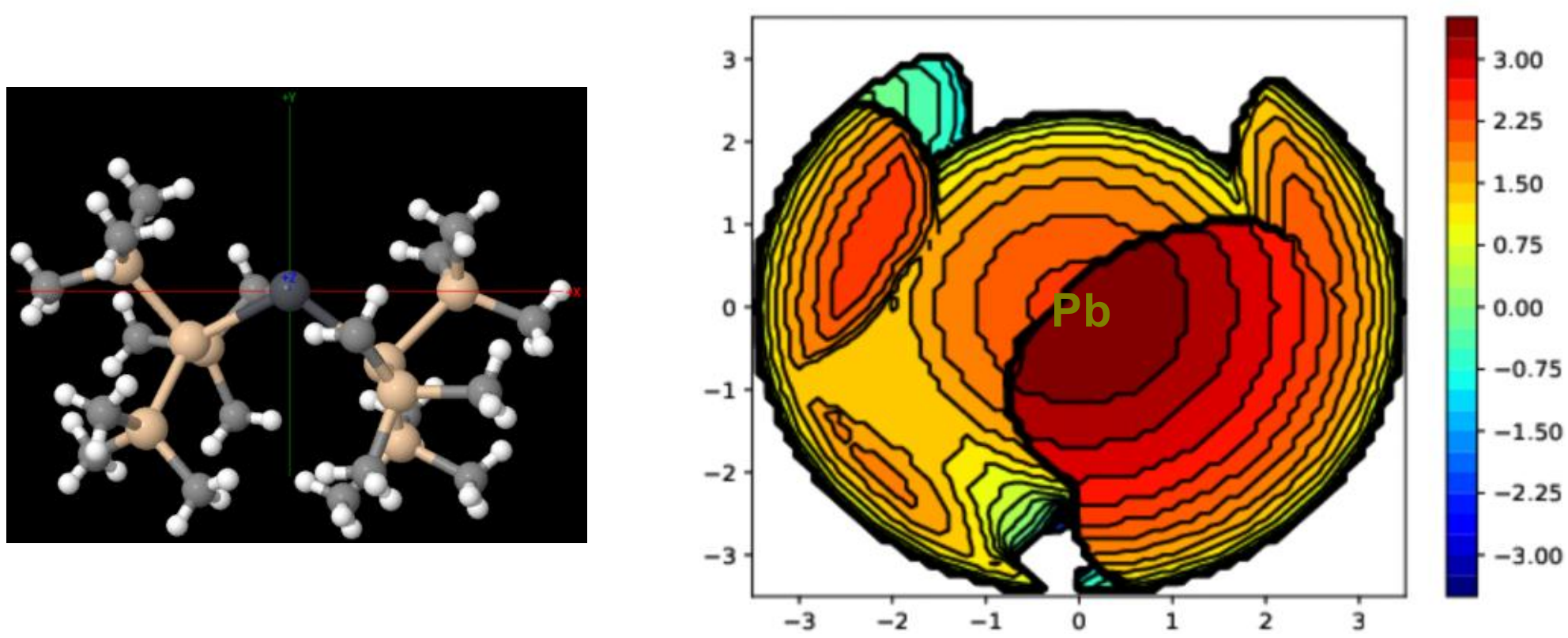


**Figure S47**. a) Orientation of **H** along the z-axis. b) Topographic steric map calculated for **H**, viewed along the z-axis. Steric map axes refer to the x- and y-axes. Brown: lead, pale pink: silicon, grey: carbon, white: hydrogen. $\%V_{bur}$ = 73.8%

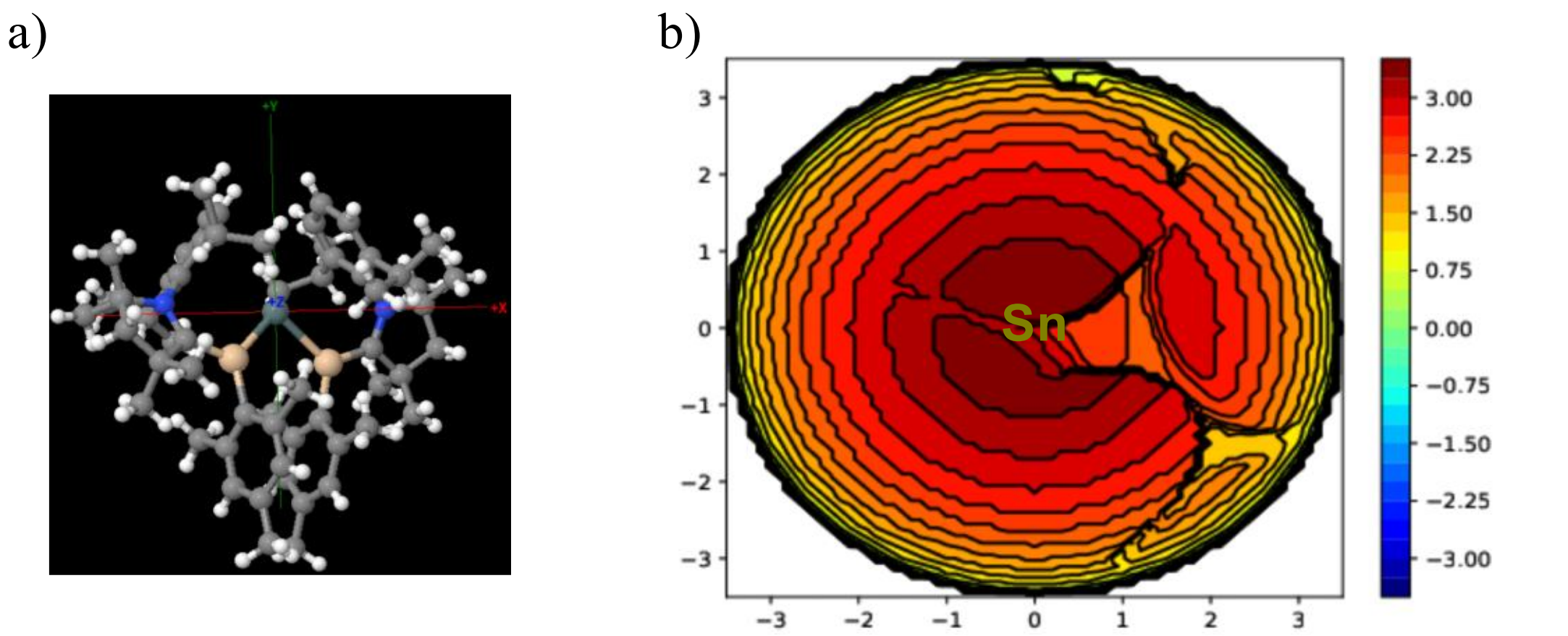


**Figure S48**. a) Orientation of **3** along the z-axis. b) Topographic steric map calculated for **3**, viewed along the z-axis. Steric map axes refer to the x- and y-axes. Brown: tin, pale pink: silicon, blue: nitrogen, grey: carbon, white: hydrogen. $\%V_{bur}$ = 96.2%

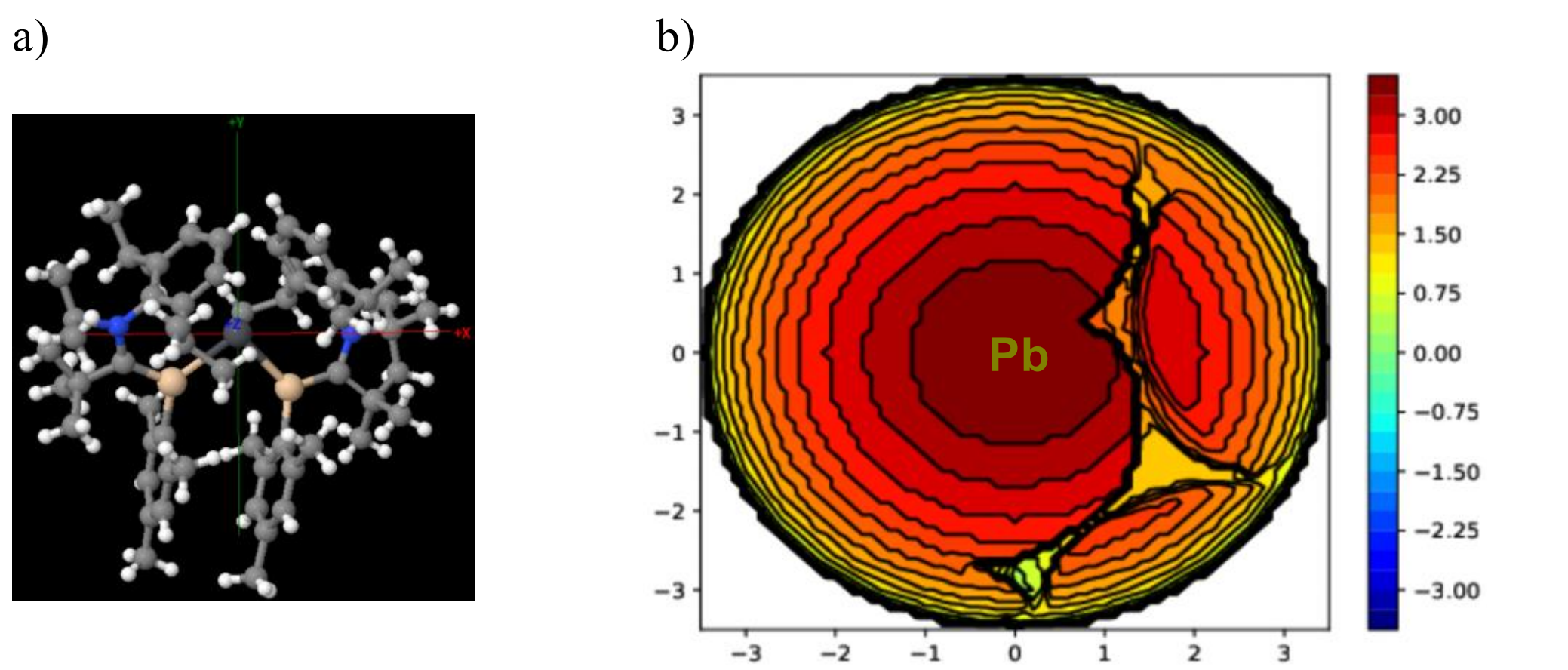


**Figure S49**. a) Orientation of **4** along the z-axis. b) Topographic steric map calculated for **4**, viewed along the z-axis. Steric map axes refer to the x- and y-axes. Brown: lead, pale pink: silicon, blue: nitrogen, grey: carbon, white: hydrogen. $\%V_{bur}$ = 94.3%

## B2.3 Excited State Analysis

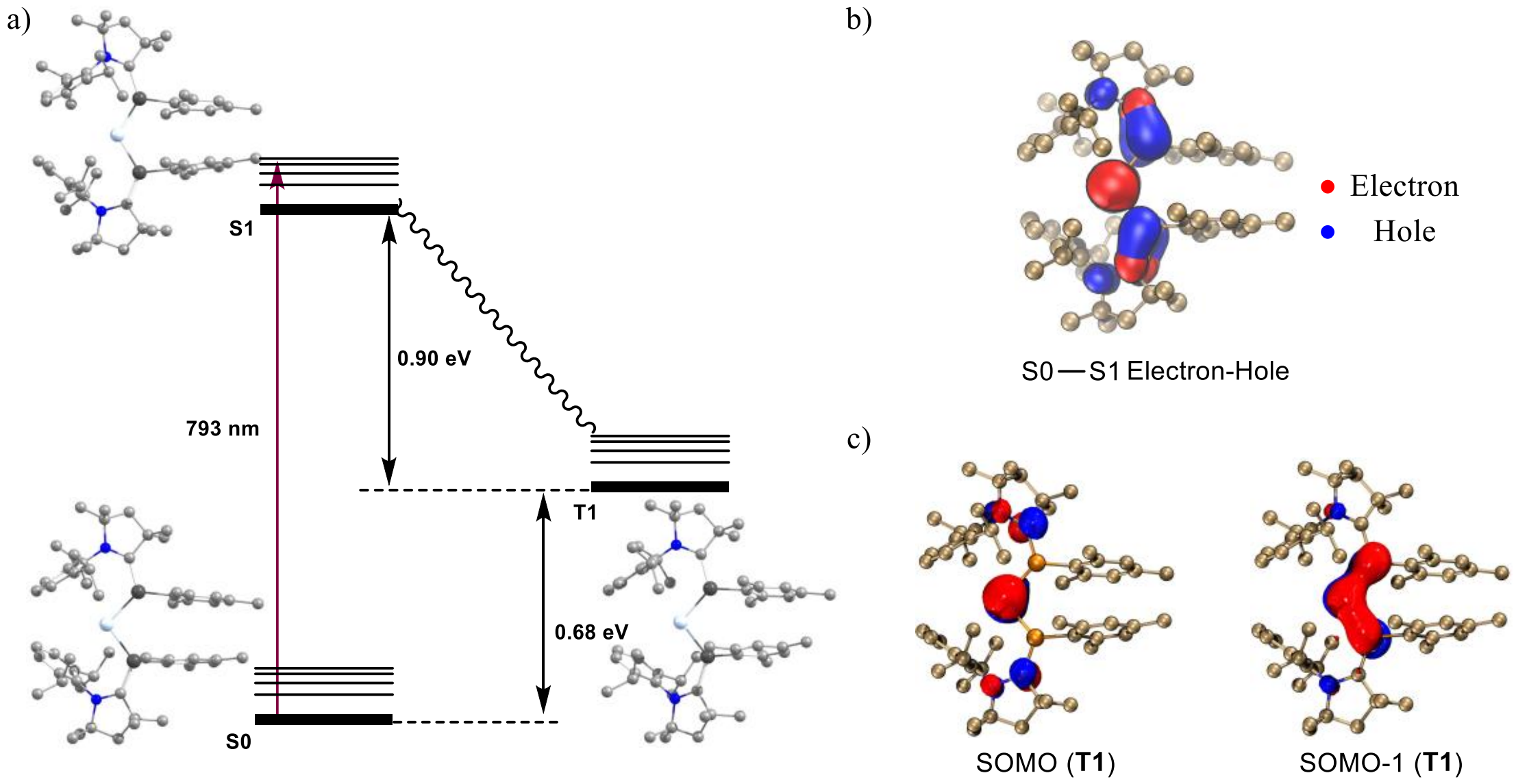


**Figure S50**. a) Simplified Jablonski diagram depicting the calculated first ($S_0$-$S_1$) vertical excitation and the $S_1$-$T_1$ energy difference of **2**. b) electron (red) hole (green) distribution for the $S_0$-$S_1$ transition of **2**. c) the two singly occupied molecular orbitals of **2** with excited $T_1$ state.

**Table S10**. First 20 calculated electronic transitions of **2**, including singlet and triplet excited states with spin–orbit coupling corrections.

| Index | Excit.energy (eV) | nm | 1000 cm$^{-1}$ | Oscil.str. |
|---|---|---|---|---|
| 1 | 0.67668 | 1832.24029 | 5.45780 | 0.00000 |
| 2 | 0.67669 | 1832.20672 | 5.45790 | 0.00000 |
| 3 | 0.67674 | 1832.07245 | 5.45830 | 0.00002 |
| 4 | 1.33448 | 929.08319 | 10.76330 | 0.00000 |
| 5 | 1.33450 | 929.06592 | 10.76350 | 0.00002 |
| 6 | 1.33468 | 928.94510 | 10.76490 | 0.00016 |
| **7** | **1.56430** | **792.58780** | **12.61690** | **0.36603** |
| 8 | 1.75725 | 705.55704 | 14.17320 | 0.00031 |
| 9 | 1.75854 | 705.03970 | 14.18360 | 0.00000 |
| 10 | 1.75864 | 704.99993 | 14.18440 | 0.00002 |
| 11 | 1.96105 | 632.23521 | 15.81690 | 0.02733 |
| 12 | 2.05661 | 602.85640 | 16.58770 | 0.00000 |
| 13 | 2.05661 | 602.85640 | 16.58770 | 0.00000 |
| 14 | 2.05664 | 602.84913 | 16.58790 | 0.00001 |
| 15 | 2.06193 | 601.30128 | 16.63060 | 0.00000 |
| 16 | 2.06194 | 601.29766 | 16.63070 | 0.00000 |
| 17 | 2.06194 | 601.29766 | 16.63070 | 0.00000 |
| 18 | 2.07514 | 597.47513 | 16.73710 | 0.00087 |
| 19 | 2.08183 | 595.55366 | 16.79110 | 0.00199 |
| 20 | 2.08561 | 594.47384 | 16.82160 | 0.00263 |

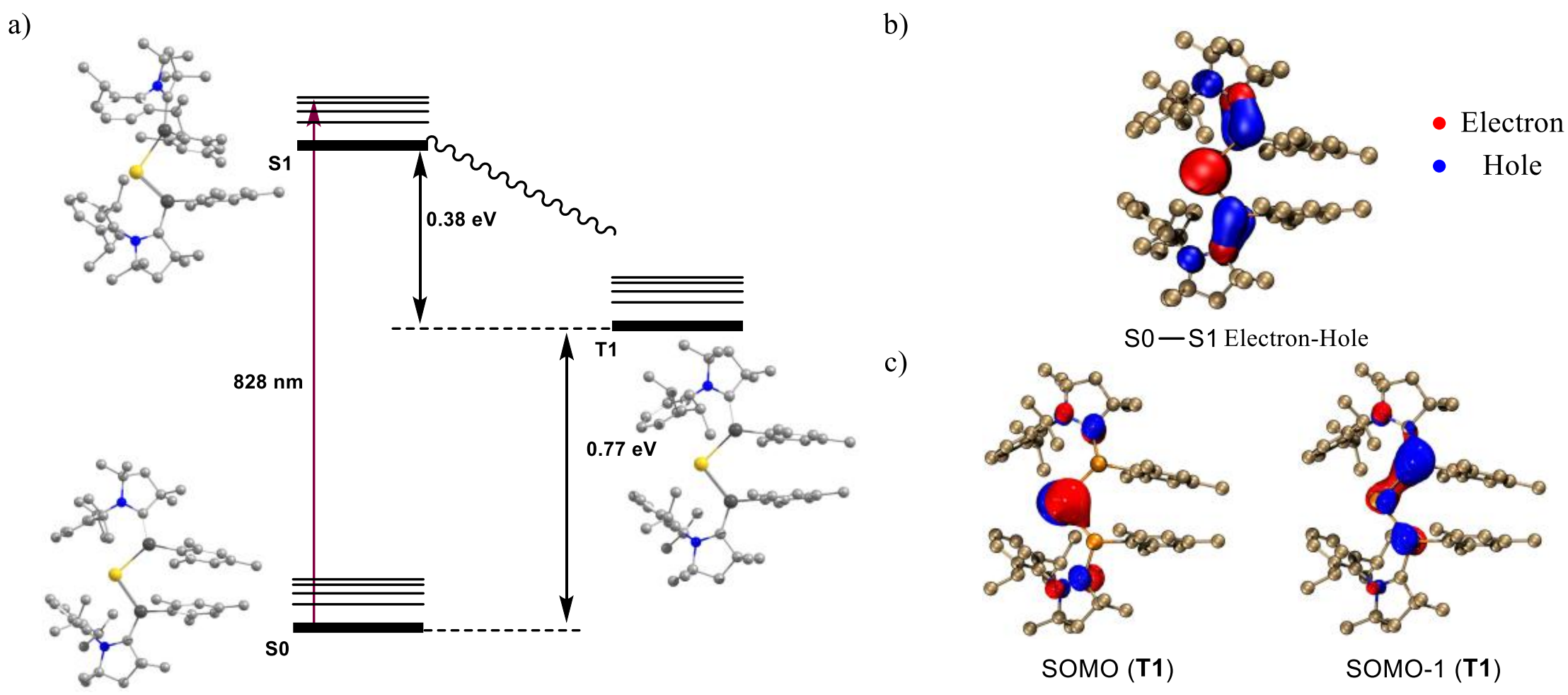


**Figure S51**. a) Simplified Jablonski diagram depicting the calculated first ($S_0$-$S_1$) vertical excitation and the $S_1$-$T_1$ energy difference of **3**. b) electron (red) hole (green) distribution for the $S_0$-$S_1$ transition of **3**. c) the two singly occupied molecular orbitals of **3** with excited $T_1$ state.

**Table S11**. First 20 calculated electronic transitions of **3**, including singlet and triplet excited states with spin–orbit coupling corrections.

| Index | Excit.energy (eV) | nm | 1000 cm$^{-1}$ | Oscil.str. |
|---|---|---|---|---|
| 1 | 0.74795 | 1657.66022 | 6.03260 | 0.00000 |
| 2 | 0.74810 | 1657.33055 | 6.03380 | 0.00005 |
| 3 | 0.74837 | 1656.72649 | 6.03600 | 0.00064 |
| 4 | 1.21383 | 1021.42970 | 9.79020 | 0.00000 |
| 5 | 1.21418 | 1021.13766 | 9.79300 | 0.00016 |
| 6 | 1.21467 | 1020.72074 | 9.79700 | 0.00073 |
| **7** | **1.49702** | **828.20545** | **12.07430** | **0.32228** |
| 8 | 1.70663 | 726.48556 | 13.76490 | 0.00065 |
| 9 | 1.71947 | 721.05859 | 13.86850 | 0.00020 |
| 10 | 1.71997 | 720.85068 | 13.87250 | 0.00016 |
| 11 | 1.87687 | 660.58932 | 15.13800 | 0.00052 |
| 12 | 1.97933 | 626.39379 | 15.96440 | 0.01392 |
| 13 | 2.02024 | 613.71161 | 16.29430 | 0.00001 |
| 14 | 2.02027 | 613.70031 | 16.29460 | 0.00001 |
| 15 | 2.02077 | 613.54970 | 16.29860 | 0.00023 |
| 16 | 2.03387 | 609.59633 | 16.40430 | 0.00002 |
| 17 | 2.03391 | 609.58518 | 16.40460 | 0.00001 |
| 18 | 2.03447 | 609.41801 | 16.40910 | 0.00026 |
| 19 | 2.04224 | 607.09826 | 16.47180 | 0.00031 |
| 20 | 2.07847 | 596.51641 | 16.76400 | 0.00002 |

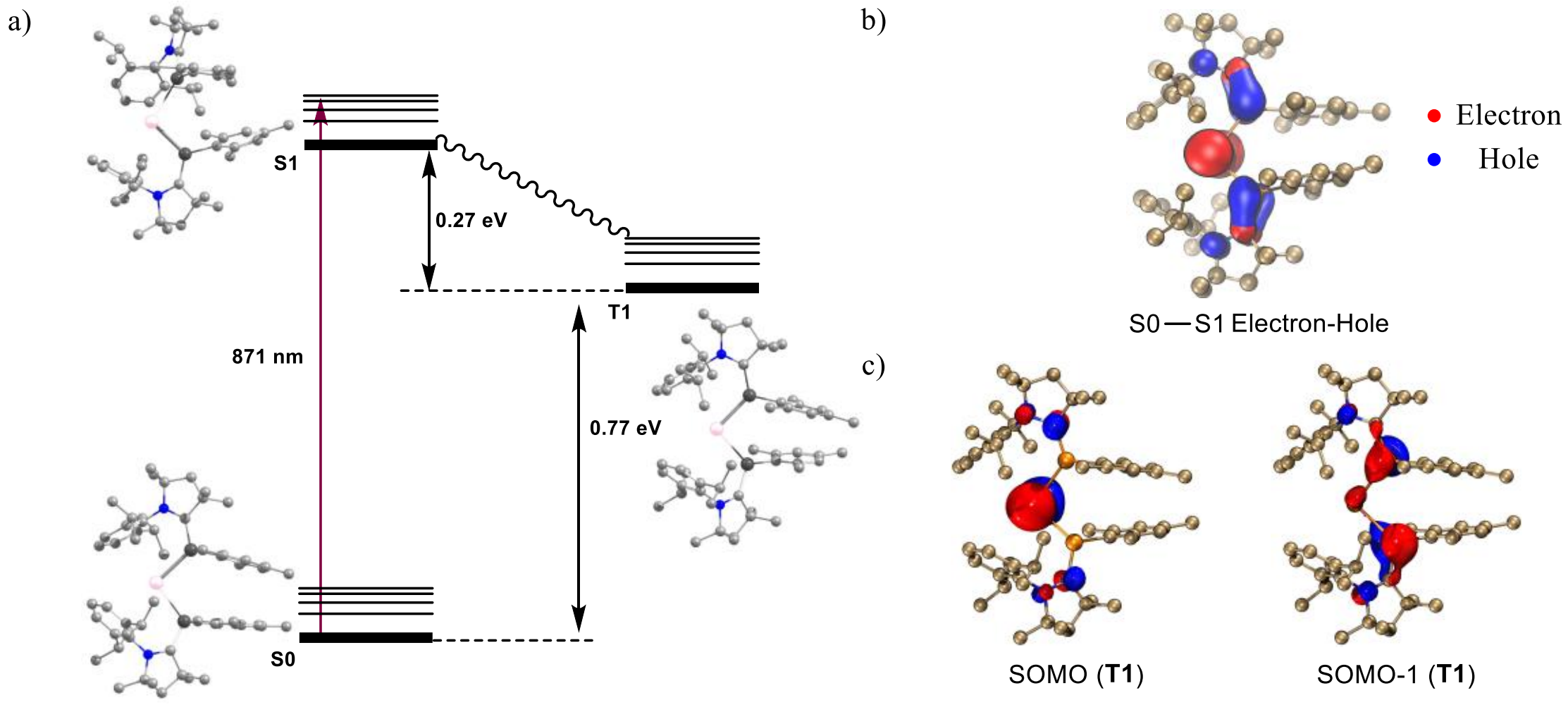


**Figure S52**. a) Simplified Jablonski diagram depicting the calculated first ($S_0$-$S_1$) vertical excitation and the $S_1$-$T_1$ energy difference of **4**. b) electron (red) hole (green) distribution for the $S_0$-$S_1$ transition of **4**. c) the two singly occupied molecular orbitals of **4** with excited $T_1$ state.

**Table S12**. First 20 calculated electronic transitions of **5**, including singlet and triplet excited states with spin–orbit coupling corrections.

| Index | Excit.energy (eV) | nm | 1000 cm$^{-1}$ | Oscil.str. |
|---|---|---|---|---|
| 1 | 0.76190 | 1627.31299 | 6.14510 | 0.00010 |
| 2 | 0.76323 | 1624.45801 | 6.15590 | 0.00020 |
| 3 | 0.76705 | 1616.37077 | 6.18670 | 0.00837 |
| 4 | 1.13334 | 1093.97233 | 9.14100 | 0.00000 |
| 5 | 1.13619 | 1091.22666 | 9.16400 | 0.00304 |
| 6 | 1.13937 | 1088.18676 | 9.18960 | 0.00209 |
| **7** | **1.42374** | **870.83749** | **11.48320** | **0.28082** |
| 8 | 1.68961 | 733.80500 | 13.62760 | 0.00581 |
| 9 | 1.93182 | 641.79916 | 15.58120 | 0.00277 |
| 10 | 1.93541 | 640.61096 | 15.61010 | 0.00034 |
| 11 | 1.94189 | 638.47182 | 15.66240 | 0.00027 |
| 12 | 1.94218 | 638.37808 | 15.66470 | 0.00011 |
| 13 | 1.94258 | 638.24362 | 15.66800 | 0.00011 |
| 14 | 1.95621 | 633.79798 | 15.77790 | 0.00033 |
| 15 | 1.97307 | 628.38155 | 15.91390 | 0.00247 |
| 16 | 1.97710 | 627.10085 | 15.94640 | 0.00003 |
| 17 | 1.98040 | 626.05654 | 15.97300 | 0.00111 |
| 18 | 1.98848 | 623.51143 | 16.03820 | 0.00561 |
| 19 | 2.00945 | 617.00598 | 16.20730 | 0.00081 |
| 20 | 2.01006 | 616.81950 | 16.21220 | 0.00016 |

## B2.4 FMO, NBO, and IBO analyses

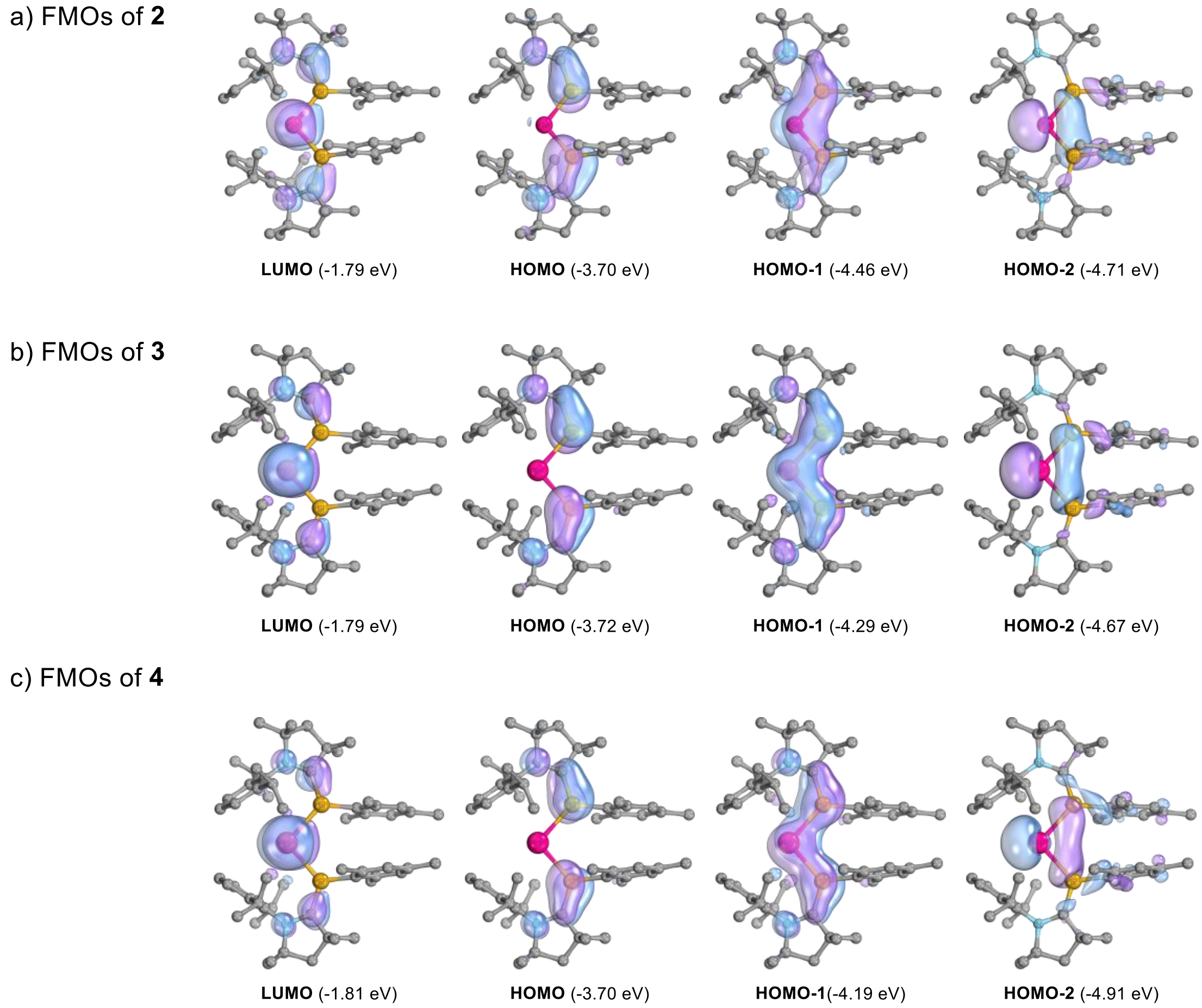


**Figure S53.** Frontier molecular orbitals (FMOs) of compounds **2–4** derived from DFT calculations.

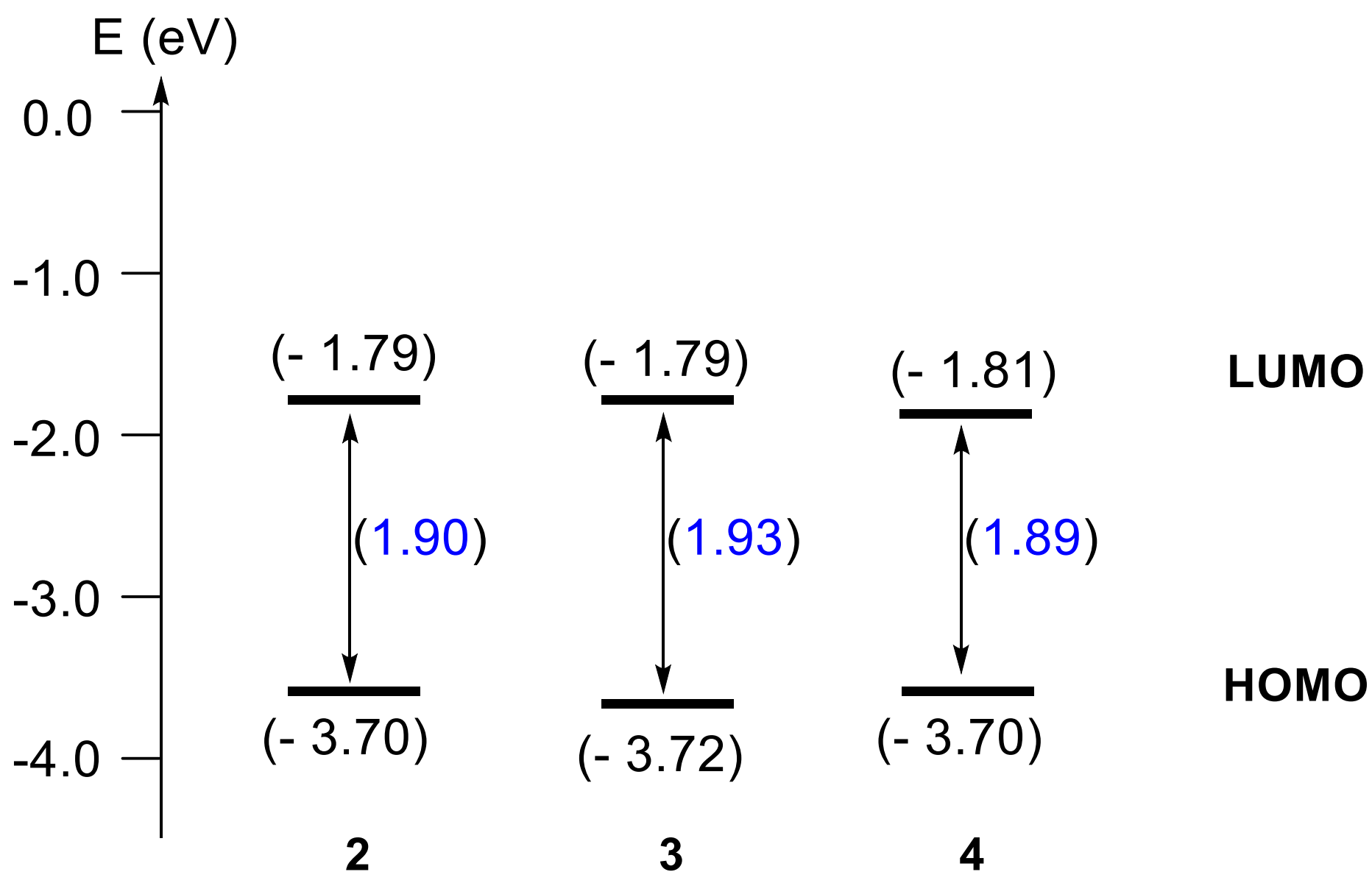


**Figure S54** Energy diagrams of the frontier Kohn–Sham orbitals of compounds **2**–**4**. HOMO = highest occupied molecular orbital; LUMO = lowest unoccupied molecular orbital.

**Table S13**. Selected Wiberg Bond Indices (WBIs) of **2-4**.

| Compounds | **2** | **3** | **4** |
|---|---|---|---|
| E(1)–Si(1) | 1.20 (E = Ge) | 1.08 (E = Sn) | 1.03 (E = Pb) |
| E(1)–Si(2) | 1.23 (E = Ge) | 1.08 (E = Sn) | 1.02 (E = Pb) |
| Si(1)–C(1) | 1.23 | 1.27 | 1.28 |
| Si(1)–C(3) | 0.77 | 0.78 | 0.78 |
| Si(2)–C(2) | 1.22 | 1.27 | 1.30 |
| Si(2)–C(4) | 0.77 | 0.78 | 0.78 |
| C(1)–N(1) | 1.18 | 1.16 | 1.15 |
| C(2)–N(2) | 1.19 | 1.15 | 1.14 |

**Table S14**. Selected Natural Population Analysis (NPA) charge (blue color, a.u.) of **2-4**.

| Compounds | **2** | **3** | **4** |
|---|---|---|---|
| Si(1) | 0.68 | 0.56 | 0.56 |
| E(1)) | -0.09 (E = Ge) | 0.22 (E = Sn) | 0.31 (E = Pb) |
| Si(2) | 0.67 | 0.55 | 0.54 |
| C(1) | -0.21 | -0.24 | -0.26 |
| C(2) | -0.22 | -0.23 | -0.25 |

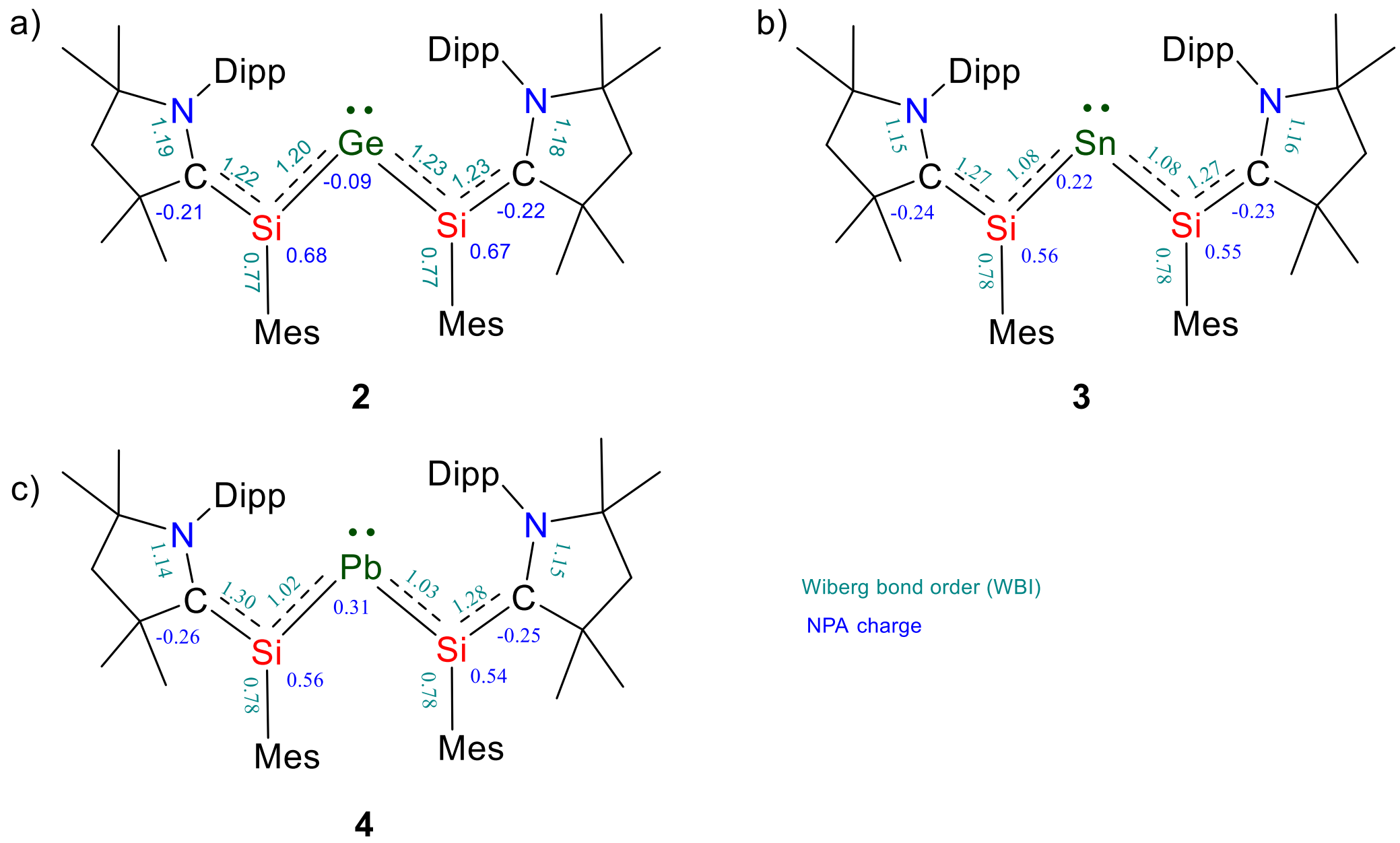


**Figure S55**. Selected Wiberg bond order (green color.) and the NPA charge (blue color) of **2-4**.

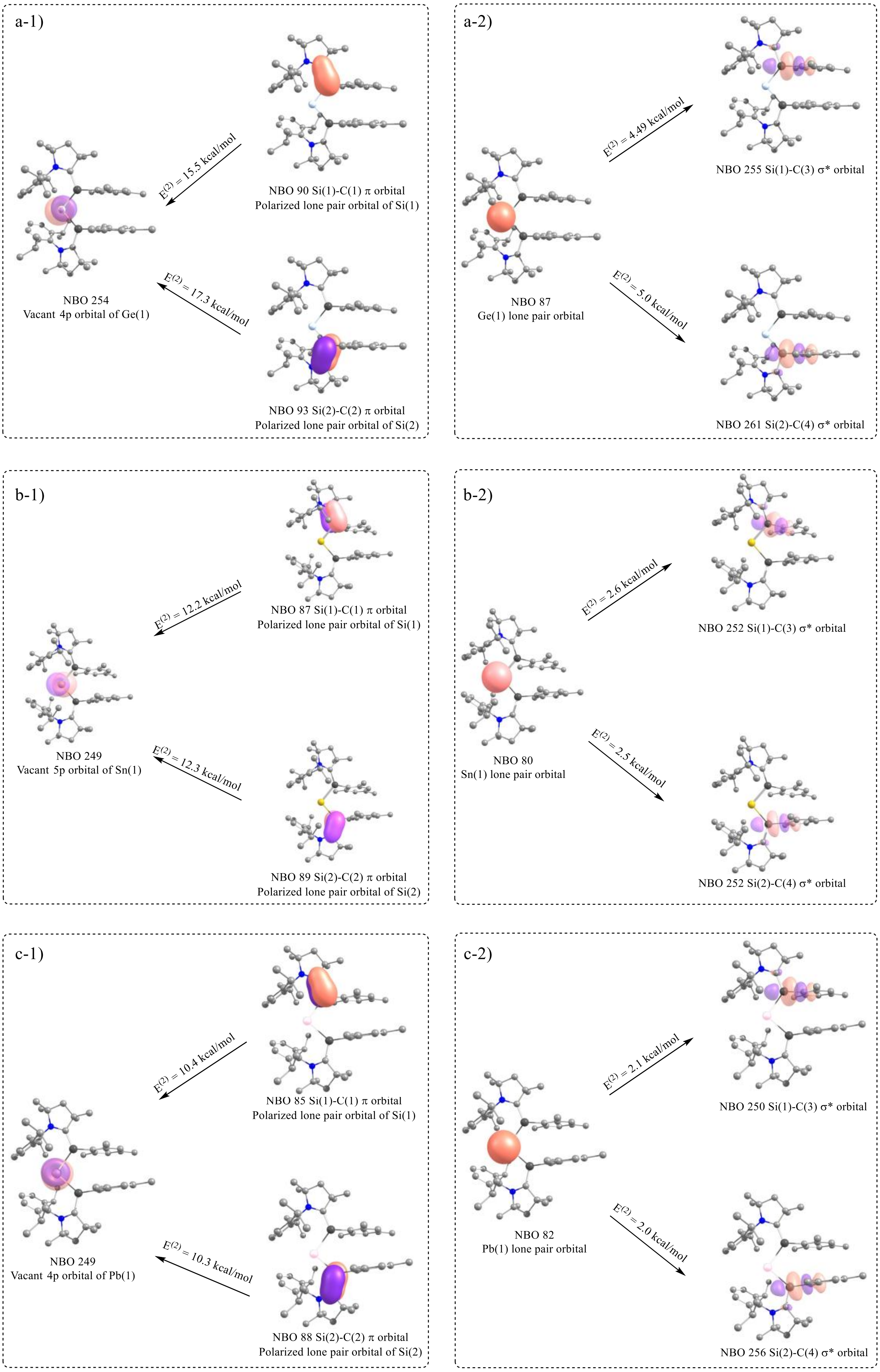


**Figure S56**. Selected NBOs of **2-4** for the second-order perturbation theory analysis.

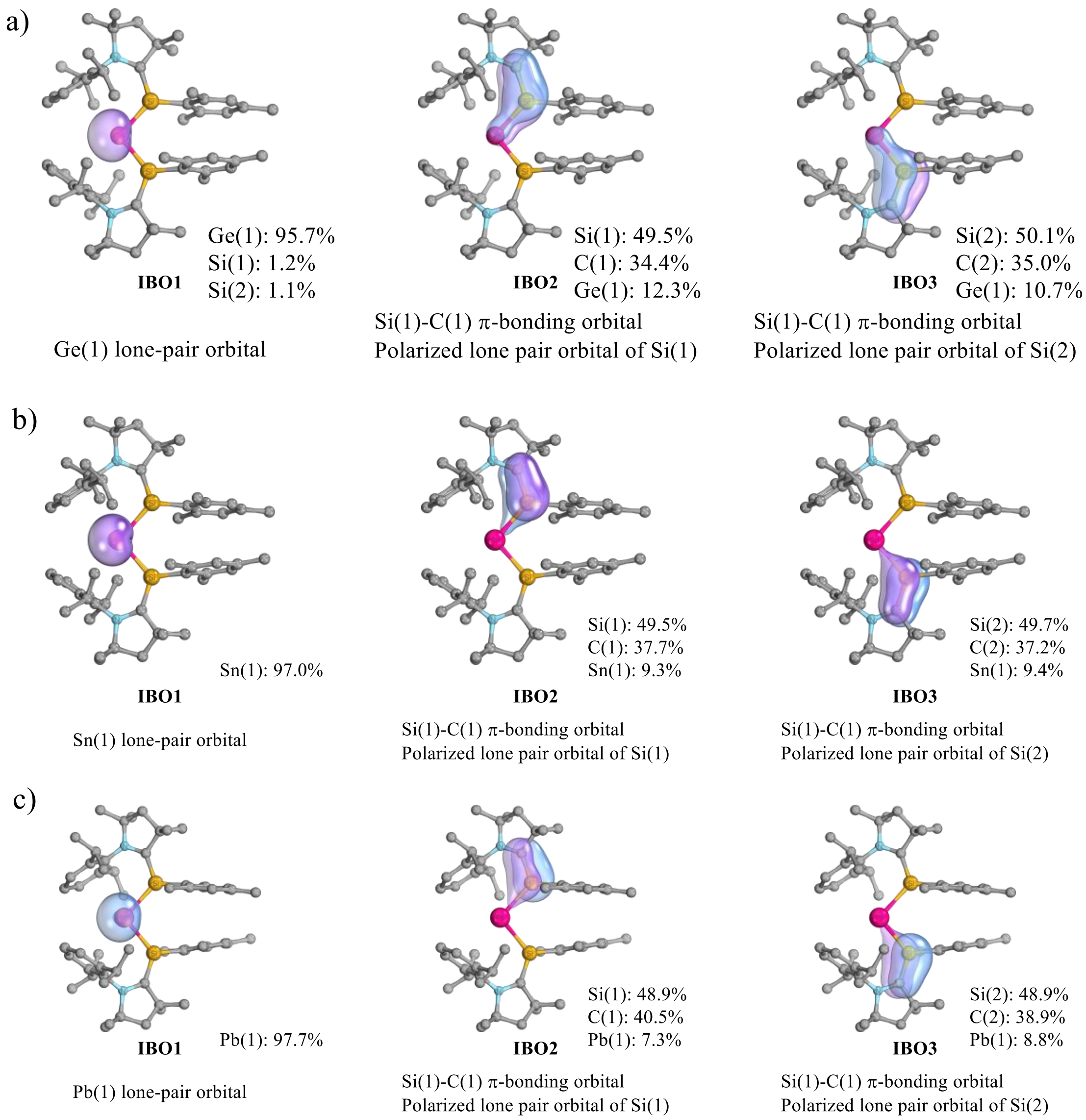


**Figure S57**. Selected IBOs for **2-4**. a) the Ge lone pair orbital and two polarized lone-pair orbitals of Si in **2**. b) the Sn lone-pair orbital and two polarized lone pair orbitals of Si in **3**. c) the Pb lone-pair orbital and two polarized lone pair orbitals of Si in **4**.

B2.5 Natural Resonance Theory (NRT) of simplified model of **2**

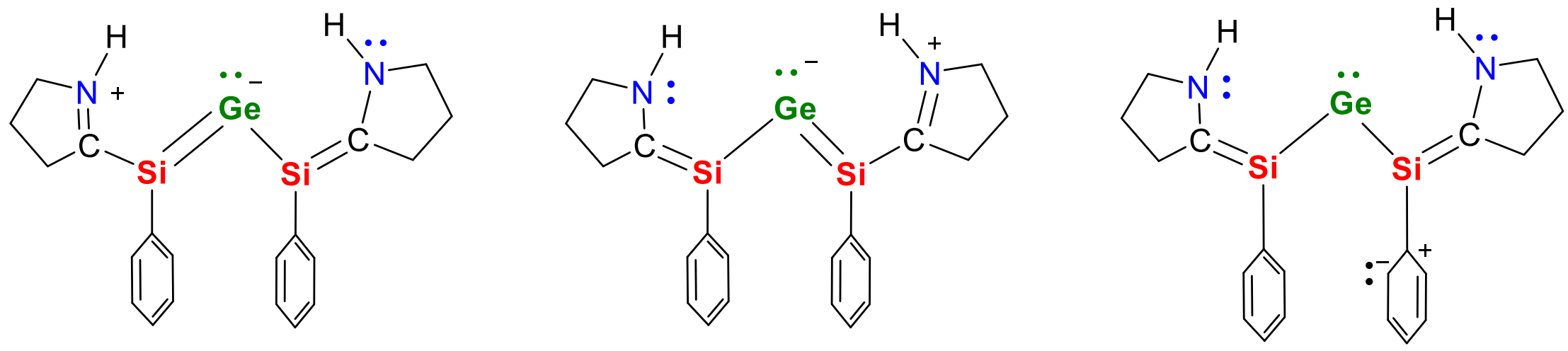


***2′***,10.87 % ***2″***, 9.13 % ***2‴***, 3.95 %

**Figure S58**. Predominant resonance structures (each > 3% contribution) for a simplified model of **2**, together with their calculated contributions.

B2.6 Energy Decomposition Analysis (EDA) of **2**

**Table S15.** Energy Decomposition Analysis of the Ge–Si Bonds at b3lyp/6-31+G(d,p) level

| Partitioning | Closed shell, charged fragments | | Open shell, neutral fragments | |
|---|---|---|---|---|
| | 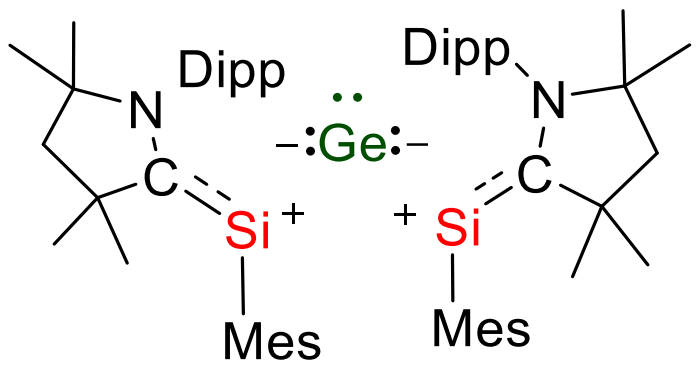 | | 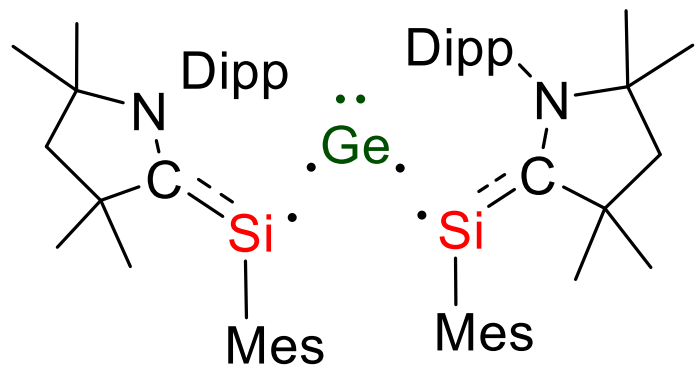 | |
| | kcal·mol$^{-1}$ | (%) | kcal·mol$^{-1}$ | (%) |
| $\Delta E_{int}$ | -543.16 | | -120.47 | |
| $\Delta E_{pauli}$ | +1465.08 | | +744.31 | |
| $\Delta E_{elstat}$ | -767.10 | 52.2% | -286.94 | 47.0% |
| $\Delta E_{orb}$ | -649.54 | 44.2% | -272.05 | 44.5% |
| $\Delta E_{disp}$ | -52.11 | 3.5% | -52.11 | 8.5% |

B2.7 Spin-density and SOMO of Ge(I) and Sn(I) radical anions, **2**$^{\bullet-}$ and **3**$^{\bullet-}$

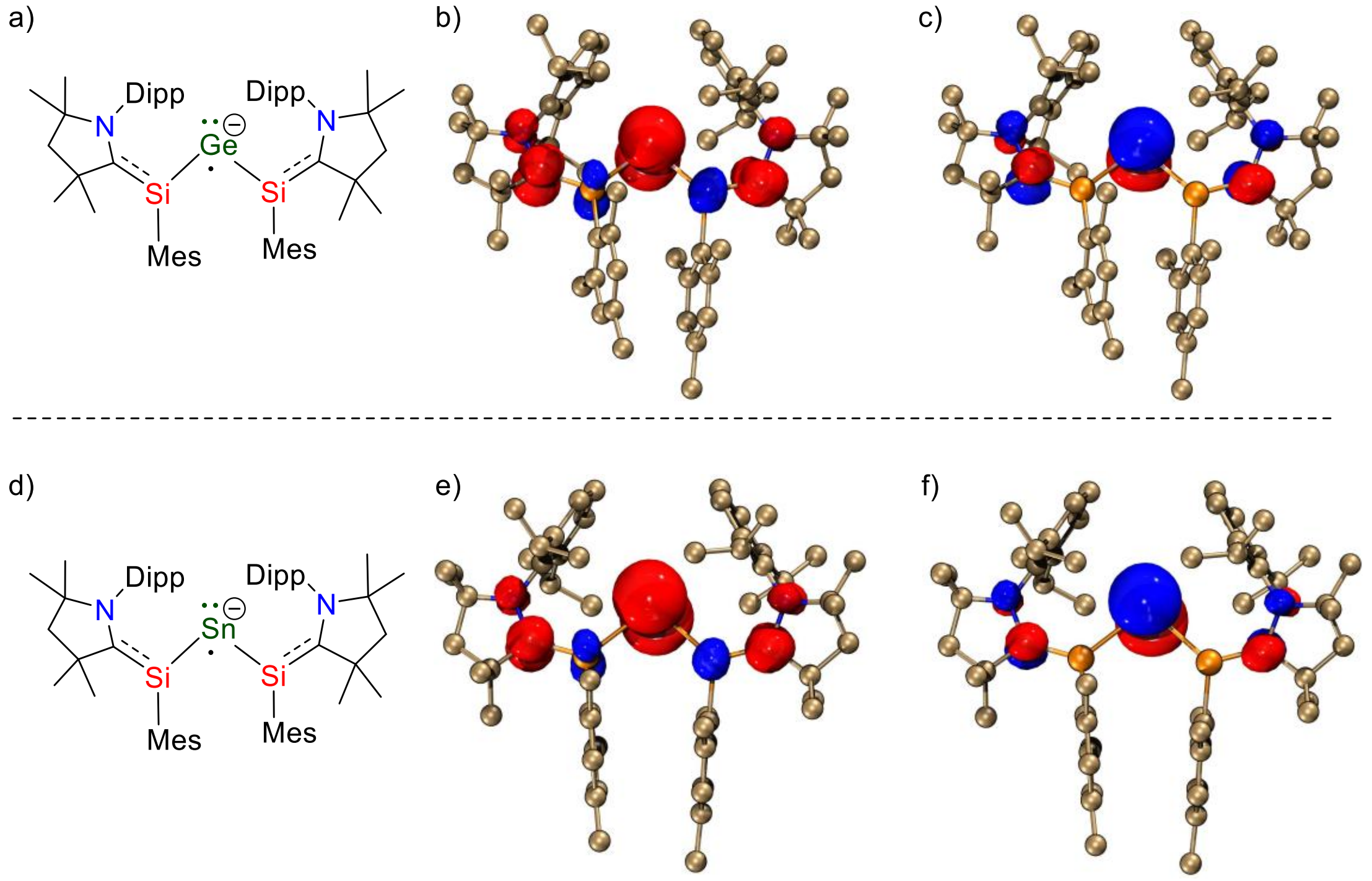


**Figure S59**. a) Molecular structure of **2**$^{\bullet-}$; b) calculated spin-density distribution of **2**$^{\bullet-}$ (isovalue = 0.003); c) SOMO of **2**$^{\bullet-}$ (isovalue = 0.05). d) Molecular structure of **3**$^{\bullet-}$; e) calculated spin-density distribution of **3**$^{\bullet-}$ (isovalue = 0.003); f) SOMO of **3**$^{\bullet-}$ (isovalue = 0.05).

**B3 Optimized x,y,z-coordinates**

**silanylidene anion** (singlet)

| | | | |
|---|---|---|---|
| Si | 0.811564 | -0.409431 | -1.032782 |
| N | -1.392960 | -0.167209 | 0.711078 |
| C | 2.743357 | -0.209879 | -0.704749 |
| C | 0.042684 | -0.108120 | 0.568204 |
| C | 3.551954 | -1.358106 | -0.458662 |
| C | 3.428993 | 1.018311 | -0.914980 |
| C | 4.946310 | -1.250283 | -0.355752 |
| H | 5.536332 | -2.150929 | -0.145191 |
| C | 4.827428 | 1.094556 | -0.805279 |
| H | 5.321385 | 2.062321 | -0.955509 |
| C | 5.609747 | -0.027035 | -0.513336 |
| C | 7.115084 | 0.059447 | -0.415323 |
| H | 7.501155 | -0.523523 | 0.438666 |
| H | 7.454647 | 1.100777 | -0.292061 |
| H | 7.608548 | -0.339825 | -1.321119 |
| C | 2.924467 | -2.728635 | -0.316547 |
| H | 2.199630 | -2.760523 | 0.510931 |
| H | 3.686598 | -3.507981 | -0.146960 |
| H | 2.349634 | -2.991500 | -1.221561 |
| C | 2.668594 | 2.272652 | -1.284692 |
| H | 2.058631 | 2.104221 | -2.189089 |
| H | 3.348593 | 3.120051 | -1.474517 |
| H | 1.959372 | 2.569881 | -0.498570 |
| C | 0.587410 | 0.209641 | 1.990092 |
| C | -0.695255 | 0.296212 | 2.865819 |
| H | -0.541094 | -0.096766 | 3.884893 |
| H | -1.003454 | 1.349910 | 2.969067 |
| C | -1.802297 | -0.456447 | 2.096039 |
| C | -3.196743 | 0.089191 | 2.417460 |
| H | -3.257733 | 1.168031 | 2.218039 |
| H | -3.431944 | -0.083152 | 3.480625 |
| H | -3.968321 | -0.407760 | 1.808831 |
| C | -1.767047 | -1.966423 | 2.440918 |
| H | -2.629120 | -2.495506 | 2.013532 |
| H | -1.789067 | -2.118676 | 3.533500 |
| H | -0.852063 | -2.428112 | 2.044975 |
| C | 1.544726 | -0.872094 | 2.531677 |
| H | 2.449776 | -0.924409 | 1.912605 |
| H | 1.077589 | -1.866744 | 2.536005 |
| H | 1.852740 | -0.635357 | 3.567312 |
| C | 1.342137 | 1.553969 | 2.049388 |
| H | 2.278426 | 1.495897 | 1.478206 |

| | | | |
|---|---|---|---|
| H | 1.595613 | 1.817579 | 3.094055 |
| H | 0.733823 | 2.370231 | 1.630913 |
| C | -2.302679 | 0.114962 | -0.339419 |
| C | -2.952026 | -0.927889 | -1.051883 |
| C | -2.597995 | 1.468600 | -0.673967 |
| C | -3.928790 | -0.606238 | -2.008535 |
| H | -4.435174 | -1.412807 | -2.547056 |
| C | -4.244259 | 0.716215 | -2.305162 |
| H | -5.005710 | 0.949061 | -3.055836 |
| C | -3.564312 | 1.743659 | -1.647778 |
| H | -3.794672 | 2.784250 | -1.894746 |
| C | -2.572745 | -2.388920 | -0.853938 |
| H | -1.764726 | -2.407791 | -0.112206 |
| C | -3.751646 | -3.227604 | -0.332021 |
| H | -4.198304 | -2.786064 | 0.572866 |
| H | -3.426911 | -4.253512 | -0.087218 |
| H | -4.552104 | -3.304663 | -1.087809 |
| C | -1.997586 | -2.998577 | -2.144150 |
| H | -2.749614 | -3.028752 | -2.951999 |
| H | -1.660717 | -4.034027 | -1.961056 |
| H | -1.131456 | -2.409627 | -2.483026 |
| C | -2.812636 | 3.635760 | 0.656220 |
| H | -3.447931 | 4.152367 | -0.082946 |
| H | -2.235101 | 4.409536 | 1.190621 |
| H | -3.481195 | 3.148338 | 1.383529 |
| C | -1.865625 | 2.625953 | -0.011368 |
| H | -1.233486 | 2.189285 | 0.770073 |
| C | -0.936788 | 3.323731 | -1.017745 |
| H | -0.277550 | 2.584306 | -1.493841 |
| H | -0.306628 | 4.076928 | -0.514159 |
| H | -1.516367 | 3.837887 | -1.804550 |

**silanylidene anion** (triplet)

| | | | |
|---|---|---|---|
| Si | -0.884535 | -0.664835 | -1.212541 |
| N | 1.268247 | 0.049234 | 0.712020 |
| C | -2.720455 | -0.142150 | -0.766054 |
| C | 0.035992 | -0.622158 | 0.500993 |
| C | -3.110441 | 0.951358 | 0.070675 |
| C | -3.793317 | -0.881700 | -1.363384 |
| C | -4.457434 | 1.219622 | 0.343360 |
| H | -4.707502 | 2.063825 | 0.998716 |
| C | -5.131237 | -0.580033 | -1.074877 |
| H | -5.921085 | -1.183722 | -1.539483 |

| | | | |
|---|---|---|---|
| C | -5.496445 | 0.458249 | -0.208069 |
| C | -6.940956 | 0.727299 | 0.138744 |
| H | -7.260893 | 0.172523 | 1.042221 |
| H | -7.619159 | 0.425203 | -0.677350 |
| H | -7.118219 | 1.796969 | 0.344188 |
| C | -2.073558 | 1.891775 | 0.629908 |
| H | -1.215308 | 1.330428 | 1.023367 |
| H | -2.482058 | 2.540396 | 1.422617 |
| H | -1.672208 | 2.537929 | -0.167977 |
| C | -3.521256 | -2.024010 | -2.319512 |
| H | -2.968686 | -1.683614 | -3.211940 |
| H | -4.460042 | -2.497657 | -2.654891 |
| H | -2.877301 | -2.791800 | -1.856524 |
| C | -0.247473 | -1.493067 | 1.741068 |
| C | 1.005143 | -1.269901 | 2.634622 |
| H | 0.761083 | -1.231968 | 3.709102 |
| H | 1.716114 | -2.101241 | 2.496931 |
| C | 1.672060 | 0.039212 | 2.142871 |
| C | 3.191685 | 0.016811 | 2.333994 |
| H | 3.649545 | -0.850505 | 1.841807 |
| H | 3.428384 | -0.032408 | 3.409419 |
| H | 3.659343 | 0.923403 | 1.919654 |
| C | 1.105712 | 1.262836 | 2.896887 |
| H | 1.617373 | 2.186632 | 2.591899 |
| H | 1.241014 | 1.144035 | 3.985396 |
| H | 0.034728 | 1.387526 | 2.694863 |
| C | -1.542729 | -1.079384 | 2.477249 |
| H | -2.403866 | -1.137024 | 1.796729 |
| H | -1.492595 | -0.048833 | 2.855797 |
| H | -1.728603 | -1.744855 | 3.340448 |
| C | -0.397852 | -2.984418 | 1.364539 |
| H | -1.251918 | -3.117308 | 0.681051 |
| H | -0.566706 | -3.608103 | 2.261983 |
| H | 0.495767 | -3.361836 | 0.844840 |
| C | 2.187028 | 0.346454 | -0.341299 |
| C | 2.365234 | 1.687466 | -0.770343 |
| C | 2.956861 | -0.686835 | -0.949943 |
| C | 3.330084 | 1.978529 | -1.746552 |
| H | 3.465380 | 3.013881 | -2.072339 |
| C | 4.095390 | 0.972710 | -2.328998 |
| H | 4.839272 | 1.214377 | -3.093999 |
| C | 3.892303 | -0.350832 | -1.935916 |
| H | 4.482836 | -1.143835 | -2.402875 |
| C | 1.474592 | 2.812091 | -0.264600 |

| | | | |
|---|---|---|---|
| H | 0.777370 | 2.364417 | 0.453122 |
| C | 2.267622 | 3.913687 | 0.455131 |
| H | 2.882516 | 3.501864 | 1.271526 |
| H | 1.587089 | 4.667525 | 0.886730 |
| H | 2.948829 | 4.438330 | -0.236540 |
| C | 0.630280 | 3.390631 | -1.412840 |
| H | 1.263103 | 3.874373 | -2.176506 |
| H | -0.076247 | 4.148266 | -1.033734 |
| H | 0.051867 | 2.588993 | -1.899274 |
| C | 4.135769 | -2.808515 | -0.167739 |
| H | 4.822824 | -2.895461 | -1.025866 |
| H | 3.959850 | -3.828545 | 0.214071 |
| H | 4.657689 | -2.237028 | 0.616280 |
| C | 2.801946 | -2.153738 | -0.567955 |
| H | 2.142117 | -2.191506 | 0.304731 |
| C | 2.119035 | -2.955867 | -1.687995 |
| H | 1.140131 | -2.516588 | -1.941078 |
| H | 1.959107 | -4.001271 | -1.371016 |
| H | 2.743343 | -2.969474 | -2.598469 |

**2** (singlet)

| | | | |
|---|---|---|---|
| Si | 1.234723 | -2.009093 | 11.390454 |
| Si | -0.045587 | 1.353599 | 11.692645 |
| N | -0.737915 | 3.241118 | 13.740154 |
| N | 1.334135 | -4.409643 | 12.965657 |
| C | 1.359973 | -1.595382 | 8.510907 |
| C | -1.357749 | 4.073540 | 11.617008 |
| C | 2.041749 | -1.473227 | 9.747016 |
| C | -0.723735 | 2.897867 | 12.408111 |
| C | 1.120825 | 2.047573 | 9.141777 |
| C | -1.382868 | 1.545990 | 15.391118 |
| C | 1.132868 | 2.159407 | 7.746417 |
| H | 2.003202 | 2.608716 | 7.258673 |
| C | 2.442540 | -4.726762 | 10.911918 |
| C | 1.952945 | -1.119339 | 7.333919 |
| H | 1.407578 | -1.221683 | 6.390419 |
| C | -0.001164 | 1.471700 | 9.791995 |
| C | -1.034873 | 0.728282 | 16.472448 |
| H | -1.800468 | 0.107204 | 16.940479 |
| C | 2.046832 | -4.762321 | 9.427352 |
| H | 2.376889 | -3.869640 | 8.885750 |
| H | 0.957641 | -4.858497 | 9.312189 |
| H | 2.518713 | -5.638594 | 8.951635 |

| | | | |
|---|---|---|---|
| C | 3.318545 | -0.848729 | 9.755073 |
| C | -0.716733 | -3.776992 | 14.183686 |
| C | 3.953961 | -4.419742 | 11.006957 |
| H | 4.536500 | -5.221105 | 10.522094 |
| H | 4.295324 | -4.329191 | 12.047940 |
| H | 4.181596 | -3.475836 | 10.493401 |
| C | 2.324190 | 2.537587 | 9.915978 |
| H | 2.646701 | 1.792568 | 10.655890 |
| H | 2.109122 | 3.461936 | 10.474675 |
| H | 3.173238 | 2.733812 | 9.244691 |
| C | 0.269463 | 0.679857 | 16.954168 |
| H | 0.516578 | 0.036401 | 17.801592 |
| C | -1.050200 | 0.946747 | 8.996856 |
| C | 3.208375 | -0.510362 | 7.331712 |
| C | 0.849860 | -2.688138 | 16.246322 |
| H | 1.446714 | -2.235749 | 17.040230 |
| C | 3.876888 | -0.389007 | 8.555729 |
| H | 4.861720 | 0.088544 | 8.576435 |
| C | 0.011254 | -2.264440 | 8.379553 |
| H | -0.422291 | -2.519374 | 9.357562 |
| H | 0.094562 | -3.198413 | 7.799700 |
| H | -0.694925 | -1.610457 | 7.848095 |
| C | -1.636665 | -4.286393 | 13.074723 |
| H | -1.004400 | -4.716769 | 12.285939 |
| C | 0.701501 | -3.791943 | 14.096225 |
| C | -1.002197 | 1.083213 | 7.602487 |
| H | -1.826153 | 0.685879 | 7.000937 |
| C | 1.496002 | -3.199088 | 15.113712 |
| C | -0.534855 | -2.723530 | 16.370958 |
| H | -1.015435 | -2.320050 | 17.265221 |
| C | 1.656321 | -3.738245 | 11.806710 |
| C | 0.077912 | 1.692916 | 6.956472 |
| C | 2.090689 | -6.098449 | 11.532400 |
| H | 1.217695 | -6.511390 | 11.003376 |
| H | 2.909568 | -6.825046 | 11.422593 |
| C | -0.370635 | 2.347208 | 14.799618 |
| C | -1.306876 | -3.244107 | 15.338412 |
| H | -2.395469 | -3.233071 | 15.425687 |
| C | 0.973359 | 2.266467 | 15.246479 |
| C | 3.430892 | -1.571118 | 14.872763 |
| H | 3.009977 | -1.127269 | 13.962231 |
| H | 4.529130 | -1.489588 | 14.816502 |
| H | 3.087400 | -0.966906 | 15.727128 |
| C | 3.013313 | -3.044901 | 15.021502 |

| | | | |
|---|---|---|---|
| H | 3.347861 | -3.560494 | 14.114282 |
| C | -2.606903 | -5.373612 | 13.575605 |
| H | -2.094891 | -6.178347 | 14.120889 |
| H | -3.147193 | -5.823884 | 12.726985 |
| H | -3.364747 | -4.947067 | 14.252195 |
| C | -2.212202 | 0.204956 | 9.618660 |
| H | -1.854553 | -0.679830 | 10.169058 |
| H | -2.926950 | -0.135707 | 8.854358 |
| H | -2.758442 | 0.822696 | 10.346849 |
| C | -2.827542 | 1.521058 | 14.894774 |
| H | -2.940622 | 2.322468 | 14.151819 |
| C | 1.718498 | -5.861688 | 12.999741 |
| C | 1.262153 | 1.431745 | 16.335851 |
| H | 2.290130 | 1.365217 | 16.698723 |
| C | 0.554872 | -6.761187 | 13.434460 |
| H | 0.216987 | -6.515150 | 14.451911 |
| H | 0.887117 | -7.810761 | 13.431237 |
| H | -0.298055 | -6.677256 | 12.750770 |
| C | 2.895807 | -6.139201 | 13.949995 |
| H | 3.791991 | -5.562998 | 13.690688 |
| H | 3.156222 | -7.207679 | 13.902170 |
| H | 2.619322 | -5.907930 | 14.986540 |
| C | 3.796483 | 0.051922 | 6.062316 |
| H | 3.484533 | -0.526256 | 5.178391 |
| H | 4.897012 | 0.068396 | 6.092642 |
| H | 3.456883 | 1.091160 | 5.905184 |
| C | 0.148119 | 1.770625 | 5.452272 |
| H | -0.852545 | 1.756824 | 4.993346 |
| H | 0.708767 | 0.909923 | 5.045710 |
| H | 0.668632 | 2.681248 | 5.116146 |
| C | -0.323290 | 4.853688 | 10.778522 |
| H | 0.552644 | 5.155114 | 11.368857 |
| H | -0.788165 | 5.766473 | 10.370297 |
| H | 0.024515 | 4.248710 | 9.932247 |
| C | 4.094873 | -0.638832 | 11.037163 |
| H | 3.566111 | 0.050218 | 11.714131 |
| H | 5.090389 | -0.214435 | 10.836937 |
| H | 4.225745 | -1.572900 | 11.603544 |
| C | -3.167229 | 0.199480 | 14.181675 |
| H | -2.543988 | 0.058052 | 13.289634 |
| H | -4.224877 | 0.194341 | 13.869457 |
| H | -3.001103 | -0.664333 | 14.845001 |
| C | -2.499856 | 3.633873 | 10.681863 |
| H | -2.125898 | 3.089323 | 9.807060 |

| | | | |
|---|---|---|---|
| H | -3.036651 | 4.526406 | 10.317842 |
| H | -3.222207 | 2.995233 | 11.212737 |
| C | 2.126492 | 3.015170 | 14.584416 |
| H | 1.709763 | 3.606856 | 13.760281 |
| C | -3.834427 | 1.773256 | 16.032286 |
| H | -3.880727 | 0.915064 | 16.721499 |
| H | -4.847598 | 1.915275 | 15.622587 |
| H | -3.575936 | 2.661077 | 16.628247 |
| C | -1.163986 | 4.653877 | 14.017902 |
| C | 2.840117 | 3.962967 | 15.566080 |
| H | 2.138363 | 4.617270 | 16.103776 |
| H | 3.561850 | 4.600974 | 15.030479 |
| H | 3.402933 | 3.395837 | 16.324948 |
| C | -2.445861 | -3.146072 | 12.429373 |
| H | -3.110231 | -2.660459 | 13.161793 |
| H | -3.074250 | -3.539995 | 11.613663 |
| H | -1.785282 | -2.376420 | 12.008948 |
| C | 3.153798 | 2.052514 | 13.962831 |
| H | 3.641689 | 1.427926 | 14.727585 |
| H | 3.940655 | 2.619533 | 13.439002 |
| H | 2.671612 | 1.386985 | 13.235401 |
| C | 3.742092 | -3.662718 | 16.228808 |
| H | 3.557225 | -3.080702 | 17.145929 |
| H | 4.831025 | -3.664537 | 16.058750 |
| H | 3.426708 | -4.696972 | 16.426231 |
| C | -1.956964 | 4.953090 | 12.742760 |
| H | -1.950511 | 6.022551 | 12.485470 |
| H | -3.006992 | 4.662198 | 12.903951 |
| C | 0.041645 | 5.598830 | 14.181105 |
| H | 0.583857 | 5.386753 | 15.110504 |
| H | -0.313358 | 6.639893 | 14.231415 |
| H | 0.747710 | 5.521407 | 13.345420 |
| C | -2.028238 | 4.779234 | 15.276824 |
| H | -1.496106 | 4.417151 | 16.168987 |
| H | -2.970213 | 4.226741 | 15.185761 |
| H | -2.276784 | 5.839526 | 15.437040 |
| Ge | 0.358699 | -0.544033 | 13.052653 |

**2** (S1-excited state)

| | | | |
|---|---|---|---|
| Si | 1.266879 | -2.163014 | 11.352144 |
| Si | -0.344112 | 1.424254 | 11.646736 |
| N | -0.844707 | 3.283026 | 13.765583 |
| N | 1.329428 | -4.490546 | 13.004507 |

| | | | |
|---|---|---|---|
| C | 1.314059 | -1.610846 | 8.509906 |
| C | -1.315831 | 4.222378 | 11.633393 |
| C | 2.031973 | -1.552252 | 9.729978 |
| C | -0.812121 | 2.985105 | 12.409075 |
| C | 1.121944 | 2.081873 | 9.242003 |
| C | -1.322917 | 1.539797 | 15.433754 |
| C | 1.261134 | 2.220713 | 7.855461 |
| H | 2.176480 | 2.669893 | 7.458599 |
| C | 2.383969 | -4.866903 | 10.911922 |
| C | 1.908607 | -1.133612 | 7.334128 |
| H | 1.344144 | -1.188072 | 6.398539 |
| C | -0.068096 | 1.519673 | 9.771437 |
| C | -0.881110 | 0.751980 | 16.504114 |
| H | -1.594186 | 0.106917 | 17.019473 |
| C | 1.901489 | -4.925921 | 9.452583 |
| H | 2.199972 | -4.036960 | 8.883991 |
| H | 0.806530 | -5.019995 | 9.403992 |
| H | 2.345558 | -5.804965 | 8.955471 |
| C | 3.331174 | -0.974178 | 9.737971 |
| C | -0.731763 | -3.846243 | 14.191187 |
| C | 3.903591 | -4.586923 | 10.908369 |
| H | 4.445568 | -5.413944 | 10.418838 |
| H | 4.305550 | -4.467007 | 11.924063 |
| H | 4.118351 | -3.665435 | 10.349431 |
| C | 2.271133 | 2.502032 | 10.125976 |
| H | 2.864235 | 1.620058 | 10.408960 |
| H | 1.928392 | 2.968479 | 11.059253 |
| H | 2.942618 | 3.202797 | 9.607377 |
| C | 0.448069 | 0.765850 | 16.913949 |
| H | 0.768283 | 0.151608 | 17.758782 |
| C | -1.058927 | 1.040192 | 8.876945 |
| C | 3.188793 | -0.577350 | 7.325511 |
| C | 0.806914 | -2.618121 | 16.195518 |
| H | 1.392645 | -2.114059 | 16.965687 |
| C | 3.883572 | -0.507724 | 8.539867 |
| H | 4.884950 | -0.066272 | 8.554026 |
| C | -0.065446 | -2.215779 | 8.401014 |
| H | -0.552393 | -2.313822 | 9.382017 |
| H | -0.014976 | -3.224266 | 7.958039 |
| H | -0.709093 | -1.601341 | 7.755438 |
| C | -1.635049 | -4.428705 | 13.106754 |
| H | -0.986608 | -4.870016 | 12.337181 |
| C | 0.686314 | -3.829670 | 14.099939 |
| C | -0.873878 | 1.191613 | 7.497133 |

| | | | |
|---|---|---|---|
| H | -1.650671 | 0.831804 | 6.815150 |
| C | 1.464157 | -3.161785 | 15.084683 |
| C | -0.575914 | -2.692858 | 16.329164 |
| H | -1.065067 | -2.270421 | 17.209612 |
| C | 1.640774 | -3.854633 | 11.810725 |
| C | 0.275627 | 1.785951 | 6.964922 |
| C | 2.045815 | -6.212788 | 11.597349 |
| H | 1.137629 | -6.625623 | 11.131104 |
| H | 2.845899 | -6.957150 | 11.468487 |
| C | -0.385278 | 2.387793 | 14.784561 |
| C | -1.335036 | -3.280658 | 15.322943 |
| H | -2.423165 | -3.298479 | 15.413050 |
| C | 0.988287 | 2.354380 | 15.148364 |
| C | 3.337186 | -1.477565 | 14.805177 |
| H | 2.849663 | -1.040220 | 13.924153 |
| H | 4.427110 | -1.360744 | 14.686414 |
| H | 3.026964 | -0.887306 | 15.681557 |
| C | 2.974031 | -2.964255 | 14.972882 |
| H | 3.311081 | -3.480977 | 14.066231 |
| C | -2.570724 | -5.524120 | 13.652557 |
| H | -2.030697 | -6.293849 | 14.221132 |
| H | -3.104359 | -6.019193 | 12.824876 |
| H | -3.334740 | -5.095637 | 14.321083 |
| C | -2.323036 | 0.377899 | 9.375443 |
| H | -2.093857 | -0.452438 | 10.060852 |
| H | -2.922594 | -0.016990 | 8.541544 |
| H | -2.951424 | 1.084523 | 9.940692 |
| C | -2.782774 | 1.420387 | 15.001735 |
| H | -2.958938 | 2.168318 | 14.216660 |
| C | 1.753468 | -5.923098 | 13.076419 |
| C | 1.373282 | 1.543439 | 16.225011 |
| H | 2.423033 | 1.518427 | 16.525138 |
| C | 0.645930 | -6.837253 | 13.614753 |
| H | 0.344110 | -6.543660 | 14.631178 |
| H | 1.015640 | -7.873462 | 13.657002 |
| H | -0.239489 | -6.823536 | 12.968481 |
| C | 2.993482 | -6.126778 | 13.966105 |
| H | 3.842929 | -5.509617 | 13.650036 |
| H | 3.306784 | -7.181553 | 13.926415 |
| H | 2.759965 | -5.885739 | 15.011079 |
| C | 3.780658 | -0.007459 | 6.061875 |
| H | 3.385755 | -0.508732 | 5.164667 |
| H | 4.878371 | -0.092354 | 6.049822 |
| H | 3.534169 | 1.065128 | 5.969953 |

| | | | |
|---|---|---|---|
| C | 0.477285 | 1.890977 | 5.474930 |
| H | -0.480180 | 1.962066 | 4.936348 |
| H | 1.004104 | 0.999030 | 5.092203 |
| H | 1.089798 | 2.766186 | 5.207995 |
| C | -0.149932 | 4.995899 | 10.975927 |
| H | 0.673537 | 5.183426 | 11.678673 |
| H | -0.504983 | 5.969205 | 10.596783 |
| H | 0.254824 | 4.431797 | 10.125457 |
| C | 4.127903 | -0.820052 | 11.014093 |
| H | 3.622656 | -0.134365 | 11.712609 |
| H | 5.134112 | -0.423301 | 10.811747 |
| H | 4.234135 | -1.773407 | 11.552741 |
| C | -3.078465 | 0.040470 | 14.386076 |
| H | -2.449043 | -0.154235 | 13.507016 |
| H | -4.134159 | -0.020010 | 14.073219 |
| H | -2.892495 | -0.767487 | 15.111213 |
| C | -2.363554 | 3.917858 | 10.547798 |
| H | -1.925897 | 3.392140 | 9.689813 |
| H | -2.794297 | 4.862667 | 10.174876 |
| H | -3.182228 | 3.304077 | 10.953218 |
| C | 2.076387 | 3.122007 | 14.402114 |
| H | 1.588892 | 3.705172 | 13.610930 |
| C | -3.760902 | 1.683714 | 16.161995 |
| H | -3.726930 | 0.868755 | 16.902994 |
| H | -4.795415 | 1.736026 | 15.785428 |
| H | -3.541644 | 2.620823 | 16.692663 |
| C | -1.318447 | 4.666427 | 14.082115 |
| C | 2.854350 | 4.086170 | 15.315646 |
| H | 2.193297 | 4.763427 | 15.873827 |
| H | 3.548151 | 4.701686 | 14.720044 |
| H | 3.458697 | 3.532909 | 16.052538 |
| C | -2.475153 | -3.335166 | 12.420650 |
| H | -3.162033 | -2.852845 | 13.135152 |
| H | -3.084245 | -3.774099 | 11.613122 |
| H | -1.838028 | -2.550157 | 11.990303 |
| C | 3.063762 | 2.161661 | 13.713847 |
| H | 3.623315 | 1.565196 | 14.452076 |
| H | 3.795654 | 2.728607 | 13.115746 |
| H | 2.536777 | 1.465126 | 13.048131 |
| C | 3.735121 | -3.541217 | 16.180225 |
| H | 3.528361 | -2.960357 | 17.093539 |
| H | 4.822331 | -3.497382 | 16.004683 |
| H | 3.466166 | -4.586426 | 16.387616 |
| C | -1.987688 | 5.051278 | 12.755415 |

| | | | |
|---|---|---|---|
| H | -1.936248 | 6.133210 | 12.561825 |
| H | -3.053383 | 4.778442 | 12.805850 |
| C | -0.155588 | 5.607398 | 14.448324 |
| H | 0.309321 | 5.289690 | 15.390611 |
| H | -0.536593 | 6.630774 | 14.589138 |
| H | 0.620013 | 5.637739 | 13.674115 |
| C | -2.319531 | 4.704646 | 15.243318 |
| H | -1.877581 | 4.299845 | 16.165857 |
| H | -3.231257 | 4.140695 | 15.014312 |
| H | -2.610849 | 5.748501 | 15.437269 |
| Ge | 0.296949 | -0.528434 | 12.650492 |

**2** (triplet)

| | | | |
|---|---|---|---|
| Si | 1.290355 | -1.967027 | 11.429953 |
| Si | -0.027196 | 1.304109 | 11.703895 |
| N | -0.602061 | 3.272752 | 13.719735 |
| N | 1.360085 | -4.423423 | 12.933853 |
| C | 1.465096 | -1.549815 | 8.568189 |
| C | -1.473862 | 4.003674 | 11.632713 |
| C | 2.133211 | -1.424339 | 9.810692 |
| C | -0.704184 | 2.893077 | 12.390802 |
| C | 0.987877 | 2.004477 | 9.112749 |
| C | -1.387295 | 1.631487 | 15.368277 |
| C | 0.951404 | 2.093088 | 7.718109 |
| H | 1.793618 | 2.554184 | 7.193373 |
| C | 2.541862 | -4.698199 | 10.907158 |
| C | 2.059915 | -1.051472 | 7.402647 |
| H | 1.526692 | -1.155555 | 6.452390 |
| C | -0.097783 | 1.411672 | 9.807269 |
| C | -1.110133 | 0.785827 | 16.448487 |
| H | -1.918378 | 0.198537 | 16.888416 |
| C | 2.204589 | -4.714665 | 9.407811 |
| H | 2.538612 | -3.806318 | 8.894950 |
| H | 1.122796 | -4.830418 | 9.248718 |
| H | 2.710688 | -5.572883 | 8.934414 |
| C | 3.399228 | -0.784296 | 9.840222 |
| C | -0.731721 | -3.816302 | 14.100053 |
| C | 4.046886 | -4.381543 | 11.062627 |
| H | 4.655744 | -5.159260 | 10.571199 |
| H | 4.351440 | -4.323544 | 12.117332 |
| H | 4.281673 | -3.418137 | 10.589217 |
| C | 2.183691 | 2.559833 | 9.852254 |
| H | 2.554904 | 1.846110 | 10.602651 |

| | | | |
|---|---|---|---|
| H | 1.931531 | 3.487839 | 10.390613 |
| H | 3.012628 | 2.779228 | 9.163566 |
| C | 0.176979 | 0.668713 | 16.965307 |
| H | 0.369712 | 0.002558 | 17.809500 |
| C | -1.175710 | 0.876853 | 9.064532 |
| C | 3.303488 | -0.416436 | 7.420789 |
| C | 0.766723 | -2.747271 | 16.224194 |
| H | 1.337966 | -2.299825 | 17.039543 |
| C | 3.960022 | -0.299214 | 8.651875 |
| H | 4.937677 | 0.192076 | 8.687571 |
| C | 0.131484 | -2.246701 | 8.424068 |
| H | -0.298781 | -2.532807 | 9.395913 |
| H | 0.234502 | -3.166055 | 7.825313 |
| H | -0.590580 | -1.597501 | 7.908864 |
| C | -1.616233 | -4.314704 | 12.958072 |
| H | -0.959058 | -4.736873 | 12.185134 |
| C | 0.687750 | -3.829523 | 14.058656 |
| C | -1.178487 | 0.993363 | 7.666361 |
| H | -2.025154 | 0.589789 | 7.101363 |
| C | 1.449385 | -3.255965 | 15.112092 |
| C | -0.621951 | -2.770206 | 16.296640 |
| H | -1.132683 | -2.360318 | 17.171250 |
| C | 1.713652 | -3.735812 | 11.785979 |
| C | -0.124375 | 1.596392 | 6.973232 |
| C | 2.178727 | -6.082407 | 11.492957 |
| H | 1.329526 | -6.492721 | 10.924689 |
| H | 3.006756 | -6.801329 | 11.402043 |
| C | -0.316671 | 2.374239 | 14.802296 |
| C | -1.358968 | -3.284865 | 15.235638 |
| H | -2.449933 | -3.262362 | 15.281406 |
| C | 1.001797 | 2.239076 | 15.298817 |
| C | 3.403597 | -1.639462 | 14.958184 |
| H | 2.989718 | -1.167754 | 14.058269 |
| H | 4.503392 | -1.568476 | 14.918416 |
| H | 3.055433 | -1.050519 | 15.821446 |
| C | 2.969940 | -3.111192 | 15.074232 |
| H | 3.330357 | -3.617970 | 14.172168 |
| C | -2.596829 | -5.410430 | 13.417788 |
| H | -2.097317 | -6.223694 | 13.962408 |
| H | -3.116092 | -5.847559 | 12.549263 |
| H | -3.370876 | -4.994893 | 14.082794 |
| C | -2.307388 | 0.132037 | 9.736968 |
| H | -2.002077 | -0.900817 | 9.970202 |
| H | -3.198795 | 0.082737 | 9.092693 |

| | | | |
|---|---|---|---|
| H | -2.601188 | 0.588057 | 10.693485 |
| C | -2.820112 | 1.702568 | 14.843721 |
| H | -2.870751 | 2.529744 | 14.123407 |
| C | 1.751145 | -5.874710 | 12.950735 |
| C | 1.220322 | 1.385052 | 16.389258 |
| H | 2.232743 | 1.268960 | 16.782308 |
| C | 0.575368 | -6.786622 | 13.323883 |
| H | 0.202729 | -6.564556 | 14.334481 |
| H | 0.910745 | -7.835093 | 13.307596 |
| H | -0.253695 | -6.688583 | 12.613132 |
| C | 2.893764 | -6.170522 | 13.937233 |
| H | 3.796972 | -5.586211 | 13.725220 |
| H | 3.158890 | -7.236893 | 13.872546 |
| H | 2.578828 | -5.965523 | 14.968259 |
| C | 3.889842 | 0.171338 | 6.162067 |
| H | 3.641966 | -0.434776 | 5.276407 |
| H | 4.985464 | 0.260504 | 6.222595 |
| H | 3.486483 | 1.183983 | 5.983108 |
| C | -0.107294 | 1.656232 | 5.466465 |
| H | -1.118869 | 1.571557 | 5.040723 |
| H | 0.495647 | 0.828655 | 5.051600 |
| H | 0.342063 | 2.593910 | 5.102714 |
| C | -0.629531 | 4.757174 | 10.583401 |
| H | 0.285643 | 5.186555 | 11.013482 |
| H | -1.220330 | 5.585570 | 10.158311 |
| H | -0.343552 | 4.093640 | 9.757971 |
| C | 4.150100 | -0.579745 | 11.137108 |
| H | 3.665361 | 0.192118 | 11.755734 |
| H | 5.189207 | -0.264902 | 10.956930 |
| H | 4.172277 | -1.491343 | 11.753945 |
| C | -3.219573 | 0.423099 | 14.087072 |
| H | -2.565487 | 0.248042 | 13.222861 |
| H | -4.261449 | 0.495743 | 13.732840 |
| H | -3.137825 | -0.463110 | 14.737141 |
| C | -2.733418 | 3.459789 | 10.928197 |
| H | -2.477352 | 2.871245 | 10.038773 |
| H | -3.368105 | 4.300720 | 10.599955 |
| H | -3.326773 | 2.829852 | 11.608913 |
| C | 2.197133 | 2.951762 | 14.678190 |
| H | 1.825429 | 3.534366 | 13.826314 |
| C | -3.835413 | 1.991116 | 15.964231 |
| H | -3.940915 | 1.129531 | 16.642559 |
| H | -4.830867 | 2.191290 | 15.535725 |
| H | -3.543557 | 2.858066 | 16.576604 |

| | | | |
|---|---|---|---|
| C | -0.951371 | 4.711982 | 13.951193 |
| C | 2.867706 | 3.912638 | 15.675561 |
| H | 2.144542 | 4.600665 | 16.139719 |
| H | 3.642601 | 4.515000 | 15.173799 |
| H | 3.356777 | 3.356434 | 16.491606 |
| C | -2.406506 | -3.168624 | 12.299524 |
| H | -3.095795 | -2.693869 | 13.016585 |
| H | -3.007478 | -3.555458 | 11.459820 |
| H | -1.737136 | -2.384362 | 11.920542 |
| C | 3.229160 | 1.957892 | 14.119905 |
| H | 3.696071 | 1.363137 | 14.920740 |
| H | 4.032572 | 2.494956 | 13.589413 |
| H | 2.750574 | 1.258645 | 13.420343 |
| C | 3.651934 | -3.751363 | 16.296869 |
| H | 3.437548 | -3.181063 | 17.214942 |
| H | 4.746286 | -3.757019 | 16.165651 |
| H | 3.324378 | -4.786481 | 16.469194 |
| C | -1.915993 | 4.949906 | 12.781964 |
| H | -1.940897 | 6.003985 | 12.467909 |
| H | -2.934759 | 4.682509 | 13.104044 |
| C | 0.287980 | 5.624822 | 13.848111 |
| H | 0.949165 | 5.499885 | 14.713579 |
| H | -0.032234 | 6.678178 | 13.821083 |
| H | 0.868230 | 5.421138 | 12.939067 |
| C | -1.611209 | 4.946476 | 15.312079 |
| H | -0.936895 | 4.674211 | 16.137590 |
| H | -2.538384 | 4.372441 | 15.421529 |
| H | -1.859273 | 6.014304 | 15.412282 |
| Ge | 0.322101 | -0.571287 | 13.066593 |

**3** (singlet)

| | | | |
|---|---|---|---|
| Sn | 0.260814 | -0.578397 | 13.207489 |
| Si | 1.263145 | -2.139412 | 11.400240 |
| Si | 0.051964 | 1.538976 | 11.725318 |
| N | -0.693737 | 3.419078 | 13.748631 |
| N | 1.455942 | -4.553406 | 12.942343 |
| C | 1.305586 | -1.644071 | 8.532369 |
| C | -1.303824 | 4.236084 | 11.612238 |
| C | 2.017060 | -1.539159 | 9.753053 |
| C | -0.667622 | 3.067146 | 12.411999 |
| C | 1.092424 | 2.088401 | 9.087978 |
| C | -1.620201 | 1.756234 | 15.292356 |
| C | 1.040531 | 2.135640 | 7.690299 |

| | | | |
|---|---|---|---|
| H | 1.885401 | 2.564857 | 7.143501 |
| C | 2.508805 | -4.816502 | 10.848280 |
| C | 1.869522 | -1.150502 | 7.348307 |
| H | 1.304653 | -1.247217 | 6.415772 |
| C | -0.001015 | 1.551807 | 9.817055 |
| C | -1.438677 | 0.873320 | 16.364482 |
| H | -2.292079 | 0.305147 | 16.740521 |
| C | 2.060034 | -4.836840 | 9.378340 |
| H | 2.350638 | -3.926965 | 8.842222 |
| H | 0.969124 | -4.953025 | 9.300593 |
| H | 2.532799 | -5.693613 | 8.869016 |
| C | 3.280234 | -0.887605 | 9.743520 |
| C | -0.583763 | -3.988329 | 14.214750 |
| C | 4.014603 | -4.472730 | 10.895012 |
| H | 4.600173 | -5.250427 | 10.376270 |
| H | 4.390582 | -4.390047 | 11.924689 |
| H | 4.200068 | -3.514277 | 10.390898 |
| C | 2.332143 | 2.607479 | 9.781830 |
| H | 2.748599 | 1.847998 | 10.459249 |
| H | 2.122565 | 3.497377 | 10.395270 |
| H | 3.113422 | 2.868862 | 9.052506 |
| C | -0.189472 | 0.697304 | 16.954470 |
| H | -0.069798 | 0.002713 | 17.789202 |
| C | -1.083953 | 0.990840 | 9.094664 |
| C | 3.121031 | -0.533934 | 7.325287 |
| C | 0.994108 | -2.853353 | 16.244338 |
| H | 1.594803 | -2.383508 | 17.025938 |
| C | 3.809088 | -0.409380 | 8.538202 |
| H | 4.786050 | 0.084495 | 8.545126 |
| C | -0.047817 | -2.308387 | 8.426820 |
| H | -0.458784 | -2.568940 | 9.413156 |
| H | 0.016132 | -3.238756 | 7.838382 |
| H | -0.763016 | -1.644932 | 7.918318 |
| C | -1.515611 | -4.527212 | 13.129424 |
| H | -0.890421 | -4.936632 | 12.324219 |
| C | 0.833043 | -3.963380 | 14.092691 |
| C | -1.097440 | 1.058190 | 7.693774 |
| H | -1.948910 | 0.637096 | 7.149429 |
| C | 1.630523 | -3.349658 | 15.097782 |
| C | -0.386418 | -2.926372 | 16.398911 |
| H | -0.860359 | -2.530410 | 17.300261 |
| C | 1.727105 | -3.859716 | 11.777837 |
| C | -0.048756 | 1.634643 | 6.971204 |
| C | 2.209763 | -6.203413 | 11.462895 |

| | | | |
|---|---|---|---|
| H | 1.326625 | -6.628598 | 10.960959 |
| H | 3.039496 | -6.910572 | 11.313923 |
| C | -0.496626 | 2.485465 | 14.816257 |
| C | -1.163661 | -3.473405 | 15.383677 |
| H | -2.250039 | -3.493745 | 15.494097 |
| C | 0.787796 | 2.281554 | 15.382169 |
| C | 3.535504 | -1.682660 | 14.852205 |
| H | 3.105389 | -1.233204 | 13.948140 |
| H | 4.631612 | -1.581576 | 14.790625 |
| H | 3.189606 | -1.097538 | 15.719150 |
| C | 3.142900 | -3.165207 | 14.981573 |
| H | 3.469806 | -3.660014 | 14.060198 |
| C | -2.433363 | -5.646749 | 13.657875 |
| H | -1.876660 | -6.431220 | 14.188576 |
| H | -2.979979 | -6.117848 | 12.824832 |
| H | -3.186737 | -5.247171 | 14.355611 |
| C | -2.220353 | 0.282149 | 9.797103 |
| H | -1.850806 | -0.617851 | 10.316193 |
| H | -2.997885 | -0.034656 | 9.085510 |
| H | -2.693355 | 0.912705 | 10.564068 |
| C | -3.012748 | 1.888621 | 14.679560 |
| H | -2.987201 | 2.731000 | 13.975603 |
| C | 1.885634 | -5.992697 | 12.946382 |
| C | 0.910125 | 1.389081 | 16.459204 |
| H | 1.892073 | 1.228547 | 16.910097 |
| C | 0.768474 | -6.933298 | 13.414948 |
| H | 0.457853 | -6.703850 | 14.445057 |
| H | 1.134705 | -7.971320 | 13.394463 |
| H | -0.110529 | -6.875772 | 12.762123 |
| C | 3.107119 | -6.244584 | 13.847229 |
| H | 3.972005 | -5.633359 | 13.563241 |
| H | 3.402371 | -7.302385 | 13.772021 |
| H | 2.863030 | -6.039559 | 14.897277 |
| C | 3.683914 | 0.033405 | 6.046692 |
| H | 3.363758 | -0.548814 | 5.168358 |
| H | 4.784586 | 0.059579 | 6.060018 |
| H | 3.332809 | 1.069150 | 5.893174 |
| C | -0.047910 | 1.648652 | 5.463407 |
| H | -1.065547 | 1.565892 | 5.051824 |
| H | 0.538226 | 0.800109 | 5.067416 |
| H | 0.410505 | 2.568202 | 5.066114 |
| C | -0.310409 | 4.957679 | 10.679566 |
| H | 0.595361 | 5.284300 | 11.208259 |
| H | -0.789585 | 5.850098 | 10.243640 |

| | | | |
|---|---|---|---|
| H | -0.007568 | 4.303957 | 9.852226 |
| C | 4.067599 | -0.661484 | 11.016167 |
| H | 3.535202 | 0.029758 | 11.690799 |
| H | 5.056628 | -0.227961 | 10.803265 |
| H | 4.212817 | -1.590078 | 11.588294 |
| C | -3.406288 | 0.642076 | 13.866234 |
| H | -2.714614 | 0.485511 | 13.028070 |
| H | -4.422955 | 0.757485 | 13.455230 |
| H | -3.392688 | -0.264671 | 14.493151 |
| C | -2.505345 | 3.768557 | 10.766957 |
| H | -2.186611 | 3.164875 | 9.908596 |
| H | -3.048131 | 4.647647 | 10.379185 |
| H | -3.206844 | 3.174674 | 11.373133 |
| C | 2.040500 | 2.993480 | 14.883301 |
| H | 1.748311 | 3.621608 | 14.033486 |
| C | -4.086397 | 2.188210 | 15.741017 |
| H | -4.269380 | 1.314515 | 16.386947 |
| H | -5.043556 | 2.440739 | 15.256723 |
| H | -3.799278 | 3.026113 | 16.394239 |
| C | -0.999626 | 4.865768 | 13.989323 |
| C | 2.639707 | 3.892200 | 15.981563 |
| H | 1.884931 | 4.553063 | 16.433972 |
| H | 3.447554 | 4.520901 | 15.573233 |
| H | 3.069841 | 3.286280 | 16.795374 |
| C | -2.380774 | -3.416339 | 12.504485 |
| H | -3.016531 | -2.926547 | 13.260174 |
| H | -3.042634 | -3.839349 | 11.730934 |
| H | -1.758296 | -2.645881 | 12.030180 |
| C | 3.112486 | 2.019702 | 14.364717 |
| H | 3.448827 | 1.324530 | 15.149293 |
| H | 3.994733 | 2.578969 | 14.012696 |
| H | 2.731309 | 1.428864 | 13.520388 |
| C | 3.900695 | -3.789456 | 16.167960 |
| H | 3.724429 | -3.221585 | 17.095583 |
| H | 4.986316 | -3.776128 | 15.978704 |
| H | 3.601057 | -4.829724 | 16.357623 |
| C | -1.820416 | 5.178388 | 12.732323 |
| H | -1.762635 | 6.238880 | 12.445762 |
| H | -2.879358 | 4.954187 | 12.936435 |
| C | 0.276577 | 5.731166 | 14.049084 |
| H | 0.837253 | 5.554247 | 14.974320 |
| H | -0.000586 | 6.796714 | 14.025795 |
| H | 0.942308 | 5.533721 | 13.199433 |
| C | -1.790930 | 5.090923 | 15.280122 |

| | | | |
|---|---|---|---|
| H | -1.228436 | 4.746596 | 16.160901 |
| H | -2.757560 | 4.573893 | 15.265617 |
| H | -1.986039 | 6.167489 | 15.401543 |

**3** (S1-excited state)

| | | | |
|---|---|---|---|
| Sn | -0.658134 | -1.114488 | 12.512174 |
| Si | 1.708071 | -2.018914 | 11.868206 |
| Si | -0.538240 | 1.531572 | 11.685128 |
| N | -0.321849 | 3.644635 | 13.586077 |
| N | 1.662861 | -4.612206 | 13.076339 |
| C | 1.820883 | -1.269714 | 9.016467 |
| C | -1.187138 | 4.526119 | 11.572938 |
| C | 2.502639 | -1.294152 | 10.268797 |
| C | -0.602505 | 3.283684 | 12.300974 |
| C | -0.007733 | 1.782127 | 8.896936 |
| C | -1.297843 | 2.282370 | 15.373843 |
| C | -0.283137 | 1.617747 | 7.533553 |
| H | 0.516530 | 1.799529 | 6.808976 |
| C | 2.912666 | -4.659846 | 11.055901 |
| C | 2.442573 | -0.711158 | 7.894062 |
| H | 1.905680 | -0.712647 | 6.940549 |
| C | -1.035506 | 1.553085 | 9.849198 |
| C | -1.115351 | 1.445010 | 16.479251 |
| H | -1.987115 | 1.089892 | 17.032109 |
| C | 2.279899 | -4.752160 | 9.652347 |
| H | 2.374005 | -3.805664 | 9.105915 |
| H | 1.212445 | -5.012735 | 9.714515 |
| H | 2.789482 | -5.533668 | 9.062055 |
| C | 3.795380 | -0.711040 | 10.330786 |
| C | -0.764803 | -4.708314 | 13.426582 |
| C | 4.368981 | -4.182881 | 10.889427 |
| H | 4.943905 | -4.925762 | 10.311240 |
| H | 4.871756 | -4.038542 | 11.855226 |
| H | 4.405040 | -3.234754 | 10.339675 |
| C | 1.399929 | 2.137121 | 9.325932 |
| H | 1.830250 | 1.338100 | 9.952089 |
| H | 1.446204 | 3.067466 | 9.908376 |
| H | 2.065970 | 2.248636 | 8.459575 |
| C | 0.156589 | 1.049676 | 16.883975 |
| H | 0.274988 | 0.400752 | 17.754570 |
| C | -2.299018 | 1.102698 | 9.397083 |
| C | 3.713943 | -0.128391 | 7.954451 |
| C | -0.472719 | -3.405358 | 15.901497 |

| | | | |
|---|---|---|---|
| H | -0.367837 | -2.894298 | 16.861491 |
| C | 4.369867 | -0.134645 | 9.184981 |
| H | 5.367525 | 0.310634 | 9.260813 |
| C | 0.418189 | -1.802310 | 8.837708 |
| H | 0.255413 | -2.734996 | 9.391931 |
| H | 0.196184 | -1.982834 | 7.775045 |
| H | -0.320804 | -1.075479 | 9.209905 |
| C | -0.974233 | -5.454239 | 12.112705 |
| H | 0.019912 | -5.698809 | 11.718728 |
| C | 0.525411 | -4.331121 | 13.891401 |
| C | -2.532090 | 0.942295 | 8.025873 |
| H | -3.515510 | 0.597641 | 7.690478 |
| C | 0.671989 | -3.671615 | 15.136664 |
| C | -1.740075 | -3.760510 | 15.448874 |
| H | -2.623230 | -3.531785 | 16.050929 |
| C | 2.063427 | -3.784058 | 12.022116 |
| C | -1.537781 | 1.200866 | 7.074352 |
| C | 2.853845 | -6.063853 | 11.721056 |
| H | 2.052386 | -6.658029 | 11.254621 |
| H | 3.790783 | -6.625885 | 11.590586 |
| C | -0.142695 | 2.718154 | 14.672295 |
| C | -1.879273 | -4.403673 | 14.219977 |
| H | -2.876753 | -4.678559 | 13.869349 |
| C | 1.150177 | 2.299373 | 15.048170 |
| C | 2.112219 | -1.745501 | 15.948387 |
| H | 1.818988 | -1.163644 | 15.063888 |
| H | 3.140442 | -1.460034 | 16.228067 |
| H | 1.450618 | -1.455214 | 16.778186 |
| C | 2.034161 | -3.250208 | 15.666553 |
| H | 2.765309 | -3.461674 | 14.878603 |
| C | -1.738237 | -6.774161 | 12.320655 |
| H | -1.281850 | -7.395405 | 13.106312 |
| H | -1.754313 | -7.358679 | 11.386235 |
| H | -2.785516 | -6.592596 | 12.611400 |
| C | -3.384040 | 0.758693 | 10.388982 |
| H | -3.041612 | -0.033817 | 11.082001 |
| H | -4.288376 | 0.377547 | 9.892625 |
| H | -3.677889 | 1.626700 | 10.999030 |
| C | -2.720034 | 2.665853 | 14.970184 |
| H | -2.655157 | 3.459682 | 14.213659 |
| C | 2.502184 | -5.837514 | 13.199002 |
| C | 1.274078 | 1.465664 | 16.167489 |
| H | 2.264002 | 1.122655 | 16.471858 |
| C | 1.732229 | -7.008440 | 13.811341 |

| | | | |
|---|---|---|---|
| H | 1.397334 | -6.773455 | 14.833271 |
| H | 2.387052 | -7.891934 | 13.862558 |
| H | 0.850229 | -7.273225 | 13.215751 |
| C | 3.787001 | -5.606505 | 14.027270 |
| H | 4.294028 | -4.679260 | 13.728983 |
| H | 4.485732 | -6.443472 | 13.870450 |
| H | 3.574225 | -5.552173 | 15.101377 |
| C | 4.321445 | 0.519606 | 6.735283 |
| H | 4.164936 | -0.092758 | 5.832607 |
| H | 5.403275 | 0.683674 | 6.854615 |
| H | 3.858039 | 1.503262 | 6.539003 |
| C | -1.789125 | 0.980518 | 5.602934 |
| H | -2.857602 | 1.072652 | 5.353901 |
| H | -1.467912 | -0.030820 | 5.296604 |
| H | -1.229361 | 1.699171 | 4.983852 |
| C | -0.312377 | 5.028627 | 10.408028 |
| H | 0.730227 | 5.201091 | 10.709952 |
| H | -0.716520 | 5.982230 | 10.031694 |
| H | -0.320650 | 4.312110 | 9.577822 |
| C | 4.643316 | -0.725205 | 11.584456 |
| H | 4.050712 | -0.939234 | 12.484365 |
| H | 5.164529 | 0.234550 | 11.723447 |
| H | 5.417635 | -1.507708 | 11.519218 |
| C | -3.463161 | 1.484898 | 14.321901 |
| H | -2.942362 | 1.122236 | 13.427599 |
| H | -4.483765 | 1.784885 | 14.032417 |
| H | -3.539998 | 0.635794 | 15.019915 |
| C | -2.606182 | 4.264222 | 11.029997 |
| H | -2.595197 | 3.585313 | 10.169222 |
| H | -3.049700 | 5.219338 | 10.702508 |
| H | -3.257626 | 3.839018 | 11.808940 |
| C | 2.396613 | 2.676753 | 14.261297 |
| H | 2.093981 | 3.367869 | 13.466167 |
| C | -3.538240 | 3.214064 | 16.153257 |
| H | -3.772742 | 2.422505 | 16.882106 |
| H | -4.497917 | 3.621171 | 15.796369 |
| H | -3.005800 | 4.011191 | 16.693739 |
| C | -0.317929 | 5.132004 | 13.813844 |
| C | 3.443176 | 3.383164 | 15.138734 |
| H | 3.015860 | 4.237605 | 15.686524 |
| H | 4.278088 | 3.752573 | 14.521764 |
| H | 3.866844 | 2.693839 | 15.886317 |
| C | -1.679044 | -4.594185 | 11.052205 |
| H | -2.691504 | -4.305373 | 11.378741 |

| | | | |
|---|---|---|---|
| H | -1.774803 | -5.152257 | 10.105935 |
| H | -1.118073 | -3.671330 | 10.852517 |
| C | 3.011917 | 1.454975 | 13.567173 |
| H | 3.366997 | 0.709263 | 14.295372 |
| H | 3.870597 | 1.760878 | 12.951101 |
| H | 2.288265 | 0.947926 | 12.913232 |
| C | 2.409590 | -4.052474 | 16.924842 |
| H | 1.748755 | -3.791975 | 17.768061 |
| H | 3.446118 | -3.837271 | 17.233029 |
| H | 2.317983 | -5.137383 | 16.763344 |
| C | -1.282294 | 5.578976 | 12.710936 |
| H | -1.062431 | 6.595726 | 12.355312 |
| H | -2.305926 | 5.590757 | 13.115807 |
| C | 1.091983 | 5.716591 | 13.596899 |
| H | 1.776128 | 5.422160 | 14.401860 |
| H | 1.030522 | 6.815635 | 13.594522 |
| H | 1.523490 | 5.396740 | 12.639451 |
| C | -0.791992 | 5.521119 | 15.214342 |
| H | -0.144018 | 5.091901 | 15.992363 |
| H | -1.821832 | 5.200576 | 15.405692 |
| H | -0.757854 | 6.617047 | 15.307603 |

**3** (triplet)

| | | | |
|---|---|---|---|
| Sn | -0.246168 | -0.750389 | 13.178279 |
| Si | 1.111817 | -2.171898 | 11.477547 |
| Si | -0.264257 | 1.418174 | 11.745738 |
| N | -0.661198 | 3.494063 | 13.700463 |
| N | 1.535509 | -4.601308 | 12.970964 |
| C | 1.214472 | -1.579470 | 8.636243 |
| C | -1.364596 | 4.279038 | 11.574363 |
| C | 1.912741 | -1.531854 | 9.867266 |
| C | -0.750109 | 3.104956 | 12.376179 |
| C | 1.040517 | 2.076625 | 9.269692 |
| C | -1.537170 | 1.956800 | 15.399633 |
| C | 1.134573 | 2.203086 | 7.880341 |
| H | 2.047061 | 2.623513 | 7.447244 |
| C | 2.449358 | -4.808432 | 10.792760 |
| C | 1.819767 | -1.090241 | 7.470483 |
| H | 1.267465 | -1.144627 | 6.527229 |
| C | -0.140464 | 1.546347 | 9.849904 |
| C | -1.301728 | 1.078059 | 16.464978 |
| H | -2.145736 | 0.569111 | 16.935039 |
| C | 1.795438 | -4.881151 | 9.400480 |

| | | | |
|---|---|---|---|
| H | 1.976994 | -3.972056 | 8.816702 |
| H | 0.709114 | -5.038088 | 9.479496 |
| H | 2.225578 | -5.728618 | 8.839954 |
| C | 3.201392 | -0.934548 | 9.893261 |
| C | -0.642270 | -4.343471 | 14.087921 |
| C | 3.927151 | -4.401539 | 10.602752 |
| H | 4.458381 | -5.169189 | 10.015229 |
| H | 4.454853 | -4.279624 | 11.558511 |
| H | 3.994608 | -3.452915 | 10.054046 |
| C | 2.208314 | 2.506059 | 10.128385 |
| H | 2.577966 | 1.664425 | 10.732431 |
| H | 1.928930 | 3.306962 | 10.830968 |
| H | 3.047332 | 2.867404 | 9.515607 |
| C | -0.011373 | 0.828281 | 16.924831 |
| H | 0.150239 | 0.133573 | 17.752798 |
| C | -1.172561 | 1.085415 | 8.997837 |
| C | 3.096655 | -0.527198 | 7.480964 |
| C | 0.601207 | -3.023997 | 16.236060 |
| H | 1.070382 | -2.488586 | 17.064298 |
| C | 3.768205 | -0.451509 | 8.707976 |
| H | 4.764056 | 0.002026 | 8.740585 |
| C | -0.165516 | -2.182800 | 8.507109 |
| H | -0.637429 | -2.342598 | 9.488191 |
| H | -0.126168 | -3.160529 | 7.999142 |
| H | -0.819870 | -1.527634 | 7.914248 |
| C | -1.385412 | -5.037677 | 12.947508 |
| H | -0.633060 | -5.390609 | 12.228954 |
| C | 0.762152 | -4.122813 | 14.081639 |
| C | -1.039221 | 1.231019 | 7.609193 |
| H | -1.849728 | 0.884793 | 6.959856 |
| C | 1.390081 | -3.435937 | 15.152312 |
| C | -0.770006 | -3.255215 | 16.263991 |
| H | -1.367268 | -2.913905 | 17.113249 |
| C | 1.718753 | -3.883450 | 11.796675 |
| C | 0.102592 | 1.792663 | 7.029442 |
| C | 2.313697 | -6.197669 | 11.462803 |
| H | 1.398496 | -6.682519 | 11.087661 |
| H | 3.156990 | -6.862253 | 11.222165 |
| C | -0.421391 | 2.588046 | 14.788488 |
| C | -1.382224 | -3.899451 | 15.192323 |
| H | -2.462920 | -4.055953 | 15.208162 |
| C | 0.897046 | 2.329030 | 15.235355 |
| C | 3.127307 | -1.582114 | 15.119828 |
| H | 2.681694 | -1.134902 | 14.222058 |

| | | | |
|---|---|---|---|
| H | 4.210157 | -1.373413 | 15.112553 |
| H | 2.689279 | -1.071158 | 15.991552 |
| C | 2.877183 | -3.097413 | 15.169677 |
| H | 3.324140 | -3.517466 | 14.261786 |
| C | -2.190413 | -6.253652 | 13.444936 |
| H | -1.583074 | -6.933790 | 14.059740 |
| H | -2.588820 | -6.824553 | 12.590318 |
| H | -3.051448 | -5.938597 | 14.055958 |
| C | -2.410197 | 0.412198 | 9.549040 |
| H | -2.142415 | -0.494651 | 10.114231 |
| H | -3.103031 | 0.123067 | 8.744241 |
| H | -2.960627 | 1.060590 | 10.248672 |
| C | -2.975685 | 2.191420 | 14.941995 |
| H | -2.961650 | 3.013135 | 14.213835 |
| C | 2.162961 | -5.964574 | 12.969750 |
| C | 1.072961 | 1.450080 | 16.313281 |
| H | 2.083842 | 1.236362 | 16.666625 |
| C | 1.273896 | -7.025707 | 13.627241 |
| H | 1.048065 | -6.769720 | 14.673239 |
| H | 1.800462 | -7.992415 | 13.620936 |
| H | 0.329174 | -7.157431 | 13.088692 |
| C | 3.526500 | -5.991581 | 13.688727 |
| H | 4.209189 | -5.217128 | 13.318970 |
| H | 4.004509 | -6.969920 | 13.524654 |
| H | 3.405480 | -5.859707 | 14.770738 |
| C | 3.708682 | 0.037205 | 6.223593 |
| H | 3.356401 | -0.494957 | 5.326132 |
| H | 4.808348 | -0.015705 | 6.245194 |
| H | 3.434801 | 1.099724 | 6.097999 |
| C | 0.254904 | 1.888188 | 5.532436 |
| H | -0.719617 | 1.885957 | 5.020577 |
| H | 0.833029 | 1.030384 | 5.145338 |
| H | 0.797017 | 2.800337 | 5.236567 |
| C | -0.388993 | 4.940654 | 10.577328 |
| H | 0.541935 | 5.268657 | 11.058595 |
| H | -0.864608 | 5.827901 | 10.127261 |
| H | -0.128945 | 4.251989 | 9.764528 |
| C | 3.976203 | -0.793851 | 11.184942 |
| H | 3.422090 | -0.185158 | 11.916257 |
| H | 4.954494 | -0.317779 | 11.018959 |
| H | 4.147373 | -1.767404 | 11.669556 |
| C | -3.571285 | 0.971909 | 14.219272 |
| H | -2.997440 | 0.715470 | 13.318023 |
| H | -4.612544 | 1.178638 | 13.920559 |

| | | | |
|---|---|---|---|
| H | -3.573543 | 0.083119 | 14.870879 |
| C | -2.623708 | 3.850545 | 10.795837 |
| H | -2.367415 | 3.227370 | 9.930044 |
| H | -3.151478 | 4.743575 | 10.419625 |
| H | -3.317441 | 3.289759 | 11.441933 |
| C | 2.129393 | 2.941472 | 14.579949 |
| H | 1.783298 | 3.571066 | 13.751871 |
| C | -3.887480 | 2.608054 | 16.110893 |
| H | -4.060954 | 1.769828 | 16.804535 |
| H | -4.872591 | 2.926528 | 15.732974 |
| H | -3.458797 | 3.435814 | 16.695419 |
| C | -0.890352 | 4.962709 | 13.909343 |
| C | 2.916770 | 3.821423 | 15.566944 |
| H | 2.274126 | 4.565119 | 16.062242 |
| H | 3.727435 | 4.358524 | 15.047932 |
| H | 3.377959 | 3.210173 | 16.359330 |
| C | -2.317610 | -4.077834 | 12.186932 |
| H | -3.095337 | -3.666690 | 12.851131 |
| H | -2.823250 | -4.611197 | 11.364772 |
| H | -1.761369 | -3.231679 | 11.760962 |
| C | 3.051144 | 1.873788 | 13.970237 |
| H | 3.503922 | 1.237676 | 14.746120 |
| H | 3.870374 | 2.349468 | 13.406767 |
| H | 2.491210 | 1.221480 | 13.285703 |
| C | 3.586302 | -3.705724 | 16.393047 |
| H | 3.273451 | -3.203080 | 17.322530 |
| H | 4.678682 | -3.586500 | 16.306637 |
| H | 3.366527 | -4.777236 | 16.513006 |
| C | -1.776200 | 5.270673 | 12.695354 |
| H | -1.697462 | 6.320077 | 12.375016 |
| H | -2.827527 | 5.091475 | 12.970876 |
| C | 0.431998 | 5.755970 | 13.866531 |
| H | 1.029207 | 5.574991 | 14.768385 |
| H | 0.211690 | 6.833865 | 13.821545 |
| H | 1.040871 | 5.495853 | 12.991859 |
| C | -1.583179 | 5.277781 | 15.237868 |
| H | -1.000554 | 4.903371 | 16.092777 |
| H | -2.590324 | 4.850318 | 15.290911 |
| H | -1.676814 | 6.369406 | 15.342696 |

**4** (singlet)

| | | | |
|---|---|---|---|
| Pb | -0.040657 | -0.893705 | -0.201707 |
| Si | 2.032095 | 0.822423 | -0.136999 |

| | | | |
|---|---|---|---|
| Si | -1.992379 | 0.910104 | 0.145856 |
| N | 4.101303 | -1.053628 | 0.448440 |
| N | -4.265159 | -0.777943 | -0.282718 |
| C | 3.726788 | 0.173939 | -0.083346 |
| C | -3.750899 | 0.455465 | 0.084581 |
| C | 1.958159 | 2.560431 | -0.936058 |
| C | -1.715414 | 2.624087 | 0.940110 |
| C | 1.808555 | 2.739888 | -2.336772 |
| C | 1.745345 | 4.032790 | -2.873772 |
| H | 1.642456 | 4.153651 | -3.956925 |
| C | 1.795996 | 5.173595 | -2.065374 |
| C | 1.897278 | 4.988598 | -0.683821 |
| H | 1.909856 | 5.865038 | -0.028603 |
| C | 1.963221 | 3.712504 | -0.107810 |
| C | -1.479654 | 3.764104 | 0.128470 |
| C | -1.214388 | 5.002985 | 0.724002 |
| H | -1.028892 | 5.868979 | 0.081789 |
| C | -1.156430 | 5.160178 | 2.112244 |
| C | -1.369924 | 4.029697 | 2.907255 |
| H | -1.313989 | 4.127778 | 3.996231 |
| C | -1.630098 | 2.768974 | 2.349943 |
| C | 1.676939 | 1.565649 | -3.279916 |
| H | 2.500389 | 0.847199 | -3.165194 |
| H | 1.652445 | 1.894519 | -4.329935 |
| H | 0.746874 | 1.005679 | -3.083024 |
| C | 1.680044 | 6.556180 | -2.658320 |
| H | 0.624621 | 6.881009 | -2.697701 |
| H | 2.068865 | 6.591722 | -3.687893 |
| H | 2.225660 | 7.301997 | -2.059241 |
| C | 1.994527 | 3.601020 | 1.399092 |
| H | 1.071210 | 3.124966 | 1.764660 |
| H | 2.063689 | 4.591582 | 1.872954 |
| H | 2.836757 | 2.986862 | 1.751892 |
| C | -1.514067 | 3.677686 | -1.378548 |
| H | -0.897768 | 2.842538 | -1.742097 |
| H | -1.129617 | 4.597712 | -1.841259 |
| H | -2.537672 | 3.506443 | -1.747790 |
| C | -0.807187 | 6.494440 | 2.722744 |
| H | -1.140254 | 6.566376 | 3.769606 |
| H | -1.256699 | 7.328499 | 2.160766 |
| H | 0.286450 | 6.651859 | 2.714536 |
| C | -1.748808 | 1.586984 | 3.287199 |
| H | -2.538449 | 0.886532 | 2.984177 |
| H | -1.948089 | 1.911665 | 4.320164 |

| | | | |
|---|---|---|---|
| H | -0.809301 | 1.010020 | 3.297149 |
| C | 5.025445 | 0.920857 | -0.485175 |
| C | 6.129523 | -0.138616 | -0.222977 |
| H | 6.353235 | -0.672112 | -1.160313 |
| H | 7.069748 | 0.315670 | 0.123372 |
| C | 5.853775 | -0.684615 | 2.235565 |
| H | 5.365828 | 0.270689 | 2.468837 |
| H | 6.939695 | -0.552439 | 2.362968 |
| H | 5.524360 | -1.428963 | 2.969675 |
| C | 5.555299 | -1.138745 | 0.790723 |
| C | 6.113299 | -2.551372 | 0.601091 |
| H | 5.609800 | -3.273946 | 1.261150 |
| H | 7.186116 | -2.555505 | 0.847716 |
| H | 6.003316 | -2.897304 | -0.433789 |
| C | 5.263447 | 2.207140 | 0.331355 |
| H | 4.508436 | 2.965553 | 0.087491 |
| H | 6.253655 | 2.627481 | 0.088387 |
| H | 5.226545 | 2.025828 | 1.414205 |
| C | 5.040459 | 1.294024 | -1.981001 |
| H | 4.809626 | 0.419956 | -2.609409 |
| H | 6.043693 | 1.658530 | -2.261206 |
| H | 4.318428 | 2.088561 | -2.206780 |
| C | -4.951262 | 1.375003 | 0.403313 |
| C | -4.864069 | 2.792693 | -0.184137 |
| H | -4.085568 | 3.394118 | 0.300162 |
| H | -5.828634 | 3.307805 | -0.038490 |
| H | -4.657370 | 2.757397 | -1.264242 |
| C | -5.144027 | 1.492262 | 1.932288 |
| H | -5.125764 | 0.512577 | 2.430680 |
| H | -6.109689 | 1.974716 | 2.159009 |
| H | -4.347132 | 2.107545 | 2.370531 |
| C | -6.131013 | 0.633392 | -0.269240 |
| H | -6.254933 | 1.020835 | -1.292566 |
| H | -7.082902 | 0.799269 | 0.257367 |
| C | -5.761750 | -0.853935 | -0.342962 |
| C | -6.354633 | -1.661374 | 0.825310 |
| H | -6.023969 | -2.707019 | 0.777779 |
| H | -7.453219 | -1.652803 | 0.756119 |
| H | -6.076761 | -1.251855 | 1.803701 |
| C | -6.269482 | -1.490609 | -1.643140 |
| H | -5.961462 | -0.916316 | -2.524974 |
| H | -7.369968 | -1.517049 | -1.628547 |
| H | -5.908716 | -2.524279 | -1.752058 |
| C | -3.450564 | -1.929339 | -0.540522 |

| | | | |
|---|---|---|---|
| C | -3.076050 | -2.793924 | 0.525708 |
| C | -2.345601 | -3.952097 | 0.222677 |
| H | -2.056706 | -4.626749 | 1.031423 |
| C | -1.962831 | -4.251440 | -1.081574 |
| H | -1.390867 | -5.158591 | -1.291550 |
| C | -2.279923 | -3.369318 | -2.109414 |
| H | -1.945340 | -3.589119 | -3.125625 |
| C | -3.009513 | -2.194851 | -1.867023 |
| C | -3.271213 | -1.254793 | -3.043578 |
| H | -3.909171 | -0.438372 | -2.680249 |
| C | -1.975554 | -0.607188 | -3.569467 |
| H | -1.521072 | 0.041349 | -2.808013 |
| H | -2.191189 | 0.016291 | -4.452540 |
| H | -1.235414 | -1.368098 | -3.867246 |
| C | -3.990804 | -1.969165 | -4.203874 |
| H | -3.322135 | -2.689537 | -4.701746 |
| H | -4.306416 | -1.238131 | -4.965682 |
| H | -4.880133 | -2.520907 | -3.869431 |
| C | -3.388537 | -2.507266 | 1.993161 |
| H | -3.998347 | -1.597209 | 2.033096 |
| C | -2.111721 | -2.220249 | 2.804326 |
| H | -1.415931 | -3.074216 | 2.779012 |
| H | -2.364560 | -2.020473 | 3.858478 |
| H | -1.584597 | -1.340465 | 2.415096 |
| C | -4.170217 | -3.652852 | 2.661698 |
| H | -5.059833 | -3.946960 | 2.087101 |
| H | -4.499528 | -3.354109 | 3.670105 |
| H | -3.541065 | -4.550239 | 2.776542 |
| C | 3.203619 | -2.158652 | 0.589164 |
| C | 2.977587 | -3.001746 | -0.534167 |
| C | 2.129634 | -4.107364 | -0.386889 |
| H | 1.949025 | -4.760220 | -1.243671 |
| C | 1.508804 | -4.389782 | 0.828245 |
| H | 0.852018 | -5.258092 | 0.921528 |
| C | 1.711129 | -3.545141 | 1.914109 |
| H | 1.207222 | -3.756143 | 2.859962 |
| C | 2.541560 | -2.415726 | 1.818382 |
| C | 2.701505 | -1.527584 | 3.046048 |
| H | 3.344289 | -0.686564 | 2.760719 |
| C | 3.368415 | -2.295904 | 4.202362 |
| H | 2.692208 | -3.066216 | 4.607634 |
| H | 3.624660 | -1.611605 | 5.027304 |
| H | 4.288574 | -2.807440 | 3.881932 |
| C | 1.369200 | -0.921913 | 3.517461 |

| | | | |
|---|---|---|---|
| H | 0.925012 | -0.297355 | 2.729424 |
| H | 1.531843 | -0.280249 | 4.398700 |
| H | 0.638699 | -1.696423 | 3.799160 |
| C | 3.614597 | -2.746250 | -1.896750 |
| H | 4.339951 | -1.931002 | -1.774438 |
| C | 4.370303 | -3.977144 | -2.427610 |
| H | 5.082212 | -4.374969 | -1.688578 |
| H | 4.931640 | -3.717534 | -3.339816 |
| H | 3.679468 | -4.793811 | -2.691687 |
| C | 2.575522 | -2.274527 | -2.928859 |
| H | 3.058112 | -2.077365 | -3.900291 |
| H | 2.087500 | -1.349330 | -2.597137 |
| H | 1.794624 | -3.036879 | -3.086253 |

**4** (S1-excited state)

| | | | |
|---|---|---|---|
| Pb | -0.329640 | -0.537664 | -1.332277 |
| Si | 2.219446 | 0.630659 | -0.562669 |
| Si | -1.996144 | 0.512379 | 0.527458 |
| N | 4.236398 | -0.882297 | 0.750852 |
| N | -4.394968 | -0.910361 | -0.090388 |
| C | 3.926279 | 0.264380 | 0.082017 |
| C | -3.771466 | 0.274540 | 0.325402 |
| C | 2.290836 | 2.342262 | -1.392653 |
| C | -1.598619 | 2.302749 | 1.127633 |
| C | 2.232050 | 2.490836 | -2.798717 |
| C | 2.056626 | 3.760798 | -3.361244 |
| H | 2.016044 | 3.859939 | -4.450787 |
| C | 1.920924 | 4.904596 | -2.564721 |
| C | 1.950442 | 4.743430 | -1.174428 |
| H | 1.817763 | 5.620305 | -0.533413 |
| C | 2.112340 | 3.488247 | -0.574519 |
| C | -1.383874 | 3.388419 | 0.228700 |
| C | -1.073741 | 4.658602 | 0.727131 |
| H | -0.924724 | 5.479428 | 0.018961 |
| C | -0.918314 | 4.907858 | 2.095760 |
| C | -1.105214 | 3.839601 | 2.972392 |
| H | -0.992699 | 4.006468 | 4.048895 |
| C | -1.450591 | 2.555449 | 2.516956 |
| C | 2.318722 | 1.275261 | -3.690306 |
| H | 3.289228 | 0.762785 | -3.601206 |
| H | 2.171755 | 1.531017 | -4.749788 |
| H | 1.530424 | 0.541337 | -3.429812 |
| C | 1.686191 | 6.261564 | -3.181591 |
| H | 0.605454 | 6.473989 | -3.263957 |

| | | | |
|---|---|---|---|
| H | 2.109023 | 6.326929 | -4.196091 |
| H | 2.128686 | 7.065851 | -2.573109 |
| C | 2.015130 | 3.371968 | 0.931625 |
| H | 1.127135 | 2.783999 | 1.217776 |
| H | 1.904121 | 4.357511 | 1.404085 |
| H | 2.889295 | 2.879625 | 1.379180 |
| C | -1.443770 | 3.229098 | -1.272276 |
| H | -0.507803 | 2.786809 | -1.649937 |
| H | -1.570690 | 4.202630 | -1.769681 |
| H | -2.261243 | 2.567674 | -1.585191 |
| C | -0.519846 | 6.277818 | 2.585496 |
| H | -0.648484 | 6.377599 | 3.674010 |
| H | -1.112150 | 7.068511 | 2.096823 |
| H | 0.540838 | 6.485994 | 2.356615 |
| C | -1.709035 | 1.502065 | 3.572792 |
| H | -1.668955 | 0.484202 | 3.161223 |
| H | -2.714672 | 1.627783 | 4.007556 |
| H | -0.986464 | 1.579601 | 4.399743 |
| C | 5.250760 | 1.008757 | -0.232086 |
| C | 6.332735 | 0.012516 | 0.268823 |
| H | 6.703626 | -0.583235 | -0.579471 |
| H | 7.199708 | 0.525842 | 0.708745 |
| C | 5.718490 | -0.363122 | 2.704705 |
| H | 5.235877 | 0.619631 | 2.784347 |
| H | 6.773823 | -0.250217 | 2.996402 |
| H | 5.243069 | -1.042250 | 3.422843 |
| C | 5.647395 | -0.923640 | 1.270373 |
| C | 6.240707 | -2.332938 | 1.273841 |
| H | 5.652609 | -3.015038 | 1.905139 |
| H | 7.262075 | -2.286613 | 1.680592 |
| H | 6.301235 | -2.758586 | 0.266263 |
| C | 5.370917 | 2.377636 | 0.465764 |
| H | 4.648668 | 3.090958 | 0.050364 |
| H | 6.380640 | 2.786271 | 0.299035 |
| H | 5.210216 | 2.311701 | 1.550987 |
| C | 5.439454 | 1.223205 | -1.747222 |
| H | 5.294256 | 0.283454 | -2.302324 |
| H | 6.466594 | 1.575546 | -1.939570 |
| H | 4.745272 | 1.975770 | -2.140381 |
| C | -4.904864 | 1.294064 | 0.637868 |
| C | -4.831296 | 2.532198 | -0.279232 |
| H | -3.977632 | 3.171562 | -0.021669 |
| H | -5.749579 | 3.135659 | -0.171312 |
| H | -4.737795 | 2.238914 | -1.335883 |

| | | | |
|---|---|---|---|
| C | -4.923138 | 1.786241 | 2.098095 |
| H | -4.922117 | 0.954962 | 2.816066 |
| H | -5.825141 | 2.394940 | 2.279155 |
| H | -4.050111 | 2.417334 | 2.303826 |
| C | -6.194541 | 0.494603 | 0.297433 |
| H | -6.521482 | 0.741509 | -0.724972 |
| H | -7.028355 | 0.741611 | 0.971787 |
| C | -5.815960 | -0.993857 | 0.347280 |
| C | -5.967638 | -1.538467 | 1.785757 |
| H | -5.805683 | -2.622053 | 1.828468 |
| H | -6.986510 | -1.340892 | 2.154970 |
| H | -5.256893 | -1.056675 | 2.470446 |
| C | -6.663142 | -1.853011 | -0.592403 |
| H | -6.657300 | -1.464076 | -1.617958 |
| H | -7.705577 | -1.866575 | -0.238748 |
| H | -6.299338 | -2.891645 | -0.618185 |
| C | -3.744899 | -1.891093 | -0.896571 |
| C | -3.125940 | -3.023052 | -0.309771 |
| C | -2.505833 | -3.965281 | -1.143732 |
| H | -2.027624 | -4.840849 | -0.698289 |
| C | -2.468349 | -3.793362 | -2.524394 |
| H | -1.968871 | -4.531256 | -3.157473 |
| C | -3.066606 | -2.671219 | -3.096401 |
| H | -3.034309 | -2.540693 | -4.180644 |
| C | -3.717950 | -1.712065 | -2.308429 |
| C | -4.392943 | -0.528356 | -2.993305 |
| H | -4.943488 | 0.019681 | -2.219048 |
| C | -3.378066 | 0.451357 | -3.602912 |
| H | -2.684641 | 0.829773 | -2.839492 |
| H | -3.896385 | 1.314747 | -4.051996 |
| H | -2.780086 | -0.031457 | -4.393295 |
| C | -5.403615 | -0.981435 | -4.061896 |
| H | -4.899315 | -1.442833 | -4.926276 |
| H | -5.974841 | -0.117420 | -4.438943 |
| H | -6.118792 | -1.718629 | -3.666373 |
| C | -3.115072 | -3.246688 | 1.195475 |
| H | -3.562036 | -2.358365 | 1.656300 |
| C | -1.693395 | -3.369339 | 1.759466 |
| H | -1.181287 | -4.265429 | 1.376669 |
| H | -1.724384 | -3.448596 | 2.859290 |
| H | -1.085638 | -2.493593 | 1.493065 |
| C | -3.950991 | -4.481491 | 1.576074 |
| H | -4.962005 | -4.443188 | 1.142614 |
| H | -4.049901 | -4.565948 | 2.670899 |

| | | | |
|---|---|---|---|
| H | -3.474924 | -5.406492 | 1.210846 |
| C | 3.336951 | -1.996566 | 0.894747 |
| C | 3.330615 | -2.983939 | -0.125321 |
| C | 2.499592 | -4.096079 | 0.044201 |
| H | 2.472890 | -4.867647 | -0.727758 |
| C | 1.692695 | -4.229905 | 1.170766 |
| H | 1.052239 | -5.107304 | 1.282643 |
| C | 1.686532 | -3.234609 | 2.142653 |
| H | 1.022982 | -3.330784 | 3.003555 |
| C | 2.503100 | -2.101332 | 2.026979 |
| C | 2.423536 | -1.017559 | 3.091895 |
| H | 3.152230 | -0.240136 | 2.834440 |
| C | 2.771873 | -1.557631 | 4.489109 |
| H | 2.005435 | -2.264407 | 4.845166 |
| H | 2.825875 | -0.734060 | 5.219251 |
| H | 3.736136 | -2.089320 | 4.498503 |
| C | 1.046170 | -0.342159 | 3.102562 |
| H | 0.784792 | 0.073123 | 2.118701 |
| H | 1.031870 | 0.481614 | 3.831797 |
| H | 0.248705 | -1.050023 | 3.376540 |
| C | 4.157837 | -2.873231 | -1.404273 |
| H | 4.860318 | -2.037456 | -1.282621 |
| C | 4.982413 | -4.142713 | -1.682099 |
| H | 5.571670 | -4.454930 | -0.806825 |
| H | 5.675517 | -3.971346 | -2.521339 |
| H | 4.336271 | -4.989301 | -1.962633 |
| C | 3.274086 | -2.543546 | -2.620288 |
| H | 3.888318 | -2.463306 | -3.532255 |
| H | 2.736392 | -1.597324 | -2.480522 |
| H | 2.518303 | -3.328623 | -2.783614 |

**4** (triplet)

| | | | |
|---|---|---|---|
| Pb | -0.025550 | -0.978544 | -0.558168 |
| Si | 2.022487 | 0.755383 | -0.303908 |
| Si | -1.994186 | 0.802778 | -0.045546 |
| N | 4.161158 | -0.974466 | 0.495672 |
| N | -4.379340 | -0.800027 | -0.221606 |
| C | 3.778971 | 0.221837 | -0.091879 |
| C | -3.804476 | 0.433547 | 0.039850 |
| C | 2.012892 | 2.542434 | -0.986237 |
| C | -1.691713 | 2.473458 | 0.827974 |
| C | 1.931113 | 2.819053 | -2.375383 |
| C | 1.935468 | 4.147641 | -2.822086 |

| | | | |
|---|---|---|---|
| H | 1.889839 | 4.345840 | -3.897704 |
| C | 1.985468 | 5.227731 | -1.933382 |
| C | 2.013161 | 4.945397 | -0.563816 |
| H | 2.024806 | 5.773824 | 0.151290 |
| C | 2.012244 | 3.631406 | -0.078147 |
| C | -1.391784 | 3.629276 | 0.063279 |
| C | -1.134463 | 4.844442 | 0.710426 |
| H | -0.902563 | 5.725529 | 0.105082 |
| C | -1.139291 | 4.957256 | 2.103786 |
| C | -1.409953 | 3.806444 | 2.852921 |
| H | -1.403158 | 3.869120 | 3.945918 |
| C | -1.671797 | 2.571583 | 2.244034 |
| C | 1.803773 | 1.711291 | -3.396112 |
| H | 2.591265 | 0.950606 | -3.285680 |
| H | 1.852240 | 2.102466 | -4.423741 |
| H | 0.844685 | 1.179533 | -3.275053 |
| C | 1.948101 | 6.651801 | -2.430776 |
| H | 0.911094 | 7.031680 | -2.462169 |
| H | 2.356787 | 6.738533 | -3.449447 |
| H | 2.519189 | 7.326013 | -1.773232 |
| C | 1.977119 | 3.405711 | 1.416205 |
| H | 1.026562 | 2.933315 | 1.709232 |
| H | 2.055314 | 4.354075 | 1.968276 |
| H | 2.789294 | 2.744180 | 1.754913 |
| C | -1.361215 | 3.592631 | -1.446711 |
| H | -0.907450 | 2.660457 | -1.815039 |
| H | -0.774768 | 4.428357 | -1.852845 |
| H | -2.377611 | 3.648755 | -1.870828 |
| C | -0.795774 | 6.265128 | 2.771936 |
| H | -1.163766 | 6.304867 | 3.808672 |
| H | -1.216349 | 7.123482 | 2.224353 |
| H | 0.299147 | 6.409260 | 2.805721 |
| C | -1.873961 | 1.360998 | 3.127020 |
| H | -2.636505 | 0.677005 | 2.728145 |
| H | -2.170514 | 1.649770 | 4.147126 |
| H | -0.941271 | 0.779827 | 3.203195 |
| C | 5.068267 | 0.997964 | -0.464207 |
| C | 6.189741 | -0.024927 | -0.138082 |
| H | 6.458807 | -0.577439 | -1.052224 |
| H | 7.106133 | 0.462590 | 0.226143 |
| C | 5.835370 | -0.509212 | 2.326664 |
| H | 5.311832 | 0.436348 | 2.518020 |
| H | 6.911380 | -0.341271 | 2.490029 |
| H | 5.499772 | -1.245224 | 3.066519 |

| | | | |
|---|---|---|---|
| C | 5.608768 | -1.010865 | 0.884687 |
| C | 6.214673 | -2.410919 | 0.763410 |
| H | 5.703188 | -3.128333 | 1.422520 |
| H | 7.273866 | -2.371224 | 1.060197 |
| H | 6.167743 | -2.793000 | -0.262973 |
| C | 5.254775 | 2.307827 | 0.329452 |
| H | 4.492171 | 3.045210 | 0.050507 |
| H | 6.241975 | 2.743323 | 0.102130 |
| H | 5.197897 | 2.150345 | 1.415208 |
| C | 5.124837 | 1.337176 | -1.967282 |
| H | 4.925558 | 0.446539 | -2.583225 |
| H | 6.130586 | 1.711338 | -2.224594 |
| H | 4.399968 | 2.116480 | -2.232675 |
| C | -4.946274 | 1.449029 | 0.269322 |
| C | -4.793198 | 2.753082 | -0.534368 |
| H | -3.987249 | 3.382147 | -0.137360 |
| H | -5.729671 | 3.333568 | -0.477385 |
| H | -4.584725 | 2.541931 | -1.594510 |
| C | -5.102064 | 1.822540 | 1.760859 |
| H | -5.164549 | 0.939324 | 2.410958 |
| H | -6.019852 | 2.417069 | 1.904629 |
| H | -4.252122 | 2.429820 | 2.097288 |
| C | -6.181679 | 0.679629 | -0.260602 |
| H | -6.317520 | 0.918163 | -1.327381 |
| H | -7.108347 | 0.965609 | 0.259058 |
| C | -5.877810 | -0.818101 | -0.127307 |
| C | -6.370715 | -1.390337 | 1.216023 |
| H | -6.151819 | -2.463015 | 1.288554 |
| H | -7.462356 | -1.265753 | 1.286559 |
| H | -5.917663 | -0.885402 | 2.077170 |
| C | -6.536710 | -1.635519 | -1.243997 |
| H | -6.343678 | -1.207362 | -2.233802 |
| H | -7.626647 | -1.641806 | -1.090445 |
| H | -6.187976 | -2.679041 | -1.238354 |
| C | -3.621521 | -1.973702 | -0.557268 |
| C | -3.163150 | -2.849298 | 0.461652 |
| C | -2.465184 | -4.005618 | 0.083927 |
| H | -2.103820 | -4.686353 | 0.857551 |
| C | -2.198646 | -4.288367 | -1.252043 |
| H | -1.642414 | -5.189390 | -1.522393 |
| C | -2.619502 | -3.403001 | -2.240739 |
| H | -2.382957 | -3.616184 | -3.285363 |
| C | -3.331951 | -2.237737 | -1.923387 |
| C | -3.747347 | -1.308806 | -3.063178 |

| | | | |
|---|---|---|---|
| H | -4.364053 | -0.507844 | -2.632846 |
| C | -2.539252 | -0.630026 | -3.732431 |
| H | -1.960881 | -0.036409 | -3.010875 |
| H | -2.879092 | 0.040726 | -4.538995 |
| H | -1.858032 | -1.373705 | -4.177377 |
| C | -4.582187 | -2.046951 | -4.126594 |
| H | -3.962285 | -2.757283 | -4.696635 |
| H | -5.003053 | -1.329273 | -4.849518 |
| H | -5.412542 | -2.615797 | -3.684185 |
| C | -3.352033 | -2.576201 | 1.950873 |
| H | -3.931456 | -1.650828 | 2.047751 |
| C | -2.010353 | -2.334266 | 2.662088 |
| H | -1.357734 | -3.220570 | 2.609267 |
| H | -2.177738 | -2.099123 | 3.726140 |
| H | -1.462887 | -1.500106 | 2.205619 |
| C | -4.119214 | -3.710256 | 2.653896 |
| H | -5.057023 | -3.964507 | 2.138126 |
| H | -4.364578 | -3.424991 | 3.689879 |
| H | -3.512925 | -4.629258 | 2.701693 |
| C | 3.301377 | -2.117143 | 0.612016 |
| C | 3.167955 | -2.980176 | -0.509793 |
| C | 2.335335 | -4.100999 | -0.395038 |
| H | 2.213629 | -4.765741 | -1.252961 |
| C | 1.656453 | -4.380552 | 0.789162 |
| H | 1.007761 | -5.257552 | 0.855975 |
| C | 1.796011 | -3.529157 | 1.880634 |
| H | 1.247965 | -3.742018 | 2.800936 |
| C | 2.604957 | -2.384797 | 1.815692 |
| C | 2.683852 | -1.476245 | 3.035451 |
| H | 3.317691 | -0.622156 | 2.768670 |
| C | 3.319186 | -2.204930 | 4.233004 |
| H | 2.658272 | -3.004622 | 4.605214 |
| H | 3.494962 | -1.504636 | 5.065837 |
| H | 4.279700 | -2.674056 | 3.969646 |
| C | 1.313341 | -0.900772 | 3.425705 |
| H | 0.867142 | -0.359661 | 2.579635 |
| H | 1.419270 | -0.200081 | 4.270236 |
| H | 0.608917 | -1.689444 | 3.732636 |
| C | 3.890680 | -2.735530 | -1.832131 |
| H | 4.600140 | -1.912800 | -1.675012 |
| C | 4.694586 | -3.966011 | -2.289146 |
| H | 5.349534 | -4.350097 | -1.492497 |
| H | 5.322876 | -3.710250 | -3.157773 |
| H | 4.032889 | -4.791008 | -2.597865 |

| | | | |
|---|---|---|---|
| C | 2.926419 | -2.287205 | -2.942850 |
| H | 3.477301 | -2.106801 | -3.880801 |
| H | 2.403731 | -1.362185 | -2.665446 |
| H | 2.161709 | -3.056070 | -3.140734 |

**E**

| | | | |
|---|---|---|---|
| Sn | 2.020382 | 1.218447 | 0.340938 |
| N | 2.929472 | 2.802989 | -0.751499 |
| Si | 3.299857 | 2.413776 | -2.436158 |
| C | 3.721020 | 0.569777 | -2.598665 |
| C | 4.810552 | 3.367290 | -3.056455 |
| C | 1.831741 | 2.760059 | -3.577459 |
| Si | 3.010033 | 4.465292 | -0.156040 |
| C | 4.791061 | 5.050886 | 0.104582 |
| C | 2.044897 | 4.663029 | 1.459158 |
| C | 2.203033 | 5.659363 | -1.388982 |
| N | 2.918724 | 1.394651 | 2.263249 |
| Si | 4.664637 | 1.516303 | 2.509944 |
| C | 5.282719 | 0.119438 | 3.634295 |
| C | 5.606740 | 1.313853 | 0.881602 |
| C | 5.160674 | 3.152349 | 3.323794 |
| Si | 1.825844 | 1.059849 | 3.612737 |
| C | 1.754639 | -0.787227 | 4.013891 |
| C | 0.065033 | 1.616977 | 3.173677 |
| C | 2.319047 | 2.015629 | 5.168201 |
| H | 4.551456 | 0.282065 | -1.932953 |
| H | 2.864029 | -0.093949 | -2.392210 |
| H | 4.039452 | 0.362049 | -3.634443 |
| H | 4.645220 | 4.455194 | -3.088528 |
| H | 5.687796 | 3.175410 | -2.417906 |
| H | 5.056619 | 3.037899 | -4.080065 |
| H | 0.947178 | 2.185888 | -3.253921 |
| H | 1.557433 | 3.826230 | -3.573344 |
| H | 2.059870 | 2.470561 | -4.617314 |
| H | 5.379630 | 4.344953 | 0.707331 |
| H | 5.307870 | 5.178497 | -0.858855 |
| H | 4.800202 | 6.025271 | 0.621850 |
| H | 0.974134 | 4.439834 | 1.310883 |
| H | 2.419539 | 4.013248 | 2.261427 |
| H | 2.117120 | 5.710923 | 1.795195 |
| H | 2.721282 | 5.685623 | -2.360367 |
| H | 1.151010 | 5.387460 | -1.574201 |
| H | 2.220925 | 6.682738 | -0.977115 |
| H | 4.854552 | 0.163034 | 4.647959 |

| | | | |
|---|---|---|---|
| H | 5.041649 | -0.867735 | 3.207233 |
| H | 6.379372 | 0.185335 | 3.735839 |
| H | 5.432880 | 0.314478 | 0.446438 |
| H | 5.329875 | 2.064644 | 0.129717 |
| H | 6.688016 | 1.399963 | 1.080569 |
| H | 4.715421 | 4.021785 | 2.819571 |
| H | 4.841557 | 3.178816 | 4.376938 |
| H | 6.256894 | 3.274301 | 3.302941 |
| H | 1.437303 | -1.362934 | 3.128052 |
| H | 2.738320 | -1.170734 | 4.326242 |
| H | 1.035157 | -0.991739 | 4.824849 |
| H | 0.034472 | 2.684804 | 2.901297 |
| H | -0.383025 | 1.035602 | 2.349753 |
| H | -0.589049 | 1.478682 | 4.051345 |
| H | 3.293387 | 1.700020 | 5.571668 |
| H | 2.367401 | 3.098154 | 4.968499 |
| H | 1.563349 | 1.848398 | 5.954215 |

**F**

| | | | |
|---|---|---|---|
| Sn | 5.136986 | 8.279685 | 9.160108 |
| P | 5.890896 | 10.462617 | 7.859441 |
| P | 4.087518 | 7.316019 | 6.919807 |
| C | 6.170524 | 11.904918 | 9.006329 |
| C | 7.343277 | 12.696097 | 8.842043 |
| C | 7.592331 | 13.739836 | 9.744878 |
| H | 8.504322 | 14.332484 | 9.637838 |
| C | 6.693002 | 14.051384 | 10.761836 |
| H | 6.910356 | 14.860674 | 11.463699 |
| C | 5.492046 | 13.351120 | 10.843893 |
| H | 4.757715 | 13.636507 | 11.601254 |
| C | 5.199488 | 12.295797 | 9.967642 |
| C | 8.312573 | 12.527718 | 7.669758 |
| H | 7.972953 | 11.679412 | 7.061461 |
| C | 8.260145 | 13.764680 | 6.754292 |
| H | 7.231147 | 13.961480 | 6.415111 |
| H | 8.892530 | 13.612410 | 5.864030 |
| H | 8.621878 | 14.668564 | 7.271046 |
| C | 9.753622 | 12.220796 | 8.104595 |
| H | 10.177780 | 13.037673 | 8.711187 |
| H | 10.400847 | 12.094184 | 7.221536 |
| H | 9.809826 | 11.293334 | 8.692016 |
| C | 3.810938 | 11.664680 | 10.058475 |
| H | 3.742588 | 10.868462 | 9.303368 |

| | | | |
|---|---|---|---|
| C | 3.532523 | 11.021040 | 11.426356 |
| H | 4.314915 | 10.294184 | 11.696329 |
| H | 2.567913 | 10.488324 | 11.411868 |
| H | 3.491666 | 11.776173 | 12.228015 |
| C | 2.722284 | 12.689708 | 9.696237 |
| H | 2.704932 | 13.531130 | 10.407783 |
| H | 1.728505 | 12.216459 | 9.708665 |
| H | 2.890182 | 13.104432 | 8.690099 |
| C | 7.460210 | 9.433254 | 7.983645 |
| C | 7.893741 | 8.806593 | 6.771170 |
| C | 9.009643 | 7.969459 | 6.785921 |
| H | 9.334309 | 7.512609 | 5.846704 |
| C | 9.728140 | 7.694976 | 7.958224 |
| C | 9.307174 | 8.312856 | 9.134183 |
| H | 9.858112 | 8.123462 | 10.060017 |
| C | 8.199375 | 9.179020 | 9.181573 |
| C | 7.183674 | 9.038911 | 5.459343 |
| H | 6.157807 | 8.637897 | 5.480065 |
| H | 7.721676 | 8.552913 | 4.631975 |
| H | 7.093073 | 10.114558 | 5.238068 |
| C | 10.891612 | 6.737352 | 7.937240 |
| H | 11.549916 | 6.924089 | 7.073718 |
| H | 10.537353 | 5.694745 | 7.851089 |
| H | 11.497032 | 6.807181 | 8.853160 |
| C | 7.916926 | 9.849144 | 10.508462 |
| H | 8.342610 | 10.864651 | 10.530668 |
| H | 8.359645 | 9.273840 | 11.334334 |
| H | 6.846269 | 9.976860 | 10.714687 |
| C | 3.945705 | 5.456986 | 6.987357 |
| C | 2.721422 | 4.822035 | 6.622608 |
| C | 2.637596 | 3.424157 | 6.674593 |
| H | 1.699464 | 2.932315 | 6.411108 |
| C | 3.732296 | 2.641839 | 7.037802 |
| H | 3.642114 | 1.553410 | 7.082917 |
| C | 4.950102 | 3.259152 | 7.303341 |
| H | 5.821489 | 2.643059 | 7.538451 |
| C | 5.087169 | 4.655100 | 7.261184 |
| C | 1.526204 | 5.598517 | 6.068123 |
| H | 1.584469 | 6.629766 | 6.433469 |
| C | 1.627039 | 5.663284 | 4.532774 |
| H | 2.578988 | 6.121287 | 4.222443 |
| H | 0.803512 | 6.262924 | 4.110339 |
| H | 1.576704 | 4.653375 | 4.092845 |
| C | 0.158248 | 5.063515 | 6.513356 |

| | | | |
|---|---|---|---|
| H | -0.067458 | 4.075610 | 6.080519 |
| H | -0.638350 | 5.749613 | 6.184105 |
| H | 0.095873 | 4.976021 | 7.609511 |
| C | 6.490549 | 5.228137 | 7.455663 |
| H | 6.430551 | 6.322921 | 7.382901 |
| C | 7.085780 | 4.898566 | 8.834147 |
| H | 6.411574 | 5.208198 | 9.648387 |
| H | 8.045726 | 5.421670 | 8.972482 |
| H | 7.267952 | 3.817678 | 8.949088 |
| C | 7.429526 | 4.775601 | 6.324550 |
| H | 7.563451 | 3.681822 | 6.322766 |
| H | 8.421695 | 5.237772 | 6.442381 |
| H | 7.031407 | 5.067061 | 5.340078 |
| C | 2.642962 | 8.004250 | 7.908289 |
| C | 2.101723 | 9.242714 | 7.432878 |
| C | 1.097927 | 9.883949 | 8.160718 |
| H | 0.692867 | 10.824217 | 7.776214 |
| C | 0.586719 | 9.363431 | 9.357678 |
| C | 1.098404 | 8.145494 | 9.803521 |
| H | 0.702537 | 7.708727 | 10.725280 |
| C | 2.106031 | 7.447822 | 9.114509 |
| C | 2.564030 | 9.865253 | 6.137766 |
| H | 3.615807 | 10.187426 | 6.194744 |
| H | 1.953355 | 10.745370 | 5.887166 |
| H | 2.501249 | 9.143711 | 5.307316 |
| C | -0.451386 | 10.114582 | 10.151062 |
| H | -1.100160 | 10.720056 | 9.499681 |
| H | 0.030829 | 10.807537 | 10.863568 |
| H | -1.087252 | 9.434416 | 10.738053 |
| C | 2.496462 | 6.093313 | 9.662951 |
| H | 1.977141 | 5.289189 | 9.117868 |
| H | 2.227813 | 6.014244 | 10.726256 |
| H | 3.566838 | 5.869857 | 9.560856 |

**G**

| | | | |
|---|---|---|---|
| Sn | 2.835523 | 11.112568 | 3.493723 |
| B | 2.596726 | 9.079154 | 4.605830 |
| N | 1.472763 | 8.382467 | 5.201648 |
| C | 1.723248 | 7.003342 | 5.167448 |
| C | 2.937554 | 6.779832 | 4.605468 |
| N | 3.502650 | 8.006363 | 4.247376 |
| C | 4.771593 | 8.096627 | 3.596782 |
| C | 5.941028 | 8.172821 | 4.388901 |
| C | 7.175034 | 8.288306 | 3.735540 |

| | | | |
|---|---|---|---|
| C | 7.250248 | 8.305857 | 2.343016 |
| C | 6.090991 | 8.202152 | 1.577210 |
| C | 4.830733 | 8.092113 | 2.183036 |
| C | 0.250352 | 8.870208 | 5.757973 |
| C | -0.778568 | 9.314417 | 4.900974 |
| C | -1.976459 | 9.765463 | 5.478635 |
| C | -2.162260 | 9.741855 | 6.857395 |
| C | -1.143455 | 9.274897 | 7.690860 |
| C | 0.079227 | 8.841058 | 7.165715 |
| B | 3.148125 | 13.030715 | 4.775476 |
| N | 4.330605 | 13.680001 | 5.303522 |
| C | 5.603337 | 13.161267 | 5.690520 |
| C | 6.569480 | 12.859029 | 4.705306 |
| C | 7.835688 | 12.427415 | 5.128464 |
| C | 8.146363 | 12.320173 | 6.482660 |
| C | 7.179384 | 12.619069 | 7.441963 |
| C | 5.892844 | 13.029528 | 7.069649 |
| C | 4.852364 | 13.340457 | 8.141260 |
| C | 5.208939 | 14.624435 | 8.909507 |
| C | 4.644011 | 12.162094 | 9.104569 |
| C | 4.066915 | 15.047463 | 5.465501 |
| C | 2.791545 | 15.307009 | 5.084737 |
| N | 2.195358 | 14.117297 | 4.658470 |
| C | 0.852167 | 14.077503 | 4.173469 |
| C | 0.608832 | 14.225837 | 2.788352 |
| C | -0.720197 | 14.167230 | 2.343250 |
| C | -1.768698 | 13.973968 | 3.239174 |
| C | -1.512328 | 13.852723 | 4.605397 |
| C | -0.203865 | 13.915341 | 5.102033 |
| C | 0.087862 | 13.826687 | 6.597264 |
| C | -1.077179 | 14.302251 | 7.475547 |
| C | 0.510612 | 12.405964 | 7.001820 |
| C | 1.739923 | 14.522453 | 1.810655 |
| C | 1.846723 | 16.039582 | 1.570131 |
| C | 1.615088 | 13.759079 | 0.485030 |
| C | 5.851391 | 8.140245 | 5.910297 |
| C | 5.671699 | 9.556122 | 6.478126 |
| C | 7.047642 | 7.443816 | 6.574326 |
| C | 3.577494 | 7.901861 | 1.336389 |
| C | 3.404231 | 6.416793 | 0.968301 |
| C | 3.550430 | 8.788743 | 0.084081 |
| C | 1.218730 | 8.391667 | 8.076818 |
| C | 0.755655 | 7.858329 | 9.437925 |
| C | 2.247596 | 9.520626 | 8.260179 |

| | | | |
|---|---|---|---|
| C | 6.291927 | 13.033271 | 3.217286 |
| C | 6.495528 | 11.724431 | 2.440353 |
| C | 7.136564 | 14.174313 | 2.625846 |
| C | -0.649068 | 9.266293 | 3.383572 |
| C | -1.609775 | 8.226834 | 2.781556 |
| C | -0.849723 | 10.648055 | 2.746603 |
| H | 1.001676 | 6.286565 | 5.553581 |
| H | 3.456729 | 5.838529 | 4.438361 |
| H | 8.092061 | 8.363813 | 4.321903 |
| H | 8.221897 | 8.398968 | 1.850964 |
| H | 6.166706 | 8.203556 | 0.488297 |
| H | -2.781165 | 10.124010 | 4.832586 |
| H | -3.104618 | 10.088403 | 7.289507 |
| H | -1.303882 | 9.265506 | 8.769575 |
| H | 8.596534 | 12.188016 | 4.381679 |
| H | 9.143019 | 11.994945 | 6.792025 |
| H | 7.425886 | 12.522074 | 8.501914 |
| H | 3.892608 | 13.511133 | 7.633341 |
| H | 4.429213 | 14.863094 | 9.651321 |
| H | 5.306749 | 15.485906 | 8.231620 |
| H | 6.163975 | 14.511463 | 9.448994 |
| H | 3.836447 | 12.389912 | 9.819055 |
| H | 5.551455 | 11.942706 | 9.690445 |
| H | 4.364525 | 11.252353 | 8.557868 |
| H | 4.823777 | 15.727979 | 5.847925 |
| H | 2.248999 | 16.250028 | 5.089322 |
| H | -0.937614 | 14.277469 | 1.279213 |
| H | -2.797018 | 13.922448 | 2.871893 |
| H | -2.347450 | 13.714801 | 5.293183 |
| H | 0.941658 | 14.493745 | 6.796888 |
| H | -0.753623 | 14.362955 | 8.526700 |
| H | -1.444018 | 15.295513 | 7.171987 |
| H | -1.925855 | 13.599712 | 7.440932 |
| H | 0.721653 | 12.358096 | 8.082027 |
| H | 1.414924 | 12.082123 | 6.469151 |
| H | -0.289266 | 11.687607 | 6.779760 |
| H | 2.679310 | 14.201495 | 2.283348 |
| H | 2.685852 | 16.268646 | 0.892748 |
| H | 2.013613 | 16.582240 | 2.512974 |
| H | 0.922504 | 16.429925 | 1.112372 |
| H | 2.515127 | 13.921306 | -0.129435 |
| H | 0.750916 | 14.097470 | -0.109716 |
| H | 1.515158 | 12.676622 | 0.658159 |
| H | 4.951478 | 7.562631 | 6.171195 |

| | | | |
|---|---|---|---|
| H | 5.597755 | 9.527782 | 7.576338 |
| H | 6.526368 | 10.193227 | 6.213502 |
| H | 4.761715 | 10.037028 | 6.092209 |
| H | 6.855675 | 7.312092 | 7.651090 |
| H | 7.971030 | 8.038712 | 6.483642 |
| H | 7.238901 | 6.451660 | 6.135757 |
| H | 2.714975 | 8.185713 | 1.956696 |
| H | 2.483860 | 6.266622 | 0.380251 |
| H | 4.256099 | 6.059703 | 0.365953 |
| H | 3.338061 | 5.787500 | 1.868589 |
| H | 2.573668 | 8.702514 | -0.418474 |
| H | 3.705767 | 9.846780 | 0.344623 |
| H | 4.319210 | 8.493257 | -0.648478 |
| H | 1.740015 | 7.565882 | 7.569985 |
| H | 1.611023 | 7.429892 | 9.984050 |
| H | 0.337255 | 8.656698 | 10.072539 |
| H | -0.009784 | 7.073394 | 9.331580 |
| H | 3.124183 | 9.156793 | 8.820052 |
| H | 1.810943 | 10.361105 | 8.821748 |
| H | 2.598012 | 9.907781 | 7.293167 |
| H | 5.238538 | 13.325513 | 3.104269 |
| H | 6.190597 | 11.841875 | 1.388446 |
| H | 7.548524 | 11.401824 | 2.449466 |
| H | 5.910301 | 10.894022 | 2.866546 |
| H | 6.892248 | 14.326216 | 1.561654 |
| H | 6.952035 | 15.120626 | 3.157993 |
| H | 8.214407 | 13.952930 | 2.695183 |
| H | 0.370092 | 8.932940 | 3.143536 |
| H | -1.474337 | 8.160589 | 1.689478 |
| H | -2.662102 | 8.493311 | 2.974242 |
| H | -1.431369 | 7.228021 | 3.209781 |
| H | -0.644712 | 10.616168 | 1.664982 |
| H | -0.187037 | 11.408662 | 3.190027 |
| H | -1.877802 | 11.017032 | 2.883099 |

**H**

| | | | |
|---|---|---|---|
| Pb | -1.133802 | -6.611768 | 17.011573 |
| Si | 0.726770 | -8.630154 | 16.769750 |
| Si | -0.693157 | -5.069234 | 19.273081 |
| Si | 1.034179 | -8.920897 | 14.432561 |
| Si | 2.845647 | -8.511265 | 17.796502 |
| Si | -0.361617 | -10.584604 | 17.566387 |
| Si | 1.248515 | -4.347525 | 20.435640 |
| Si | -1.775692 | -3.087451 | 18.510181 |

| | | | |
|---|---|---|---|
| Si | -2.166596 | -6.023355 | 20.880209 |
| C | 2.315596 | -7.666679 | 13.801660 |
| H | 3.296047 | -7.817900 | 14.281522 |
| H | 1.998096 | -6.631396 | 14.009201 |
| H | 2.454450 | -7.766783 | 12.711344 |
| C | 1.653206 | -10.668786 | 14.018970 |
| H | 2.575952 | -10.913898 | 14.567836 |
| H | 1.864630 | -10.753210 | 12.938812 |
| H | 0.895963 | -11.427929 | 14.273212 |
| C | -0.589661 | -8.639774 | 13.487783 |
| H | -0.948194 | -7.602861 | 13.595643 |
| H | -1.387447 | -9.306893 | 13.851782 |
| H | -0.445893 | -8.836899 | 12.411369 |
| C | 3.538541 | -6.756801 | 17.582393 |
| H | 2.813382 | -6.001150 | 17.920417 |
| H | 3.749432 | -6.564180 | 16.517717 |
| H | 4.475637 | -6.614152 | 18.146859 |
| C | 4.059943 | -9.726386 | 16.982102 |
| H | 4.227518 | -9.477348 | 15.922019 |
| H | 3.681488 | -10.759850 | 17.030636 |
| H | 5.037719 | -9.696937 | 17.493683 |
| C | 2.693197 | -8.986917 | 19.628185 |
| H | 1.879404 | -8.438567 | 20.125201 |
| H | 3.627390 | -8.779832 | 20.176716 |
| H | 2.480842 | -10.064602 | 19.718449 |
| C | -0.960509 | -10.323762 | 19.349307 |
| H | -1.718788 | -9.526834 | 19.409638 |
| H | -0.124208 | -10.039139 | 20.006748 |
| H | -1.411354 | -11.247723 | 19.750574 |
| C | 0.835753 | -12.058499 | 17.559615 |
| H | 1.243457 | -12.242442 | 16.553662 |
| H | 0.318227 | -12.974267 | 17.894484 |
| H | 1.683946 | -11.882377 | 18.240242 |
| C | -1.858863 | -11.010654 | 16.478474 |
| H | -1.549722 | -11.235632 | 15.444930 |
| H | -2.579334 | -10.177062 | 16.438209 |
| H | -2.385561 | -11.896237 | 16.873845 |
| C | 2.411207 | -5.718103 | 21.032347 |
| H | 2.911880 | -6.236775 | 20.205743 |
| H | 1.876075 | -6.469667 | 21.633423 |
| H | 3.190615 | -5.267973 | 21.671655 |
| C | 2.228612 | -3.142637 | 19.342030 |
| H | 2.564161 | -3.624853 | 18.409786 |
| H | 3.123369 | -2.780392 | 19.876892 |

| | | | |
|---|---|---|---|
| H | 1.619700 | -2.265805 | 19.068489 |
| C | 0.704378 | -3.427027 | 22.009341 |
| H | -0.022761 | -2.627868 | 21.806865 |
| H | 1.586624 | -2.974510 | 22.494973 |
| H | 0.249290 | -4.125475 | 22.730238 |
| C | -1.701913 | -1.645550 | 19.744741 |
| H | -0.665608 | -1.318762 | 19.925195 |
| H | -2.147505 | -1.918365 | 20.714217 |
| H | -2.261523 | -0.783214 | 19.342627 |
| C | -3.608295 | -3.433525 | 18.138018 |
| H | -3.728330 | -4.246862 | 17.403869 |
| H | -4.089598 | -2.530811 | 17.723877 |
| H | -4.157024 | -3.719159 | 19.049835 |
| C | -0.952377 | -2.500449 | 16.897991 |
| H | -1.071786 | -3.244141 | 16.091561 |
| H | 0.126738 | -2.326253 | 17.038756 |
| H | -1.406422 | -1.557199 | 16.548043 |
| C | -2.935367 | -4.688344 | 21.990801 |
| H | -2.163822 | -4.090510 | 22.499629 |
| H | -3.571807 | -5.155982 | 22.762232 |
| H | -3.564833 | -3.998499 | 21.406330 |
| C | -1.221880 | -7.253149 | 21.974603 |
| H | -0.432956 | -6.746344 | 22.553378 |
| H | -0.746107 | -8.040724 | 21.369277 |
| H | -1.906614 | -7.740189 | 22.690091 |
| C | -3.596973 | -6.944853 | 20.026921 |
| H | -3.240202 | -7.815876 | 19.451444 |
| H | -4.143050 | -6.281871 | 19.337141 |
| H | -4.314523 | -7.320676 | 20.776518 |

**2$^{\bullet-}$**

| | | | |
|---|---|---|---|
| Si | 1.308978 | -2.025418 | 11.423164 |
| Si | 0.093325 | 1.419321 | 11.714507 |
| N | -0.564459 | 3.319539 | 13.763094 |
| N | 1.401517 | -4.465827 | 12.955266 |
| C | 1.391030 | -1.604409 | 8.527939 |
| C | -1.373762 | 4.072806 | 11.646881 |
| C | 2.081560 | -1.462262 | 9.758055 |
| C | -0.608369 | 2.944295 | 12.394690 |
| C | 0.987180 | 2.058037 | 9.026060 |
| C | -1.502707 | 1.631129 | 15.284246 |
| C | 0.881608 | 2.107151 | 7.630970 |
| H | 1.687342 | 2.572868 | 7.054015 |
| C | 2.577154 | -4.710900 | 10.912958 |

| | | | |
|---|---|---|---|
| C | 1.945834 | -1.099358 | 7.344182 |
| H | 1.389019 | -1.216994 | 6.408206 |
| C | -0.047279 | 1.466050 | 9.798245 |
| C | -1.327457 | 0.749275 | 16.356579 |
| H | -2.172786 | 0.147059 | 16.698662 |
| C | 2.281758 | -4.721836 | 9.404668 |
| H | 2.618935 | -3.805025 | 8.908419 |
| H | 1.204133 | -4.837122 | 9.219629 |
| H | 2.805192 | -5.573297 | 8.933859 |
| C | 3.326476 | -0.776646 | 9.747568 |
| C | -0.661012 | -3.839394 | 14.161047 |
| C | 4.084130 | -4.418503 | 11.100725 |
| H | 4.702418 | -5.206443 | 10.632520 |
| H | 4.360293 | -4.350102 | 12.162393 |
| H | 4.337818 | -3.458738 | 10.628342 |
| C | 2.221561 | 2.642269 | 9.675816 |
| H | 2.670633 | 1.925313 | 10.377647 |
| H | 1.987659 | 3.545748 | 10.259993 |
| H | 2.981810 | 2.900495 | 8.922101 |
| C | -0.093092 | 0.611978 | 16.986889 |
| H | 0.023982 | -0.084295 | 17.821137 |
| C | -1.134110 | 0.869340 | 9.111542 |
| C | 3.175206 | -0.439144 | 7.326322 |
| C | 0.880144 | -2.770903 | 16.249251 |
| H | 1.469461 | -2.313073 | 17.047104 |
| C | 3.851305 | -0.290397 | 8.542585 |
| H | 4.812797 | 0.234571 | 8.555025 |
| C | 0.063989 | -2.320689 | 8.415399 |
| H | -0.338428 | -2.593614 | 9.401660 |
| H | 0.169709 | -3.249662 | 7.829876 |
| H | -0.673214 | -1.686623 | 7.900778 |
| C | -1.567644 | -4.321763 | 13.030569 |
| H | -0.920058 | -4.727229 | 12.241021 |
| C | 0.759004 | -3.870977 | 14.084930 |
| C | -1.207729 | 0.943428 | 7.711915 |
| H | -2.062435 | 0.487830 | 7.199772 |
| C | 1.535445 | -3.288994 | 15.124812 |
| C | -0.506670 | -2.792050 | 16.353296 |
| H | -0.999085 | -2.372043 | 17.233932 |
| C | 1.734919 | -3.745308 | 11.776456 |
| C | -0.211716 | 1.561992 | 6.951305 |
| C | 2.188164 | -6.096615 | 11.478469 |
| H | 1.322010 | -6.472017 | 10.910485 |
| H | 2.995908 | -6.837849 | 11.366308 |

| | | | |
|---|---|---|---|
| C | -0.391148 | 2.394595 | 14.833091 |
| C | -1.265427 | -3.307119 | 15.308377 |
| H | -2.355878 | -3.275075 | 15.373021 |
| C | 0.877832 | 2.220091 | 15.441273 |
| C | 3.490114 | -1.682042 | 14.934964 |
| H | 3.021111 | -1.196686 | 14.068919 |
| H | 4.586727 | -1.614846 | 14.829437 |
| H | 3.198889 | -1.106810 | 15.828991 |
| C | 3.053839 | -3.152295 | 15.047995 |
| H | 3.378207 | -3.651775 | 14.128276 |
| C | -2.542468 | -5.422505 | 13.490697 |
| H | -2.033575 | -6.251163 | 14.003039 |
| H | -3.088265 | -5.837636 | 12.626457 |
| H | -3.296326 | -5.017724 | 14.186027 |
| C | -2.206600 | 0.108042 | 9.857024 |
| H | -1.762657 | -0.745022 | 10.394128 |
| H | -2.980442 | -0.275989 | 9.173488 |
| H | -2.696422 | 0.725550 | 10.624188 |
| C | -2.878270 | 1.734684 | 14.634920 |
| H | -2.826602 | 2.545527 | 13.897543 |
| C | 1.775173 | -5.903291 | 12.944163 |
| C | 0.998081 | 1.338942 | 16.525338 |
| H | 1.974557 | 1.199082 | 16.996233 |
| C | 0.601839 | -6.827909 | 13.309343 |
| H | 0.234840 | -6.617494 | 14.325220 |
| H | 0.929398 | -7.879845 | 13.276237 |
| H | -0.232323 | -6.711756 | 12.606780 |
| C | 2.927049 | -6.241757 | 13.913780 |
| H | 3.833685 | -5.661177 | 13.705404 |
| H | 3.182661 | -7.310955 | 13.832605 |
| H | 2.622911 | -6.046204 | 14.950864 |
| C | 3.718246 | 0.154452 | 6.050199 |
| H | 3.454262 | -0.456925 | 5.171744 |
| H | 4.814957 | 0.256946 | 6.079805 |
| H | 3.298400 | 1.162111 | 5.879047 |
| C | -0.265377 | 1.573248 | 5.443382 |
| H | -1.289751 | 1.417249 | 5.069286 |
| H | 0.364974 | 0.768807 | 5.022383 |
| H | 0.109339 | 2.523858 | 5.028893 |
| C | -0.529907 | 4.833580 | 10.602571 |
| H | 0.391768 | 5.246308 | 11.035025 |
| H | -1.112651 | 5.673725 | 10.184318 |
| H | -0.251636 | 4.173278 | 9.771742 |
| C | 4.091629 | -0.517510 | 11.026549 |

| | | | |
|---|---|---|---|
| H | 3.527794 | 0.168123 | 11.681215 |
| H | 5.077978 | -0.070877 | 10.823010 |
| H | 4.235668 | -1.436189 | 11.615362 |
| C | -3.250101 | 0.450395 | 13.874378 |
| H | -2.482655 | 0.200341 | 13.129175 |
| H | -4.218487 | 0.574094 | 13.359194 |
| H | -3.336605 | -0.408546 | 14.560919 |
| C | -2.639755 | 3.555869 | 10.931823 |
| H | -2.382729 | 2.948879 | 10.054343 |
| H | -3.256988 | 4.404888 | 10.584413 |
| H | -3.251087 | 2.942169 | 11.610868 |
| C | 2.123706 | 2.938135 | 14.940749 |
| H | 1.826446 | 3.518774 | 14.059366 |
| C | -3.978443 | 2.091946 | 15.649746 |
| H | -4.144301 | 1.275275 | 16.371817 |
| H | -4.936195 | 2.269429 | 15.131717 |
| H | -3.730725 | 2.995899 | 16.228259 |
| C | -0.845861 | 4.758410 | 13.970955 |
| C | 2.683023 | 3.899809 | 16.004771 |
| H | 1.910652 | 4.584843 | 16.387880 |
| H | 3.507396 | 4.506299 | 15.592747 |
| H | 3.080048 | 3.341099 | 16.868705 |
| C | -2.366396 | -3.164062 | 12.405725 |
| H | -3.053152 | -2.706472 | 13.136832 |
| H | -2.969301 | -3.530059 | 11.557130 |
| H | -1.694230 | -2.375144 | 12.043204 |
| C | 3.217264 | 1.961634 | 14.478303 |
| H | 3.607386 | 1.357300 | 15.313251 |
| H | 4.065127 | 2.517876 | 14.043290 |
| H | 2.823406 | 1.272189 | 13.717796 |
| C | 3.767286 | -3.810566 | 16.242642 |
| H | 3.567377 | -3.259721 | 17.176564 |
| H | 4.859638 | -3.805098 | 16.088623 |
| H | 3.451586 | -4.852082 | 16.400640 |
| C | -1.807839 | 5.019878 | 12.799975 |
| H | -1.825736 | 6.077269 | 12.490478 |
| H | -2.828437 | 4.758815 | 13.123391 |
| C | 0.413218 | 5.650090 | 13.846535 |
| H | 1.054139 | 5.568487 | 14.732995 |
| H | 0.121429 | 6.708384 | 13.742371 |
| H | 1.009222 | 5.370669 | 12.967772 |
| C | -1.501066 | 5.032259 | 15.327821 |
| H | -0.828962 | 4.760793 | 16.156203 |
| H | -2.430469 | 4.460912 | 15.446898 |

| | | | |
|---|---|---|---|
| H | -1.739121 | 6.104643 | 15.415426 |
| Ge | 0.440987 | -0.512393 | 13.060371 |

**3•-**

| | | | |
|---|---|---|---|
| Si | 1.247857 | -2.212686 | 11.446341 |
| Si | 0.021475 | 1.586290 | 11.770207 |
| N | -0.646464 | 3.510989 | 13.783700 |
| N | 1.620153 | -4.661084 | 12.919493 |
| C | 1.125658 | -1.634585 | 8.586298 |
| C | -1.155713 | 4.372475 | 11.610460 |
| C | 1.921787 | -1.587210 | 9.759634 |
| C | -0.609191 | 3.151567 | 12.406835 |
| C | 1.071341 | 2.045067 | 9.103717 |
| C | -2.013088 | 1.938336 | 15.095665 |
| C | 1.020293 | 2.085340 | 7.705505 |
| H | 1.893769 | 2.448525 | 7.154448 |
| C | 2.535118 | -4.846179 | 10.736818 |
| C | 1.647755 | -1.195793 | 7.363598 |
| H | 1.020158 | -1.253784 | 6.468466 |
| C | -0.053349 | 1.596150 | 9.846404 |
| C | -2.117421 | 1.024043 | 16.150418 |
| H | -3.074513 | 0.529869 | 16.336167 |
| C | 2.005321 | -4.866276 | 9.293883 |
| H | 2.208228 | -3.928340 | 8.764352 |
| H | 0.919465 | -5.043294 | 9.281298 |
| H | 2.492905 | -5.683365 | 8.732249 |
| C | 3.209712 | -0.998518 | 9.668244 |
| C | -0.304391 | -4.160963 | 14.378965 |
| C | 4.042095 | -4.503472 | 10.684134 |
| H | 4.607009 | -5.290671 | 10.152272 |
| H | 4.474667 | -4.388990 | 11.687781 |
| H | 4.191562 | -3.557202 | 10.146508 |
| C | 2.346323 | 2.480493 | 9.790797 |
| H | 2.713306 | 1.690003 | 10.461294 |
| H | 2.192156 | 3.373631 | 10.415347 |
| H | 3.139046 | 2.696773 | 9.057679 |
| C | -1.027064 | 0.731242 | 16.967362 |
| H | -1.127853 | 0.011337 | 17.784032 |
| C | -1.174527 | 1.109701 | 9.126340 |
| C | 2.937134 | -0.667254 | 7.257045 |
| C | 1.405665 | -2.912609 | 16.222779 |
| H | 2.056507 | -2.391854 | 16.929502 |
| C | 3.695945 | -0.562199 | 8.426677 |
| H | 4.694893 | -0.115450 | 8.374808 |

| | | | |
|---|---|---|---|
| C | -0.283611 | -2.178376 | 8.612312 |
| H | -0.802165 | -1.885658 | 9.536487 |
| H | -0.290847 | -3.280229 | 8.583589 |
| H | -0.866758 | -1.806307 | 7.756423 |
| C | -1.302312 | -4.761724 | 13.390534 |
| H | -0.723317 | -5.153907 | 12.543997 |
| C | 1.092676 | -4.090244 | 14.117824 |
| C | -1.183272 | 1.154847 | 7.723573 |
| H | -2.061684 | 0.780988 | 7.186050 |
| C | 1.950767 | -3.424980 | 15.037493 |
| C | 0.047419 | -3.024584 | 16.504515 |
| H | -0.359077 | -2.602447 | 17.427288 |
| C | 1.788442 | -3.913813 | 11.720262 |
| C | -0.101036 | 1.651409 | 6.992429 |
| C | 2.290345 | -6.246475 | 11.348678 |
| H | 1.361692 | -6.657611 | 10.921235 |
| H | 3.099125 | -6.956275 | 11.111079 |
| C | -0.760546 | 2.566115 | 14.844129 |
| C | -0.796402 | -3.633785 | 15.581711 |
| H | -1.869115 | -3.677498 | 15.785113 |
| C | 0.359097 | 2.243056 | 15.655105 |
| C | 3.781494 | -1.718111 | 14.603905 |
| H | 3.178359 | -1.264973 | 13.806661 |
| H | 4.848250 | -1.598574 | 14.348084 |
| H | 3.591611 | -1.152587 | 15.529819 |
| C | 3.438000 | -3.206927 | 14.773046 |
| H | 3.674084 | -3.695655 | 13.821305 |
| C | -2.114344 | -5.912038 | 14.015501 |
| H | -1.474833 | -6.668204 | 14.492738 |
| H | -2.723746 | -6.415478 | 13.245895 |
| H | -2.809056 | -5.534772 | 14.784091 |
| C | -2.375474 | 0.527957 | 9.836797 |
| H | -2.069490 | -0.245400 | 10.559394 |
| H | -3.084513 | 0.077685 | 9.124205 |
| H | -2.913857 | 1.288796 | 10.421478 |
| C | -3.251009 | 2.234934 | 14.258122 |
| H | -2.976851 | 3.027477 | 13.552325 |
| C | 2.096940 | -6.065928 | 12.859839 |
| C | 0.197196 | 1.340967 | 16.717065 |
| H | 1.059558 | 1.087111 | 17.338601 |
| C | 1.073340 | -7.072943 | 13.409236 |
| H | 0.840263 | -6.863242 | 14.464144 |
| H | 1.484735 | -8.093576 | 13.348690 |
| H | 0.138625 | -7.053958 | 12.835704 |

| | | | |
|---|---|---|---|
| C | 3.409855 | -6.303206 | 13.637359 |
| H | 4.222480 | -5.652232 | 13.294681 |
| H | 3.736342 | -7.348154 | 13.509153 |
| H | 3.256551 | -6.128703 | 14.710658 |
| C | 3.454238 | -0.152366 | 5.936433 |
| H | 3.105016 | -0.771985 | 5.094126 |
| H | 4.555380 | -0.127068 | 5.909434 |
| H | 3.096285 | 0.875650 | 5.748168 |
| C | -0.100544 | 1.642568 | 5.483685 |
| H | -1.122900 | 1.653664 | 5.073146 |
| H | 0.399918 | 0.736670 | 5.096102 |
| H | 0.443481 | 2.508735 | 5.072471 |
| C | -0.124753 | 5.012915 | 10.657111 |
| H | 0.807545 | 5.280015 | 11.172842 |
| H | -0.540760 | 5.932657 | 10.208064 |
| H | 0.123090 | 4.324820 | 9.839191 |
| C | 4.066920 | -0.787083 | 10.897315 |
| H | 3.544576 | -0.147094 | 11.628046 |
| H | 5.025255 | -0.307704 | 10.641129 |
| H | 4.278498 | -1.729200 | 11.424228 |
| C | -3.685969 | 1.015335 | 13.430258 |
| H | -2.852846 | 0.642085 | 12.820090 |
| H | -4.521036 | 1.281068 | 12.759462 |
| H | -4.023476 | 0.190437 | 14.080273 |
| C | -2.406481 | 4.033555 | 10.772728 |
| H | -2.152966 | 3.393912 | 9.917814 |
| H | -2.862411 | 4.959484 | 10.376515 |
| H | -3.163047 | 3.515939 | 11.381538 |
| C | 1.739465 | 2.834453 | 15.402494 |
| H | 1.667850 | 3.423302 | 14.480151 |
| C | -4.421687 | 2.751321 | 15.112560 |
| H | -4.797121 | 1.972766 | 15.796851 |
| H | -5.263580 | 3.056340 | 14.468011 |
| H | -4.131891 | 3.617791 | 15.727490 |
| C | -0.754295 | 4.975682 | 13.982724 |
| C | 2.165409 | 3.761533 | 16.555355 |
| H | 1.401823 | 4.523699 | 16.775040 |
| H | 3.109649 | 4.280231 | 16.317216 |
| H | 2.325450 | 3.184696 | 17.481715 |
| C | -2.266901 | -3.707494 | 12.818654 |
| H | -2.869160 | -3.237716 | 13.613920 |
| H | -2.960814 | -4.179512 | 12.101725 |
| H | -1.719942 | -2.909085 | 12.299034 |
| C | 2.810714 | 1.759662 | 15.163156 |

| | | | |
|---|---|---|---|
| H | 2.973976 | 1.139115 | 16.059554 |
| H | 3.774317 | 2.230949 | 14.904558 |
| H | 2.517527 | 1.090500 | 14.341475 |
| C | 4.323109 | -3.815457 | 15.875821 |
| H | 4.204619 | -3.267311 | 16.825008 |
| H | 5.387599 | -3.754397 | 15.593002 |
| H | 4.083672 | -4.870376 | 16.073304 |
| C | -1.563524 | 5.364952 | 12.733132 |
| H | -1.413374 | 6.415875 | 12.438177 |
| H | -2.635043 | 5.243280 | 12.959764 |
| C | 0.617227 | 5.693799 | 13.978271 |
| H | 1.151237 | 5.555738 | 14.926147 |
| H | 0.479902 | 6.777776 | 13.829889 |
| H | 1.255195 | 5.314699 | 13.168813 |
| C | -1.492265 | 5.330030 | 15.276536 |
| H | -0.945518 | 4.969287 | 16.160814 |
| H | -2.495063 | 4.883875 | 15.297665 |
| H | -1.596012 | 6.424031 | 15.358306 |
| Sn | 0.151080 | -0.605660 | 13.219351 |

## C. References


[1] N. Hurkes, S. Spirk, F. Belaj, R. Pietschnig, *Z. Anorg. Allg. Chem.* **2013**, *639*, 2631–2636.

[2] X. Zhang, L. Ling, M. Luo, X. Zeng, *Angew. Chem. Int. Ed.* **2019**, *58*, 16785–16789.

[3] D. H. Harris, M. F. Lappert, *J. Chem. Soc., Chem. Commun.* **1974**, 895–896.

[4] Y. Wang, A. Kostenko, S. Yao, M. Driess, *J. Am. Chem. Soc.* **2017**, *139*, 13499–13506.

[5] S. K. Kushvaha, P. Kallenbach, S. M. N. V. T. Gorantla, R. Herbst-Irmer, D. Stalke, H. W. Roesky, *Chem. Eur. J.* **2024**, *30*, e202303113.

[6] S. Yao, M. S. Budde, X. Yang, Y. Xiong, L. Zhao, M. Driess, *Angew. Chem. Int. Ed.* **2025**, *64*, e202414696.

[7] Y.-P. Zhou, S. Raoufmoghaddam, T. Szilvási, M. Driess, *Angew. Chem. Int. Ed.* **2016**, *55*, 12868–12872.

[8] Y. Xiong, S. Dong, S. Yao, C. Dai, J. Zhu, S. Kemper, M. Driess, *Angew. Chem. Int. Ed.* **2022**, *61*, e202209250.

[9] Y. Xiong, S. Dong, S. Yao, C. Lorent, K. B. Krause, G. Vijaykumar, J. Zhu, C. Limberg, M. Driess, *Nat. Synth* **2023**, *2*, 678–687.

[10] G. M. Sheldrick, *Acta Crystallogr., Sect. A: Found. Adv.* **2015**, *71*, 3–8.

[11] O. V. Dolomanov, L. J. Bourhis, R. J. Gildea, J. a. K. Howard, H. Puschmann, *J. Appl. Crystallogr.* **2009**, *42*, 339–341.

[12] F. Neese, *WIREs Comput. Mol. Sci.* **2012**, *2*, 73–78.

[13] A. D. Becke, *J. Chem. Phys.* **1993**, *98*, 1372–1377.

[14] S. Grimme, *J. Comput. Chem.* **2006**, *27*, 1787–1799.

[15] S. Grimme, *J. Comput. Chem.* **2004**, *25*, 1463–1473.

[16] F. Weigend, R. Ahlrichs, *Phys. Chem. Chem. Phys.* **2005**, *7*, 3297–3305.

[17] F. Weigend, *Phys. Chem. Chem. Phys.* **2006**, *8*, 1057–1065.

[18] E. D. Glendening, J. K. Badenhoop, A. E. Reed, J. E. Carpenter, J. A. Bohmann, K. Morales, C. R. Landis, F. Weinhold, “NBO 7.0 (Univ. of Wisconsin, 2018)”

[19] Chemcraft, Chemcraft-graphical software for visualization of quantum chemistry computations. Version 1.8, build 682. http://www.chemcraftprog.com

[20] G. Knizia, *J. Chem. Theory Comput.* **2013**, *9*, 4834–4843.

[21] G. Knizia, J. E. M. N. Klein, *Angew. Chem. Int. Ed.* **2015**, *54*, 5518–5522.

[22] W. Humphrey, A. Dalke, K. Schulten, *J. Mol. Graphics* **1996**, *14*, 33–38.

[23] T. Lu, Q. Chen, *J. Phys. Chem. A* **2023**, *127*, 7023–7035.

[24] M. J. Frisch, G. W. Trucks, H. B. Schlegel, G. E. Scuseria, M. A. Robb, J. R. Cheeseman, G. Scalmani, V. Barone, G. A. Petersson, H. Nakatsuji, X. Li, M. Caricato, A. V. Marenich, J. Bloino, B. G. Janesko, R. Gomperts, B. Mennucci, H. P. Hratchian, J. V. Ortiz, A. F. Izmaylov, J. L. Sonnenberg, D. Williams-Young, F. Ding, F. Lipparini, F. Egidi, J. Goings, B. Peng, A. Petrone, T. Henderson, D. Ranasinghe, V. G. Zakrzewski, J. Gao, N. Rega, G. Zheng, W. Liang, M. Hada, M. Ehara, K. Toyota, R. Fukuda, J. Hasegawa, M. Ishida, T. Nakajima, Y. Honda, O. Kitao, H. Nakai, T. Vreven, K. Throssell, J. A. Montgomery, Jr., J. E. Peralta, F. Ogliaro, M. J. Bearpark, J. J. Heyd, E. N. Brothers, K. N. Kudin, V. N. Staroverov, T. A. Keith, R. Kobayashi, J. Normand, K. Raghavachari, A. P. Rendell, J. C. Burant, S. S. Iyengar, J. Tomasi, M. Cossi, J. M. Millam, M. Klene, C. Adamo, R. Cammi, J. W. Ochterski, R. L. Martin, K. Morokuma, O. Farkas, J. B. Foresman, and D. J. Fox Gaussian 16 Rev. C.01 **2016**.

[25] T. Lu, *J. Chem. Phys.* **2024**, *161*, 082503.

[26] T. Lu, F. Chen, *J. Comput. Chem.* **2012**, *33*, 580–592.

[27] A. Poater, F. Ragone, R. Mariz, R. Dorta, L. Cavallo, *Chem. Eur. J.* **2010**, *16*, 14348–14353.

[28] A. Poater, F. Ragone, S. Giudice, C. Costabile, R. Dorta, S. P. Nolan, L. Cavallo, *Organometallics* **2008**, *27*, 2679–2681.

[29] L. Falivene, Z. Cao, A. Petta, L. Serra, A. Poater, R. Oliva, V. Scarano, L. Cavallo, *Nat. Chem.* **2019**, *11*, 872–879.

[30] C. Adamo, V. Barone, *J. Chem. Phys.* **1998**, *108*, 664–675.

[31] C. van Wüllen, *J. Chem. Phys.* **1998**, *109*, 392–399.

[32] E. Van Lenthe, J. G. Snijders, E. J. Baerends, *J. Chem. Phys.* **1996**, *105*, 6505–6516.

[33] E. van Lenthe, E. J. Baerends, J. G. Snijders, *J. Chem. Phys.* **1994**, *101*, 9783–9792.

[34] E. van Lenthe, E. J. Baerends, J. G. Snijders, *J. Chem. Phys.* **1993**, *99*, 4597–4610.

[35] D. A. Pantazis, X.-Y. Chen, C. R. Landis, F. Neese, *J. Chem. Theory Comput.* **2008**, *4*, 908–919.

[36] F. Neese, F. Wennmohs, A. Hansen, U. Becker, *Chem. Phys.* **2009**, *356*, 98–109.

[37] F. Neese, *J. Chem. Phys.* **2005**, *122*, 34107.

[38] V. Barone, M. Cossi, *J. Phys. Chem. A* **1998**, *102*, 1995–2001.

[39] S. Miertuš, E. Scrocco, J. Tomasi, *Chem. Phys.* **1981**, *55*, 117–129.
